# Supplementary material for: Copper-catalysed asymmetric reductive cross-coupling of prochiral alkenes
Source: Nat Commun. 2022 May 11;13:2570. doi: 10.1038/s41467-022-30286-8 (PMC9095606; doi:10.1038/s41467-022-30286-8)
Supplement: Supplementary file 1 — Supplementary Information [file 41467_2022_30286_MOESM1_ESM.pdf]

Supplementary Information

Copper-Catalysed Asymmetric Reductive Cross Coupling of Prochiral Alkenes

Wan Seok Yoon[1,2], Won Jun Jang[1,2], Woojin Yoon[3], Hoseop Yun[3]*
and Jaesook Yun[1]*

[1]Department of Chemistry and Institute of Basic Science
Sungkyunkwan University, Suwon 16419, Korea
[2]These authors contributed equally: Wan Seok Yoon, Won Jun Jang
[3]Department of Energy Systems Research and Department of Chemistry
Ajou University, Suwon 16499, Korea
Fax: (+82)-31-290-7075; *e-mail: hsyun@ajou.ac.kr; jaesook@skku.edu

Ⅰ. Supplementary Notes

CuCl, LiO*t*-Bu and other commercial reagents were purchased from Aldrich and used as received. Commercial chiral ligands were purchased from Strem. Chiral NHC ligand **L2**,[1,2] enyne (**1** and **4**)[3,4] and alkylidene diesters (**2**)[5,6] were prepared by following literature procedures. Tetrahydrofuran (THF) was purified using PureSolv solvent purification system, from Innovative Technology, Inc. Reactions with oxygen- and moisture-sensitive materials were carried out with the standard Schlenk technique. Flash chromatography was performed on silica gel from Merck (70–230 mesh). All ^{1}H NMR spectra were obtained on Bruker at 500 systems and reported in parts per million (ppm) downfield from tetramethylsilane. ^{13}C NMR spectra were reported in ppm referenced to deuteriochloroform (77.16 ppm). High performance liquid chromatography (HPLC) was performed using Younglin Acme 9100 series. Infrared spectra (IR) were obtained on Nicolet 205 FT-IR and were recorded in cm^{-1}. Optical rotation was measured with Model 343 plus polarimeter equipped with a sodium lamp source (589 nm). High resolution mass spectra (HRMS) were obtained at Korea Basic Science Institute (Cheongju, Korea) or Korea Basic Science Institute (Daegu, Korea) and reported in the form of *m/z* (intensity relative to peak = 100). X-ray structural analysis was supported by Ajou University.

Ⅱ. Supplementary Methods

General Procedure for the Cu-Catalysed Coupling of Terminal Enyne (1) with 2.

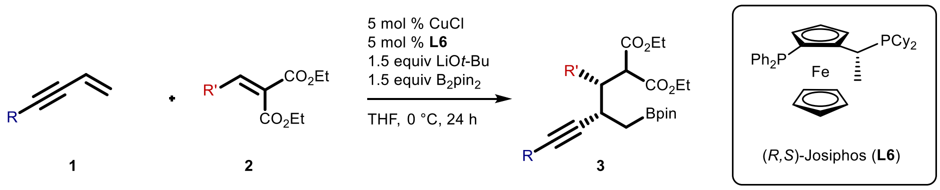

A mixture of CuCl (5 mol %, 0.025 mmol), **L6** (5 mol %, 0.025 mmol), LiO*t*-Bu (1.5 equiv, 0.75 mmol), and B_2pin_2 (1.5 equiv, 0.75 mmol) in THF (0.7 mL) was stirred for 15 min in a Schlenk tube under an atmosphere of nitrogen. Substrate **1** (1.5 equiv, 0.75 mmol) and **2** (1 equiv, 0.5 mmol) dissolved in THF (0.3 mL) were added to the reaction mixture at 0 °C. The reaction mixture was stirred at 0 °C and monitored by TLC. Upon complete consumption of **2**, the reaction mixture was diluted with water (3 mL) and extracted with dichloromethane (5 mL x 3). The combined organic layers were washed with brine, dried over $MgSO_4$, and concentrated in vacuo. The residue was purified by column on silica gel using ethyl acetate/hexane as eluent.

Characterization of compound 3.

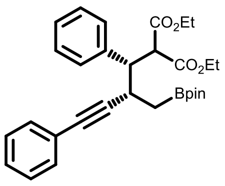

3a

Diethyl 2-(1,4-diphenyl-2-((4,4,5,5-tetramethyl-1,3,2-dioxaborolan-2-yl)methyl)but-3-yn-1-yl)malonate (3a): By following the general procedure, **3a** was obtained in 94% yield in >98:2 diastereomeric ratio (white solid). ^{1}H NMR (500 MHz, $CDCl_3$) δ 7.42–7.38 (m, 4H), 7.32–7.29 (m, 3H), 7.25–7.19 (m, 3H), 4.31–4.25 (m, 2H), 4.25 (d, *J* = 11.7 Hz, 1H), 3.89–3.79 (m. 2H), 3.52 (dd, *J* = 11.7, 4.2 Hz, 1H), 3.38 (ddd, *J* = 10.2, 6.1, 4.3 Hz, 1H), 1.31 (t, *J* = 7.1 Hz, 3H), 1.22 (s, 6H), 1.21 (s, 6H), 0.91–0.82 (m, 2H), 0.87 (t, *J* = 7.1 Hz, 3H); ^{13}C NMR (125 MHz, $CDCl_3$) δ 168.3, 167.9, 137.3, 131.5, 129.9, 128.2, 127.7, 127.7, 127.3, 123.9, 91.1, 84.1, 83.3, 61.6, 61.1, 56.5, 49.5, 30.6, 25.0, 24.6, 14.1, 13.6. The carbon bound to the boron was not detected due to quadrupolar relaxation; IR (neat) 2980, 1751, 1732, 1369, 1143, 755, 697, 667 cm^{-1}; HRMS (ESI) calcd for [$C_{30}H_{37}BO_6$+Na^+]: 527.2581, found: 527.2581; 94% ee was measured by chiral HPLC on IA column (*i*-PrOH:hexanes = 5:95, 0.5 mL/min, wavelength = 254 nm, 20 °C); t_R = 10.12 min (major), t_R = 8.56 min (minor); $[\alpha]_D^{20}$ = –137.9 (c = 1.0, $CHCl_3$).

<rac-3a>

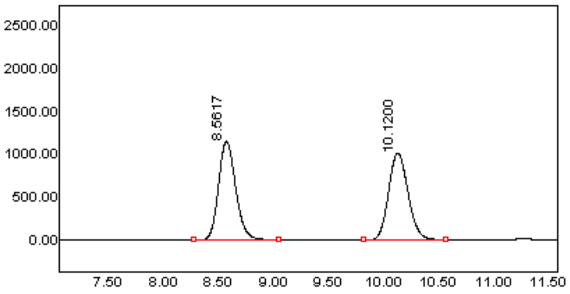

<chiral-3a>

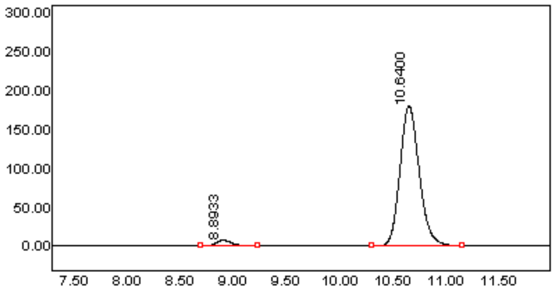

time (min) ↓ area ↓ area ratio ↓

RT[분]	면적[mV*sec]	면적비[%]
8.5617	12422.8633	50.06
10.1200	12391.6219	49.94
	24814.4844	

RT[분]	면적[mV*sec]	면적비[%]
8.8933	72.5637	3.10
10.6400	2271.6203	96.90
	2344.1840	

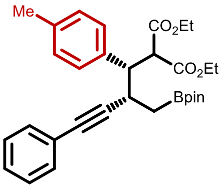

3b

Diethyl 2-(4-phenyl-2-((4,4,5,5-tetramethyl-1,3,2-dioxaborolan-2-yl)methyl)-1-(p-tolyl)but-3-yn-1-yl)malonate (3b): By following the general procedure, **3b** was obtained in 94% yield in >98:2 diastereomeric ratio (colorless oil). ^{1}H NMR (500 MHz, $CDCl_3$) δ 7.41–7.39 (m, 2H), 7.31–7.28 (m, 3H), 7.27–7.25 (m, 2H), 7.05–7.04 (m, 2H), 4.31–4.25 (m, 2H), 4.23 (d, *J* = 11.5 Hz, 1H), 3.90–3.81 (m, 2H), 3.47 (dd, *J* = 11.5, 4.0 Hz, 1H), 3.37–3.33 (m, 1H), 2.28 (s, 3H), 1.31 (t, *J* = 7.0 Hz, 3H), 1.22 (s,

6H), 1.21 (s, 6H), 0.90 (t, *J* = 7.0 Hz, 3H), 0.86–0.84 (m, 2H); ^{13}C NMR (125 MHz, $CDCl_3$) δ 168.4, 168.0, 136.7, 134.2, 131.5, 129.7, 128.4, 128.2, 127.6, 124.0, 91.2, 84.0, 83.3, 61.6, 61.1, 56.5, 49.1, 30.6, 25.0, 24.6, 21.1, 17.1 (C-B), 14.1, 13.7; IR (neat) 2980, 1731, 1369, 1143, 753, 692, 667 cm^{-1}; HRMS (ESI) calcd for [$C_{31}H_{39}BO_6$+Na^+]: 541.2737, found: 541.2737; 91% ee was measured by chiral HPLC on IA column (*i*-PrOH:hexanes = 5:95, 0.5 mL/min, wavelength = 254 nm, 20 °C); t_R = 10.17 min (major), t_R = 8.29 min (minor); $[\alpha]_D^{20}$ = –224.2 (c = 1.5, $CHCl_3$).

<rac-3b>

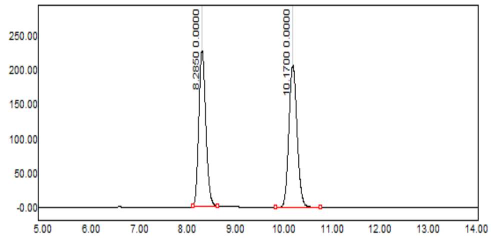

RT[분]	면적[mV*sec]	면적비[%]
8.2850	2292.4215	48.12
10.1700	2471.5549	51.88
	4763.9766	

<chiral-3b>

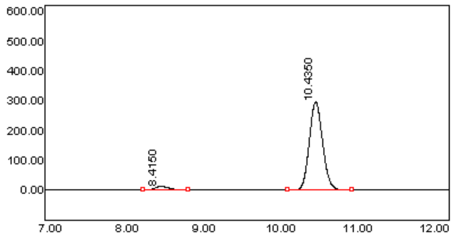

RT[분]	면적[mV*sec]	면적비[%]
8.4150	167.6907	4.46
10.4350	3590.1406	95.54
	3757.8313	

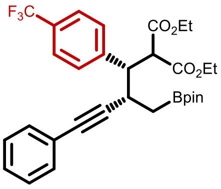

3c

Diethyl 2-(4-phenyl-2-((4,4,5,5-tetramethyl-1,3,2-dioxaborolan-2-yl)methyl)-1-(4-(trifluoromethyl)phenyl)but-3-yn-1-yl)malonate (3c): By following the general procedure, **3c** was obtained in 90% yield in >98:2 diastereomeric ratio (colorless oil). ^{1}H NMR (500 MHz, $CDCl_3$) δ 7.53 (brs, 4H), 7.41–7.39 (m, 2H), 7.32–7.30 (m, 3H), 4.33–4.27 (m, 2H), 4.25 (d, *J* = 12.0 Hz, 1H), 3.89–3.83 (m, 2H), 3.60 (dd, *J* = 12.0, 4.0 Hz, 1H), 3.43–3.39 (m, 1H), 1.32 (t, *J* = 7.0 Hz, 3H), 1.23 (s, 6H), 1.22 (s, 6H), 0.90–0.76 (m, 5H); ^{13}C NMR (125 MHz, $CDCl_3$) δ 167.9, 167.6, 141.7, 131.5, 130.1, 129.6 (q, *J* = 32.5 Hz), 128.3, 128.0, 126.4 (q, *J* = 271 Hz), 124.7 (q, *J* = 3.8 Hz), 123.5, 90.3, 84.6, 83.4, 61.9, 61.3, 56.2, 49.2, 30.5, 25.0, 24.6, 17.1 (C-B), 14.1, 13.6; IR (neat) 3020, 1730, 1325, 1216, 1127, 754, 691, 667 cm^{-1}; HRMS (ESI) calcd for [$C_{31}H_{36}BF_3O_6$+Na^+]: 595.2455, found: 595.2456; 94% ee was measured by chiral HPLC on IA column (*i*-PrOH:hexanes = 5:95, 0.5 mL/min, wavelength = 254 nm, 20 °C); t_R = 11.83 min (major), t_R = 7.69 min (minor); $[\alpha]_D^{20}$ = –155.7 (c = 1.3, $CHCl_3$).

<rac-3c>

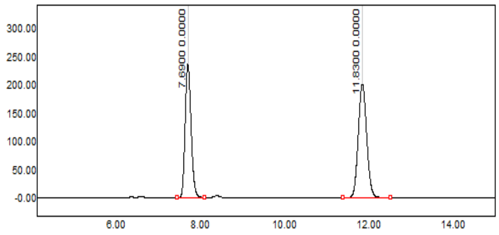

<chiral-3c>

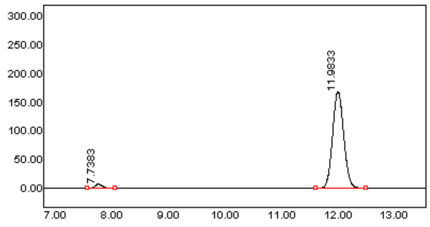

RT[분]	면적[mV*sec]	면적비[%]
7.6900	2318.5246	45.21
11.8300	2809.3584	54.79
	5127.8828	

RT[분]	면적[mV*sec]	면적비[%]
7.7383	70.2849	2.95
11.9833	2308.7420	97.05
	2379.0270	

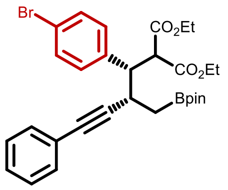

3d

Diethyl 2-(1-(4-bromophenyl)-4-phenyl-2-((4,4,5,5-tetramethyl-1,3,2-dioxaborolan-2-yl)methyl)but-3-yn-1-yl)malonate (3d): By following the general procedure, **3d** was obtained in 91% yield in >98:2 diastereomeric ratio (colorless oil). ^{1}H NMR (500 MHz, $CDCl_3$) δ 7.40–7.38 (m, 4H), 7.31–7.27 (m, 5H), 4.30–4.26 (m, 2H), 4.19 (d, J = 11.5 Hz, 1H), 3.91–3.84 (m, 2H), 3.50 (dd, J = 11.5, 4.0 Hz, 1H), 3.38–3.34 (m, 1H), 1.31 (t, J = 7.0 Hz, 3H), 1.222 (s, 6H), 1.215 (s, 6H), 0.93 (t, J = 7.0 Hz, 3H), 0.88–0.79 (m, 2H); ^{13}C NMR (125 MHz, $CDCl_3$) δ 168.0, 167.7, 136.5, 131.51, 131.48, 130.9, 128.3, 127.9, 123.6, 121.4, 90.6, 84.4, 83.4, 61.8, 61.3, 56.2, 48.9, 30.5, 25.0, 24.6, 17.0 (C-B), 14.1, 13.7; IR (neat) 3019, 1729, 1370, 1263, 1143, 752, 691, 667 cm^{-1}; HRMS (ESI) calcd for [$C_{30}H_{36}BBrO_6$+Na^+]: 605.1686, found: 605.1686; 95% ee was measured by chiral HPLC on IA column (*i*-PrOH:hexanes = 5:95, 0.5 mL/min, wavelength = 254 nm, 20 °C); t_R = 12.69 min (major), t_R = 8.68 min (minor); $[\alpha]_D^{20}$ = –163.9 (c = 1.3, $CHCl_3$).

<rac-3d>

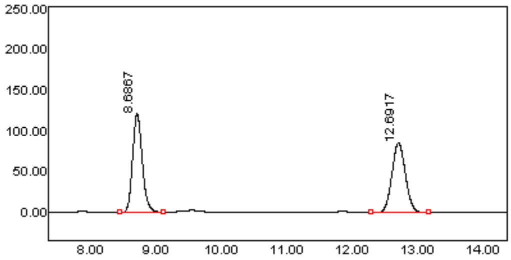

<chiral-3d>

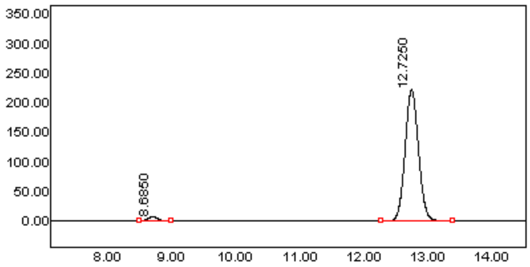

RT[분]	면적[mV*sec]	면적비[%]
8.6867	1250.5408	50.10
12.6917	1245.2996	49.90
	2495.8404	

RT[분]	면적[mV*sec]	면적비[%]
8.6850	78.3524	2.37
12.7250	3232.6619	97.63
	3311.0145	

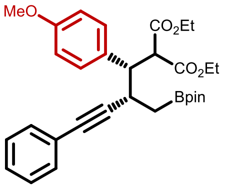

3e

Diethyl 2-(1-(4-methoxyphenyl)-4-phenyl-2-((4,4,5,5-tetramethyl-1,3,2-dioxaborolan-2-yl)methyl)but-3-yn-1-yl)malonate (3e): By following the general procedure, **3e** was obtained in 88% yield in >98:2 diastereomeric ratio (colorless oil). ^{1}H NMR (500 MHz, $CDCl_3$) δ 7.41–7.39 (m, 2H), 7.31–7.29 (m, 5H), 6.80–6.78 (m, 2H), 4.30–4.24 (m, 2H), 4.20 (d, J = 11.5 Hz, 1H), 3.91–3.81 (m, 2H), 3.76 (s, 3H), 3.47 (dd, J = 11.5, 4.0 Hz, 1H), 3.37–3.32 (m, 1H), 1.31 (t, J = 7.0 Hz, 3H), 1.221 (s, 6H), 1.216 (s, 6H), 0.91 (t, J = 7.0 Hz, 3H), 0.86–0.85 (m, 2H); ^{13}C NMR (125 MHz, $CDCl_3$) δ 168.4, 168.0,

158.1, 131.5, 130.8, 129.4, 128.2, 127.7, 123.9, 113.1, 91.2, 84.1, 83.3, 61.6, 61.1, 56.6, 55.2, 48.7, 30.7, 25.0, 24.6, 14.1, 13.7. The carbon bound to the boron was not detected due to quadrupolar relaxation; IR (neat) 3020, 1729, 1370, 1219, 1143, 772, 687, 670 cm^{-1}; HRMS (ESI) calcd for [$C_{31}H_{39}BO_7$+Na^+]: 557.2687, found: 557.2687; 93% ee was measured by chiral HPLC on IA column (*i*-PrOH:hexanes = 5:95, 0.5 mL/min, wavelength = 254 nm, 20 °C); t_R = 14.80 min (major), t_R = 9.67 min (minor); $[\alpha]_D^{20}$ = –202.2 (c = 1.5, $CHCl_3$).

<rac-3e>

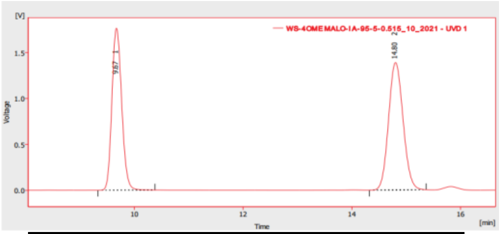

Reten. Time [min]	Area [mV.s]	Area [%]
9.670	22006.696	46.1
14.803	25721.069	53.9
Total	47727.765	100.0

<chiral-3e>

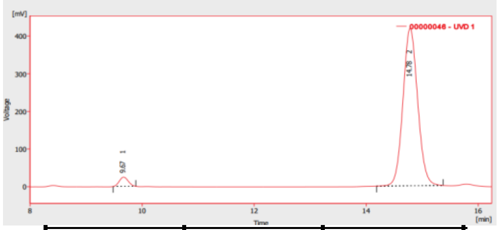

Reten. Time [min]	Area [mV.s]	Area [%]
9.670	273.194	3.5
14.783	7621.209	96.5
Total	7894.403	100.0

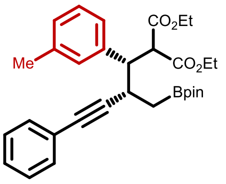

3f

Diethyl 2-(4-phenyl-2-((4,4,5,5-tetramethyl-1,3,2-dioxaborolan-2-yl)methyl)-1-(m-tolyl)but-3-yn-1-yl)malonate (3f): By following the general procedure, **3f** was obtained in 89% yield in >98:2 diastereomeric ratio (yellow solid). ^{1}H NMR (500 MHz, $CDCl_3$) δ 7.41–7.39 (m, 2H), 7.31–7.28 (m, 3H), 7.19–7.17 (m, 2H), 7.14–7.11 (m, 1H), 7.02–7.00 (m, 1H), 4.31–4.26 (m, 2H), 4.24 (d, *J* = 11.5 Hz, 1H), 3.88–3.82 (m, 2H), 3.48 (dd, *J* = 11.5, 4.0 Hz, 1H), 3.38–3.34 (m, 1H), 2.29 (s, 3H), 1.31 (t, *J* = 7.0 Hz, 3H), 1.221 (s, 6H), 1.216 (s, 6H), 0.89–0.86 (m, 5H); ^{13}C NMR (125 MHz, $CDCl_3$) δ 168.4, 167.9, 137.2, 136.9, 131.5, 130.7, 129.1, 128.2, 127.9, 127.63, 127.55, 126.9, 124.0, 91.2, 84.0, 83.3, 61.6, 61.1, 56.4, 49.4, 30.6, 25.0, 24.6, 21.5, 14.1, 13.6. The carbon bound to the boron was not detected due to quadrupolar relaxation; IR (neat) 2981, 1728, 1370, 1218, 1143, 771, 753, 691, 667 cm^{-1}; HRMS (ESI) calcd for [$C_{31}H_{39}BO_6$+Na^+]: 541.2737, found: 541.2738; 95% ee was measured by chiral HPLC on IA column (*i*-PrOH:hexanes = 5:95, 0.5 mL/min, wavelength = 254 nm, 20 °C); t_R = 9.80 min (major), t_R = 8.12 min (minor); $[\alpha]_D^{20}$ = –172.7 (c = 1.3, $CHCl_3$).

<rac-3f>

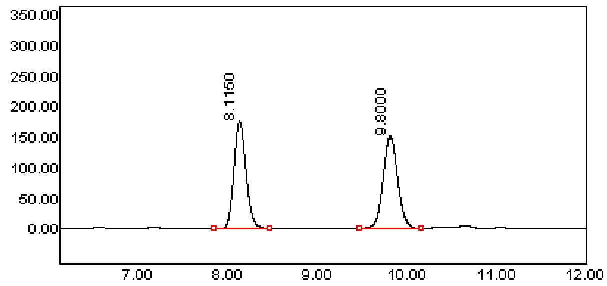

RT[분]	면적[mV*sec]	면적비[%]
8.1150	1695.1031	49.67
9.8000	1717.8695	50.33
	3412.9727	

<chiral-3f>

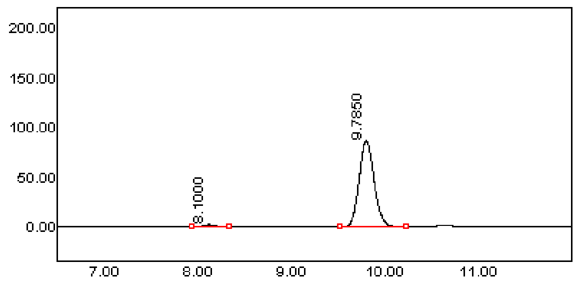

RT[분]	면적[mV*sec]	면적비[%]
8.1000	23.1871	2.32
9.7850	975.4108	97.68
	998.5979	

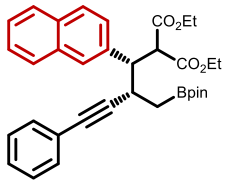

3g

Diethyl 2-(1-(naphthalen-2-yl)-4-phenyl-2-((4,4,5,5-tetramethyl-1,3,2-dioxaborolan-2-yl)methyl)but-3-yn-1-yl)malonate (3g): By following the general procedure, **3g** was obtained in 92% yield in >98:2 diastereomeric ratio (colorless oil). ^{1}H NMR (500 MHz, $CDCl_3$) δ 7.83 (s, 1H), 7.79–7.73 (m, 3H), 7.59–7.58 (m, 1H), 7.44–7.42 (m, 4H), 7.34–7.31 (m, 3H), 4.38 (d, J = 12.0 Hz, 1H), 4.34–4.28 (m, 2H), 3.81–3.73 (m, 2H), 3.71 (dd, J = 12.0, 4.0 Hz, 1H), 3.47–3.43 (m, 1H), 1.33 (t, J = 7.0 Hz, 3H), 1.211 (s, 6H), 1.206 (s, 6H), 0.96–0.82 (m, 2H), 0.77 (t, J = 7.0 Hz, 3H); ^{13}C NMR (125 MHz, $CDCl_3$) δ 168.3, 167.9, 135.1, 133.0, 132.8, 131.6, 128.9, 128.2, 128.0, 127.9, 127.7, 127.5, 127.2, 125.7, 125.6, 123.9, 91.1, 84.3, 83.3, 61.7, 61.1, 56.5, 49.5, 30.8, 25.0, 24.6, 17.0 (C-B), 14.1, 13.6; IR (neat) 3019, 1746, 1729, 1370, 1218, 1143, 772, 689, 669 cm^{-1}; HRMS (ESI) calcd for [$C_{34}H_{39}BO_6$+Na^+]: 577.2737, found: 577.2736; 94% ee was measured by chiral HPLC on IA column (*i*-PrOH:hexanes = 5:95, 0.5 mL/min, wavelength = 254 nm, 20 °C); t_R = 16.70 min (major), t_R = 11.09 min (minor); $[\alpha]_D^{20}$ = –215.1 (c = 1.5, $CHCl_3$).

<rac-3g>

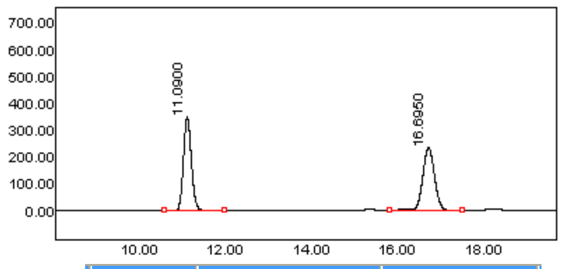

RT[분]	면적[mV*sec]	면적비[%]
11.0900	4506.6687	49.39
16.6950	4618.4199	50.61
	9125.0891	

<chiral-3g>

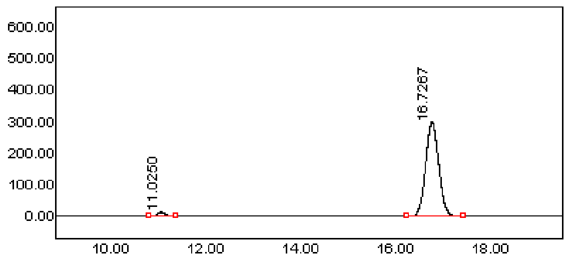

RT[분]	면적[mV*sec]	면적비[%]
11.0250	166.1465	2.82
16.7267	5721.7027	97.18
	5887.8492	

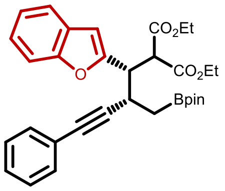

3h

Diethyl 2-(1-(benzofuran-2-yl)-4-phenyl-2-((4,4,5,5-tetramethyl-1,3,2-dioxaborolan-2-yl)methyl)but-3-yn-1-yl)malonate (3h): By following the general procedure, **3h** was obtained in 86% yield in >98:2 diastereomeric ratio (colorless oil). ^{1}H NMR (500 MHz, $CDCl_3$) δ 7.49–7.48 (m, 1H), 7.42–7.39 (m, 3H), 7.31–7.29 (m, 3H), 7.22–7.15 (m, 2H), 6.65 (s, 1H), 4.31–4.27 (m, 3H), 4.00–3.88 (m, 3H), 3.40–3.36 (m, 1H), 1.32 (t, *J* = 7.0 Hz, 3H), 1.24 (s, 12H), 1.07–1.06 (m, 2H), 0.92 (t, *J* = 7.0 Hz, 3H); ^{13}C NMR (125 MHz, $CDCl_3$) δ 167.7, 167.6, 155.5, 154.5, 131.6, 128.4, 128.2, 127.8, 123.8, 123.6, 122.5, 120.7, 111.0, 105.0, 90.3, 83.8, 83.4, 61.8, 61.5, 55.2, 44.1, 30.4, 25.0, 24.6, 14.1, 13.7. The carbon bound to the boron was not detected due to quadrupolar relaxation; IR (neat) 3019, 1731, 1370, 1218, 1143, 772, 689, 669 cm^{-1}; HRMS (ESI) calcd for [$C_{32}H_{37}BO_7$+Na^+]: 567.2530, found: 567.2531; 95% ee was measured by chiral HPLC on IA column (*i*-PrOH:hexanes = 5:95, 0.5 mL/min, wavelength = 254 nm, 20 °C); t_R = 14.28 min (major), t_R = 10.60 min (minor); $[\alpha]_D^{20}$ = –197.5 (c = 1.3, $CHCl_3$).

<rac-3h>

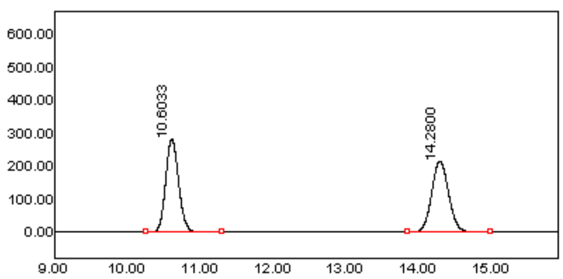

RT[분]	면적[mV*sec]	면적비[%]
10.6033	3489.2793	49.93
14.2800	3499.2359	50.07
	6988.5156	

<chiral-3h>

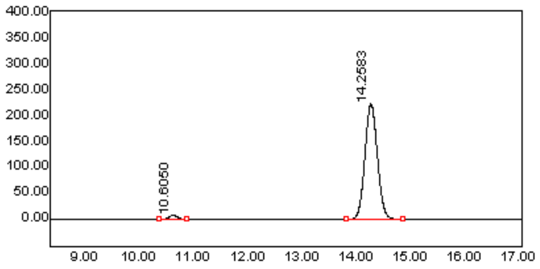

RT[분]	면적[mV*sec]	면적비[%]
10.6050	95.2793	2.59
14.2583	3588.4863	97.41
	3683.7656	

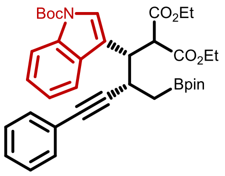

3i

Diethyl 2-(1-(1-(tert-butoxycarbonyl)-1H-indol-3-yl)-4-phenyl-2-((4,4,5,5-tetramethyl-1,3,2-dioxaborolan-2-yl)methyl)but-3-yn-1-yl)malonate (3i): By following the general procedure, **3i** was obtained in 71% yield in 90:10 diastereomeric ratio (white solid). ^{1}H NMR (500 MHz, $CDCl_3$) δ 8.11 (brs, 1H), 7.72–7.63 (m, 2H), 7.46–7.44 (m, 2H), 7.32–7.27 (m, 3H), 7.25–7.19 (m, 2H), 4.33–4.26 (m, 2H), 4.20 (d, *J* = 11.5 Hz, 1H), 3.95 (dd, 11.5, 4.0 Hz, 1H), 3.84–3.74 (m, 2H), 3.44–3.40 (m, 1H), 1.58 (s, 9H), 1.32 (t, *J* = 7.0 Hz, 3H), 1.20 (s, 6H), 1.19 (s, 6H), 1.01–0.87 (m, 2H), 0.79 (t, *J* = 7.0 Hz, 3H); ^{13}C NMR (125 MHz, $CDCl_3$) δ 168.2, 167.7, 149.6, 131.6, 131.5, 128.2, 128.1, 127.8, 124.2, 123.7, 122.4, 117.2, 114.9, 91.3, 83.9, 83., 83.2, 61.7, 61.2, 57.1, 31.4, 28.2, 28.1, 25.0, 24.5, 17.1 (C-B), 14.1, 13.5; IR (neat) 3020, 1730, 1371, 1219, 1154, 772, 687, 669 cm^{-1}; HRMS (ESI) calcd for [$C_{37}H_{46}BNO_8$+Na^+]: 666.3214, found: 666.3214; 93% ee was measured by chiral HPLC on IA column (*i*-PrOH:hexanes = 5:95, 0.5 mL/min, wavelength = 254 nm, 20 °C); t_R = 18.45 min (major), t_R = 17.64 min (minor); $[\alpha]_D^{20}$ =

–113.1 (c = 1.0, $CHCl_3$).

<rac-3i>

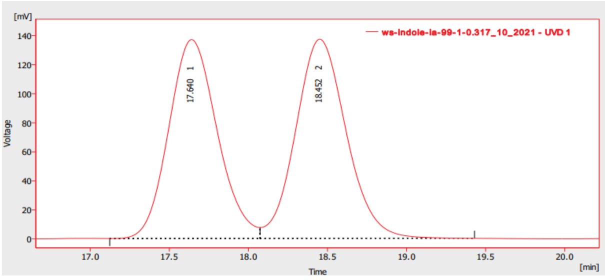

<chiral-3i>

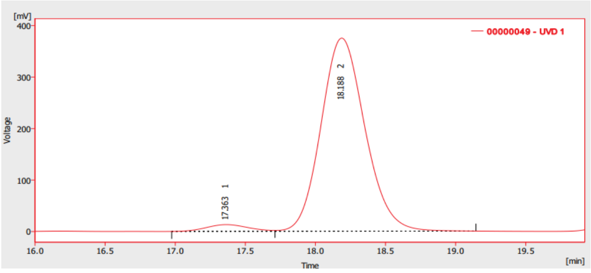

Reten. Time [min]	Area [mV.s]	Area [%]
17.640	2909.115	48.9
18.452	3044.619	51.1
Total	5953.734	100.0

Reten. Time [min]	Area [mV.s]	Area [%]
17.363	275.037	3.3
18.188	8178.319	96.7
Total	8453.356	100.0

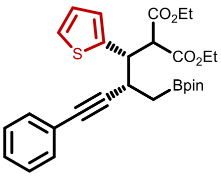

3j

Diethyl 2-(4-phenyl-2-((4,4,5,5-tetramethyl-1,3,2-dioxaborolan-2-yl)methyl)-1-(thiophen-2-yl)but-3-yn-1-yl)malonate (3j): By following the general procedure, **3j** was obtained in 88% yield in >98:2 diastereomeric ratio (colorless oil). ^{1}H NMR (500 MHz, $CDCl_3$) δ 7.47–7.45 (m, 2H), 7.33–7.29 (m, 3H), 7.17–7.16 (m, 1H), 7.00–6.99 (m, 1H), 6.90–6.89 (m, 1H), 4.27 (q, J = 7.0 Hz, 2H), 4.15 (d, J = 11.5 Hz, 1H), 3.94–3.89 (m, 3H), 3.40–3.36 (m, 1H), 1.30 (t, J = 7.0 Hz, 3H), 1.24 (s, 6H), 1.23 (s, 6H), 1.04–0.89 (m, 5H); ^{13}C NMR (125 MHz, $CDCl_3$) δ 167.8, 167.7, 139.5, 131.6, 128.2, 127.8, 127.6, 125.9, 124.8, 123.8, 90.8, 84.6, 83.3, 61.7, 61.3, 57.9, 45.6, 30.8, 25.0, 24.6, 16.8 (C-B), 14.1, 13.7; IR (neat) 3020, 1730, 1219, 1143, 772, 698, 670 cm^{-1}; HRMS (ESI) calcd for [$C_{28}H_{35}BSO_6$+Na^+]: 533.2145, found: 533.2145; 95% ee was measured by chiral HPLC on IA column (i-PrOH:hexanes = 5:95, 0.5 mL/min, wavelength = 254 nm, 20 °C); t_R = 9.84 min (major), t_R = 8.81 min (minor); $[\alpha]_D^{20}$ = –145.9 (c = 1.2, $CHCl_3$).

<rac-3j>

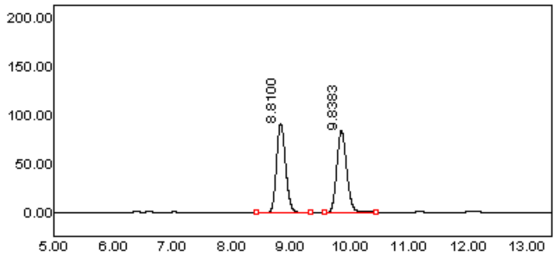

RT[분]	면적[mV*sec]	면적비[%]
8.8100	980.0191	49.43
9.8383	1002.6851	50.57
	1982.7043	

<chiral-3j>

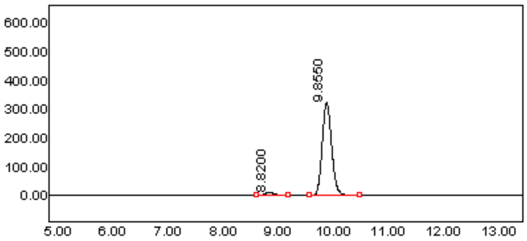

RT[분]	면적[mV*sec]	면적비[%]
8.8200	104.3798	2.64
9.8550	3852.8328	97.36
	3957.2125	

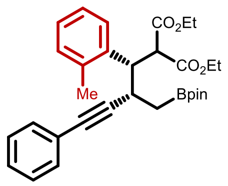

3k

Diethyl 2-(4-phenyl-2-((4,4,5,5-tetramethyl-1,3,2-dioxaborolan-2-yl)methyl)-1-(o-tolyl)but-3-yn-1-yl)malonate (3k): By following the general procedure, **3k** was obtained in 68% yield in >98:2 diastereomeric ratio (white solid). ^{1}H NMR (500 MHz, $CDCl_3$) δ 7.65–7.63 (m, 1H), 7.42–7.40 (m, 2H), 7.31–7.29 (m, 3H), 7.12–7.07 (m, 3H), 4.31–4.25 (m, 2H), 4.22 (d, J = 11.5 Hz, 1H), 3.93 (dd, J = 11.5, 4.0 Hz, 1H), 3.80 (q, J = 7.0 Hz, 2H), 3.46–3.42 (m, 1H), 2.40 (s, 3H), 1.31 (t, J = 7.0 Hz, 3H), 1.201 (s, 6H), 1.197 (s, 6H), 0.96–0.79 (m, 5H); ^{13}C NMR (125 MHz, $CDCl_3$) δ 168.5, 168.0, 137.7, 136.2, 131.5, 130.1, 128.2, 128.1, 127.7, 126.8, 125.5, 123.9, 91.4, 84.3, 83.3, 61.6, 61.1, 56.9, 43.0, 31.2, 25.0, 24.5, 20.6, 14.1, 13.5. The carbon bound to the boron was not detected due to quadrupolar relaxation; IR (neat) 3021, 1729, 1369, 1219, 1143, 772, 687, 671 cm^{-1}; HRMS (ESI) calcd for [$C_{31}H_{39}BO_6$+Na^+]: 541.2737, found: 541.2737; 87% ee was measured by chiral HPLC on IA column (i-PrOH:hexanes = 1:99, 0.5 mL/min, wavelength = 254 nm, 20 °C); t_R = 10.51 min (major), t_R = 11.17 min (minor); $[\alpha]_D^{20}$ = –159.0 (c = 1.3, $CHCl_3$).

<rac-3k>

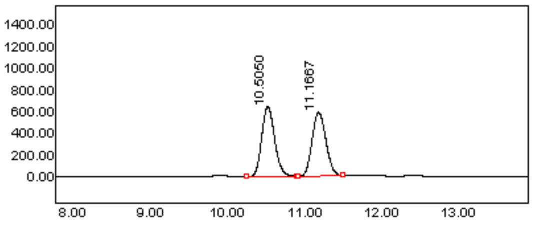

RT[분]	면적[mV*sec]	면적비[%]
10.5050	7294.1945	50.05
11.1667	7279.5703	49.95
	14573.7656	

<chiral-3k>

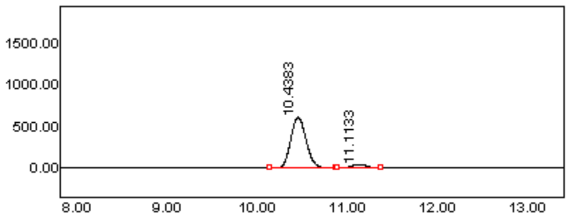

RT[분]	면적[mV*sec]	면적비[%]
10.4383	7266.8555	93.43
11.1133	510.6914	6.57
	7777.5469	

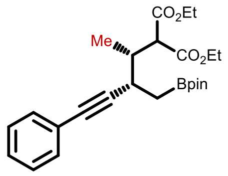

3l

Diethyl 2-(5-phenyl-3-((4,4,5,5-tetramethyl-1,3,2-dioxaborolan-2-yl)methyl)pent-4-yn-2-yl)malonate (3l): By following the general procedure, **3l** was obtained in 64% yield in >98:2 diastereomeric ratio (colorless oil). ^{1}H NMR (500 MHz, $CDCl_3$) δ 7.39–7.37 (m, 2H), 7.30–7.28 (m, 3H), 4.24–4.17 (m, 4H), 3.54 (d, J = 10.0 Hz, 1H), 3.07–3.03 (m, 1H), 2.43–2.35 (m, 1H), 1.29–1.25 (m, 20H), 1.06 (d, J = 6.8 Hz, 3H); ^{13}C NMR (125 MHz, $CDCl_3$) δ 168.8, 168.6, 131.6, 128.1, 127.5, 123.9, 90.7, 83.5, 83.3, 61.3, 61.2, 57.0, 38.2, 31.0, 25.0, 24.7, 14.10, 14.05, 12.4. The carbon bound to the boron was not detected due to quadrupolar relaxation; IR (neat) 3021, 1727, 1370, 1219, 1143, 772, 687, 670 cm^{-1}; HRMS (ESI) calcd for [$C_{25}H_{35}BO_6$+Na^+]: 465.2424, found: 465.2425; 88% ee was measured by chiral HPLC on IA column (i-PrOH:hexanes = 5:95, 0.5 mL/min, wavelength = 254 nm, 20 °C); t_R = 10.19 min (major), t_R = 9.46 min (minor); $[\alpha]_D^{20}$ = –22.5 (c = 0.8, $CHCl_3$).

<rac-3l>

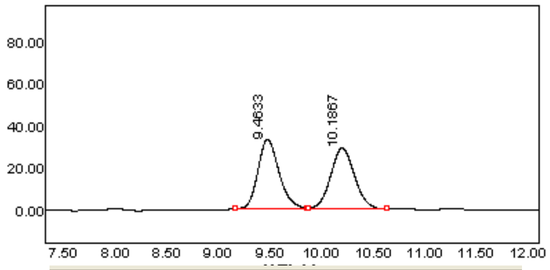

RT[분]	면적[mV*sec]	면적비[%]
9.4633	461.5679	50.23
10.1867	457.3876	49.77
	918.9555	

<chiral-3l>

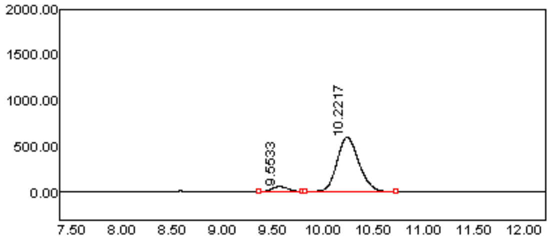

RT[분]	면적[mV*sec]	면적비[%]
9.5533	631.8943	6.54
10.2217	9024.9156	93.46
	9656.8102	

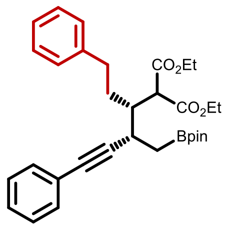

3m

Diethyl 2-(1,6-diphenyl-4-((4,4,5,5-tetramethyl-1,3,2-dioxaborolan-2-yl)methyl)hex-5-yn-3-yl)malonate (3m): By following the general procedure, **3m** was obtained in 71% yield in >98:2 diastereomeric ratio (colorless oil). ^{1}H NMR (500 MHz, $CDCl_3$) δ 7.36–7.35 (m, 2H), 7.27–7.24 (m, 5H), 7.18–7.14 (m, 3H), 4.22–4.18 (m, 4H), 3.72 (d, J = 8.5 Hz, 1H), 3.15–3.13 (m, 1H), 2.82–2.67 (m, 2H), 2.43–2.39 (m, 1H), 1.98–1.81 (m, 2H), 1.28–1.25 (m, 19H), 1.11–1.07 (m, 1H); ^{13}C NMR (125 MHz, $CDCl_3$) δ 168.9, 168.8, 142.5, 131.5, 128.4, 128.3, 128.1, 127.5, 125.8, 124.0, 91.8, 83.4, 82.9, 61.4, 61.3, 55.5, 42.9, 34.9, 31.2, 30.8, 25.0, 24.7, 14.1, 14.0. The carbon bound to the boron was not detected due to quadrupolar relaxation; IR (neat) 3021, 1727, 1370, 1219, 1143, 772, 686, 672 cm^{-1}; HRMS (ESI) calcd for [$C_{32}H_{41}BO_6$+Na^+]: 555.2894, found: 555.2895; 95% ee was measured by chiral HPLC on IA column (*i*-PrOH:hexanes = 5:95, 0.5 mL/min, wavelength = 254 nm, 20 °C); t_R = 10.71 min (major), t_R = 10.12 min (minor); $[\alpha]_D^{20}$ = –41.3 (c = 0.9, $CHCl_3$).

<rac-3m>

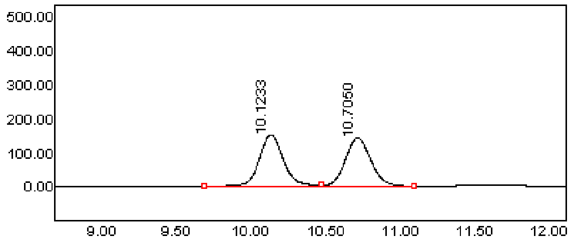

RT[분]	면적[mV*sec]	면적비[%]
10.1233	1644.9211	50.16
10.7050	1634.6776	49.84
	3279.5988	

<chiral-3m>

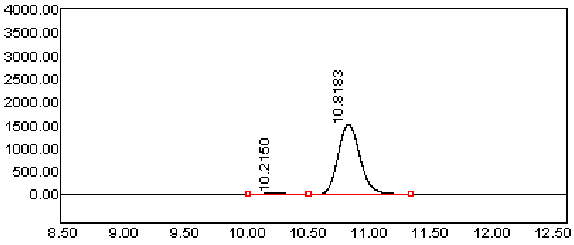

RT[분]	면적[mV*sec]	면적비[%]
10.2150	509.7802	2.61
10.8183	19011.9828	97.39
	19521.7625	

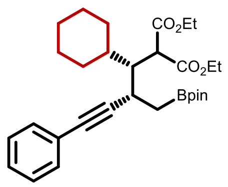

3n

Diethyl 2-(1-cyclohexyl-4-phenyl-2-((4,4,5,5-tetramethyl-1,3,2-dioxaborolan-2-yl)methyl)but-3-yn-1-yl)malonate (3n): By following the general procedure, **3n** was obtained in 55% yield in >98:2 diastereomeric ratio (colorless oil). ^{1}H NMR (500 MHz, $CDCl_3$) δ 7.35–7.33 (m, 2H), 7.26–7.24 (m, 3H), 4.23–4.16 (m, 4H), 3.80 (d, J = 6.5 Hz, 1H), 3.21–3.17 (m, 1H), 2.31–2.27 (m, 1H), 1.94–1.56 (m, 7H), 1.30–1.24 (m, 21H), 1.23–1.07 (m, 3H); ^{13}C NMR (125 MHz, $CDCl_3$) δ 169.7, 169.4, 131.4, 128.1, 127.4, 124.3, 93.1, 83.5, 83.2, 61.3, 61.2, 52.8, 48.6, 38.7, 32.6, 30.6, 29.5, 27.1, 27.0, 26.5, 25.0, 24.6, 16.6 (C-B), 14.02, 13.95; IR (neat) 3032, 1726, 1370, 1219, 1143, 1124, 772, 687, 670 cm^{-1}; HRMS (ESI) calcd for [$C_{30}H_{43}BO_6$+Na^+]: 533.3050, found: 533.3052; 95% ee was measured by chiral HPLC on IA column (i-PrOH:hexanes = 5:95, 0.5 mL/min, wavelength = 254 nm, 20 °C); t_R = 13.10 min (major), t_R = 11.35 min (minor); $[\alpha]_D^{20}$ = –33.9 (c = 0.9, $CHCl_3$).

<rac-3n>

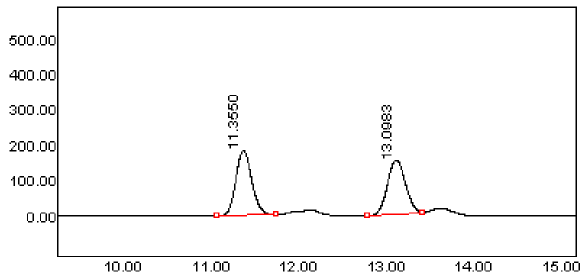

RT[분]	면적[mV*sec]	면적비[%]
11.3550	2134.2742	49.84
13.0983	2147.5654	50.16
	4281.8398	

<chiral-3n>

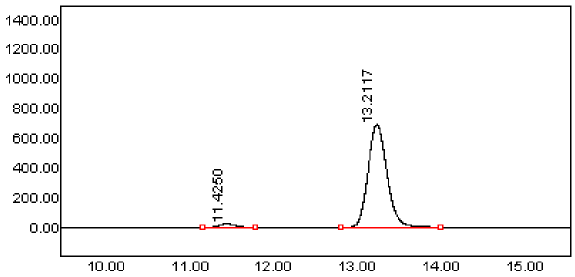

RT[분]	면적[mV*sec]	면적비[%]
11.4250	288.9834	2.59
13.2117	10872.0172	97.41
	11161.0008	

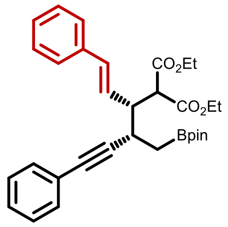

3o

Diethyl 2-(1,6-diphenyl-4-((4,4,5,5-tetramethyl-1,3,2-dioxaborolan-2-yl)methyl)hex-1-en-5-yn-3-yl)malonate (3o): By following the general procedure, **3o** was obtained in 94% yield in >98:2 diastereomeric ratio (colorless oil). ^{1}H NMR (500 MHz, $CDCl_3$) δ 7.44–7.42 (m, 2H), 7.34–7.27 (m, 7H), 7.22–7.19 (m, 1H), 6.50 (d, J = 16.0 Hz, 1H), 6.20 (dd, J = 16.0, 10.0 Hz, 1H), 4.28–4.22 (m, 2H), 4.09–4.02 (m, 2H), 3.86 (d, J = 10.5 Hz, 1H), 3.22–3.19 (m, 1H), 3.09–3.04 (m, 1H), 1.29 (t, J = 7.0 Hz, 3H), 1.26–1.24 (m, 13H), 1.12 (t, J = 7.0 Hz, 3H), 1.05–1.01 (m, 1H); ^{13}C NMR (125 MHz, $CDCl_3$) δ 168.2, 168.1, 137.0, 134.6, 131.7,90.6, 83.6, 83.3, 61.5, 61.3, 55.9, 48.5, 30.3, 25.0, 24.6, 14.09, 14.06. The carbon bound to the boron was not detected due to quadrupolar relaxation; IR (neat) 3032, 1728, 14444, 1370, 1219, 1143, 772, 688, 671 cm^{-1}; HRMS (ESI) calcd for [$C_{32}H_{39}BO_6$+Na^+]: 553.2737, found: 553.2739; 96% ee was measured by chiral HPLC on IA column (i-PrOH:hexanes = 5:95, 0.5 mL/min, wavelength = 254 nm, 20 °C); t_R = 17.16 min (major), t_R = 11.12 min (minor); $[\alpha]_D^{20}$ = –245.3 (c = 1.6, $CHCl_3$).

<rac-3o>

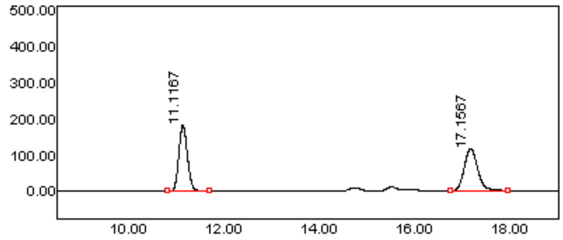

RT[분]	면적[mV*sec]	면적비[%]
11.1167	2306.8148	50.13
17.1567	2295.0127	49.87
	4601.8273	

<chiral-3o>

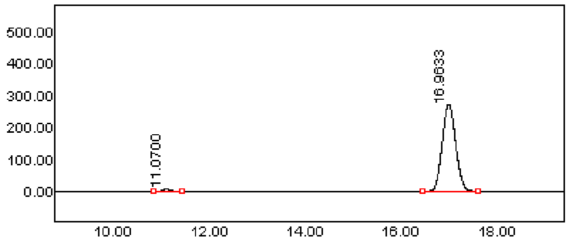

RT[분]	면적[mV*sec]	면적비[%]
11.0700	112.9796	2.06
16.9633	5380.2672	97.94
	5493.2469	

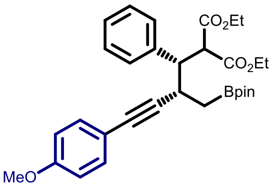

3p

Diethyl 2-(4-(4-methoxyphenyl)-1-phenyl-2-((4,4,5,5-tetramethyl-1,3,2-dioxaborolan-2-yl)methyl)but-3-yn-1-yl)malonate (3p): By following the general procedure, **3p** was obtained in 84% yield in >98:2 diastereomeric ratio (colorless oil). ^{1}H NMR (500 MHz, $CDCl_3$) δ 7.38–7.37 (m, 2H), 7.34–7.33 (m, 2H), 7.25–7.18 (m, 3H), 6.84–6.82 (m, 2H), 4.30–4.27 (m, 2H), 4.25 (d, *J* = 11.8 Hz, 1H), 3.85–3.81 (m, 2H), 3.81 (s, 3H), 3.50 (dd, *J* = 11.7, 4.2 Hz, 1H), 3.36 (m, 1H), 1.31 (t, *J* = 7.0 Hz, 1H), 1.22 (s, 6H), 1.21 (s, 6H), 0.90–0.80 (m, 2H), 0.86 (t, *J* = 7.0 Hz, 3H); ^{13}C NMR (125 MHz, $CDCl_3$) δ 168.3, 168.0, 159.1, 137.4, 132.9, 129.9, 127.7, 127.2, 116.1, 113.8, 89.4, 83.9, 83.2, 61.6, 61.1, 56.5, 55.3, 49.5, 30.6, 25.0, 24.6, 14.1, 13.6. The carbon bound to the boron was not detected due to quadrupolar relaxation; IR (neat) 3033, 1749, 1606, 1219, 773, 685, 672 cm^{-1}; HRMS (ESI) calcd for [$C_{31}H_{39}O_7B$+Na^+]: 557.2687, found: 557.2686; 95% ee was measured by chiral HPLC on IA column (*i*-PrOH:hexanes = 5:95, 0.5 mL/min, wavelength = 254 nm, 20 °C); t_R = 12.84 min (major), t_R = 11.00 min (minor); $[\alpha]_D^{20}$ = –166.2 (c = 1.0, $CHCl_3$).

<rac-3p>

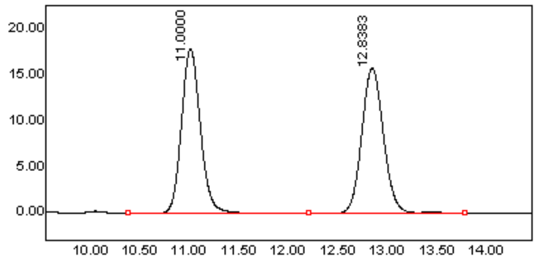

RT[분]	면적[mV*sec]	면적비[%]
11.0000	240.0699	49.67
12.8383	243.2364	50.33
	483.3063	

<chiral-3p>

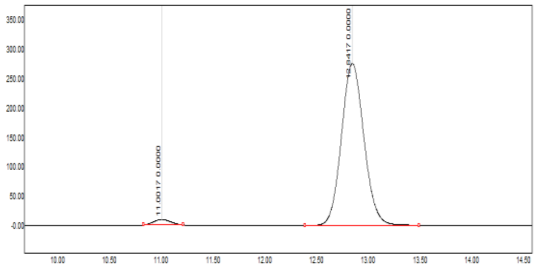

RT[분]	면적[mV*sec]	면적비[%]
11.0017	104.8632	2.46
12.8417	4161.1273	97.54
	4265.9906	

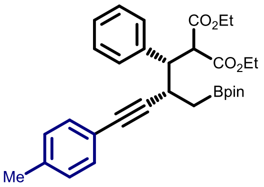

3q

Diethyl 2-(4-(*p*-tolyl)-1-phenyl-2-((4,4,5,5-tetramethyl-1,3,2-dioxaborolan-2-yl)methyl)but-3-yn-1-yl)malonate (3q): By following the general procedure, **3q** was obtained in 79% yield in >98:2 diastereomeric ratio (colorless oil). ^{1}H NMR (500 MHz, $CDCl_3$) δ 7.38–7.37 (m, 2H), 7.31–7.29 (m, 2H), 7.23–7.20 (m, 3H), 7.11–7.10 (m, 2H), 4.32–4.25 (m, 2H), 4.25 (d, J = 11.8 Hz, 1H), 3.88–3.78 (m, 2H), 3.51 (dd, J = 11.7, 4.3 Hz, 1H), 3.39–3.35 (m, 1H), 2.35 (s, 3H), 1.31 (t, J = 7.1 Hz, 3H), 1.22 (s, 6H), 1.21 (s, 6H), 0.90–0.80 (m, 2H), 0.86 (t, J = 7.1 Hz, 3H); ^{13}C NMR (125 MHz, $CDCl_3$) δ 168.3, 167.9, 137.7, 137.4, 131.4, 129.9, 128.9, 127.7, 127.2, 120.8, 90.2, 84.1, 83.3, 61.6, 61.1, 56.5, 49.5, 30.6, 29.7, 25.0, 25.0, 24.6, 21.4, 17.1 (C-B), 14.1, 13.6; IR (neat) 3032, 1729, 1370, 1219, 1143, 775, 685, 672 cm^{-1}; HRMS (ESI) calcd for [$C_{31}H_{39}BO_6$+Na^+]: 541.2737, found: 541.2736; 94% ee was measured by chiral HPLC on IA column (*i*-PrOH:hexanes = 5:95, 0.5 mL/min, wavelength = 254 nm, 20 °C); t_R = 10.36 min (major), t_R = 8.87 min (minor); $[\alpha]_D^{20}$ = –663.7 (c = 2.3, $CHCl_3$).

<rac-3q>

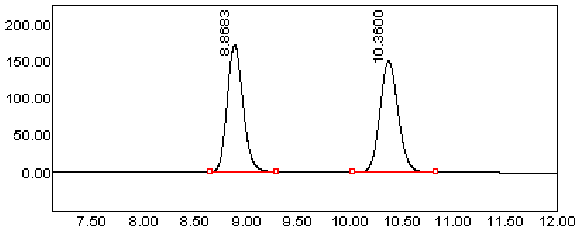

RT[분]	면적[mV*sec]	면적비[%]
8.8683	1840.9580	49.93
10.3600	1845.8561	50.07
	3686.8141	

<chiral-3q>

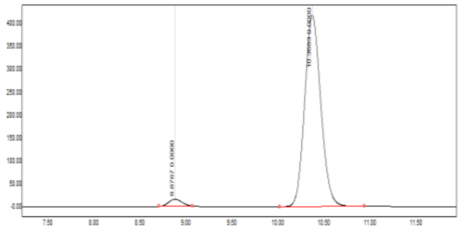

RT[분]	면적[mV*sec]	면적비[%]
8.8767	154.2730	2.94
10.3683	5094.8227	97.06
	5249.0957	

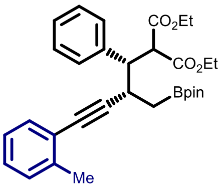

3r

Diethyl 2-(4-(*o*-tolyl)-1-phenyl-2-((4,4,5,5-tetramethyl-1,3,2-dioxaborolan-2-yl)methyl)but-3-yn-1-yl)malonate (3r): By following the general procedure, **3r** was obtained in 66% yield in >98:2 diastereomeric ratio (colorless oil). ^{1}H NMR (500 MHz, $CDCl_3$) δ 7.40–7.39 (m, 3H), 7.24–7.18 (m, 5H), 7.14–7.11 (m, 1H), 4.32–4.25 (m, 2H), 4.27 (d, J = 11.9 Hz, 1H), 3.86–3.80 (m, 2H), 3.53 (dd, J = 11.7, 4.4 Hz, 1H), 3.43 (ddd, J = 10.9, 6.5, 4.5 Hz, 1H), 2.41 (s, 3H), 1.32 (t, J = 7.1 Hz, 3H), 1.21 (s, 6H), 1.20 (s, 6H), 0.87–0.85 (m, 2H), 0.86 (t, J = 7.1 Hz, 3H); ^{13}C NMR (125 MHz, $CDCl_3$) δ 168.3, 167.9, 140.2, 137.4, 132.0, 129.8, 129.3, 127.7, 127.7, 127.3, 125.4, 123.6, 94.9, 83.3, 83.1, 61.6, 61.1, 56.6, 49.4, 30.7, 29.7, 25.0, 24.7, 20.9, 14.1, 13.6. The carbon bound to the boron was not detected due to quadrupolar relaxation; IR (neat) 3033, 1729, 1370, 1219, 1143, 774, 685, 672 cm^{-1}; HRMS (ESI) calcd for [$C_{31}H_{39}BO_6$+Na^+]: 541.2737, found: 541.2736; 64% ee was measured by chiral HPLC on IA column (*i*-PrOH:hexanes = 5:95, 0.5 mL/min, wavelength = 254 nm, 20 °C); t_R = 10.20 min (major), t_R = 8.39

min (minor); $[\alpha]_D^{20}$ = –61.6 (c = 0.9, $CHCl_3$).

<rac-3r>

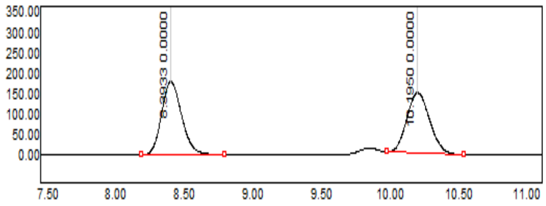

RT[분]	면적[mV*sec]	면적비[%]
8,3933	1832,2898	51,67
10,1950	1713,8629	48,33
	3546,1527	

<chiral-3r>

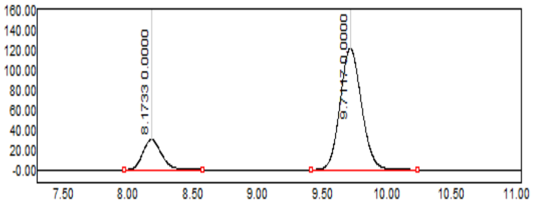

RT[분]	면적[mV*sec]	면적비[%]
8,1733	307,2359	17,96
9,7117	1403,8247	82,04
	1711,0605	

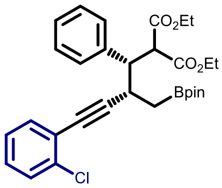

3s

Diethyl 2-(4-(2-chlorophenyl)-1-phenyl-2-((4,4,5,5-tetramethyl-1,3,2-dioxaborolan-2-yl)methyl)but-3-yn-1-yl)malonate (3s): By following the general procedure in condition B, **3s** was obtained in 90% yield in >98:2 diastereomeric ratio (colorless oil). ^{1}H NMR (500 MHz, $CDCl_3$) δ 7.44–7.38 (m, 4H), 7.25–7.17 (m, 5H), 4.36 (d, *J* = 11.6 Hz, 1H), 4.32–4.24 (m, 2H), 3.86–3.80 (m, 2H), 3.56–3.47 (m, 2H), 1.30 (t, *J* = 7.1 Hz, 3H), 1.23 (s, 6H), 1.22 (s, 6H), 0.88–0.85 (m, 2H), 0.86 (t, *J* = 7.1 Hz, 3H); ^{13}C NMR (125 MHz, $CDCl_3$) δ 168.3, 168.0, 137.3, 135.9, 133.4, 129.9, 129.2, 128.7, 127.8, 127.3, 126.3, 123.7, 96.7, 83.3, 81.0, 61.6, 61.1, 56.4, 49.3, 30.7, 25.0, 24.9, 24.7, 14.1, 13.6. The carbon bound to the boron was not detected due to quadrupolar relaxation; IR (neat) 3033, 1729, 1370, 1219, 1143, 768, 685, 672 cm^{-1}; HRMS (EI$^+$) calcd for [$C_{30}H_{36}BClO_6$]: 538.2299, found: 538.2292; 83% ee was measured by chiral HPLC on IA column (*i*-PrOH:hexanes = 5:95, 0.5 mL/min, wavelength = 254 nm, 20 °C); t_R = 10.78 min (major), t_R = 8.76 min (minor); $[\alpha]_D^{20}$ = –117.6 (c = 1.0, $CHCl_3$).

<rac-3s>

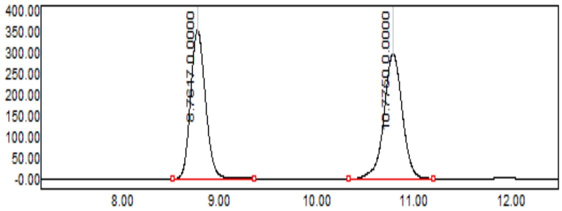

RT[분]	면적[mV*sec]	면적비[%]
8,7617	3729,6293	48,85
10,7750	3905,5770	51,15
	7635,2063	

<chiral-3s>

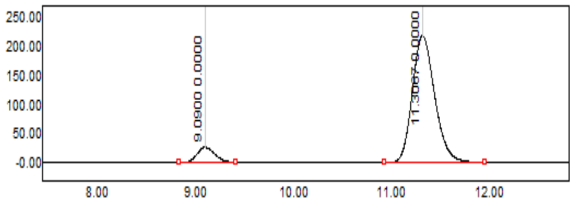

RT[분]	면적[mV*sec]	면적비[%]
9,0900	330,2141	8,72
11,3067	3458,6754	91,28
	3788,8895	

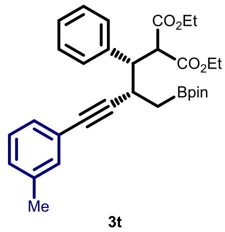

3t

Diethyl 2-(1-phenyl-2-((4,4,5,5-tetramethyl-1,3,2-dioxaborolan-2-yl)methyl)-4-(m-tolyl)but-3-yn-1-yl)malonate (3t): By following the general procedure, **3t** was obtained in 82% yield in >98:2 diastereomeric ratio (colorless oil). ^{1}H NMR (500 MHz, $CDCl_3$) δ 7.39–7.38 (m, 2H), 7.25–7.17 (m, 6H), 7.11–7.09 (m, 1H), 4.32–4.24 (m, 2H), 4.25 (d, J = 11.7 Hz, 1H), 3.88–3.79 (m, 2H), 3.51 (dd, J = 11.7, 4.3 Hz, 1H), 3.38 (ddd, J = 10.3, 6.3, 4.3 Hz, 1H), 2.33 (s, 3H), 1.31 (t, J = 7.1 Hz, 3H), 1.23 (s, 6H), 1.22 (s, 6H), 0.90–0.83 (m, 2H), 0.87 (t, J = 7.1 Hz, 3H); ^{13}C NMR (125 MHz, $CDCl_3$) δ 168.3, 167.9, 137.8, 137.4, 132.1, 129.9, 128.6, 128.5, 128.1, 127.7, 127.2, 123.7, 90.7, 84.3, 83.3, 61.6, 61.1, 56.5, 49.5, 30.6, 29.7, 26.0, 25.0, 24.6, 21.2, 14.1, 17.1 (C-B), 13.6; IR (neat) 3033, 1729, 1370, 1219, 1143, 775, 686, 672 cm^{-1}; HRMS (EI$^+$) calcd for [$C_{31}H_{39}BO_6$]: 518.2845, found: 518.2836; 93% ee was measured by chiral HPLC on IA column (i-PrOH:hexanes = 5:95, 0.5 mL/min, wavelength = 254 nm, 20 °C); t_R = 9.80 min (major), t_R = 8.01 min (minor); $[\alpha]_D^{20}$ = –117.2 (c = 1.0, $CHCl_3$).

<rac-3t>

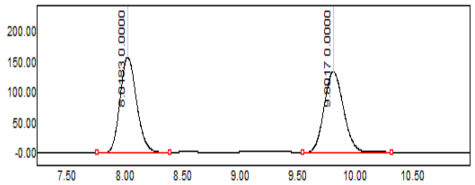

RT[분]	면적[mV*sec]	면적비[%]
8.0183	1574.4881	50.22
9.8017	1560.6830	49.78
	3135.1711	

<chiral-3t>

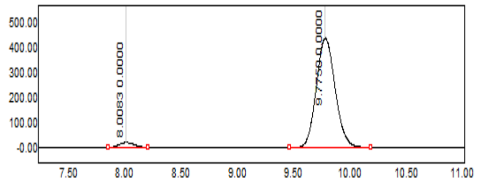

RT[분]	면적[mV*sec]	면적비[%]
8.0083	187.0898	3.55
9.7750	5081.6922	96.45
	5268.7820	

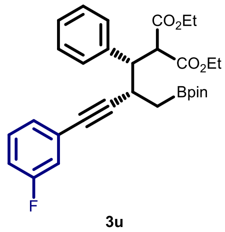

3u

Diethyl 2-(4-(3-fluorophenyl)-1-phenyl-2-((4,4,5,5-tetramethyl-1,3,2-dioxaborolan-2-yl)methyl)but-3-yn-1-yl)malonate (3u): By following the general procedure in condition B, **3u** was obtained in 92% yield in >98:2 diastereomeric ratio (colorless oil). ^{1}H NMR (500 MHz, $CDCl_3$) δ 7.37–7.35 (m, 2H), 7.28–7.20 (m, 4H), 7.18–7.17 (m, 1H), 7.10–7.07 (m, 1H), 7.02–6.98 (m, 1H), 4.32–4.26 (m, 2H), 4.21 (d, J = 11.7 Hz, 1H), 3.89–3.80 (m, 2H), 3.52 (dd, J = 11.7, 4.3 Hz, 1H), 3.37 (ddd, J = 10.2, 6.0, 4.4 Hz, 1H), 1.31 (t, J = 7.1 Hz, 3H), 1.22 (s, 6H), 1.21 (s, 6H), 0.91–0.81 (m, 2H), 0.87 (t, J = 7.1 Hz, 3H); ^{13}C NMR (125 MHz, $CDCl_3$) δ 168.3, 167.8, 162.4 (d, J = 246.1 Hz), 137.2, 129.8, 129.7, 127.8, 127.4 (d, J = 3.1 Hz), 127.3, 125.7 (d, J = 9.6 Hz), 118.3 (d, J = 22.5 Hz), 115.1 (d, J = 21.3 Hz), 92.3, 83.3, 61.7, 61.2, 56.4, 49.4, 30.6, 29.7, 25.0, 24.6, 17.0 (C-B), 14.1, 13.6; IR (neat) 3032, 1729, 1371, 1219, 1143, 767, 684, 672 cm^{-1}; HRMS (ESI) calcd for [$C_{30}H_{36}BFO_6$+Na^+]: 545.2487, found: 545.2483; 90% ee was measured by chiral HPLC on IA column (i-PrOH:hexanes = 5:95, 0.5 mL/min, wavelength = 254 nm, 20 °C); t_R = 10.44 min (major), t_R = 8.44 min (minor); $[\alpha]_D^{20}$ = –110.6 (c = 1.0, $CHCl_3$).

<rac-3u>

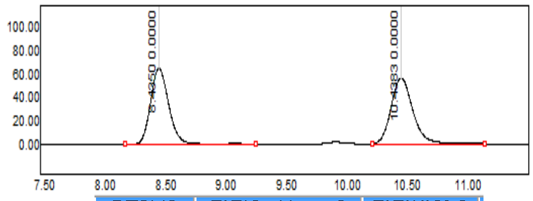

RT[분]	면적[mV*sec]	면적비[%]
8.4350	678.1305	49.42
10.4383	694.0889	50.58
	1372.2193	

<chiral-3u>

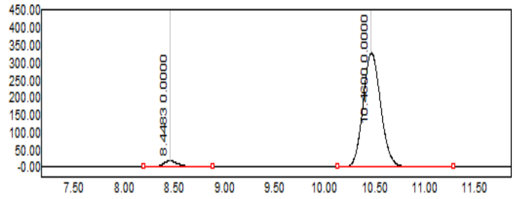

RT[분]	면적[mV*sec]	면적비[%]
8.4483	202.4774	4.73
10.4600	4077.8953	95.27
	4280.3727	

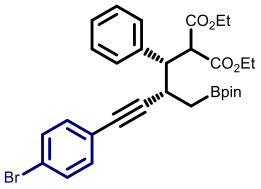

3v

Diethyl 2-(4-(4-bromophenyl)-1-phenyl-2-((4,4,5,5-tetramethyl-1,3,2-dioxaborolan-2-yl)methyl)but-3-yn-1-yl)malonate (3v): By following the general procedure, **3v** was obtained in 80% yield in >98:2 diastereomeric ratio (colorless oil). ^{1}H NMR (500 MHz, $CDCl_3$) δ 7.44–7.42 (m, 2H), 7.36–7.34 (m, 2H), 7.24–7.19 (m, 4H), 4.28 (q, J = 7.0 Hz, 2H), 4.21 (d, J = 11.7 Hz, 1H), 3.89–3.80 (m, 2H), 3.51 (dd, J = 11.7, 4.2 Hz, 1H), 3.38–3.34 (m, 1H), 1.31 (t, J = 7.1 Hz, 3H), 1.22 (s, 6H), 1.21 (s, 6H), 0.90–0.83 (m, 2H), 0.87 (t, J = 7.1 Hz, 3H); ^{13}C NMR (125 MHz, $CDCl_3$) δ 168.2, 167.8, 137.2, 133.0, 131.5, 129.8, 127.7, 127.3, 122.8, 121.8, 92.4, 83.5, 83.3, 83.1, 61.7, 61.2, 56.4, 49.4, 30.7, 29.7, 26.0, 25.0, 24.6, 14.1, 13.6. The carbon bound to the boron was not detected due to quadrupolar relaxation; IR (neat) 3033, 1728, 1370, 1219, 1143, 775, 685, 671 cm^{-1}; HRMS (ESI) calcd for [$C_{30}H_{36}BBrO_6$+Na^+]: 605.1686, found: 605.1685; 91% ee was measured by chiral HPLC on IA column (*i*-PrOH:hexanes = 5:95, 0.5 mL/min, wavelength = 254 nm, 20 °C); t_R = 10.96 min (major), t_R = 9.13 min (minor); $[\alpha]_D^{20}$ = –131.9 (c = 1.1, $CHCl_3$).

<rac-3v>

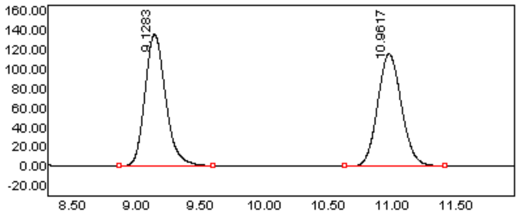

RT[분]	면적[mV*sec]	면적비[%]
9.1283	1518.2205	50.43
10.9617	1492.4976	49.57
	3010.7180	

<chiral-3v>

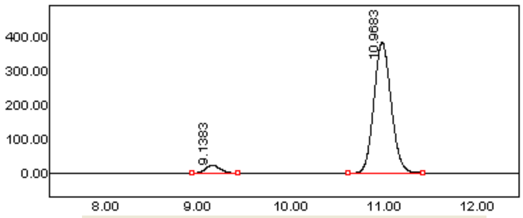

RT[분]	면적[mV*sec]	면적비[%]
9.1383	246.1256	4.72
10.9683	4969.8703	95.28
	5215.9957	

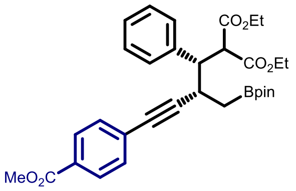

3w

Diethyl 2-(4-(4-(methoxycarbonyl)phenyl)-1-phenyl-2-((4,4,5,5-tetramethyl-1,3,2-dioxaborolan-2-yl)methyl)but-3-yn-1-yl)malonate (3w): By following the general procedure in condition B, **3w** was obtained in 80% yield in >98:2 diastereomeric ratio (colorless oil). ^{1}H NMR (500 MHz, $CDCl_3$) δ 7.98–7.97 (m, 2H), 7.46–7.44 (m, 2H), 7.37–7.36 (m, 2H), 7.25–7.20 (m, 3H), 4.31–4.26 (m, 2H), 4.22 (d, J = 11.7 Hz, 2H), 3.92 (s, 3H), 3.88–3.81 (m, 2H), 3.53 (dd, J = 11.7, 4.2 Hz, 1H), 3.40 (ddd, J = 10.1, 6.0, 4.4 Hz, 1H), 1.31 (t, J = 7.1 Hz, 3H), 1.22 (s, 6H), 1.21 (s, 6H), 0.93–0.83 (m, 2H), 0.87 (t, J = 7.1 Hz, 3H); ^{13}C NMR (125 MHz, $CDCl_3$) δ 168.2, 167.8, 166.6, 137.2, 131.4, 129.8, 129.4, 129.1, 128.6, 127.8, 127.4, 94.6, 83.4, 61.7, 61.2, 56.4, 52.2, 49.4, 30.8, 25.0, 24.6, 14.1, 13.6. The carbon bound to the boron was not detected due to quadrupolar relaxation; IR (neat) 3032, 1724, 1441, 1370, 1219, 1143, 771, 685, 672 cm^{-1}; HRMS (ESI) calcd for [$C_{32}H_{39}BO_8$+Na^+]: 585.2636, found: 585.2637; 35% ee was measured by chiral HPLC on IA column (*i*-PrOH:hexanes = 5:95, 0.5 mL/min, wavelength = 254 nm, 20 °C); t_R = 15.30 min (major), t_R = 13.54 min (minor); $[\alpha]_D^{20}$ = –358.1 (c = 1.8, $CHCl_3$).

<rac-3w>

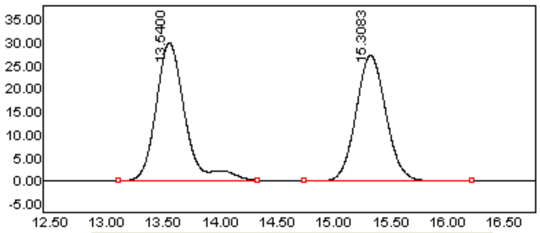

RT[분]	면적[mV*sec]	면적비[%]
13.5400	530.5644	51.54
15.3083	498.9414	48.46
	1029.5058	

<chiral-3w>

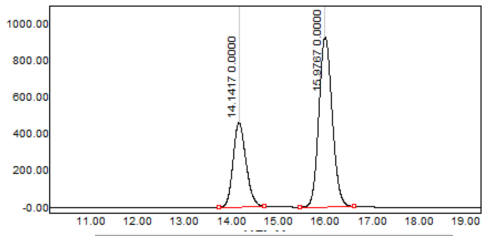

RT[분]	면적[mV*sec]	면적비[%]
14.1417	8718.1812	32.33
15.9767	18251.3000	67.67
	26969.4813	

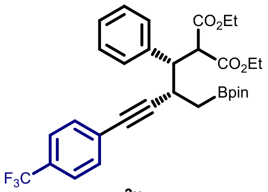

3x

Diethyl 2-(1-phenyl-2-((4,4,5,5-tetramethyl-1,3,2-dioxaborolan-2-yl)methyl)-4-(4-(trifluoromethyl)phenyl)but-3-yn-1-yl)malonate (3x): By following the general procedure in condition B, **3x** was obtained in 90% yield in >98:2 diastereomeric ratio (colorless oil). ^{1}H NMR (500 MHz, $CDCl_3$) δ 7.57–7.55 (m, 2H), 7.50–7.48 (m, 2H), 7.36–7.35 (m, 2H), 7.27–7.20 (m, 3H), 4.31–4.27 (m, 2H), 4.21 (d, J = 11.7 Hz, 1H), 3.90–3.80 (m, 2H), 3.53 (dd, J = 11.7, 4.2 Hz, 1H), 3.42–3.37 (m, 1H), 1.31 (t, J = 7.1 Hz, 3H), 1.23 (s, 6H), 1.22 (s, 6H), 0.93–0.85 (m, 2H), 0.87 (t, J = 7.1 Hz, 3H); ^{13}C NMR (125 MHz, $CDCl_3$) δ 168.2, 167.8, 137.1, 131.7, 129.7(q, J = 32.3 Hz), 128.8, 128.4, 127.8, 127.4, 125.2 (q, J = 3.7 Hz), 124.0 (q, J = 272.1 Hz), 94.0, 83.5, 83.4, 61.7, 61.2, 56.4, 49.3, 30.7, 25.1, 25.0, 24.6, 14.1, 13.6. The carbon bound to the boron was not detected due to quadrupolar relaxation; IR (neat) 3033, 1730, 1324, 1219, 1068, 770, 685, 673 cm^{-1}; HRMS (ESI) calcd for [$C_{31}H_{36}BF_3O_6$+Na^+]: 595.2455, found: 595.2455; 86% ee was measured by chiral HPLC on IA column (*i*-PrOH:hexanes = 5:95, 0.5

mL/min, wavelength = 254 nm, 20 °C); t_R = 10.04 min (major), t_R = 8.25 min (minor); $[\alpha]_D^{20}$ = –73.5 (c = 1.0, $CHCl_3$).

<rac-3x>

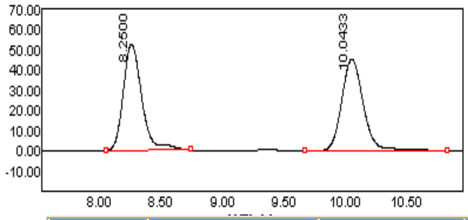

RT[분]	면적[mV*sec]	면적비[%]
8.2500	554.3072	49.34
10.0433	569.2411	50.66
	1123.5482	

<chiral-3x>

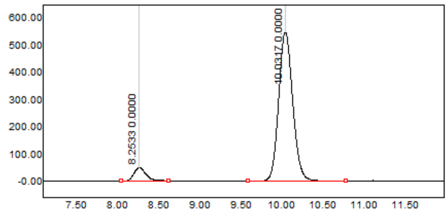

RT[분]	면적[mV*sec]	면적비[%]
8.2533	507.3543	7.25
10.0317	6489.9027	92.75
	6997.2570	

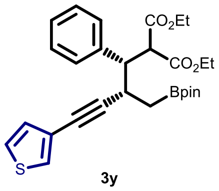

3y

Diethyl 2-(1-phenyl-2-((4,4,5,5-tetramethyl-1,3,2-dioxaborolan-2-yl)methyl)-4-(thiophen-3-yl)but-3-yn-1-yl)malonate (3y): By following the general procedure, **3y** was obtained in 74% yield in >98:2 diastereomeric ratio (white solid). ^{1}H NMR (500 MHz, $CDCl_3$) δ 7.38–7.35 (m, 3H), 7.26–7.19 (m, 4H), 7.08–7.07 (m, 1H), 4.28 (q, J = 7.1 Hz, 2H), 4.23 (d, J = 11.7 Hz, 1H), 3.88–3.78 (m, 2H), 3.50 (dd, J = 11.7, 4.2 Hz, 1H), 3.36 (ddd, J = 10.2, 6.1, 4.3 Hz, 1H), 1.31 (t, J = 7.1 Hz, 3H), 1.22 (s, 6H), 1.21 (s, 6H), 0.90–0.83 (m, 2H), 0.86 (t, J = 7.1 Hz, 3H); ^{13}C NMR (125 MHz, $CDCl_3$) δ 168.3, 167.9, 137.3, 129.9, 129.8, 127.7, 127.3, 124.9, 122.9, 90.5, 83.3, 79.3, 61.6, 61.1, 56.4, 49.4, 30.7, 29.7, 25.0, 24.6, 14.1, 13.6. The carbon bound to the boron was not detected due to quadrupolar relaxation; IR (neat) 3033, 1729, 1444, 1370, 1219, 1143, 773, 685, 673 cm^{-1}; HRMS (ESI) calcd for [$C_{28}H_{35}BO_6S+Na^+$]: 533.2145, found: 533.2145; 94% ee was measured by chiral HPLC on IA column (*i*-PrOH:hexanes = 5:95, 0.5 mL/min, wavelength = 254 nm, 20 °C); t_R = 11.36 min (major), t_R = 9.69 min (minor); $[\alpha]_D^{20}$ = –137.6 (c = 1.1, $CHCl_3$).

<rac-3y>

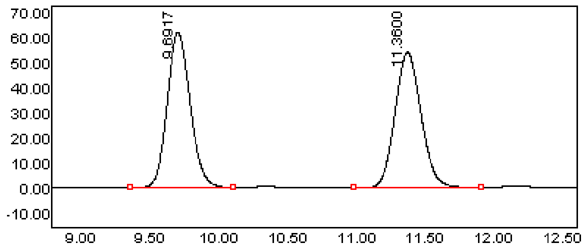

RT[분]	면적[mV*sec]	면적비[%]
9.6917	723.2727	50.15
11.3600	718.8569	49.85
	1442.1296	

<chiral-3y>

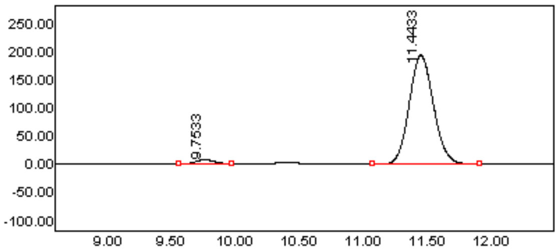

RT[분]	면적[mV*sec]	면적비[%]
9.7533	87.3943	3.29
11.4433	2565.1191	96.71
	2652.5135	

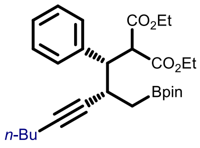

3z

Diethyl 2-(1-phenyl-2-((4,4,5,5-tetramethyl-1,3,2-dioxaborolan-2-yl)methyl)oct-3-yn-1-yl)malonate (3z): By following the general procedure, **3z** was obtained in 86% yield in >98:2 diastereomeric ratio (colorless oil). ^{1}H NMR (500 MHz, $CDCl_3$) δ 7.33–7.32 (m, 2H), 7.22–7.16 (m, 3H), 4.26 (q, *J* = 7.0 Hz, 2H), 4.18 (d, *J* = 12.0 Hz, 1H), 3.85–3.78 (m, 2H), 3.39 (dd, *J* = 12.0, 4.0 Hz, 1H), 3.14–3.10 (m, 1H), 2.18 (td, *J* = 7.0, 2.0 Hz, 2H), 1.52–1.38 (m, 4H), 1.30 (t, *J* = 7.0 Hz, 3H), 1.22 (s, 6H), 1.21 (s, 6H), 0.91 (t, *J* = 7.0 Hz, 3H), 0.86 (t, *J* = 7.0 Hz, 3H), 0.77–0.67 (m, 2H); ^{13}C NMR (125 MHz, $CDCl_3$) δ 168.3, 168.0, 137.6, 129.9, 127.5, 127.0, 84.0, 83.1, 80.9, 61.5, 61.0, 56.6, 49.3, 31.0, 30.0, 24.9, 24.6, 21.9, 18.6, 14.0, 13.59, 13.57. The carbon bound to the boron was not detected due to quadrupolar relaxation; IR (neat) 3033, 1728, 1444, 1370, 1219, 1143, 775, 685, 671 cm^{-1}; HRMS (ESI) calcd for [$C_{28}H_{41}BO_6$+Na^+]: 507.2894, found: 507.2896; 80% ee was measured by chiral HPLC on IA column (*i*-PrOH:hexanes = 5:95, 0.5 mL/min, wavelength = 254 nm, 20 °C); t_R = 12.73 min (major), t_R = 9.88 min (minor); $[\alpha]_D^{20}$ = –80.7 (c = 0.9, $CHCl_3$).

<rac-3z>

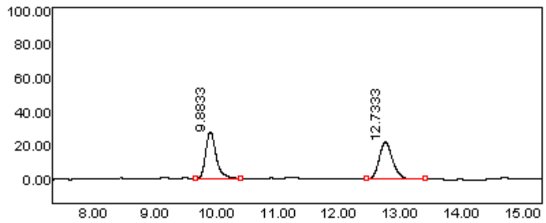

RT[분]	면적[mV*sec]	면적비[%]
9.8833	335.0193	50.59
12.7333	327.1976	49.41
	662.2169	

<chiral-3z>

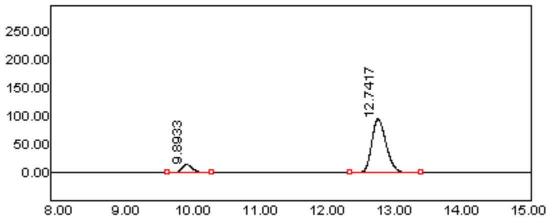

RT[분]	면적[mV*sec]	면적비[%]
9.8933	158.2510	10.03
12.7417	1419.7853	89.97
	1578.0362	

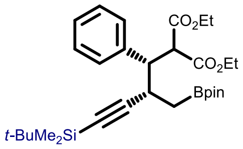

3aa

Diethyl 2-(4-(tert-butyldimethylsilyl)-1-phenyl-2-((4,4,5,5-tetramethyl-1,3,2-dioxaborolan-2-yl)methyl)but-3-yn-1-yl)malonate (3aa): By following the general procedure, **3aa** was obtained in 83% yield in >98:2 diastereomeric ratio (colorless oil). ^{1}H NMR (500 MHz, $CDCl_3$) δ 7.36–7.34 (m, 2H), 7.22–7.18 (m, 3H), 4.31–4.22 (m, 2H), 4.19 (d, *J* = 11.5 Hz, 1H), 3.86–3.79 (m, 2H), 3.41 (dd, *J* = 11.5, 4.5 Hz, 1H), 3.22–3.18 (m, 1H), 1.30 (t, *J* = 7.0 Hz, 3H), 1.223 (s, 6H), 1.215 (s, 6H), 0.95 (s, 9H), 0.86 (t, *J* = 7.0 Hz, 3H), 0.78–0.69 (m, 2H), 0.112 (s, 3H), 0.105 (s, 3H); ^{13}C NMR (125 MHz, $CDCl_3$) δ 168.3, 167.9, 137.3, 129.8, 127.6, 127.2, 108.7, 86.3, 83.2, 61.5, 61.0, 56.5, 49.0, 30.8, 26.2, 25.0, 24.6, 16.6, 14.0, 13.6, -4.53. The carbon bound to the boron was not detected due to quadrupolar relaxation; IR (neat) 3032, 1729, 1444, 1370, 1219, 1143, 772, 685, 673 cm^{-1}; HRMS (ESI) calcd for [$C_{30}H_{47}BSiO_6$+Na^+]: 565.3133, found: 565.3134; 86% ee was measured by chiral HPLC on IA column (*i*-PrOH:hexanes = 5:95, 0.5 mL/min, wavelength = 254 nm, 20 °C); t_R = 11.42 min (major), t_R = 8.65 min (minor); $[\alpha]_D^{20}$ =

–77.5 (c = 0.9, $CHCl_3$).

<rac-3aa>

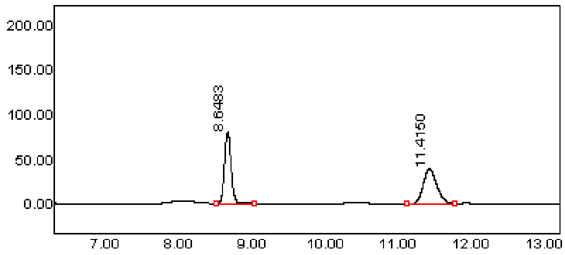

RT[분]	면적[mV*sec]	면적비[%]
8.6483	486.1489	49.82
11.4150	489.6231	50.18
	975.7721	

<chiral-3aa>

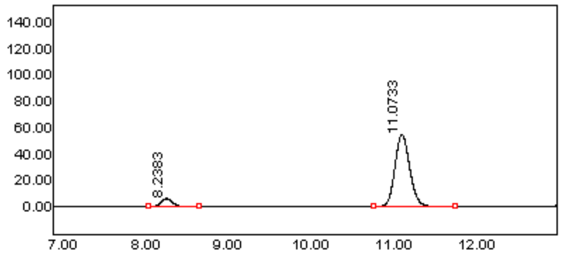

RT[분]	면적[mV*sec]	면적비[%]
8.2383	51.7449	7.13
11.0733	674.3291	92.87
	726.0739	

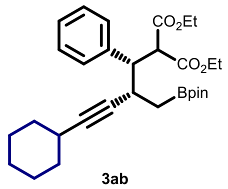

3ab

Diethyl 2-(4-cyclohexyl-1-phenyl-2-((4,4,5,5-tetramethyl-1,3,2-dioxaborolan-2-yl)methyl)but-3-yn-1-yl)malonate (3ab): By following the general procedure, **3ab** was obtained in 84% yield in >98:2 diastereomeric ratio (colorless oil). ^1H NMR (500 MHz, $CDCl_3$) δ 7.33–7.32 (m, 2H), 7.22–7.15 (m, 3H), 4.28–4.23 (m, 2H), 4.17 (d, *J* = 12.0 Hz, 1H), 3.85–3.78 (m, 2H), 3.39 (dd, *J* = 12.0, 4.5 Hz, 1H), 3.14–3.10 (m, 1H), 2.44–2.33 (m, 1H), 1.79–1.64 (m, 4H), 1.51–1.41 (m, 3H), 1.33–1.28 (m, 6H), 1.22 (s, 6H), 1.21 (s, 6H), 0.86 (t, *J* = 7.0 Hz, 3H), 0.76–0.65 (m, 2H); ^{13}C NMR (125 MHz, $CDCl_3$) δ 168.3, 168.1, 137.6, 129.9, 127.5, 127.0, 88.1, 83.1, 80.8, 61.5, 61.0, 56.5, 49.4, 32.9, 32.8, 29.9, 29.2, 26.0, 25.0, 24.9, 24.8, 24.6, 17.5 (C–B), 14.1, 13.6; IR (neat) 3032, 1728, 1446, 1370, 1219, 1143, 772, 700, 671 cm^{-1}; HRMS (ESI) calcd for $[C_{30}H_{43}BO_6+Na^+]$: 533.3050, found: 533.3052; 84% ee was measured by chiral HPLC on IA column (*i*-PrOH:hexanes = 5:95, 0.5 mL/min, wavelength = 254 nm, 20 °C); t_R = 12.90 min (major), t_R = 9.47 min (minor); $[\alpha]_D^{20}$ = –92.9 (c = 0.9, $CHCl_3$).

<rac-3ab>

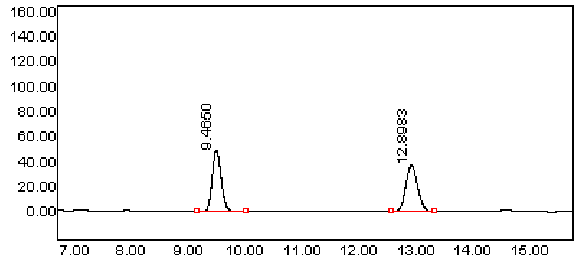

RT[분]	면적[mV*sec]	면적비[%]
9.4650	535.8252	50.06
12.8983	534.5264	49.94
	1070.3516	

<chiral-3ab>

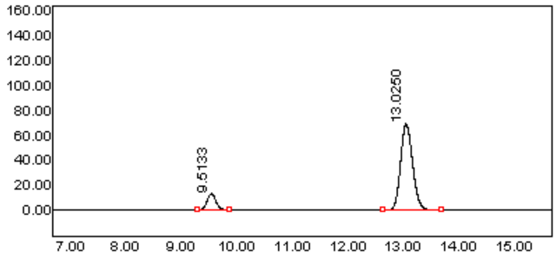

RT[분]	면적[mV*sec]	면적비[%]
9.5133	131.7919	11.28
13.0250	1037.0105	88.72
	1168.8024	

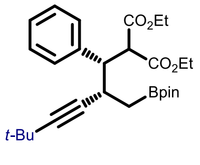

3ac

Diethyl 2-(5,5-dimethyl-1-phenyl-2-((4,4,5,5-tetramethyl-1,3,2-dioxaborolan-2-yl)methyl)hex-3-yn-1-yl)malonate (3ac): By following the general procedure, **3ac** was obtained in 85% yield in >98:2 diastereomeric ratio (colorless oil). ^{1}H NMR (500 MHz, $CDCl_3$) δ 7.33–7.31 (m, 2H), 7.22–7.15 (m, 3H), 4.29–4.22 (m, 2H), 4.14 (d, J = 11.5 Hz, 1H), 3.86–3.79 (m, 2H), 3.38 (dd, J = 11.5, 4.0 Hz, 1H), 3.10–3.06 (m, 1H), 1.30 (t, J = 7.0 Hz, 3H), 1.22 (s, 15H), 1.21 (s, 6H), 0.85 (t, J = 7.0 Hz, 3H), 0.75–0.62 (m, 2H); ^{13}C NMR (125 MHz, $CDCl_3$) δ 168.4, 168.1, 137.6, 129.9, 127.4, 127.0, 92.2, 83.1, 79.3, 61.5, 61.0, 56.5, 49.5, 31.2, 29.7, 27.5, 25.0, 24.6, 17.5(C–B), 14.1, 13.6; IR (neat) 3032, 1729, 1445, 1370, 1219, 1143, 769, 685, 671 cm^{-1}; HRMS (ESI) calcd for [$C_{28}H_{41}BO_6$+Na^+]: 507.2894, found: 507.2894; 81% ee was measured by chiral HPLC on IA column (i-PrOH:hexanes = 5:95, 0.5 mL/min, wavelength = 254 nm, 20 °C); t_R = 11.53 min (major), t_R = 8.94 min (minor); $[\alpha]_D^{20}$ = –57.5 (c = 0.9, $CHCl_3$).

<rac-3ac>

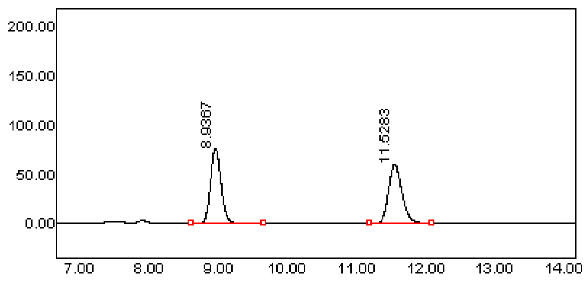

RT[분]	면적[mV*sec]	면적비[%]
8.9367	805.1453	49.63
11.5283	817.1326	50.37
	1622.2779	

<chiral-3ac>

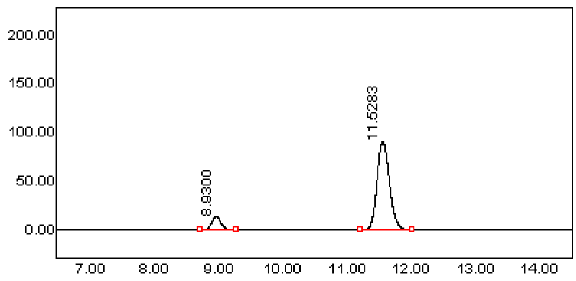

RT[분]	면적[mV*sec]	면적비[%]
8.9300	124.6965	9.41
11.5283	1200.6465	90.59
	1325.3430	

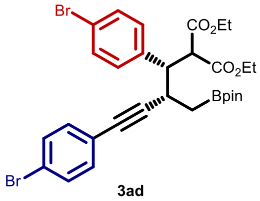

3ad

Diethyl 2-(1,4-bis(4-bromophenyl)-2-((4,4,5,5-tetramethyl-1,3,2-dioxaborolan-2-yl)methyl)but-3-yn-1-yl)malonate (3ad): By following the general procedure, **3ad** was obtained in 76% yield in >98:2 diastereomeric ratio (colorless oil). ^{1}H NMR (500 MHz, $CDCl_3$) δ 7.45–7.42 (m, 2H), 7.40–7.38 (m, 2H), 7.25–7.23 (m, 4H), 4.28 (q, J = 7.1 Hz, 2H), 4.15 (d, J = 11.7 Hz, 1H), 3.91–3.85 (m, 2H), 3.49 (dd, J = 11.7, 4.2 Hz, 1H), 3.34 (ddd, J = 10.1, 6.1, 4.2 Hz, 1H), 1.31 (t, J = 7.1 Hz, 3H), 1.22 (s, 6H), 1.21 (s, 6H), 0.94 (t, J = 7.1 Hz, 3H), 0.88–0.78 (m, 2H); ^{13}C NMR (125 MHz, $CDCl_3$) δ 168.0, 167.6, 136.4, 133.0, 131.5, 131.4, 130.9, 122.5, 122.1, 121.5, 91.9, 83.4, 83.2, 61.8, 61.4, 56.2, 48.8, 30.6, 25.0, 24.6, 14.1, 13.7. The carbon bound to the boron was not detected due to quadrupolar relaxation; IR (neat) 3032, 1728, 1446, 1370, 1219, 1143, 773, 685, 671 cm^{-1}; HRMS (ESI) calcd for [$C_{30}H_{35}BBr_2O_6$+Na^+]: 683.0791, found: 683.0790; 92% ee was measured by chiral HPLC on IA column (i-PrOH:hexanes = 5:95, 0.5 mL/min, wavelength = 254 nm, 20 °C); t_R = 14.03 min (major), t_R = 9.40 min (minor); $[\alpha]_D^{20}$ = –121.5 (c = 1.1, $CHCl_3$).

<rac-3ad>

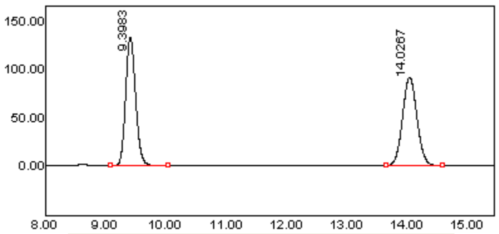

RT[분]	면적[mV*sec]	면적비[%]
9.3983	1531.3868	50.13
14.0267	1523.3696	49.87
	3054.7564	

<chiral-3ad>

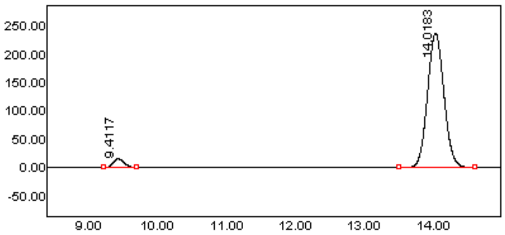

RT[분]	면적[mV*sec]	면적비[%]
9.4117	173.2244	4.21
14.0183	3937.8031	95.79
	4111.0277	

General Procedure for the Cu-Catalysed Coupling of Internal Enyne (4) with 2.

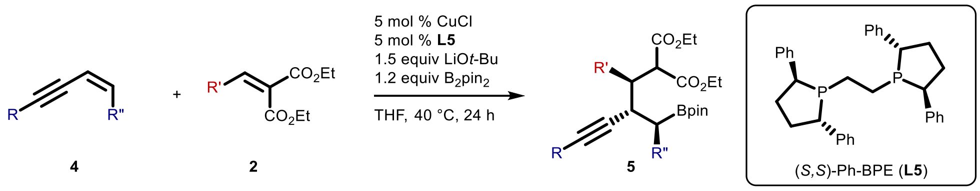

A mixture of CuCl (5 mol %, 0.025 mmol), **L5** (5 mol %, 0.025 mmol), LiO*t*-Bu (1.5 equiv, 0.75 mmol), and B_2pin_2 (1.2 equiv, 0.6 mmol) in THF (0.7 mL) was stirred for 15 min in a Schlenk tube under an atmosphere of nitrogen. Substrate **4** (1.2 equiv, 0.6 mmol) and **2** (1 equiv, 0.5 mmol) dissolved in THF (0.3 mL) were added to the reaction mixture. The reaction mixture was stirred at 40 °C and monitored by TLC. Upon complete consumption of **2**, the reaction mixture was diluted with water (3 mL) and extracted with dichloromethane (5 mL x 3). The combined organic layers were washed with brine, dried over $MgSO_4$, and concentrated in vacuo. The residue was purified by column on silica gel using ethyl acetate/hexane as eluent.

Characterization of compound 5.

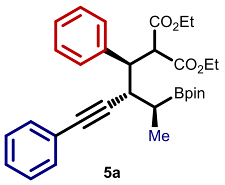

5a

Diethyl 2-(1,4-diphenyl-2-(1-(4,4,5,5-tetramethyl-1,3,2-dioxaborolan-2-yl)ethyl)but-3-yn-1-yl)malonate (5a): By following the general procedure, **5a** was obtained in 92% yield in >98:2 diastereomeric ratio (colorless oil). ^1H NMR (500 MHz, $CDCl_3$) δ 7.46–7.43 (m, 4H), 7.33–7.29 (m, 3H), 7.27–7.23 (m, 2H), 7.22–7.19 (m, 1H), 4.33–4.25 (m, 2H), 4.29 (d, J = 11.7 Hz, 1H), 3.84–3.83 (m, 2H), 3.67 (dd, J = 11.7, 4.1 Hz, 1H), 3.19 (dd, J = 11.1, 4.1 Hz, 1H), 1.34 (s, 6H), 1.33 (s, 6H), 1.33–1.31 (m, 3H), 1.04 (d, J = 7.5 Hz, 3H), 0.89 (t, J = 7.1 Hz, 3H), 0.87–0.82 (m, 1H); ^{13}C NMR (125 MHz, $CDCl_3$) δ 168.1, 167.9, 137.9, 131.6, 129.8, 128.2, 127.7, 127.7, 127.2, 123.9, 89.8, 85.8, 83.4, 61.5, 61.0, 57.1, 47.6, 38.1, 24.9,

24.8, 24.6, 20.5 (C-B), 14.5, 14.2, 13.6; IR (neat) 3033, 1728, 1491, 1445, 1219, 1142, 771, 686, 671 cm^{-1}; HRMS (ESI) calcd for [$C_{31}H_{39}BO_6$+Na^+]: 541.2737, found: 541.2735; >99% ee was measured by chiral HPLC on ADH column (*i*-PrOH:hexanes = 1:99, 0.5 mL/min, wavelength = 254 nm, 20 °C); t_R = 8.51 min (major), t_R = 10.93 min (minor); $[\alpha]_D^{20}$ = –91.6 (c = 0.9, $CHCl_3$).

<rac-5a>

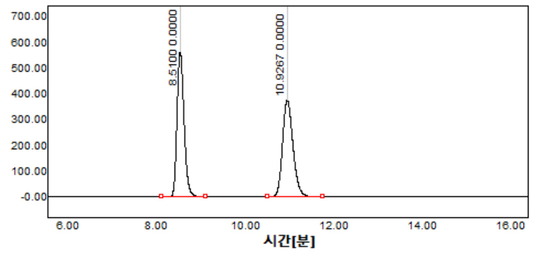

RT[분]	면적[mV*sec]	면적비[%]
8.5100	6207.4344	50.16
10.9267	6167.4234	49.84
	12374.8578	

<chiral-5a>

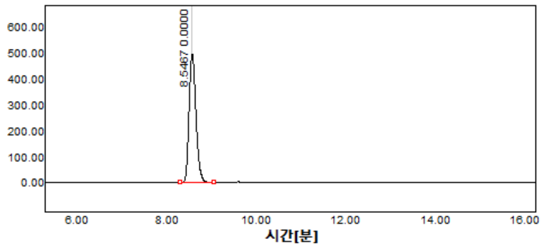

RT[분]	면적[mV*sec]	면적비[%]
8.5467	5355.1895	100.00
	5355.1895	

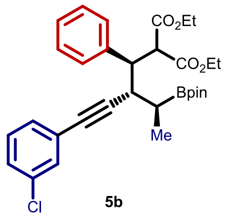

5b

Diethyl 2-(4-(3-chlorophenyl)-1-phenyl-2-(1-(4,4,5,5-tetramethyl-1,3,2-dioxaborolan-2-yl)ethyl)but-3-yn-1-yl)malonate (5b): By following the general procedure, **5b** was obtained in 88% yield in >98:2 diastereomeric ratio (colorless oil). ^{1}H NMR (500 MHz, $CDCl_3$) δ 7.50–7.46 (m, 3H), 7.41–7.39 (m, 1H), 7.25–7.18 (m, 5H), 4.38 (d, *J* = 11.7 Hz, 1H), 4.33–4.24 (m, 2H), 3.84–3.82 (m, 2H), 3.69 (dd, *J* = 11.7, 4.3 Hz, 1H), 3.31 (dd, *J* = 11.0, 4.2 Hz, 1H), 1.34 (s, 6H), 1.33 (s, 6H), 1.33–1.30 (m, 3H), 1.07 (d, *J* = 7.5 Hz, 3H), 0.91–0.86 (m, 1H), 0.88 (t, J = 7.1 Hz, 3H); ^{13}C NMR (125 MHz, $CDCl_3$) δ 168.1, 168.0, 137.9, 135.9, 133.4, 129.9, 129.2, 128.6, 127.8, 127.8, 126.3, 123.8, 95.5, 83.4, 82.8, 61.5, 61.0, 57.0, 47.5, 38.3, 24.9, 24.8, 14.6, 14.1, 13.6. The carbon bound to the boron was not detected due to quadrupolar relaxation; IR (neat) 3033, 1729, 1474, 1369, 1219, 1142, 770, 685, 672 cm^{-1}; HRMS (ESI) calcd for [$C_{31}H_{38}BClO_6$+Na^+]: 575.2348, found: 575.2349; >99% ee was measured by chiral HPLC on IA column (*i*-PrOH:hexanes = 1:99, 0.5 mL/min, wavelength = 254 nm, 20 °C); t_R = 9.02 min (major), t_R = 9.86 min (minor); $[\alpha]_D^{20}$ = –106.6 (c = 1.0, $CHCl_3$).

<rac-5b>

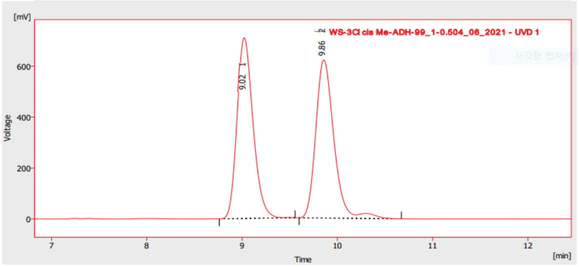

<chiral-5b>

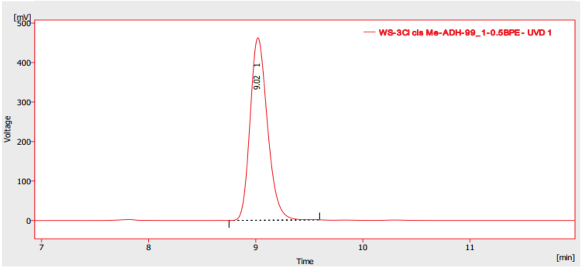

	Reten. Time [min]	Area [mV.s]	Area [%]
1	9.023	8292.664	50.2
2	9.858	8224.125	49.8
	Total	16516.789	100.0

	Reten. Time [min]	Area [mV.s]	Area [%]
1	9.020	5200.831	100.0
	Total	5200.831	100.0

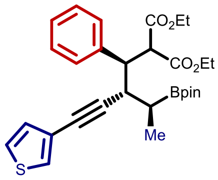

5c

Diethyl 2-(1-phenyl-2-(1-(4,4,5,5-tetramethyl-1,3,2-dioxaborolan-2-yl)ethyl)-4-(thiophen-3-yl)but-3-yn-1-yl)malonate (5c): By following the general procedure, **5c** was obtained in 88% yield in >98:2 diastereomeric ratio (colorless oil). ^{1}H NMR (500 MHz, $CDCl_3$) δ 7.44–7.43 (m, 2H), 7.39–7.38 (m, 1H), 7.26–7.23 (m, 3H), 7.22–7.19 (m, 1H), 7.11 (dd, J = 5.0, 1.0 Hz, 1H), 4.33–4.23 (m, 2H), 4.27 (d, J = 11.8 Hz, 1H), 3.83 (m, 2H), 3.66 (dd, J = 11.7, 4.0 Hz, 1H), 3.16 (dd, J = 11.1, 4.0 Hz, 1H), 1.34 (s, 6H), 1.33 (s, 6H), 1.33–1.31 (m, 3H), 1.03 (d, J = 7.4 Hz, 3H), 0.89 (t, J = 7.1 Hz, 3H), 0.86–0.81 (m, 1H); ^{13}C NMR (125 MHz, $CDCl_3$) δ 168.0, 167.9, 137.9, 130.0, 129.8, 127.7, 127.6, 127.2, 125.0, 122.9, 89.3, 83.4, 80.8, 61.5, 61.0, 57.0, 47.6, 38.1, 24.8, 24.7, 14.5, 14.2, 13.6. The carbon bound to the boron was not detected due to quadrupolar relaxation; IR (neat) 3033, 1729, 1544, 1445, 1379, 1219, 1142, 769, 685, 672 cm^{-1}; HRMS (EI$^+$) calcd for [$C_{29}H_{37}BSO_6$]: 524.2409, found: 524.2404; >99% ee was measured by chiral HPLC on ADH column (i-PrOH:hexanes = 1:99, 0.3 mL/min, wavelength = 254 nm, 20 °C); t_R = 9.71 min (major), t_R = 14.00 min (minor); $[\alpha]_D^{20}$ = –73.6 (c = 0.9, $CHCl_3$).

<rac-5c>

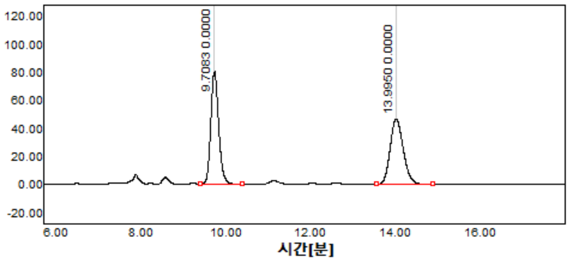

RT[분]	면적[mV*sec]	면적비[%]
9.7083	1046.4416	50.93
13.9950	1008.4203	49.07
	2054.8619	

<chiral-5c>

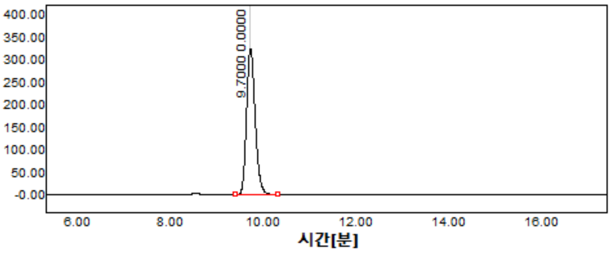

RT[분]	면적[mV*sec]	면적비[%]
9.7000	4219.1898	100.00
	4219.1898	

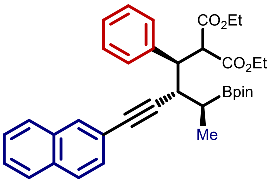

5d

Diethyl 2-(4-(naphthalen-2-yl)-1-phenyl-2-(1-(4,4,5,5-tetramethyl-1,3,2-di oxaborolan-2-yl)ethyl)but-3-yn-1-yl)malonate (5d): By following the general procedure, **5d** was obtained in 80% yield in >98:2 diastereomeric ratio (colorless oil). ^{1}H NMR (500 MHz, $CDCl_3$) δ 7.95 (s, 1H), 7.82–7.77 (m, 3H), 7.51–7.45 (m, 5H), 7.28–7.25 (m, 2H), 7.23–7.20 (m, 1H), 4.34 (d, J = 11.6 Hz, 1H), 4.33–4.27 (m, 2H), 3.86–3.84 (m, 2H), 3.70 (dd, J = 11.7, 4.1 Hz, 1H), 3.25 (dd, J = 11.1, 4.0 Hz, 1H), 1.35 (s, 6H), 1.34 (s, 6H), 1.34–1.32 (m, 3H), 1.09 (d, J = 7.5 Hz, 3H), 0.94–0.88 (m, 1H), 0.90

(t, J = 7.1 Hz, 3H); ^{13}C NMR (125 MHz, $CDCl_3$) δ 168.1, 168.0, 138.0, 128.7, 128.8, 127.7, 127.7, 127.6, 126.4, 121.3, 90.2, 86.1, 83.4, 61.6, 61.1, 57.1, 47.7, 38.2, 24.9, 24.8, 14.6, 14.2, 13.6. The carbon bound to the boron was not detected due to quadrupolar relaxation; IR (neat) 3033, 1729, 1544, 14444, 1219, 1142, 767, 685, 673 cm^{-1}; HRMS (ESI) calcd for [$C_{35}H_{41}BO_6$+Na^+]: 591.2894, found: 591.2894; >99% ee was measured by chiral HPLC on ADH column (*i*-PrOH:hexanes = 1:99, 0.5 mL/min, wavelength = 254 nm, 20 °C); t_R = 10.25 min (major), t_R = 10.87 min (minor); $[\alpha]_D^{20}$ = –120.5 (c = 1.0, $CHCl_3$).

<rac-5d>

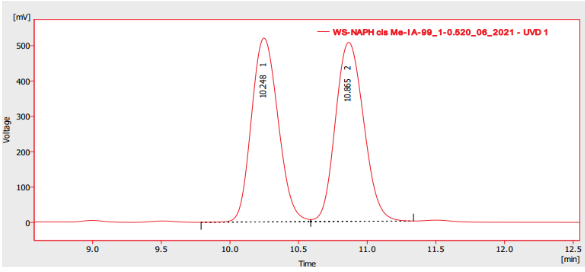

	Reten. Time [min]	Area [mV.s]	Area [%]
1	10.248	7020.491	49.2
2	10.865	7247.968	50.8
	Total	14268.459	100.0

<chiral-5d>

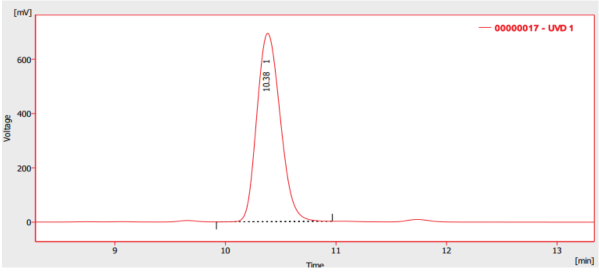

	Reten. Time [min]	Area [mV.s]	Area [%]
1	10.382	10180.243	100.0
	Total	10180.243	100.0

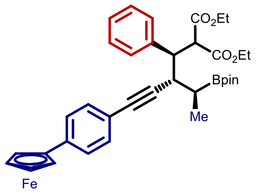

5e

Diethyl 2-(4-(4-ferrocenylphenyl)-1-phenyl-2-(1-(4,4,5,5-tetramethyl-1,3,2-dioxaborolan-2-yl)ethyl)but-3-yn-1-yl)malonate (5e): By following the general procedure, **5e** was obtained in 73% yield in >98:2 diastereomeric ratio (red solid). ^{1}H NMR (500 MHz, $CDCl_3$) δ 7.47–7.46 (m, 2H), 7.42–7.41 (m, 2H), 7.36–7.35 (m, 2H), 7.28–7.26 (m, 2H), 7.23–7.20 (m, 1H), 4.65 (t, J = 1.8 Hz, 2H), 4.33 (t, J = 1.8 Hz, 2H), 4.32–4.26 (m, 3H), 4.04 (s, 4H), 3.85–3.82 (m, 2H), 3.68 (dd, J = 11.7, 4.0 Hz, 1H), 3.20 (dd, J = 11.1, 4.0 Hz, 1H), 1.34 (s, 6H), 1.33 (s, 6H), 1.33–1.32 (m, 3H), 1.06 (d, J = 7.4 Hz, 3H), 0.91–0.86 (m, 1H), 0.89 (t, J = 7.1 Hz, 3H); ^{13}C NMR (125 MHz, $CDCl_3$) δ 168.1, 168.0, 139.1, 138.0, 131.6, 130.0, 127.7, 127.2, 125.8, 121.2, 89.8, 86.0, 83.4, 69.7, 69.2, 66.5, 61.5, 61.0, 57.1, 47.7, 38.2, 24.8, 24.9, 14.6, 14.2, 13.6. The carbon bound to the boron was not detected due to quadrupolar relaxation; IR (neat) 3033, 1729, 1544, 1444, 1380, 1219, 1142, 774, 685, 673 cm^{-1}; HRMS (ESI) calcd for [$C_{41}H_{47}BFeO_6$+Na^+]: 725.2713, found: 725.2712; >99% ee was measured by chiral HPLC on ADH column (*i*-PrOH:hexanes = 1:99, 0.5 mL/min, wavelength = 254 nm, 20 °C); t_R = 15.54 min (major), t_R = 20.79 min (minor); $[\alpha]_D^{20}$ = –77.3 (c = 0.9, $CHCl_3$).

<rac-5e>

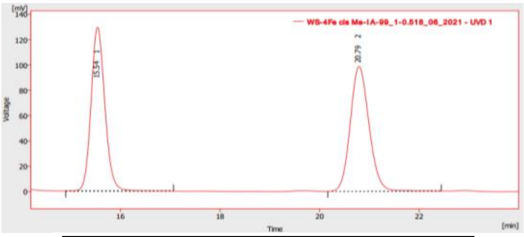

	Reten. Time [min]	Area [mV.s]	Area [%]
1	15.538	2487.758	49.1
2	20.792	2576.996	50.9
	Total	5064.754	100.0

<chiral-5e>

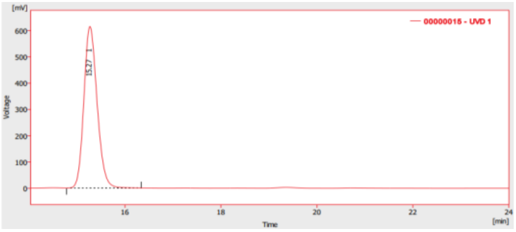

	Reten. Time [min]	Area [mV.s]	Area [%]
1	15.270	11785.756	100.0
	Total	11785.756	100.0

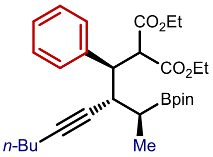

5f

Diethyl 2-(1-phenyl-2-(1-(4,4,5,5-tetramethyl-1,3,2-dioxaborolan-2-yl)ethyl)oct-3-yn-1-yl)malonate (5f): By following the general procedure, **5f** was obtained in 70% yield (colorless oil). ^{1}H NMR (500 MHz, $CDCl_3$) δ 7.40–7.39 (m, 2H), 7.24–7.16 (m, 3H), 4.36–4.20 (m, 3H), 3.86–3.79 (m, 2H), 3.54 (dd, *J* = 11.6, 4.0 Hz, 1H), 2.94–2.90 (m, 1H), 2.23 (td, *J* = 7.0, 2.3 Hz, 2H), 1.55–1.49 (m, 2H), 1.46–1.39 (m, 2H), 1.32, (s, 6H), 1.31 (s, 6H), 1.31 (t, J = 7.1 Hz, 3H), 0.95 (d, *J* = 7.5 Hz, 3H), 0.92 (t, *J* = 7.3 Hz, 3H), 0.88 (t, *J* = 7.1 Hz, 3H), 0.73–0.67 (m, 1H); ^{13}C NMR (125 MHz, $CDCl_3$) δ 168.1, 168.0, 142.1, 138.2, 129.9, 129.5, 128.8, 127.5, 127.0, 85.7, 83.2, 79.3, 61.4, 60.9, 57.1, 47.5, 37.5, 31.1, 24.8, 24.8, 21.9, 18.6, 14.5, 14.1, 13.6. The carbon bound to the boron was not detected due to quadrupolar relaxation; IR (neat) 3033, 1728, 1544, 1444, 1370, 1319, 1219, 1142, 775, 685, 672 cm^{-1}; HRMS (ESI) calcd for [$C_{29}H_{43}BO_6$+Na^+]: 521.3050, found: 521.3051; $[\alpha]_D^{20}$ = –108.4 (c = 1.0, $CHCl_3$).

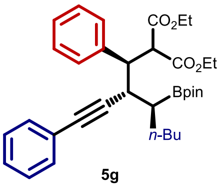

5g

Diethyl 2-(1-phenyl-2-(phenylethynyl)-3-(4,4,5,5-tetramethyl-1,3,2-dioxaborolan-2-yl)heptyl)malonate (5g): By following the general procedure, **5g** was obtained in 85% yield in >98:2 diastereomeric ratio (colorless oil). ^{1}H NMR (500 MHz, $CDCl_3$) δ 7.48–7.43 (m, 4H), 7.33–7.29 (m, 3H), 7.27–7.24 (m, 2H), 7.21–7.18 (m, 1H), 4.34–4.23 (m, 2H), 4.29 (d, *J* = 12.0 Hz, 1H), 3.83 (q, *J* = 7.1 Hz, 2H), 3.59 (dd, *J* = 11.7, 2.2 Hz, 1H), 3.23 (dd, *J* = 11.6, 2.3 Hz, 2H), 1.50–1.43 (m, 1H), 1.37 (s, 12H), 1.32 (t, *J* = 7.1 Hz, 3H), 1.28–1.19 (m, 4H), 1.18–1.11 (m, 2H), 0.89 (t, *J* = 7.1 Hz, 3H), 0.81 (t, *J* = 6.7 Hz, 3H); ^{13}C NMR (125 MHz, $CDCl_3$) δ 168.0, 167.9, 137.7, 131.6, 129.9, 128.2, 127.7, 127.6, 127.1, 124.0, 89.9, 85.7, 83.5, 61.5, 61.0, 57.0, 47.7, 37.0, 31.0, 30.0, 25.1, 25.0, 23.1, 14.2, 14.0, 13.6. The carbon bound to the boron was not detected due to quadrupolar relaxation; IR (neat) 3033, 1730, 1544, 1444, 1374, 1219, 1142, 774, 685, 673 cm^{-1}; HRMS (EI$^+$) calcd for [$C_{34}H_{45}BO_6$]: 560.3315, found: 560.3311; >99% ee was measured by chiral HPLC on ADH column (*i*-PrOH:hexanes = 1:99, 0.3 mL/min, wavelength = 254 nm, 20 °C); t_R = 11.91 min (major), t_R = 12.43 min (minor); $[\alpha]_D^{20}$ = –99.1 (c = 0.9, $CHCl_3$).

<rac-5g>

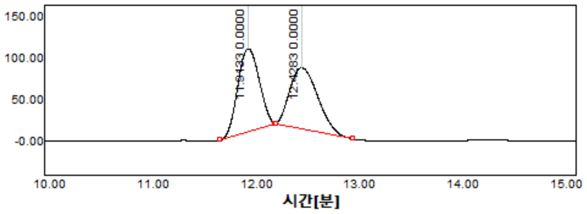

RT[분]	면적[mV*sec]	면적비[%]
11.9133	1433.2948	50.94
12.4283	1380.3491	49.06
	2813.6439	

<chiral-5g>

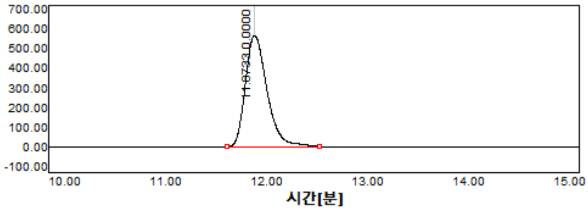

RT[분]	면적[mV*sec]	면적비[%]
11.8733	8411.1375	100.00
	8411.1375	

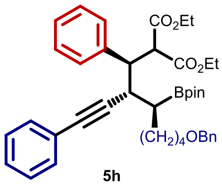

5h

Diethyl 2-(7-(benzyloxy)-1-phenyl-2-(phenylethynyl)-3-(4,4,5,5-tetramethyl-1,3,2-dioxaborolan-2-yl)heptyl)malonate (5h): By following the general procedure, **5h** was obtained in 72% yield in >98:2 diastereomeric ratio (colorless oil). ^{1}H NMR (500 MHz, $CDCl_3$) δ 7.48–7.46 (m, 2H), 7.44–7.42 (m, 2H), 7.33–7.19 (m, 11H), 4.42 (s, 2H), 4.34–4.24 (m, 2H), 4.29 (d, *J* = 11.8 Hz, 1H), 3.85–3.80 (m, 2H), 3.59 (dd, *J* = 11.7, 3.6 Hz, 1H), 3.39 (t, *J* = 6.7 Hz, 2H), 3.23 (dd, *J* = 11.6, 3.6 Hz, 1H), 1.73–1.66 (m, 1H), 1.59–1.45 (m, 3H), 1.36 (s, 6H), 1.35 (s, 6H), 1.32 (t, *J* = 7.1 Hz, 3H), 1.27–1.16 (m, 2H), 0.90–0.84 (m, 1H), 0.89 (t, *J* = 7.1 Hz, 3H); ^{13}C NMR (125 MHz, $CDCl_3$) δ 168.0, 167.9, 138.8, 137.7, 131.6, 129.9, 128.3, 128.2, 127.7, 127.5, 127.3, 127.2, 123.9, 89.8, 85.8, 83.5, 72.7, 70.4, 61.5, 61.0, 57.0, 47.7, 36.9, 30.1, 25.4, 25.0, 24.9, 14.2, 13.5. The carbon bound to the boron was not detected due to quadrupolar relaxation; IR (neat) 3033, 1729, 1544, 1444, 1372, 1219, 1141, 777, 686, 672 cm^{-1}; HRMS (FAB$^+$) calcd for [$C_{41}H_{52}BO_7$]: 667.3813, found: 667.3801; 99% ee was measured by chiral HPLC on ADH column (*i*-PrOH:hexanes = 1:99, 0.3 mL/min, wavelength = 254 nm, 20 °C); t_R = 7.78 min (major), t_R = 9.60 min (minor); $[\alpha]_D^{20}$ = –80.1 (c = 0.6, $CHCl_3$).

<rac-5h>

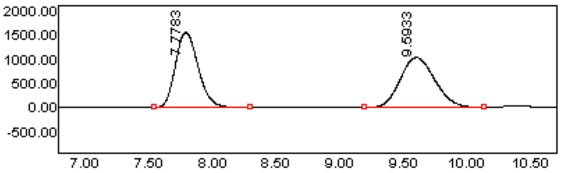

RT[분]	면적[mV*sec]	면적비[%]
7.7783	19399.9813	49.56
9.5933	19745.8469	50.44
	39145.8281	

<chiral-5h>

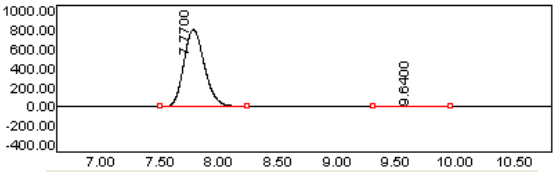

RT[분]	면적[mV*sec]	면적비[%]
7.7700	9889.2805	99.40
9.6400	59.2831	0.60
	9948.5633	

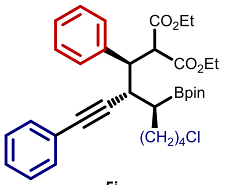

5i

Diethyl 2-(7-chloro-1-phenyl-2-(phenylethynyl)-3-(4,4,5,5-tetramethyl-1,3,2-dioxaborolan-2-yl)heptyl)malonate (5i): By following the general procedure, **5i** was obtained in 97% yield in >98:2 diastereomeric ratio (colorless oil). ^{1}H NMR (500 MHz, $CDCl_3$) δ 7.48–7.46 (m, 2H), 7.45–7.42 (m, 2H), 7.34–7.30 (m, 3H), 7.28–7.25 (m, 2H), 7.23–7.20 (m, 1H), 4.34–4.23 (m, 2H), 4.28 (d, J = 11.7 Hz, 1H), 3.85–3.81 (m, 2H), 3.60 (dd, J = 11.7, 3.7 Hz, 1H), 3.45 (t, J = 6.7 Hz, 2H), 3.24 (dd, J = 11.5, 3.7 Hz, 1H), 1.74–1.63 (m, 3H), 1.51–1.43 (m, 2H), 1.38 (s, 6H), 1.37 (s, 6H), 1.32 (t, J = 7.1 Hz, 3H), 1.29–1.21 (m, 1H), 0.90–0.84 (m, 1H), 0.89 (t, J = 7.1 Hz, 3H); ^{13}C NMR (125 MHz, $CDCl_3$) δ 168.0, 167.8, 137.6, 131.6, 129.9, 128.3, 127.8, 127.7, 127.2, 123.8, 89.6, 85.9, 83.6, 61.5, 61.1, 56.9, 47.7, 45.0, 36.8, 32.9, 29.5, 26.1, 25.1, 25.0, 14.2, 13.6. The carbon bound to the boron was not detected due to quadrupolar relaxation; IR (neat) 3033, 1729, 1544, 1444, 1372, 1219, 1141, 770, 686, 672 cm^{-1}; HRMS (EI$^+$) calcd for [$C_{34}H_{44}BClO_6$]: 594.2926, found: 594.2917; 99% ee was measured by chiral HPLC on ADH column (*i*-PrOH:hexanes = 1:99, 0.3 mL/min, wavelength = 254 nm, 20 °C); t_R = 14.31 min (major), t_R = 15.22 min (minor); $[\alpha]_D^{20}$ = –128.2 (c = 1.0, $CHCl_3$).

<rac-5i>

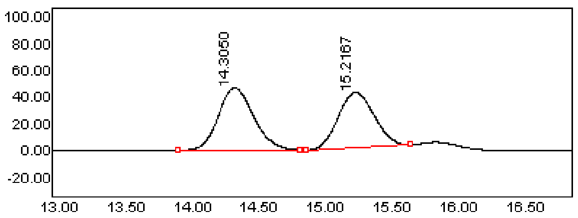

RT[분]	면적[mV*sec]	면적비[%]
14.3050	858.8601	52.54
15.2167	775.9665	47.46
	1634.8266	

<chiral-5i>

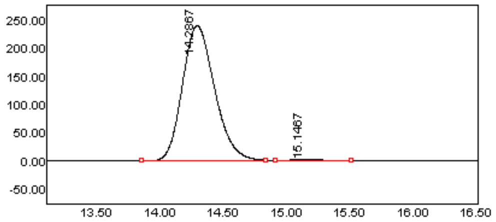

RT[분]	면적[mV*sec]	면적비[%]
14.2867	4253.4344	99.13
15.1467	37.3962	0.87
	4290.8305	

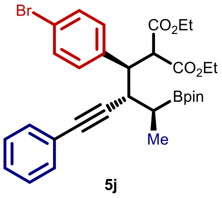

5j

Diethyl 2-(1-(4-bromophenyl)-4-phenyl-2-(1-(4,4,5,5-tetramethyl-1,3,2-dioxaborolan-2-yl)ethyl)but-3-yn-1-yl)malonate (5j): By following the general procedure, **5j** was obtained in 84% yield in >98:2 diastereomeric ratio (colorless oil). ^{1}H NMR (500 MHz, $CDCl_3$) δ 7.44–7.31 (m, 9H), 4.32–4.25 (m, 2H), 4.22 (d, J = 11.7 Hz, 1H), 3.91–3.84 (m, 2H), 3.64 (dd, J = 11.7, 4.0 Hz, 1H), 3.16 (dd, J = 11.2, 4.0 Hz, 1H), 1.33 (s, 6H), 1.32 (s, 6H), 1.32–1.30 (m, 3H), 1.04 (d, J = 7.4 Hz, 3H), 0.95 (t, J = 7.1 Hz, 3H), 0.83–0.79 (m, 1H); ^{13}C NMR (125 MHz, $CDCl_3$) δ 167.8, 167.7, 137.1, 131.6, 131.5, 130.8, 128.3, 127.9, 123.7, 121.3, 89.3, 86.0, 83.4, 61.6, 61.2, 56.8, 47.0, 38.0, 29.7, 24.9, 24.8, 14.5, 14.1, 13.7. The carbon bound to the boron was not detected due to quadrupolar relaxation; IR (neat) 3033, 1730, 1544, 1444, 1369, 1219, 1142, 773, 686, 672 cm^{-1}; HRMS (EI$^+$) calcd for [$C_{31}H_{38}BBrO_6$]: 596.1950, found: 596.1943; 99% ee was measured by chiral HPLC on ADH column (*i*-PrOH:hexanes = 1:99, 0.3 mL/min,

wavelength = 254 nm, 20 °C); t_R = 8.41 min (major), t_R = 9.55 min (minor); $[\alpha]_D^{20}$ = –109.3 (c = 1.0, $CHCl_3$).

<rac-5j>

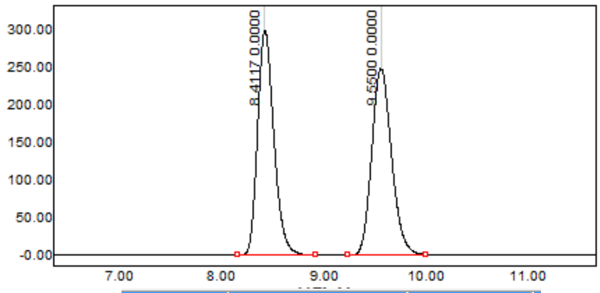

RT[분]	면적[mV*sec]	면적비[%]
8.4117	3341.9199	49.86
9.5500	3361.0844	50.14
	6703.0047	

<chiral-5j>

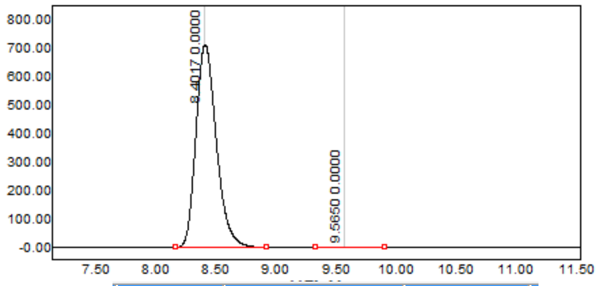

RT[분]	면적[mV*sec]	면적비[%]
8.4017	8400.7719	99.72
9.5650	23.6674	0.28
	8424.4391	

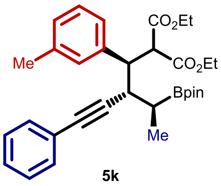

5k

Diethyl 2-(4-phenyl-2-(1-(4,4,5,5-tetramethyl-1,3,2-dioxaborolan-2-yl)ethyl)-1-(m-tolyl)but-3-yn-1-yl)malonate (5k): By following the general procedure, **5k** was obtained in 91% yield in >98:2 diastereomeric ratio (colorless oil). ^{1}H NMR (500 MHz, $CDCl_3$) δ 7.45–7.43 (m, 2H), 7.33–7.25 (m, 5H), 7.15–7.12 (m, 1H), 7.02–7.00 (m, 1H), 4.32–4.24 (m, 2H), 4.28 (d, J = 11.7 Hz, 1H), 3.85 (q, J = 7.1 Hz, 2H), 3.63 (dd, J = 11.6, 4.1 Hz, 1H), 3.17 (dd, J = 11.1, 4.1 Hz, 1H), 2.29 (s, 3H), 1.34 (s, 6H), 1.33 (s, 6H) 1.33–1.31 (m, 3H), 1.04 (d, J = 7.5 Hz, 3H), 0.90 (t, J = 7.1 Hz, 3H), 0.87–0.83 (m, 1H); ^{13}C NMR (125 MHz, $CDCl_3$) δ 168.1, 167.9, 137.8, 136.8, 131.6, 130.7, 128.2, 127.9, 127.6, 127.5, 126.8, 124.0, 90.0, 85.7, 53.3, 61.5, 61.0, 57.1, 47.5, 38.0, 24.9, 24.8, 21.5, 14.6, 14.2, 13.6. The carbon bound to the boron was not detected due to quadrupolar relaxation; IR (neat) 3033, 1729, 1544, 1444, 1369, 1219, 1142, 767, 686, 672 cm^{-1}; HRMS (ESI) calcd for $[C_{32}H_{41}BO_6+Na^+]$: 555.2894, found: 555.2897; >99% ee was measured by chiral HPLC on ADH column (i-PrOH:hexanes = 1:99, 0.3 mL/min, wavelength = 254 nm, 20 °C); t_R = 7.89 min (major), t_R = 8.87 min (minor); $[\alpha]_D^{20}$ = –127.5 (c = 1.1, $CHCl_3$).

<rac-5k>

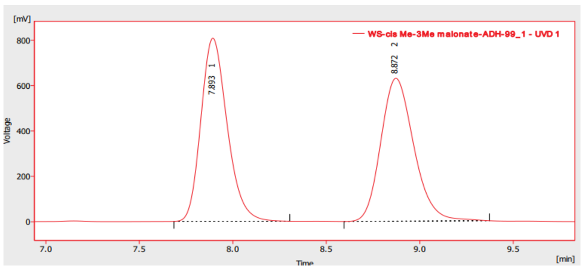

	Reten. Time [min]	Area [mV.s]	Area [%]
1	7.893	7889.500	50.5
2	8.872	7739.157	49.5
	Total	15628.657	100.0

<chiral-5k>

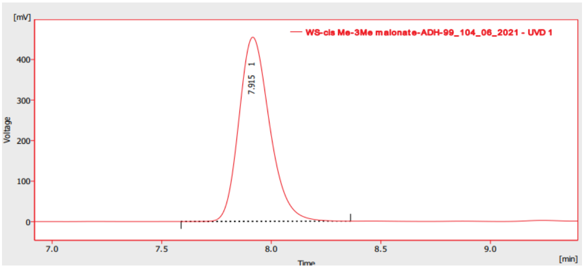

	Reten. Time [min]	Area [mV.s]	Area [%]
1	7.915	4426.872	100.0
	Total	4426.872	100.0

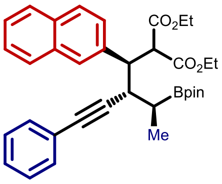

5l

Diethyl 2-(1-(naphthalen-2-yl)-4-phenyl-2-(1-(4,4,5,5-tetramethyl-1,3,2-dioxaborolan-2-yl)ethyl)but-3-yn-1-yl)malonate (5l): By following the general procedure, **5l** was obtained in 97% yield in 98:2 diastereomeric ratio (white solid). ^{1}H NMR (500 MHz, $CDCl_3$) δ 7.89 (s, 1H), 7.79–7.73 (m, 3H), 7.67–7.66 (m, 1H), 7.48–7.46 (m, 2H), 7.43–7.41 (m, 2H), 7.35–7.30 (m, 3H), 4.42 (d, J = 11.7 Hz, 1H), 4.36–4.26 (m, 2H), 3.87 (dd, J = 11.7, 4.1 Hz, 1H), 3.82–3.71 (m, 2H), 3.26 (dd, J = 11.1, 4.1 Hz, 1H), 1.36 (s, 6H), 1.35 (s, 6H), 1.35–1.33 (m, 3H), 1.03 (d, J = 7.4 Hz, 3H), 0.87–0.83 (m, 1H), 0.79 (t, J = 7.1 Hz, 3H); ^{13}C NMR (125 MHz, $CDCl_3$) δ 168.1, 167.9, 135.7, 133.0, 132.8, 131.6, 128.3, 128.0, 127.7, 127., 127.2, 125.7, 125.5, 124.0, 89.9, 85.9, 83.4, 61.6, 61.0, 57.1, 47.6, 38.3, 24.9, 24.8, 14.6, 14.2, 13.6. The carbon bound to the boron was not detected due to quadrupolar relaxation; IR (neat) 3033, 1729, 1544, 1443, 1368, 1219, 1142, 777, 686, 672 cm^{-1}; HRMS (ESI) calcd for [$C_{35}H_{41}BO_6$+Na^+]: 591.2894, found: 591.2894; 99% ee was measured by chiral HPLC on ADH column (i-PrOH:hexanes = 1:99, 0.3 mL/min, wavelength = 254 nm, 20 °C); t_R = 16.47 min (major), t_R = 19.61 min (minor); $[\alpha]_D^{20}$ = –146.3 (c = 1.4, $CHCl_3$).

<rac-5l>

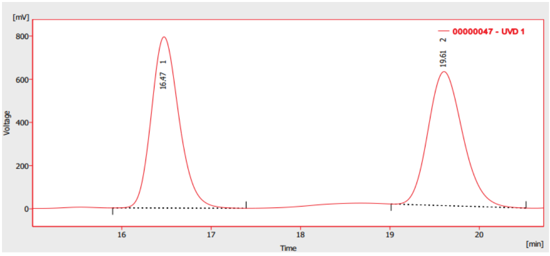

<chiral-5l>

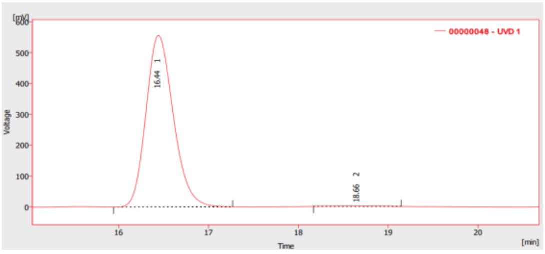

Reten. Time [min]	Area [mV.s]	Area [%]
16.442	11800.925	99.6
18.655	46.386	0.4
Total	11847.311	100.0

Reten. Time [min]	Area [mV.s]	Area [%]
16.473	17050.399	50.5
19.605	16724.139	49.5
Total	33774.538	100.0

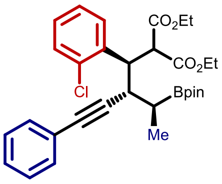

5m

Diethyl 2-(1-(2-chlorophenyl)-4-phenyl-2-(1-(4,4,5,5-tetramethyl-1,3,2-dioxaborolan-2-yl)ethyl)but-3-yn-1-yl)malonate (5m): By following the general procedure, **5m** was obtained in 65% yield in >98:2 diastereomeric ratio (white solid). ^{1}H NMR (500 MHz, $CDCl_3$) δ 7.78–7.77 (m, 1H), 7.42–7.40 (m, 2H), 7.34–7.29 (m, 4H), 7.20–7.17 (m, 1H), 7.15–7.12 (m, 1H), 4.38–4.25 (m, 3H), 4.22 (d, J = 11.4 Hz, 1H), 3.90–3.84 (m, 2H), 3.44 (dd, J = 8.5, 4.2 Hz, 1H), 1.34 (t, J = 7.1 Hz, 3H), 1.29 (s, 6H), 1.27 (s, 6H), 1.06–0.98 (m, 1H), 0.97 (d, J = 7.1 Hz, 3H), 0.93 (t, J = 7.1 Hz, 3H); ^{13}C NMR (125 MHz, $CDCl_3$) δ 167.9, 167.5, 136.7, 135.7, 131.5, 129.9, 129.3, 128.2, 128.1, 127.7, 126.2, 123.9, 89.4, 86.5, 83.4, 61.6, 61.2, 57.4, 42.3, 38.0, 25.0, 24.7, 14.1, 13.9, 13.6. The carbon bound to the boron was

not detected due to quadrupolar relaxation; IR (neat) 3033, 1730, 1544, 1443, 1219, 1142, 773, 685, 673 cm^{-1}; HRMS (ESI) calcd for [$C_{31}H_{38}BClO_6$+Na^+]: 575.2348, found: 575.2347; 99% ee was measured by chiral HPLC on ADH column (*i*-PrOH:hexanes = 1:99, 0.3 mL/min, wavelength = 254 nm, 20 °C); t_R = 15.93 min (major), t_R = 24.25 min (minor); $[\alpha]_D^{20}$ = –72.6 (c = 0.7, $CHCl_3$).

<rac-5m>

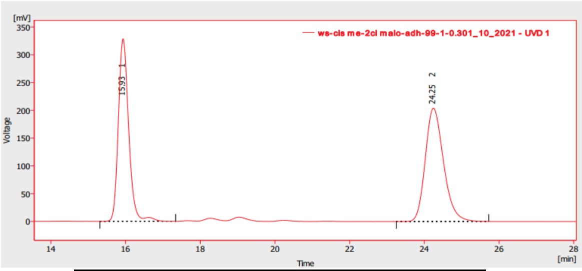

	Reten. Time [min]	Area [mV.s]	Area [%]
1	15.933	6331.798	47.7
2	24.245	6928.838	52.3
	Total	13260.636	100.0

<chiral-5m>

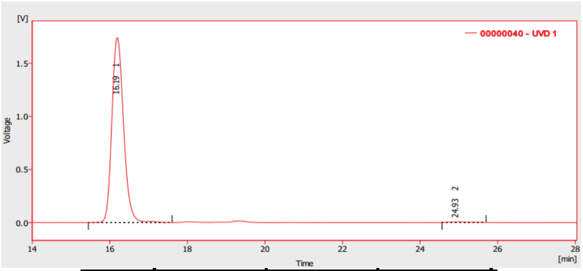

	Reten. Time [min]	Area [mV.s]	Area [%]
1	16.192	36540.797	99.5
2	24.932	183.008	0.5
	Total	36723.805	100.0

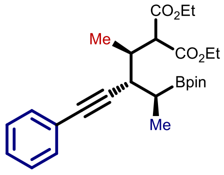

5n

Diethyl 2-(5-phenyl-3-(1-(4,4,5,5-tetramethyl-1,3,2-dioxaborolan-2-yl)ethyl)pent-4-yn-2-yl)malonate (5n): By following the general procedure, **5n** was obtained in 16% yield in >98:2 diastereomeric ratio (colorless oil). ^{1}H NMR (500 MHz, $CDCl_3$) δ 7.42–7.41 (m, 2H), 7.30–7.26 (m, 3H), 4.24–4.15 (m, 4H), 3.57 (d, *J* = 9.7 Hz, 1H), 2.80 (dd, *J* = 10.3, 3.9 Hz, 1H), 2.54–2.47 (m, 1H), 1.29–1.25 (m, 19H), 1.16 (d, *J* = 7.3 Hz, 3H), 1.08 (d, *J* = 6.7 Hz, 3H); ^{13}C NMR (125 MHz, $CDCl_3$) δ 168.7, 168.4, 131.7, 128.1, 127.5, 124.0, 89.8, 84.2, 83.3, 61.2, 61.2, 57.3, 38.5, 36.3, 24.8, 24.7, 14.3, 14.1, 14.0, 13.0. The carbon bound to the boron was not detected due to quadrupolar relaxation; IR (neat) 3019, 1728, 1460, 1370, 1321, 1217, 1143, 755, 692, 668 cm^{-1}; HRMS (ESI) calcd for [$C_{26}H_{37}BO_6$+Na^+]: 479.2581, found: 479.2581; >99% ee was measured by chiral HPLC on ADH column (*i*-PrOH:hexanes = 1:99, 0.5 mL/min, wavelength = 254 nm, 20 °C); t_R = 13.42 min (major), t_R = 14.23 min (minor).

<rac-5n>

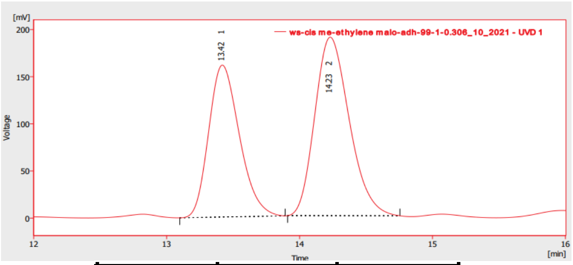

Reten. Time [min]	Area [mV.s]	Area [%]
13.418	2589.641	43.1
14.230	3413.175	56.9
Total	6002.816	100.0

<chiral-5n>

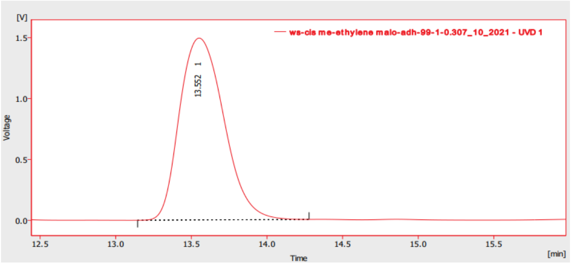

Reten. Time [min]	Area [mV.s]	Area [%]
13.552	30379.897	100.0
Total	30379.897	100.0

Organic transformations of 3a and characterization of products in Fig. 5c in the main text.

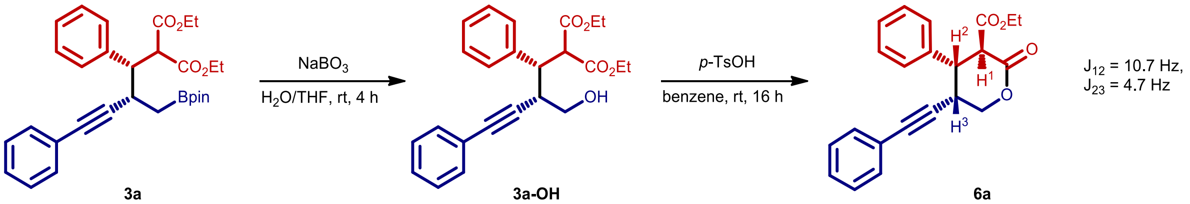

3a **3a-OH** **6a**

Ethyl 2-oxo-4-phenyl-5-(phenylethynyl)tetrahydro-2H-pyran-3-carboxylate (6a) Sodium perborate was sequentially added to a stirred solution of **3a** in H_2O/THF (5 mL). The reaction was stirred for 4 h at room temperature and quenched by water. The mixture was extracted by ethyl acetate, dried over $MgSO_4$, filtered and concentrated. The crude mixture was purified by flash column chromatography, and **3a-OH** was obtained as a colorless oil. To a solution **3a-OH** in benzene (2 mL) was added *p*-toluenesulfonic acid (0.1 equiv.). The reaction mixture was stirred for 16 h at room temperature. The reaction mixture was concentrated in vacuo and purified by flash chromatography, and **6a** was obtained as a colorless oil in 85% overall yield. ^{1}H NMR (500 MHz, $CDCl_3$) δ 7.39–7.36 (m, 2H), 7.32–7.19 (m, 8H), 4.66 (dd, *J* = 11.4, 4.7 Hz, 1H), 4.46 (t, *J* = 11.0 Hz, 1H), 4.12 (q, *J* = 7.1 Hz, 2H), 3.79 (d, *J* = 10.7 Hz, 1H), 3.66 (t, *J* = 10.7 Hz, 1H), 3.31 (td, *J* = 10.6, 4.7 Hz, 1H), 1.12 (t, *J* = 7.1 Hz, 3H); ^{13}C NMR (125 MHz, $CDCl_3$) δ 167.4, 166.2, 139.0, 131.6, 128.9, 128.5, 128.2, 128.0, 127.5, 122.2, 85.5, 84.1, 71.0, 62.0, 54.6, 47.6, 34.1, 13.9; IR (neat) 3032, 1734, 1492, 1472, 1219, 1150, 772, 688, 672 cm^{-1}; HRMS (ESI) calcd for [$C_{22}H_{20}O_4$+Na$^+$]: 371.1259, found: 371.1259.

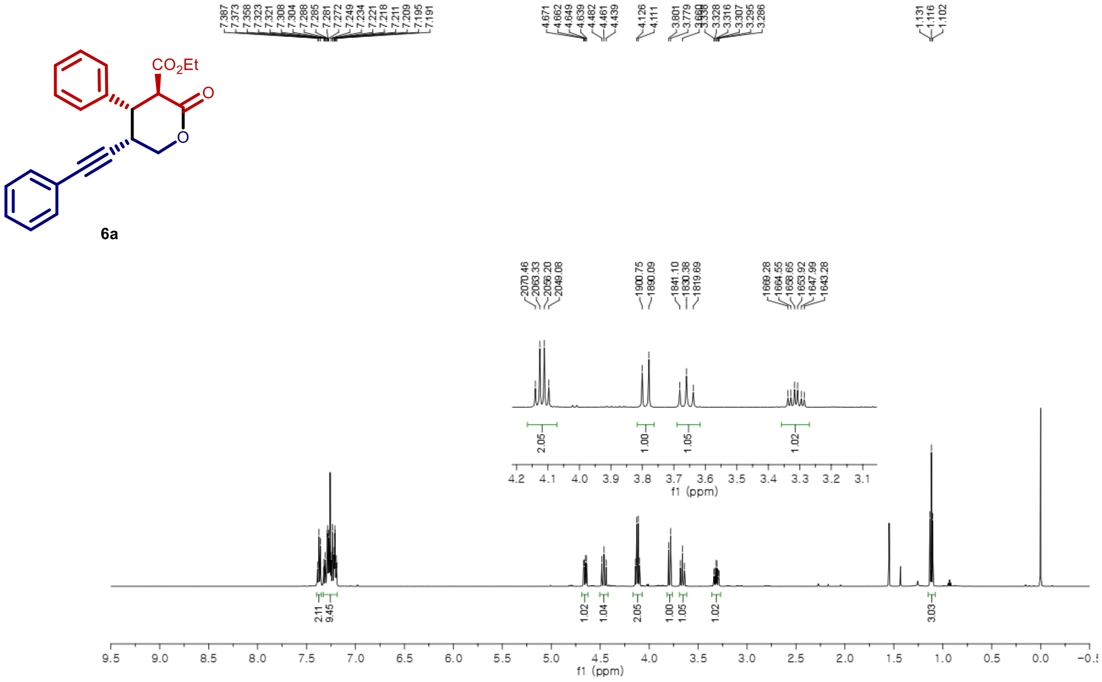

Supplementary Figure 1. ^{1}H NMR of compound **6a**.

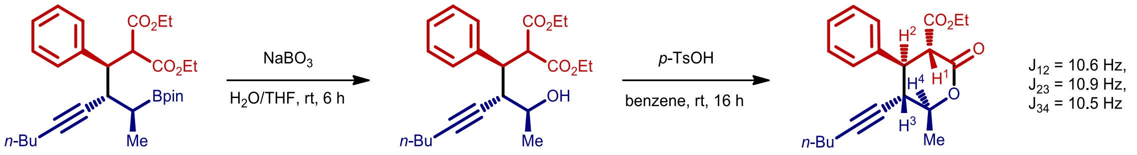

5f **5f-OH** **6f**

Ethyl 5-(hex-1-yn-1-yl)-6-methyl-2-oxo-4-phenyltetrahydro-2H-pyran-3-carboxylate (6f) Sodium perborate was sequentially added to a stirred solution of **5f** in H_2O/THF (5 mL) at 0 °C. The reaction was stirred for 6 h at room temperature and quenched by water. The mixture was extracted by ethyl acetate, dried over $MgSO_4$, filtered and concentrated. The crude mixture was purified by flash column chromatography, and **5f-OH** was obtained as a colorless oil in 49% yield. To a solution **5f-OH** in benzene (2 mL) was added *p*-toluenesulfonic acid (0.1 equiv.). The reaction mixture was stirred for 16 h at room temperature. The reaction mixture was concentrated in vacuo and purified by flash chromatography, and **6f** was obtained as a colorless oil in 72% yield (overall 37% yield). ^{1}H NMR (500 MHz, $CDCl_3$) δ 7.34–7.31 (m, 2H), 7.28–7.27 (m, 1H), 7.20–7.18 (m, 2H), 4.50 (dq, J = 10.5, 6.3 Hz, 1H), 4.11–4.07 (m, 2H), 3.66 (d, J = 10.6 Hz, 1H), 3.49 (t, J = 10.9 Hz, 1H), 2.74–2.69 (m, 1H), 1.98 (td, J = 6.9, 2.2 Hz, 2H), 1.57 (d, J = 6.3 Hz, 3H), 1.27–1.21 (m, 2H), 1.16–1.08 (m, 2H), 1.09 (t, J = 7.1 Hz, 3H), 0.77 (t, J = 7.3 Hz, 3H); ^{13}C NMR (125 MHz, $CDCl_3$) δ 167.7, 166.4, 139.4, 128.7, 127.7, 127.6, 86.5, 19.9, 15.6, 61.9, 55.1, 47.7, 40.3, 30.5, 21.5, 20.5, 18.1, 13.9, 13.5; IR (neat) 3032, 1731, 1445, 1219, 1141, 772, 685, 672 cm^{-1}; HRMS (ESI) calcd for [$C_{21}H_{26}O_4$+Na^+]: 365.1729, found: 365.1728; >99% ee was measured by chiral HPLC on OJH column (*i*-PrOH:hexanes = 5:95, 0.5 mL/min, wavelength = 254 nm, 20 °C); t_R = 13.40 min (major), t_R = 15.54 min (minor).

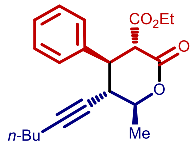

6f

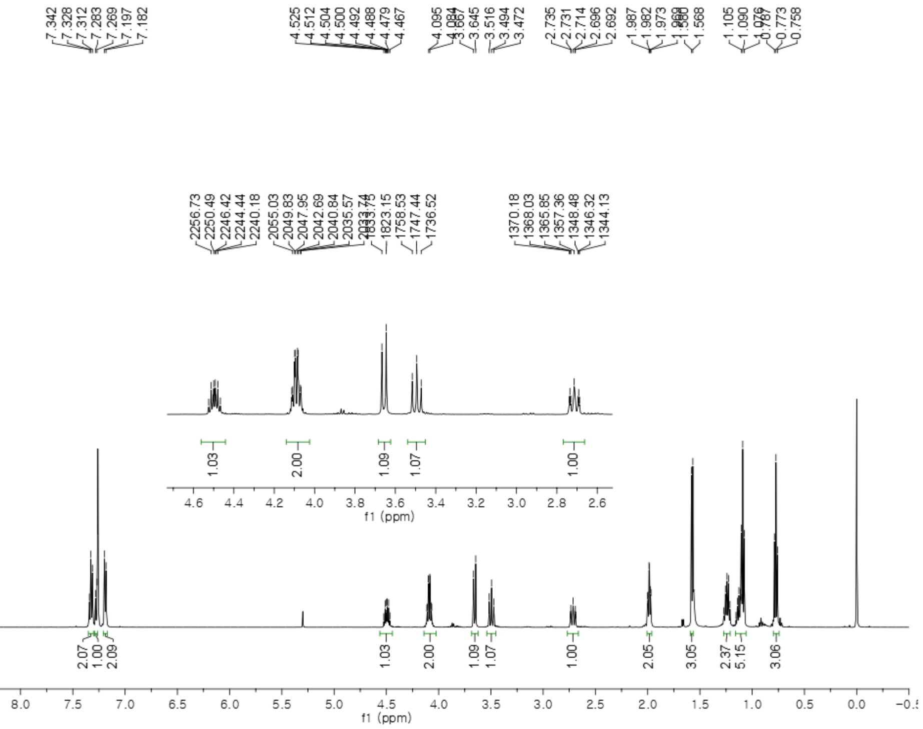

Supplementary Figure 2. ^{1}H NMR of compound **6f**.

<rac-6f>

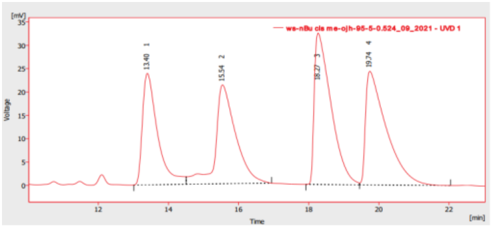

	Reten. Time [min]	Area [mV.s]	Area [%]
1	13.398	715.708	19.7
2	15.542	850.998	23.4
3	18.268	1073.686	29.5
4	19.743	1000.104	27.5
	Total	3640.496	100.0

<chiral-6f>

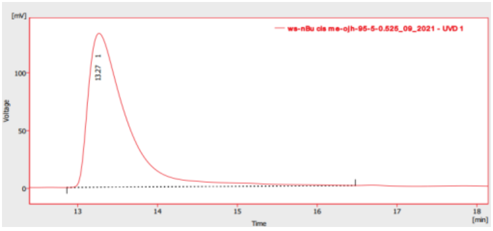

	Reten. Time [min]	Area [mV.s]	Area [%]
1	13.267	4423.178	100.0
	Total	4423.178	100.0

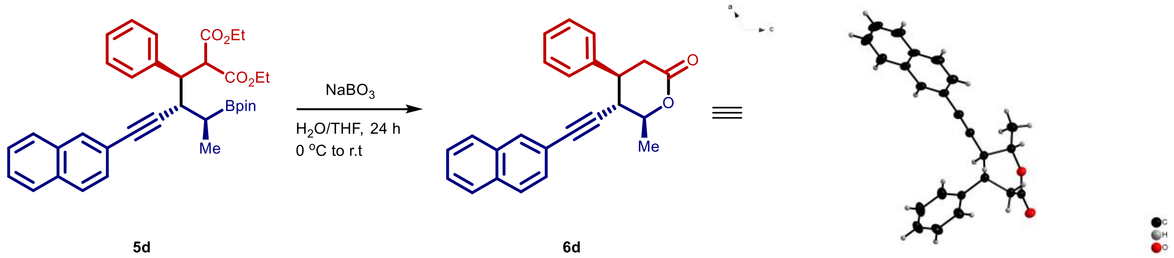

5d **6d**

6-methyl-5-(naphthalen-2-ylethynyl)-4-phenyltetrahydro-2H-pyran-2-one (6d) Sodium perborate was sequentially added to a stirred solution of **5d** in H_2O/THF (5 mL) at 0 °C. The reaction was stirred for 24 h at room temperature and quenched by water. The mixture was extracted by ethyl acetate, dried over $MgSO_4$, filtered and concentrated. The crude mixture was purified by flash column chromatography,

and **6d** was obtained as a white solid in 59% yield. ^{1}H NMR (500 MHz, $CDCl_3$) δ 7.78–7.17 (m, 1H), 7.74–7.69 (m, 3H), 7.48–7.45 (m, 2H), 7.41–7.38 (m, 2H), 7.33–7.30 (m, 3H), 7.24–7.22 (m, 1H), 4.58 (dq, *J* = 10.3, 6.2 Hz, 1H), 3.36 (dt, *J* = 10.0, 6.9 Hz, 1H), 3.04 (dd, *J* = 17.7, 6.9 Hz, 1H), 2.90 (t, *J* = 10.5 Hz, 1H), 2.76 (dd, *J* = 17.7, 9.6 Hz, 1H), 1.69 (d, *J* = 6.3 Hz, 3H); ^{13}C NMR (125 MHz, $CDCl_3$) δ 170.2, 141.6, 131.4, 128.9, 128.2, 127.9, 127.7, 127.6, 127.5, 127.2, 126.7, 126.6, 86.3, 86.0, 78.9, 44.1, 41.8, 36.5, 20.7; HRMS (ESI) calcd for [$C_{24}H_{20}O_2$+Na^+]: 363.1361, found: 363.1363.

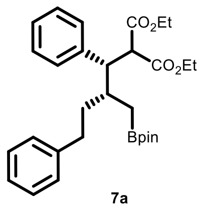

7a

Ethyl 3,6-diphenyl-4-((4,4,5,5-tetramethyl-1,3,2-dioxaborolan-2-yl)methyl)hexanoate (7a) A Pd/C (15 mg) was added to the solution of **3a** (0.3 mmol) in THF (3 mL) and stirred for 4 h under H_2 atmosphere. After completion of reaction, the mixture was filtered through a celite and evaporate under reduced pressure. The crude mixture was purified the residue by column chromatography and **7a** was obtained in 98% yield, >98:2 d.r. ^{1}H NMR (500 MHz, CDCl3) δ 7.26–7.14 (m, 8H), 7.16–7.11 (d, *J* = 7.2 Hz, 2H), 4.21 (q, *J* = 7.1 Hz, 2H), 4.02 (d, *J* = 11.3 Hz, 1H), 3.89–3.82 (m, 2H), 3.57 (dd, *J* = 11.3, 4.6 Hz, 1H), 2.63 (td, *J* = 12.5, 4.8 Hz, 1H), 2.43 (dd, *J* = 11.8, 5.3 Hz, 1H), 2.04–2.03 (m, 1H), 1.88–1.81 (m, 1H), 1.30 (t, *J* = 7.3 Hz, 6H), 1.29 (s, 6H), 1.28 (s, 6H), 1.26–1.17 (m, 1H), 0.92 (t, *J* = 7.1 Hz, 3H), 0.88 (dd, *J* = 15.5, 6.0 Hz, 1H), 0.68 (dd, *J* = 15.5, 8.3 Hz, 1H); ^{13}C NMR (125 MHz, CDCl3) δ 142.8, 138.5, 129.7, 128.4, 128.3, 127.8, 126.7, 125.6, 83.2, 61.5, 61.0, 55.8, 50.2, 37.2, 34.3, 34.1, 25.0, 24.9, 15.0 (C-B), 14.2, 13.7; IR (neat) 3027, 1731, 1603, 1453, 1370, 1216, 1097, 753 cm^{-1} ; HRMS (ESI) calcd for [$C_{30}H_{41}BO_6$+Na^+]: 531.2894, found: 531.2894.

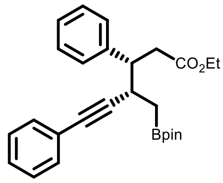

8a

Ethyl 3,6-diphenyl-4-((4,4,5,5-tetramethyl-1,3,2-dioxaborolan-2-yl)methyl)hex-5-ynoate (8a) **3a** (0.6 mmol) and water (90 μL) were added to a solution of NaCl (3 equiv, 1.8 mmol) in DMSO (3 mL) at room temperature. The reaction mixture was stirred for 24 h at 160 °C. Upon completion of the reaction, the reaction mixture was quenched with water and extracted with ethyl acetate. The combined organic layers were dried over $MgSO_4$ and concentrated in vacuo. The crude product was purified by flash chromatography, and **8a** was obtained in 85% yield, >98:2 d.r. ^{1}H NMR (500 MHz, CDCl3) δ 7.36–7.33 (m, 4H), 7.28–7.25 (m, 5H), 7.22–7.19 (m, 1H), 4.02 (qd, *J* = 7.1, 1.5 Hz, 2H), 3.34–3.30 (m, 1H), 3.20 (td, *J* = 8.0, 4.9 Hz, 1H), 2.98–2.87 (m, 2H), 1.23 (s, 12H), 1.11 (t, *J* = 7.1, 3H), 0.94 (d, *J* = 8.0, 2H); ^{13}C NMR (125 MHz, CDCl3) δ 172.3, 140.3, 131.5, 129.0, 128.1, 127.9, 127.6, 126.9, 124.0, 91.9, 83.4, 83.3, 60.3, 46.5, 38.8, 33.3, 25.0, 24.6, 16.3 (C-B), 14.1; IR (neat) 3029, 2336, 1733, 1370, 1217, 1144, 771 cm^{-1} ; HRMS (ESI) calcd for [$C_{27}H_{33}BO_4$+Na^+]: 455.2370, found: 455.2370.

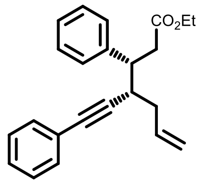

9a

Ethyl 3-phenyl-4-(phenylethynyl)hept-6-enoate (9a) Vinylmagnesium bromide solution (1.0 M in THF, 4 equiv, 0.68 mmol) was added to a solution of **8a** (1 equiv, 0.17 mmol) in THF (2 mL) and the reaction mixture was stirred for 30 min at –78 °C. A solution of iodine (4 equiv, 0.68 mmol) in MeOH (2 mL) was added. The mixture was stirred for another 30 min, and allowed to warm to 0 °C. Upon completion of the reaction, the reaction mixture was quenched with sat. $Na_2S_2O_3$ (aq) and extracted with ethyl acetate. The combined organic layers were dried over $MgSO_4$ and concentrated in vacuo. The crude product was purified by flash chromatography, to yield **9a** in 74% yield, >98:2 d.r. ^{1}H NMR (500 MHz, CDCl3) δ 7.40–7.38 (m, 4H), 7.31–7.28 (m, 5H), 7.26–7.23 (m, 1H), 5.93–5.85 (m, 1H), 5.09 (d, *J* = 6.8 Hz, 1H), 5.06 (s, 1H), 4.05 (q, *J* = 7.2 Hz, 2H), 3.38 (td, *J* = 7.6, 4.7 Hz, 1H), 3.07–3.03 (m, 1H), 2.99 (dd, *J* = 15.9, 7.3 Hz, 1H), 2.88 (dd, *J* = 15.9, 8.0 Hz, 1H), 2.20–2.15 (m, 1H), 2.12–2.04 (m, 1H), 1.14 (t, *J* = 7.1 Hz, 3H); ^{13}C NMR (125 MHz, CDCl3) δ 172.2, 140.2, 136.0, 131.6, 128.9, 128.2, 128.0, 127.8, 127.0, 123.7, 116.8, 90.1, 84.6, 60.4, 44.1, 39.1, 37.6, 37.5, 14.1; IR (neat) 3032, 2408, 1733, 1442, 1219, 1160, 772 cm^{-1} ; HRMS (ESI) calcd for $[C_{23}H_{24}BO_2+Na^+]$: 355.1674, found: 355.1675; 97:3 er was measured by chiral HPLC on IA column (iPrOH:hexanes = 0.5:99.5, 0.5 mL/min, wavelength = 254 nm, 20 °C); t_R = 9.70 min (major), t_R = 9.23 min (minor)

<rac-9a>

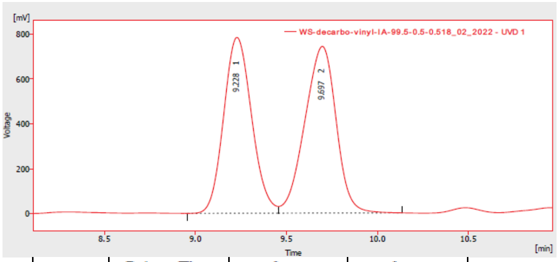

	Reten. Time [min]	Area [mV.s]	Area [%]
1	9.228	8374.442	48.5
2	9.697	8874.667	51.5
	Total	17249.110	100.0

<chiral-9a>

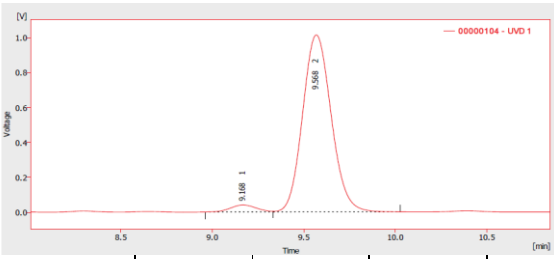

	Reten. Time [min]	Area [mV.s]	Area [%]
1	9.168	400.517	3.5
2	9.568	11024.961	96.5
	Total	11425.479	100.0

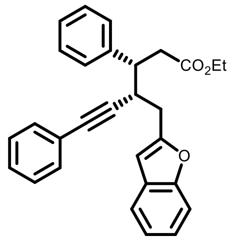

10a

Ethyl 4-(benzofuran-2-ylmethyl)-3,6-diphenylhex-5-ynoate (10a) A solution of benzofuran (0.4 mmol) in THF (1 mL) was cooled to –78 °C and treated with *n*-BuLi (1.6 M in hexanes, 0.4 mmol). The cooling bath was removed, and the mixture was stirred at room temperature for 1 h. The mixture was cooled to –78 °C and a solution of **8a** (0.17 mmol) in THF (1 mL) was added. The mixture was stirred at –78 °C for 1 h and then, a solution of NBS (0.4 mmol) in THF (1 mL) was added. After 1 h at –78 °C, sat. $Na_2S_2O_3$ (aq) was added and the reaction mixture was allowed to room temperature. The reaction mixture was diluted with water and extracted with ethyl acetate. The combined organic layers were dried over $MgSO_4$ and concentrated in vacuo. The crude product was

purified by column chromatography, and **10a** was obtained in 54% yield, >98:2 d.r. ^{1}H NMR (500 MHz, CDCl3) δ 7.50–7.49 (m, 1H), 7.45–7.42 (m, 3H), 7.36–7.33 (m, 4H), 7.29–7.28 (m, 4H), 7.24–7.17 (m, 2H), 6.50 (s, 1H), 4.05 (q, *J* = 6.9 Hz, 2H), 3.60–3.57 (m, 1H), 3.46–3.42 (m, 1H), 3.04 (dd, *J* = 16.0, 7.3 Hz, 1H), 2.94 (dd, *J* = 16.0, 8.0 Hz, 1H), 2.87 (dd, *J* = 15.0, 6.6 Hz, 1H), 2.80 (dd, *J* = 15.0, 8.3 Hz, 1H), 1.13 (t, *J* = 7.1 Hz, 3H); ^{13}C NMR (125 MHz, CDCl3) δ 171.9, 156.2, 154.8, 139.6, 131.6, 129.1, 128.8, 128.3, 128.1, 128.0, 127.3, 123.4, 122.5, 120.5, 110.9, 103.8, 89.2, 85.1, 60.5, 43.9, 39.2, 36.2, 32.7, 14.1; IR (neat) 3031, 2408, 1732, 1454, 1219, 1162, 772 cm^{-1} ; HRMS (ESI) calcd for [$C_{29}H_{26}BO_3$+Na^+]: 445.1780, found: 445.1779; 96:4 er was measured by chiral HPLC on IA column (iPrOH:hexanes = 1:99, 0.5 mL/min, wavelength = 254 nm, 20 °C); t_R = 12.63 min (major), t_R = 11.99 min (minor).

<rac-10a>

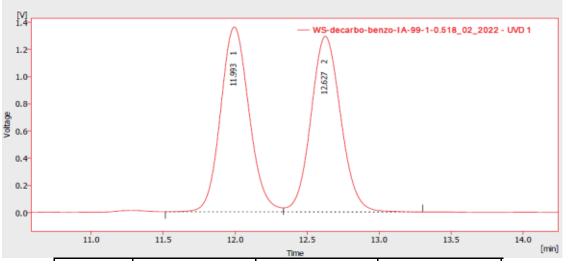

	Reten. Time [min]	Area [mV.s]	Area [%]
1	11.993	18702.934	50.1
2	12.627	18622.365	49.9
	Total	37325.299	100.0

<chiral-10a>

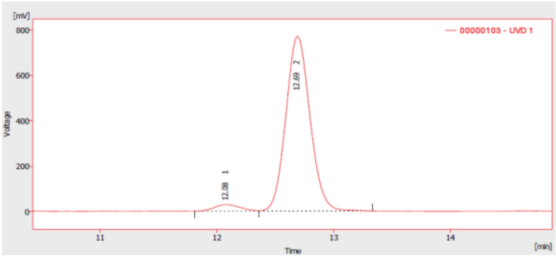

	Reten. Time [min]	Area [mV.s]	Area [%]
1	12.078	432.929	3.8
2	12.690	11038.737	96.2
	Total	11471.666	100.0

Ⅲ. Supplementary Discussion

Supplementary Table 1. Hammett plot.

Substituent	δp	%ee	log(er)
OMe	-0.27	95	1.6
Me	-0.17	94	1.5
H	0	93	1.4
Br	0.23	91	1.3
CO_2Me	0.45	25	0.3
CF_3	0.54	57	0.6

The data used in Fig. 5a in the main text.

Radical trapping experiments with TEMPO[7].

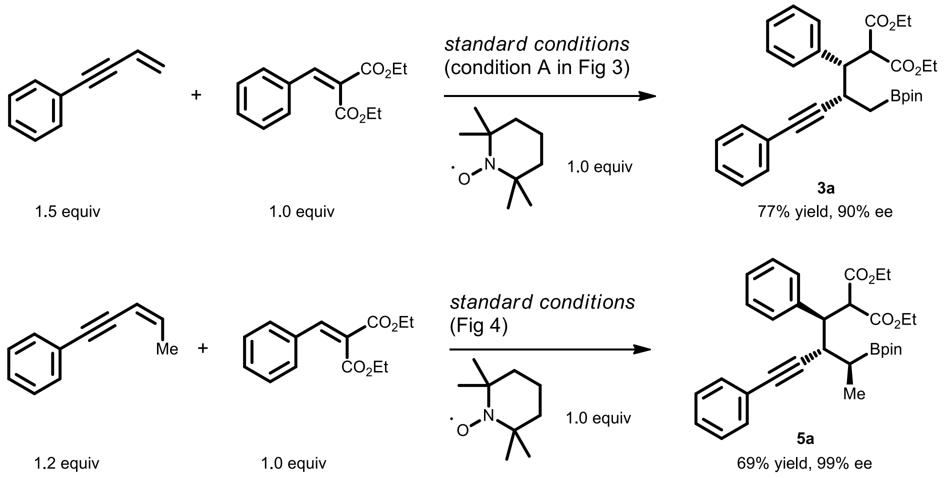

Scaled-up reaction of coupling 1a and 2a.

A mixture of CuCl (3 mol %, 6 mg), (*R,S*)-Josiphos (3 mol %, 39 mg), LiO*t*-Bu (1.5 equiv, 240 mg), and B_2pin_2 (1.5 equiv, 762 mg) in THF (3 mL) was stirred for 15 min in a Schlenk tube under an atmosphere of nitrogen. Substrate **1a** (3 mmol, 385 mg) and **2a** (2 mmol, 497 mg) dissolved in THF (1 mL) were added to the reaction mixture at 0 °C. The reaction mixture was stirred at 0 °C and monitored by TLC. After completion of reaction, the reaction mixture was diluted with water. The aqueous layer was extracted with dichloromethane and washed with brine, the combined organic layers were dried over $MgSO_4$, and concentrated in vacuo. The product **3a** was purified by silica gel chromatography and obtained in 61 % yield (1.23 mmol, 620 mg) with >98:2 dr, 92% ee.

<chiral-3a>

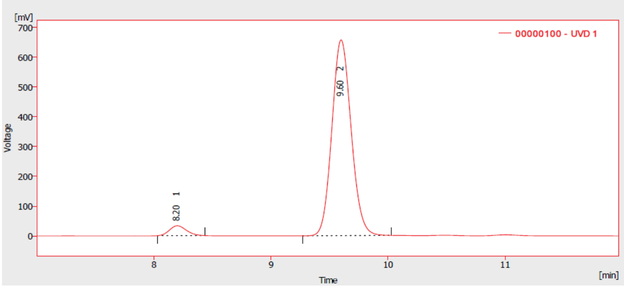

	Reten. Time [min]	Area [mV.s]	Area [%]
1	8.200	330.841	4.2
2	9.598	7522.178	95.8
	Total	7853.019	100.0

Supplementary Table 2. Extended screening table of chiral ligands.

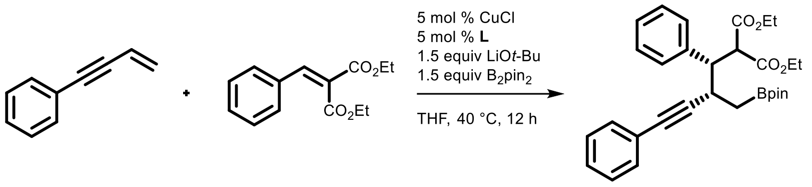

entry	ligand	yield (%)[a]	dr[b]	%ee[c]
1	**L1**	91	>98:2	-
2	**L2**	45	>98:2	0
3	(*R*)-**L3**	35	>98:2	0
4	(*R,R*)-**L4**	41	>98:2	0
5	(*S,S*)-**L5**	95	>98:2	45
6	(*R,S*)-**L6**	97	>98:2	86
7	(*R,S*)-**L7**	72	>98:2	17
8	(*R,S*)-**L8**	96	>98:2	14
9	(*R,S*)-**L9**	90	>98:2	74
10	(*R,S*)-**L10**	66	>98:2	30

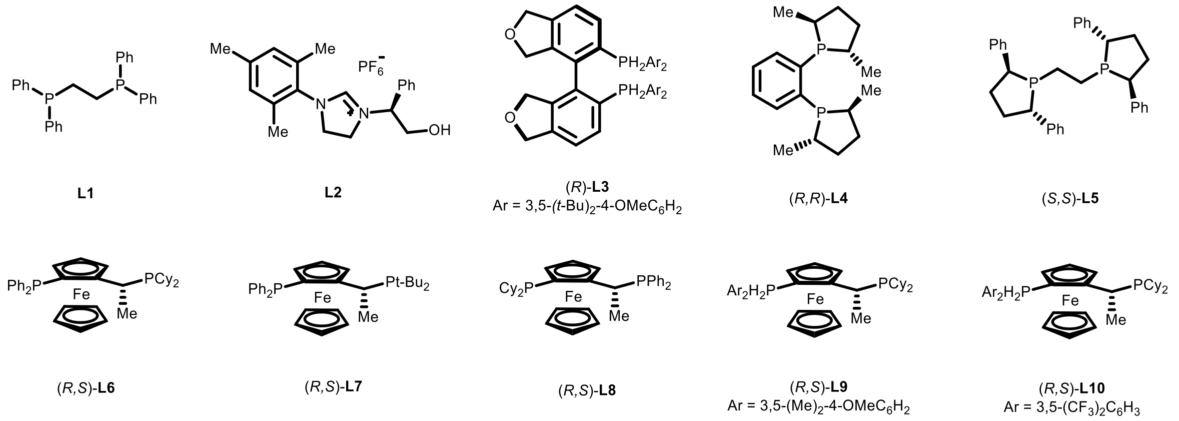

General reaction conditions: **1a** (0.75 mmol), **2a** (0.5 mmol), CuCl (0.025 mmol), **L** (0.025 mmol), LiO*t*-Bu (0.75 mmol), B_2pin_2 (0.75 mmol) in THF (1 mL) at 40 ºC. [a]Isolated yield. [b]Diastereomeric ratio (dr) was determined by ^{1}H NMR analysis of a crude reaction mixture. [c]Enantiomeric excess (ee) was determined by chiral HPLC analysis.

Supplementary Table 3. Screening of copper sources.

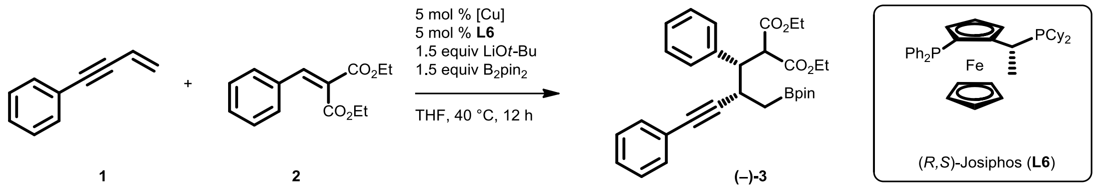

entry	[Cu]	yield (%)[a]	dr[b]	%ee[c]
1	$Cu(CH_3CN)_4PF_6$	98	>98:2	36
2	$Cu(OAc)_2$	97	>98:2	44
3	CuTc	95	>98:2	35
4	CuBr	97	>98:2	85
5	CuI	75	>98:2	84
6[d]	CuCl	94	>98:2	93

General reaction conditions: **1a** (0.75 mmol), **2a** (0.5 mmol), [Cu] (0.025 mmol), **L6** (0.025 mmol), LiO*t*-Bu (0.75 mmol), B_2pin_2 (0.75 mmol) in THF (1 mL) at 40 ºC. CuTc = Copper thiophene-2-carboxylate. [a]Isolated yield. [b]Diastereomeric ratio (dr) was determined by ^1H NMR analysis of a crude reaction mixture. [c]Enantiomeric excess (ee) was determined by chiral HPLC analysis. [d]The reaction was performed at 0 ºC for 24 h.

Supplementary Table 4. Screening of internal enynes.

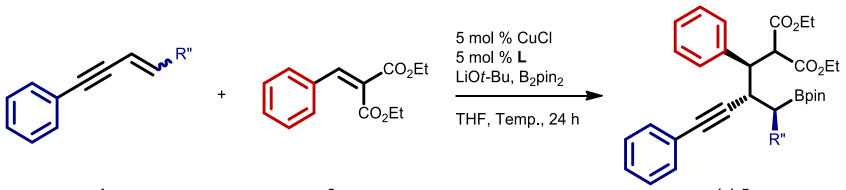

4 2 (–)-5

Entry	R″	Ligand	Temperature	Yield (%)[a]	dr[b]	%ee[c]
1[d]	cis-Me	**L6**	0	73	>98:2	52
2[d]	cis-Me	**L5**	0	45	>98:2	99
3	cis-Me	**L5**	40	92	>98:2	>99
4	cis-*n*-Bu	**L5**	40	85	>98:2	99
5	trans-*n*-Bu	**L5**	40	40	80:20	97(+)

General reaction conditions: **1** (0.6 mmol), **2a** (0.5 mmol), CuCl (0.025 mmol), **L** (0.025 mmol), LiO*t*-Bu (0.75 mmol), B_2pin_2 (0.6 mmol) in THF (1 mL) at 40 ºC. [a]Isolated yield. [b]Diastereomeric ratio (dr) was determined by ^{1}H NMR analysis of a crude reaction mixture. [c]Enantiomeric excess (ee) was determined by chiral HPLC analysis. [d]The reaction was performed using **1** (0.75 mmol), B_2pin_2 (0.75 mmol).

Supplementary Table 5. Crystal data and structure refinement for compound **3a**.

Empirical formula	C30 H37 B O6	
Formula weight	504.40	
Temperature	200 K	
Wavelength	1.54184 Å	
Crystal system	Orthorhombic	
Space group	$P2_12_12_1$	
Unit cell dimensions	a = 8.70170(10) Å	α= 90°.
	b = 10.4140(2) Å	β= 90°.
	c = 31.4514(4) Å	γ = 90°.
Volume	2850.11(7) $Å^3$	
Z	4	
Density (calculated)	1.176 Mg/m^3	
Absorption coefficient	0.644 mm^{-1}	
F(000)	1080	
Crystal size	0.14 x 0.08 x 0.06 mm^3	
Theta range for data collection	2.810 to 74.494°.	
Index ranges	-10<=h<=10, -12<=k<=13, -39<=l<=39	
Reflections collected	19020	
Independent reflections	5814 [R(int) = 0.0298]	
Completeness to theta = 67.684°	99.9 %	
Absorption correction	Semi-empirical from equivalents	
Max. and min. transmission	1.0000 and 0.86733	
Refinement method	Full-matrix least-squares on F^2	
Data / restraints / parameters	5814 / 0 / 438	
Goodness-of-fit on F^2	1.059	
Final R indices [I>2sigma(I)]	R1 = 0.0360, wR2 = 0.0901	
R indices (all data)	R1 = 0.0398, wR2 = 0.0924	
Absolute structure parameter	0.03(8)	
Extinction coefficient	n/a	
Largest diff. peak and hole	0.126 and -0.194 e.$Å^{-3}$	

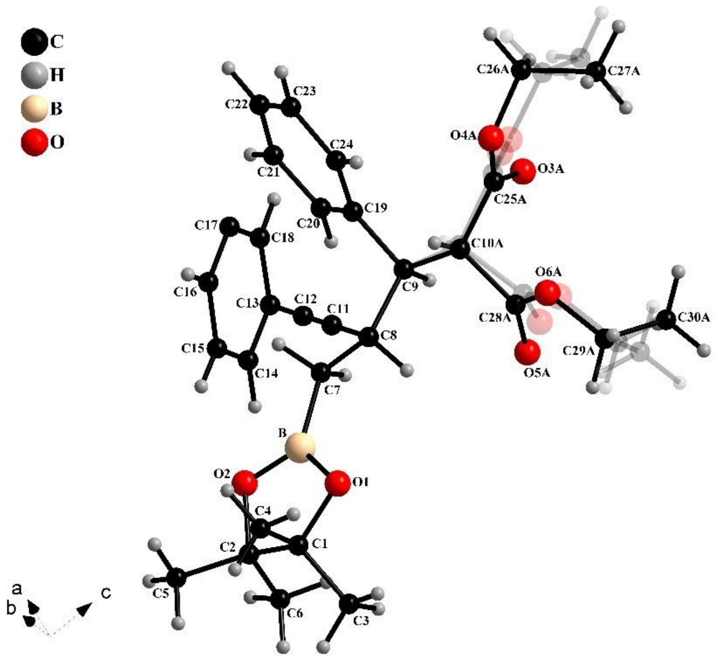

C
H
B
O
C26A
C27A
C22
C23
O4A
O3A
C24
C21
C25A
C19
C17
C20
C18
C10A
C9
O6A
C16
C13
C12
C11
C28A
C30A
C8
C29A
C15
C14
O5A
C7
B
O2
O1
C4
C2
C1
C5
C6
C3
a
b
c

Supplementary Table 6. Crystal data and structure refinement for compound **5m**.

Empirical formula	C31 H38 B Cl O6	
Formula weight	552.87	
Temperature	290(1) K	
Wavelength	0.7107 Å	
Crystal system	Monoclinic	
Space group	$P2_1$	
Unit cell dimensions	a = 10.6389(13) Å	α= 90°.
	b = 9.0576(9) Å	β = 94°.
	c = 16.7744(17) Å	γ = 90°.
Volume	1612.0(3) $Å^3$	
Z	2	
Density (calculated)	1.139 Mg/m^3	
Absorption coefficient	0.156 mm^{-1}	
F(000)	588	
Crystal size	0.88 x 0.40 x 0.10 mm^3	
Theta range for data collection	3.144 to 24.998°.	
Index ranges	-12<=h<=12, -10<=k<=9, -19<=l<=19	
Reflections collected	10724	
Independent reflections	5022 [R(int) = 0.0313]	
Completeness to theta = 24.998°	99.9 %	
Absorption correction	Semi-empirical from equivalents	
Max. and min. transmission	1.0000 and 0.5056	
Refinement method	Full-matrix least-squares on F^2	
Data / restraints / parameters	5022 / 1 / 353	
Goodness-of-fit on F^2	1.030	
Final R indices [I>2sigma(I)]	R1 = 0.0540, wR2 = 0.1401	
R indices (all data)	R1 = 0.0937, wR2 = 0.1626	
Absolute structure parameter	0.00(4)	
Extinction coefficient	n/a	
Largest diff. peak and hole	0.282 and -0.156 e.$Å^{-3}$	

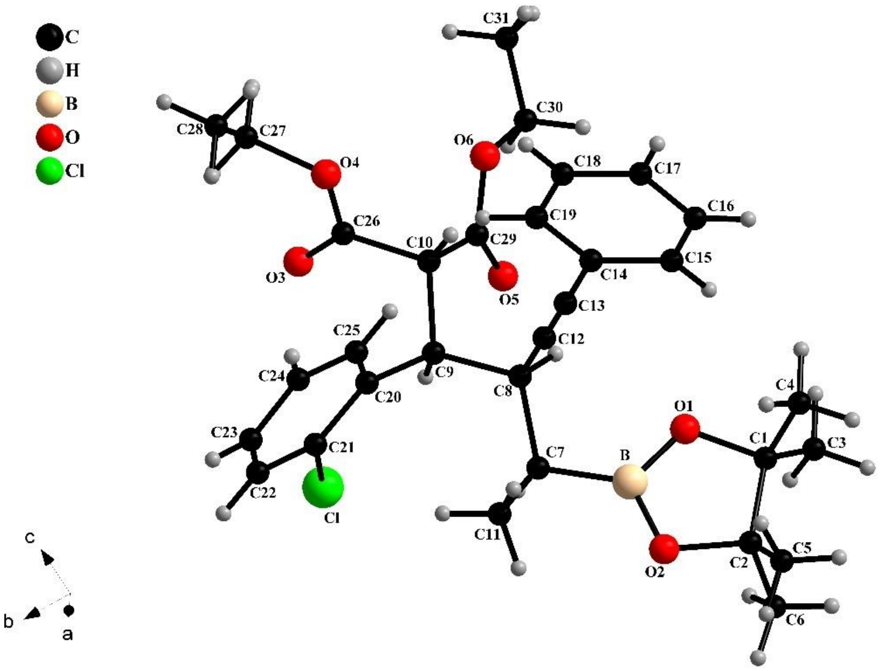
C
H
B
O
Cl
C31
C30
C28
C27
O6
O4
C18
C17
C26
C19
C16
C10
C29
C14
C15
O3
O5
C13
C25
C12
C9
C8
C24
C20
C4
O1
C23
C21
C7
B
C1
C3
C22
Cl
C11
O2
C2
C5
C6
c
b
a

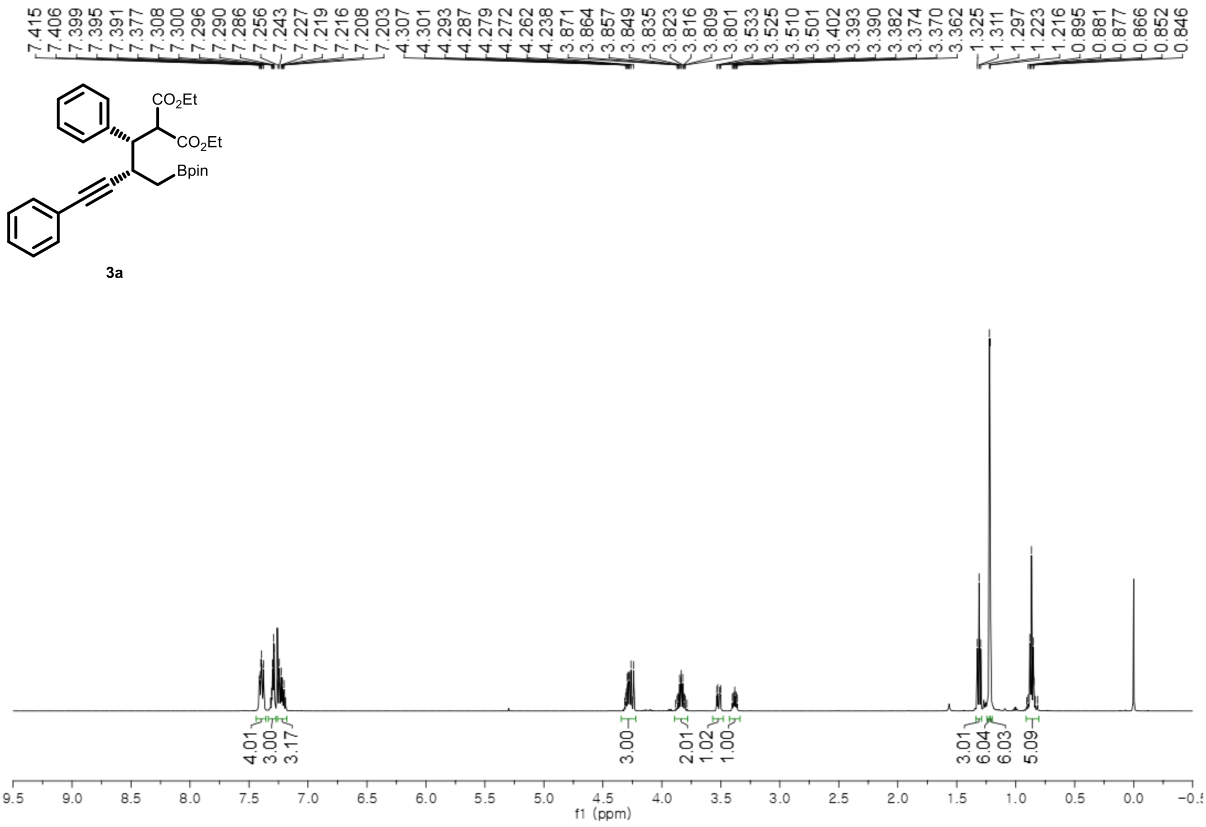

Supplementary Figure 3. ^{1}H NMR of compound **3a**.

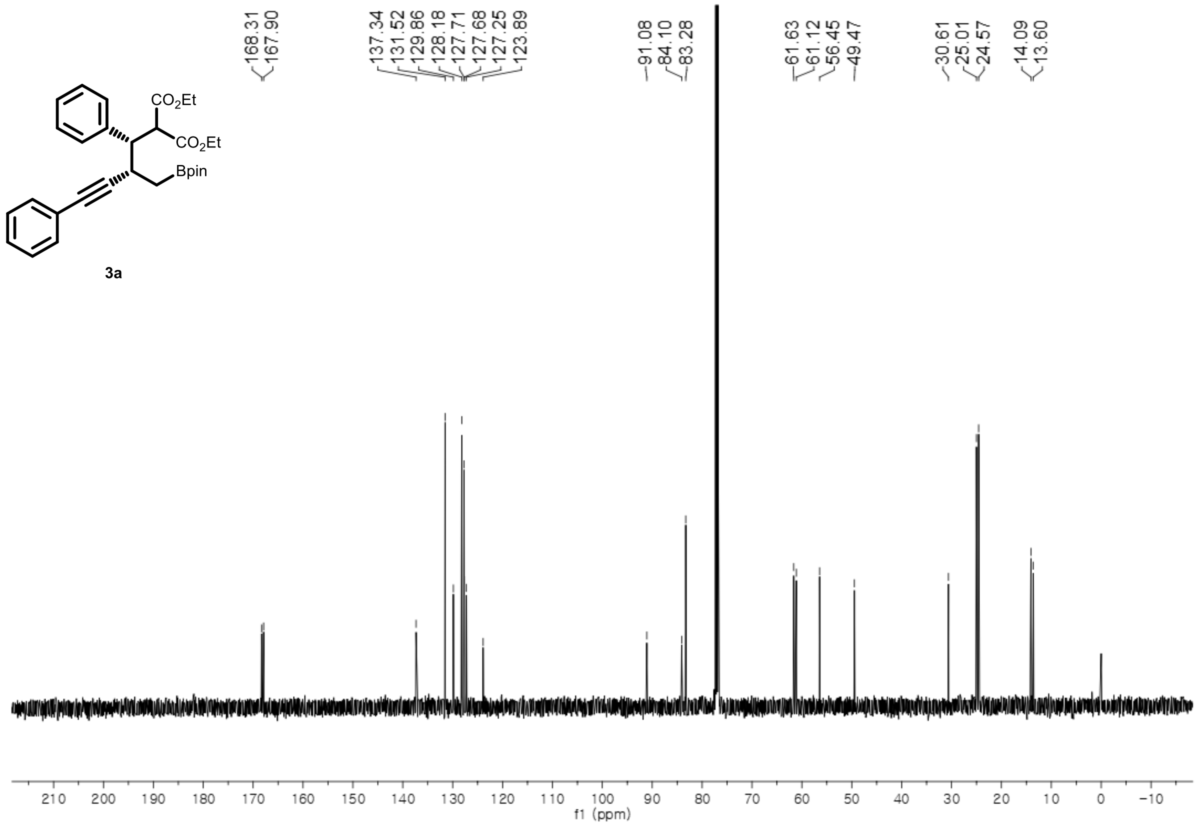

Supplementary Figure 4. ^{13}C NMR of compound **3a**.

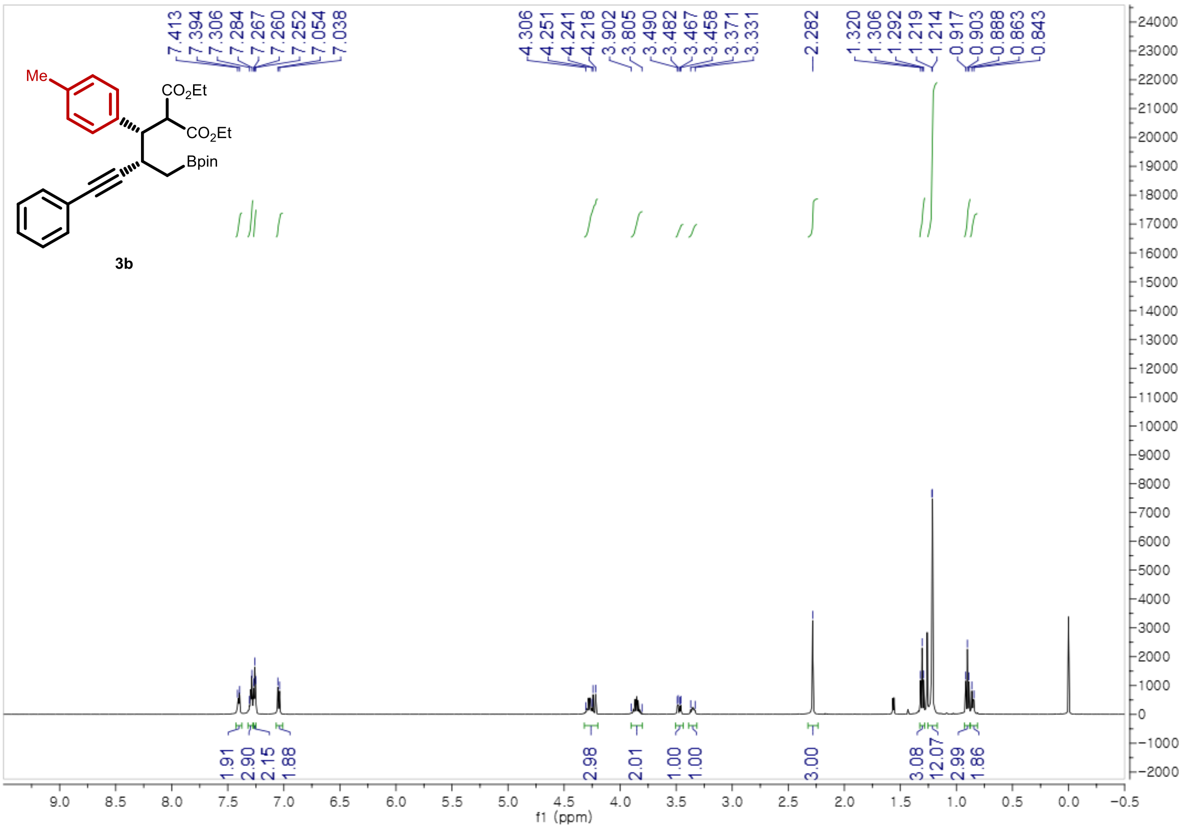

Supplementary Figure 5. ^{1}H NMR of compound **3b**.

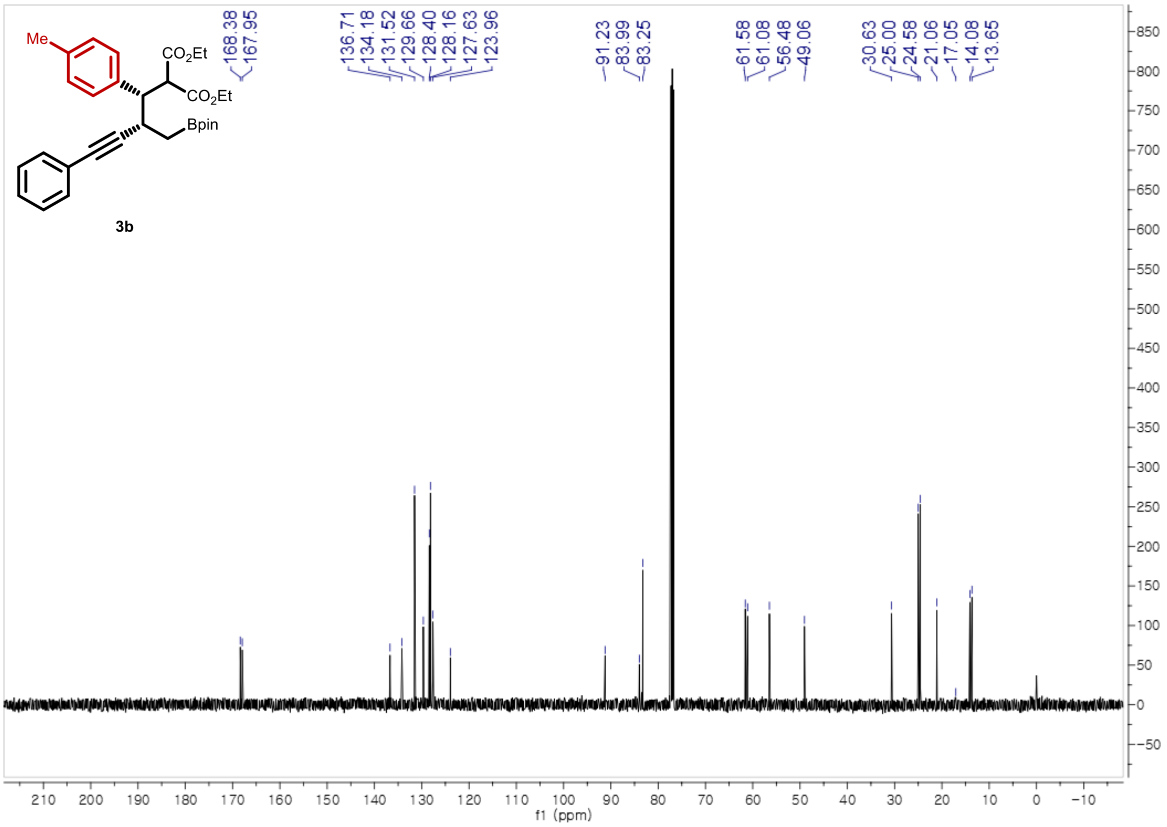

Supplementary Figure 6. ^{13}C NMR of compound **3b**.

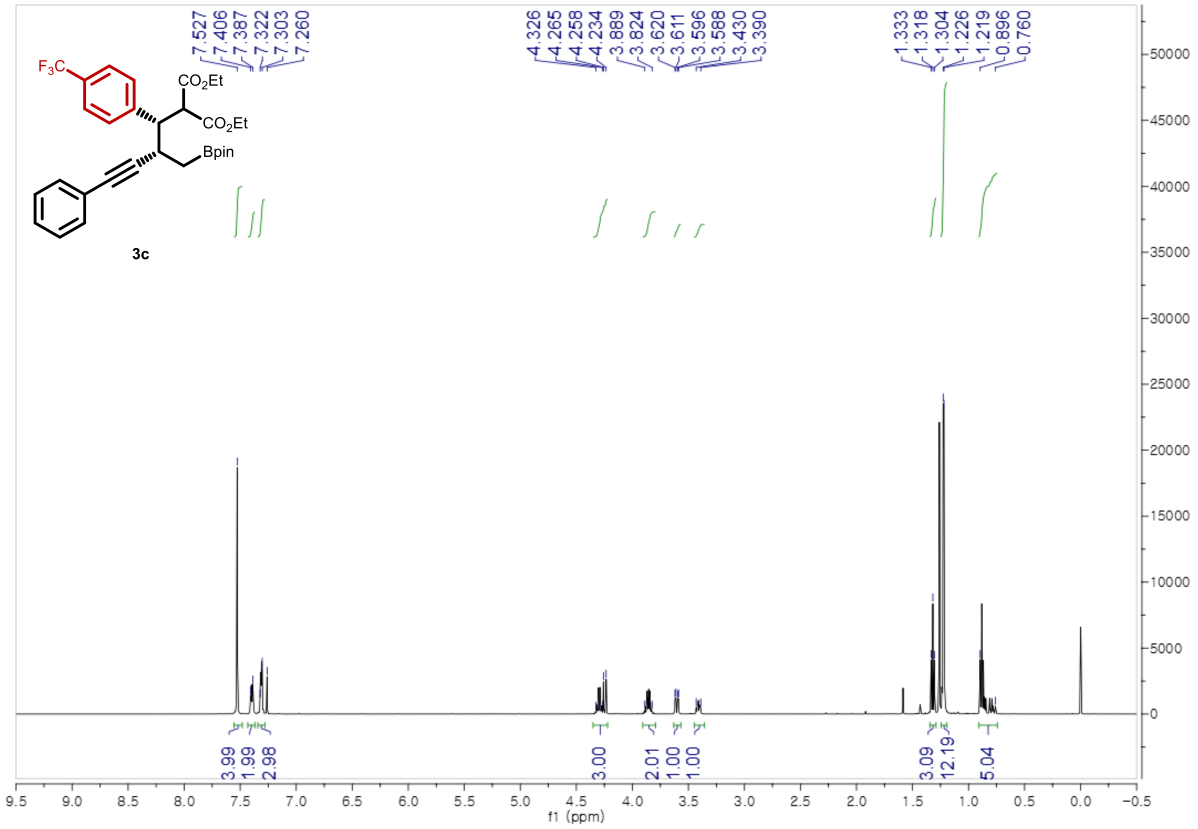

Supplementary Figure 7. ^{1}H NMR of compound **3c**.

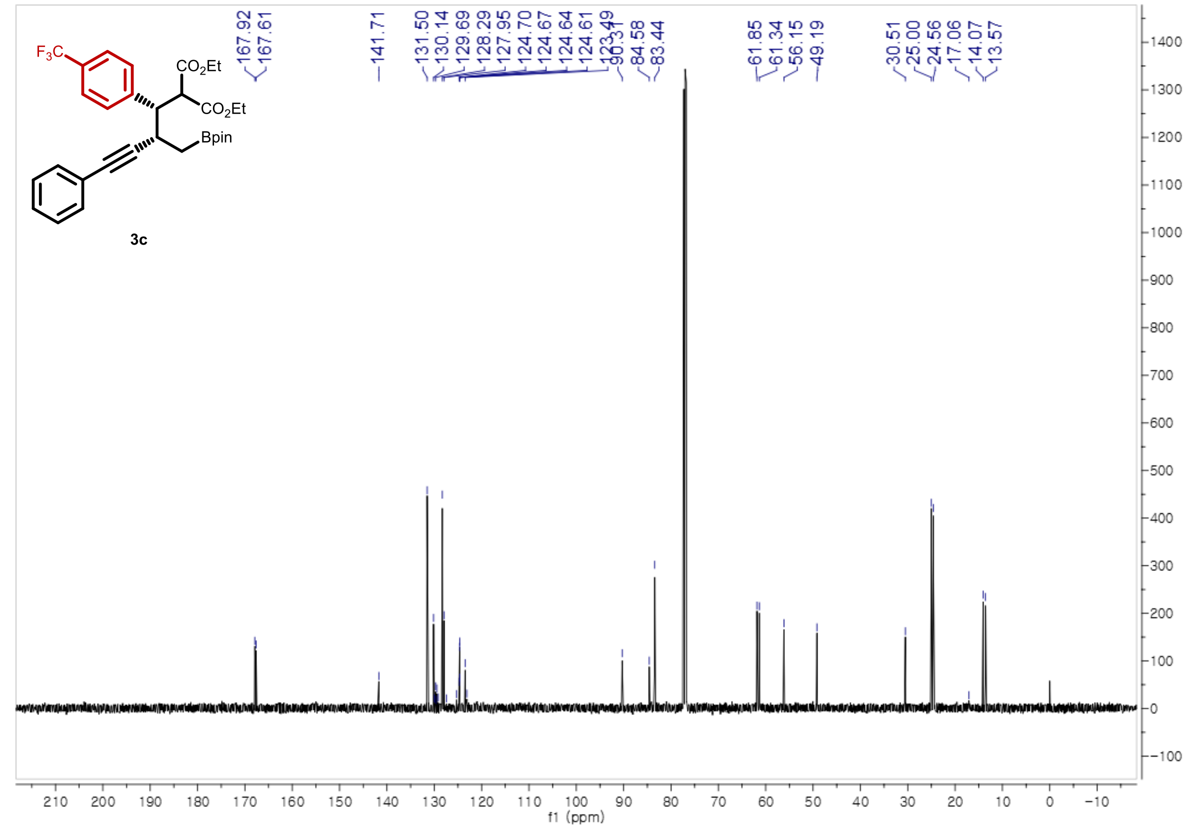

Supplementary Figure 8. ^{13}C NMR of compound **3c**.

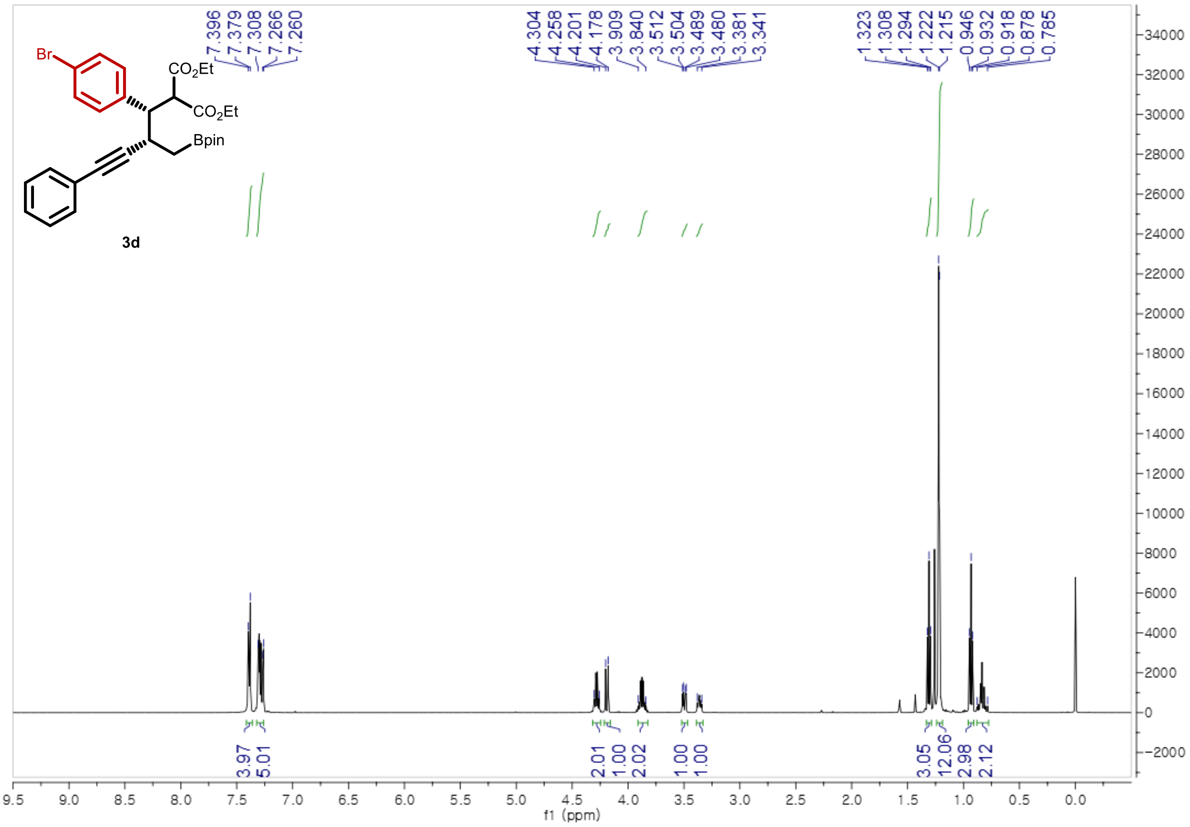

Supplementary Figure 9. ^{1}H NMR of compound **3d**.

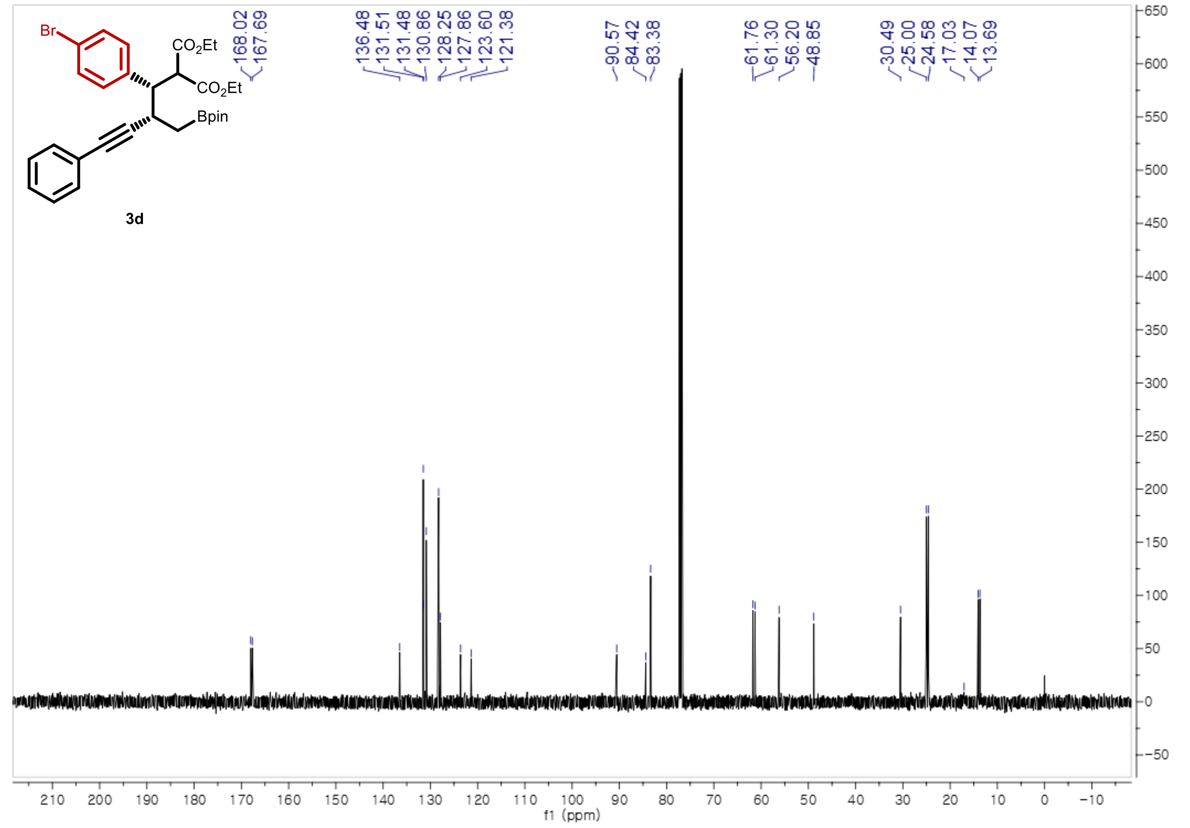

Supplementary Figure 10. ^{13}C NMR of compound **3d**.

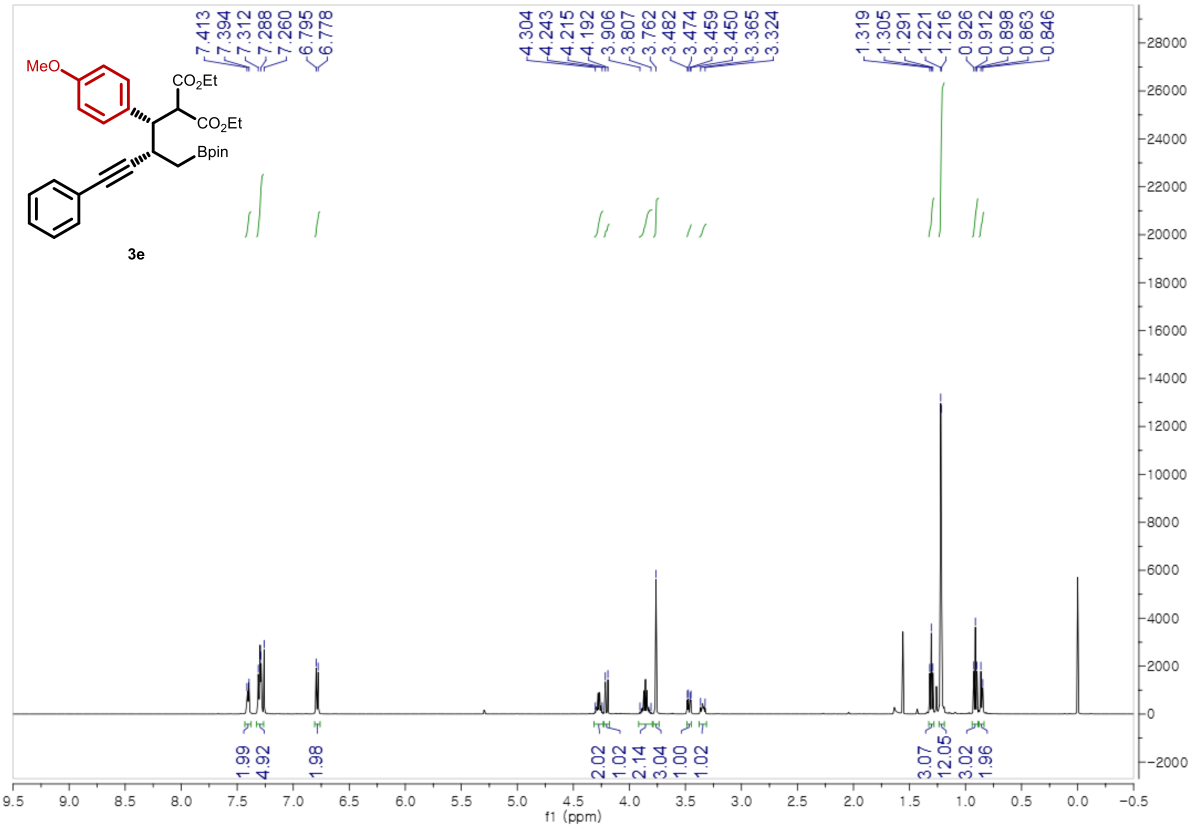

Supplementary Figure 11. ^{1}H NMR of compound **3e**.

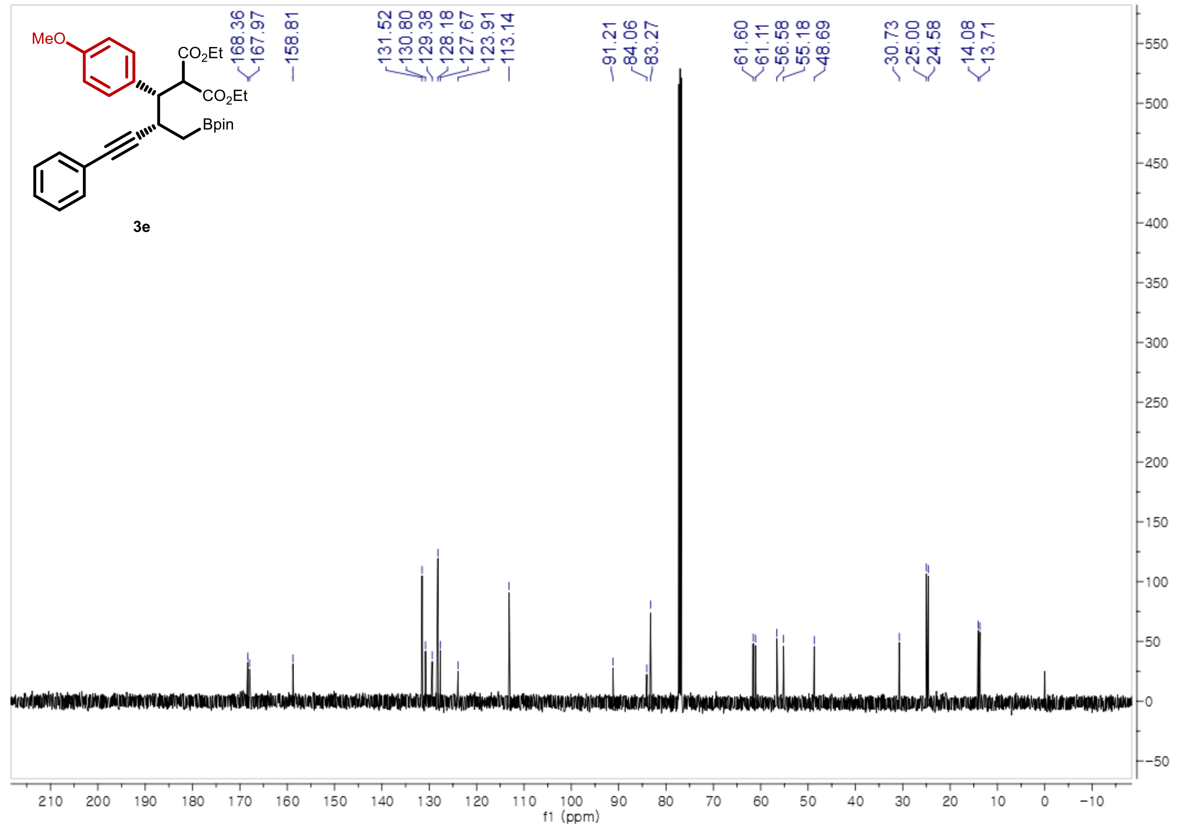

Supplementary Figure 12. ^{13}C NMR of compound **3e**.

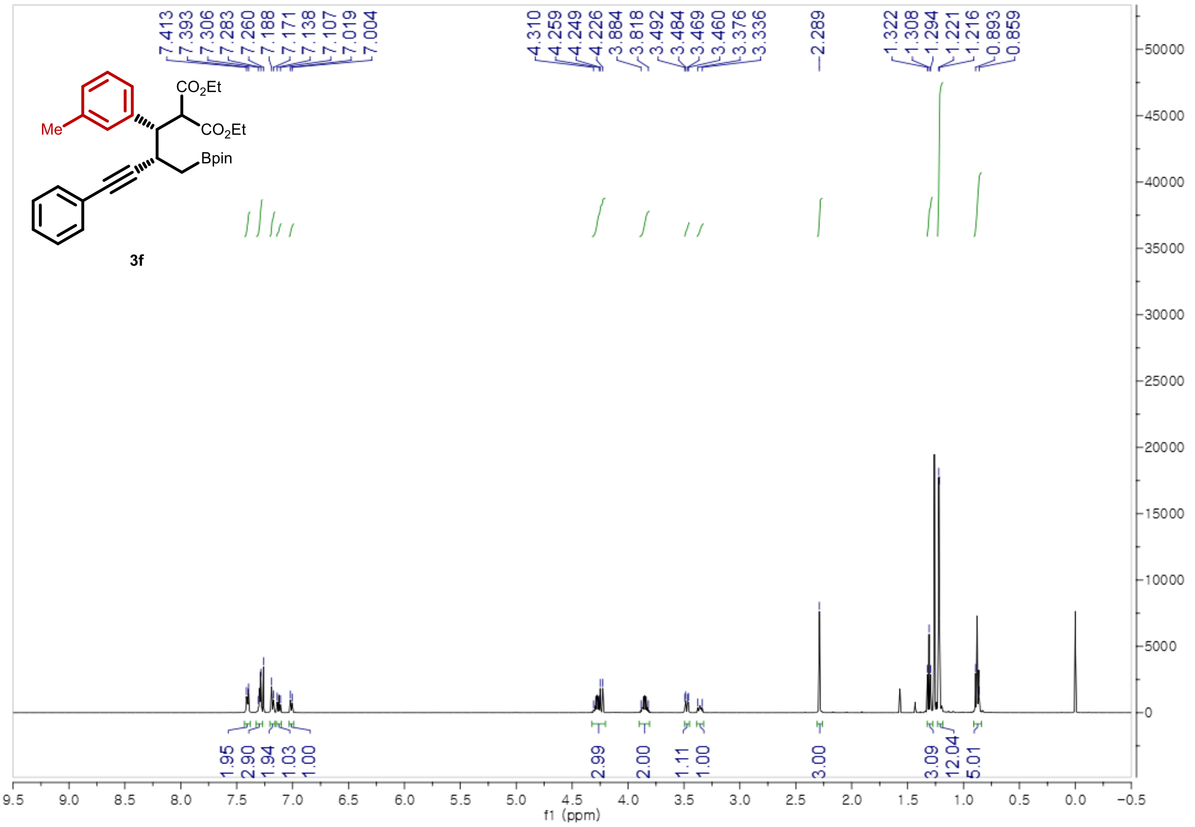

Supplementary Figure 13. ^{1}H NMR of compound **3f**.

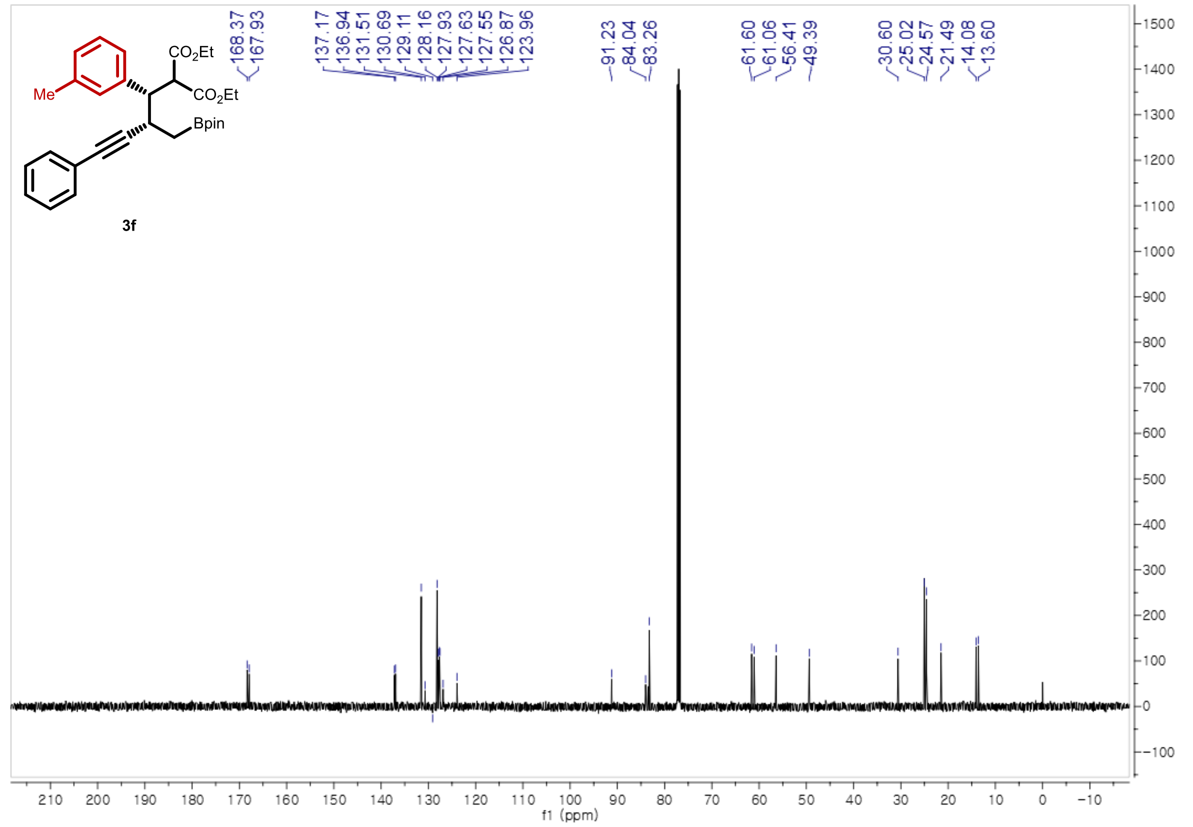

Supplementary Figure 14. ^{13}C NMR of compound **3f**.

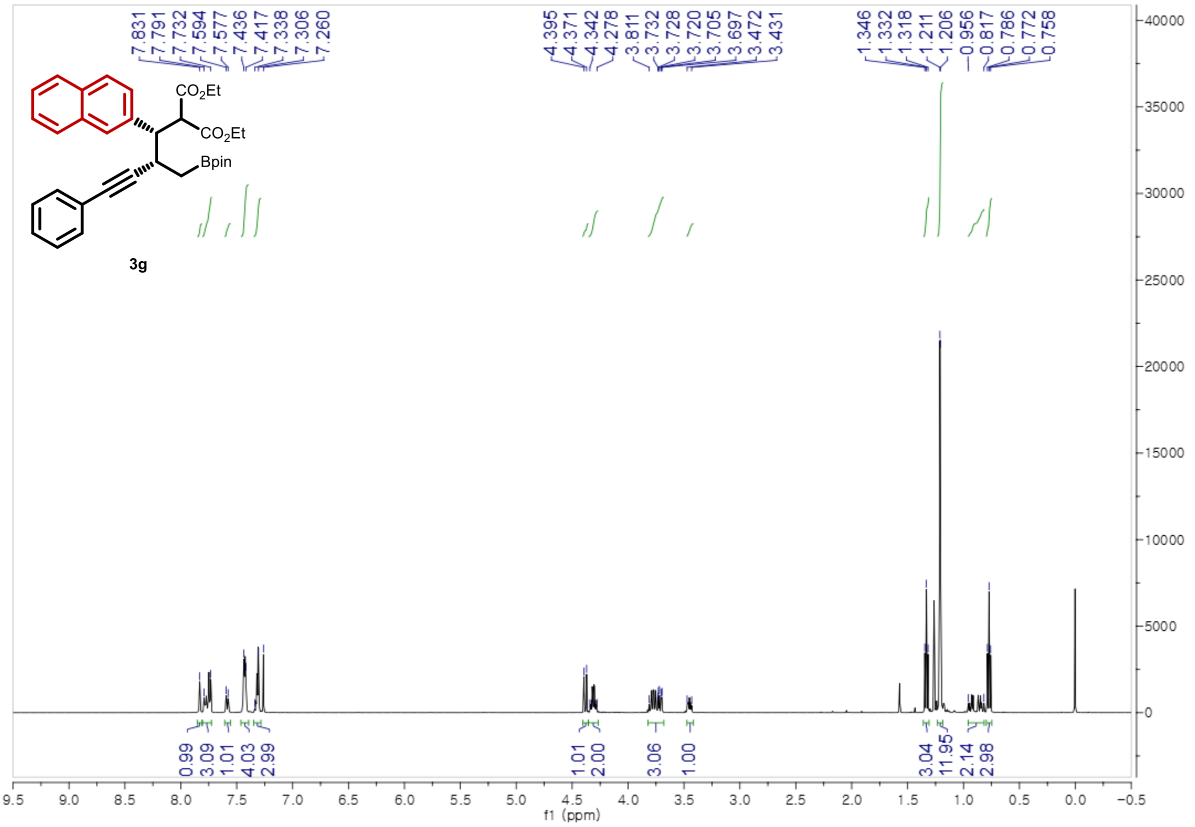

Supplementary Figure 15. ^{1}H NMR of compound **3g**.

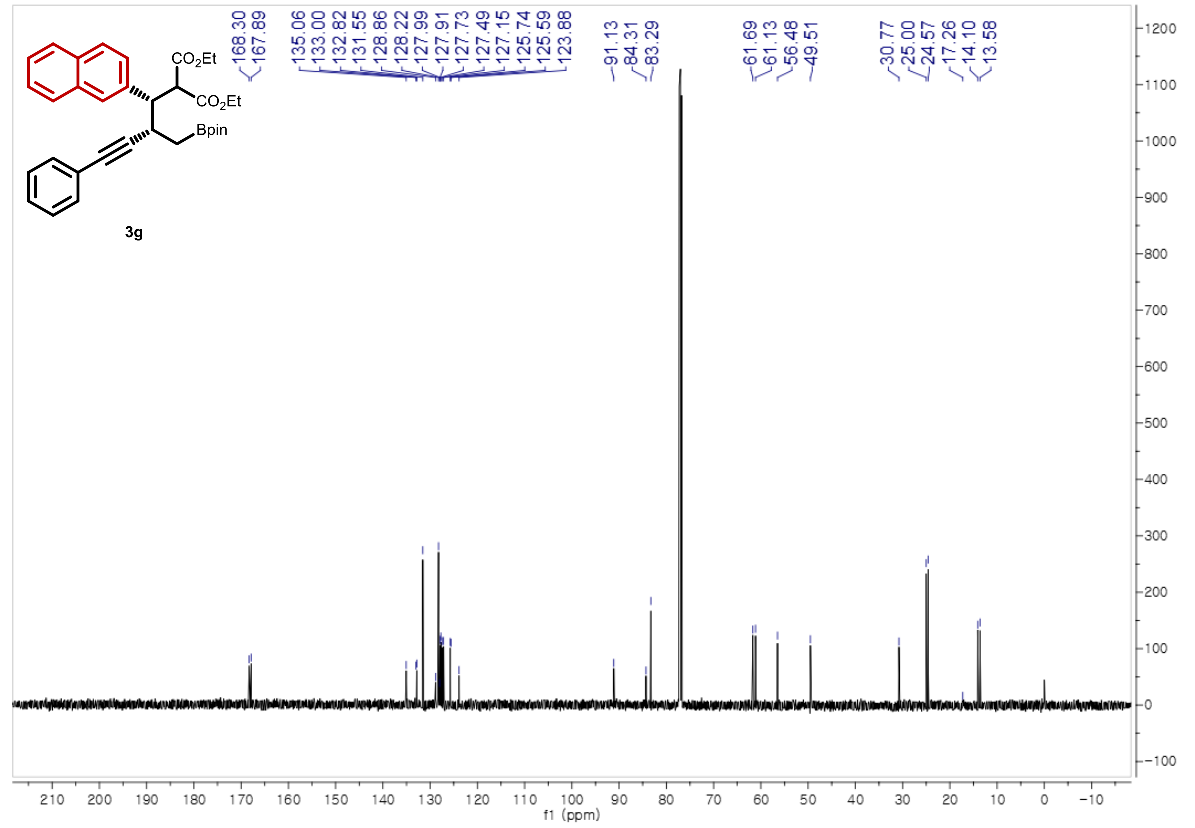

Supplementary Figure 16. ^{13}C NMR of compound **3g**.

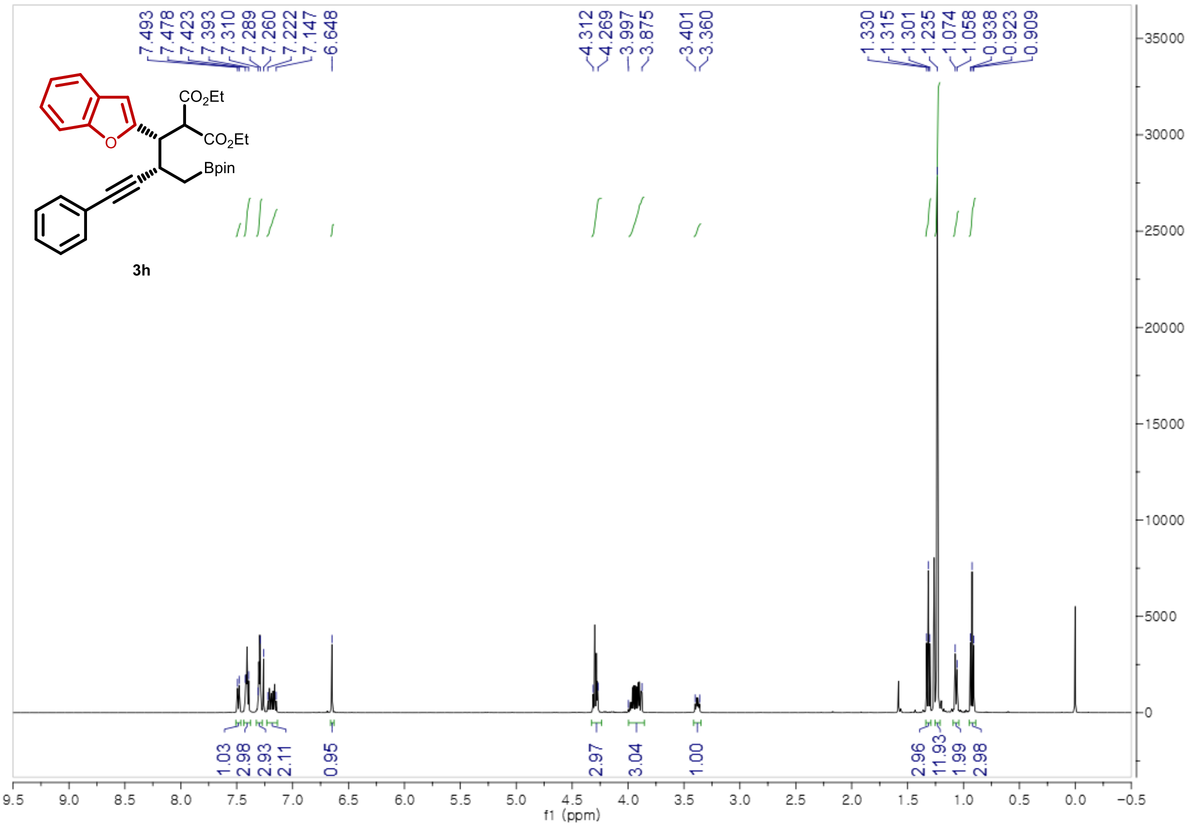

Supplementary Figure 17. ^{1}H NMR of compound **3h**.

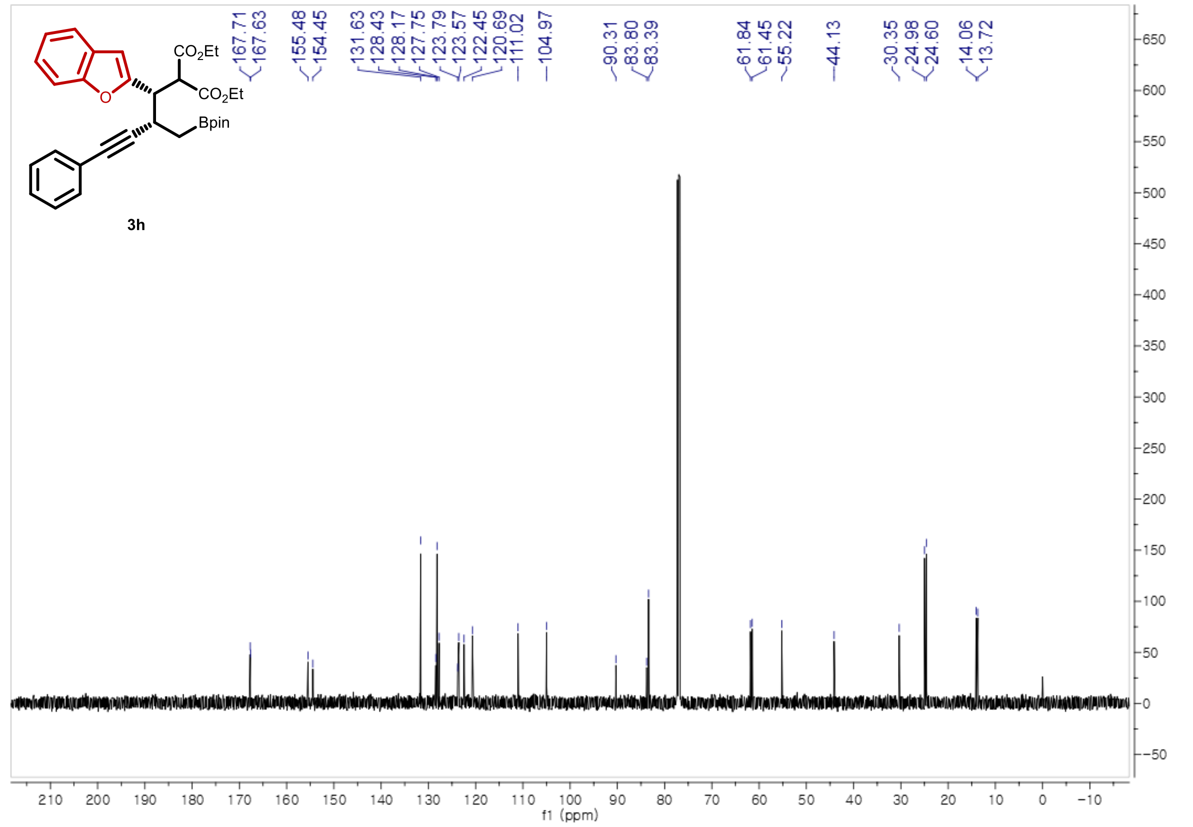

Supplementary Figure 18. ^{13}C NMR of compound **3h**.

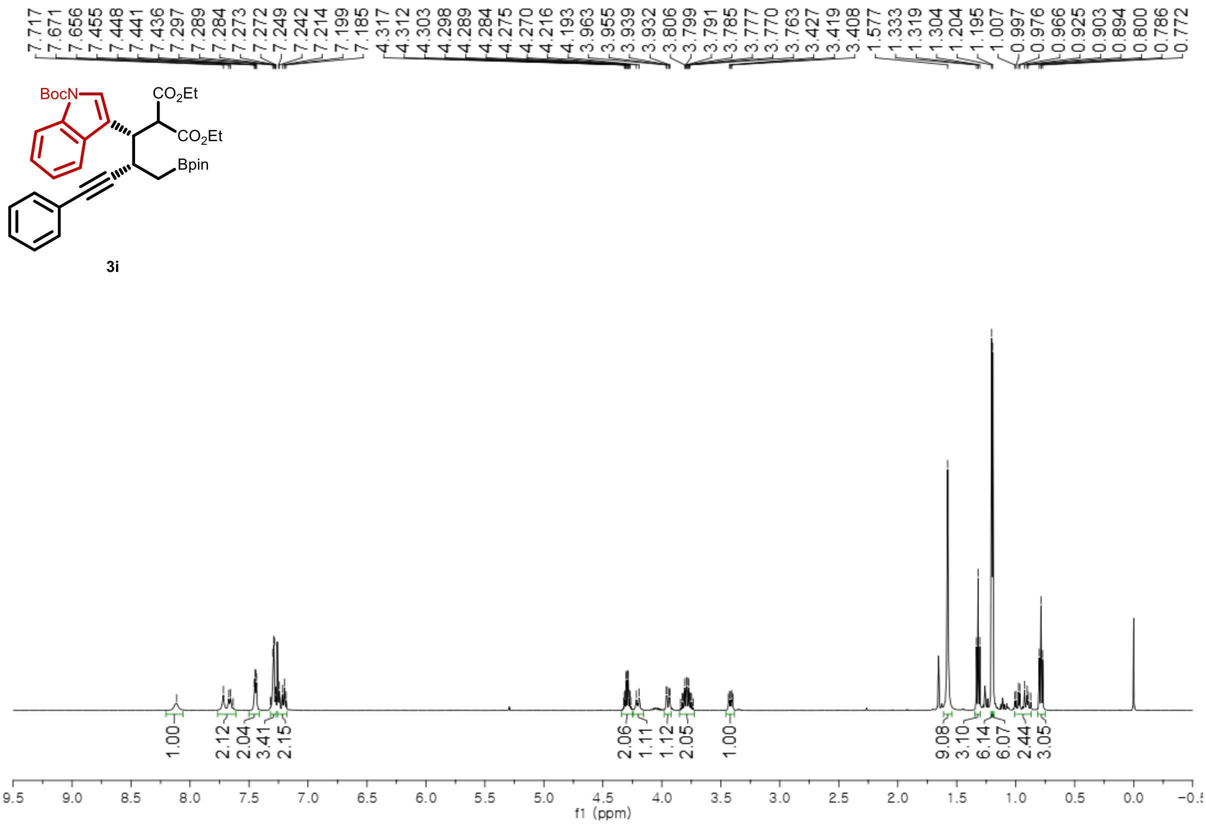

Supplementary Figure 19. ^{1}H NMR of compound **3i**.

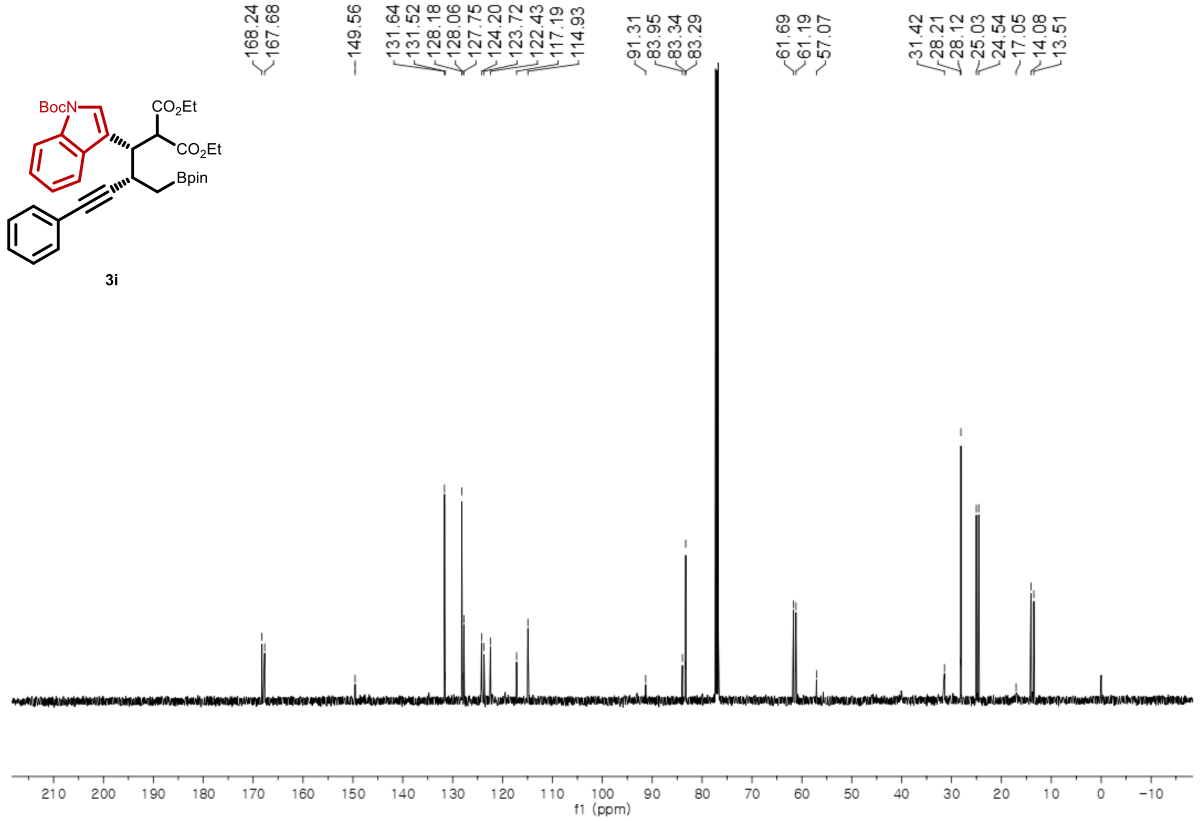

Supplementary Figure 20. ^{13}C NMR of compound **3i**.

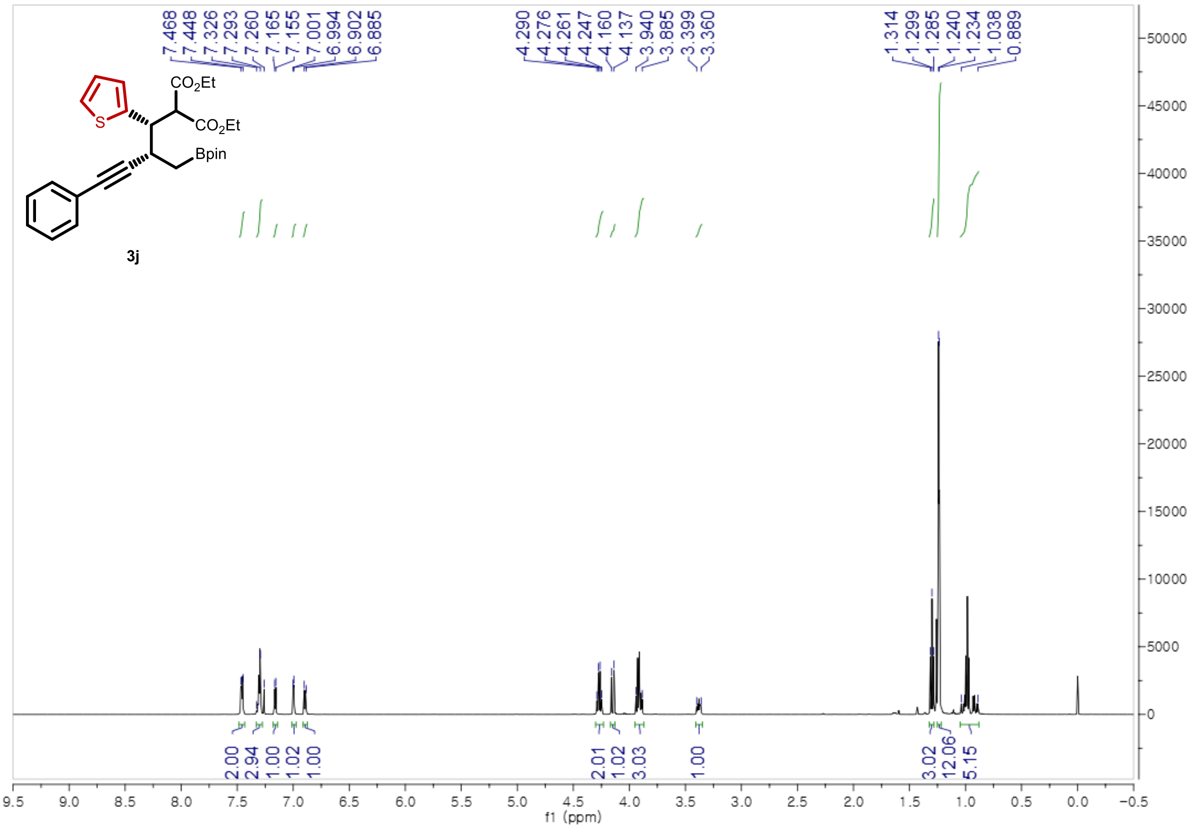

Supplementary Figure 21. ^{1}H NMR of compound **3j**.

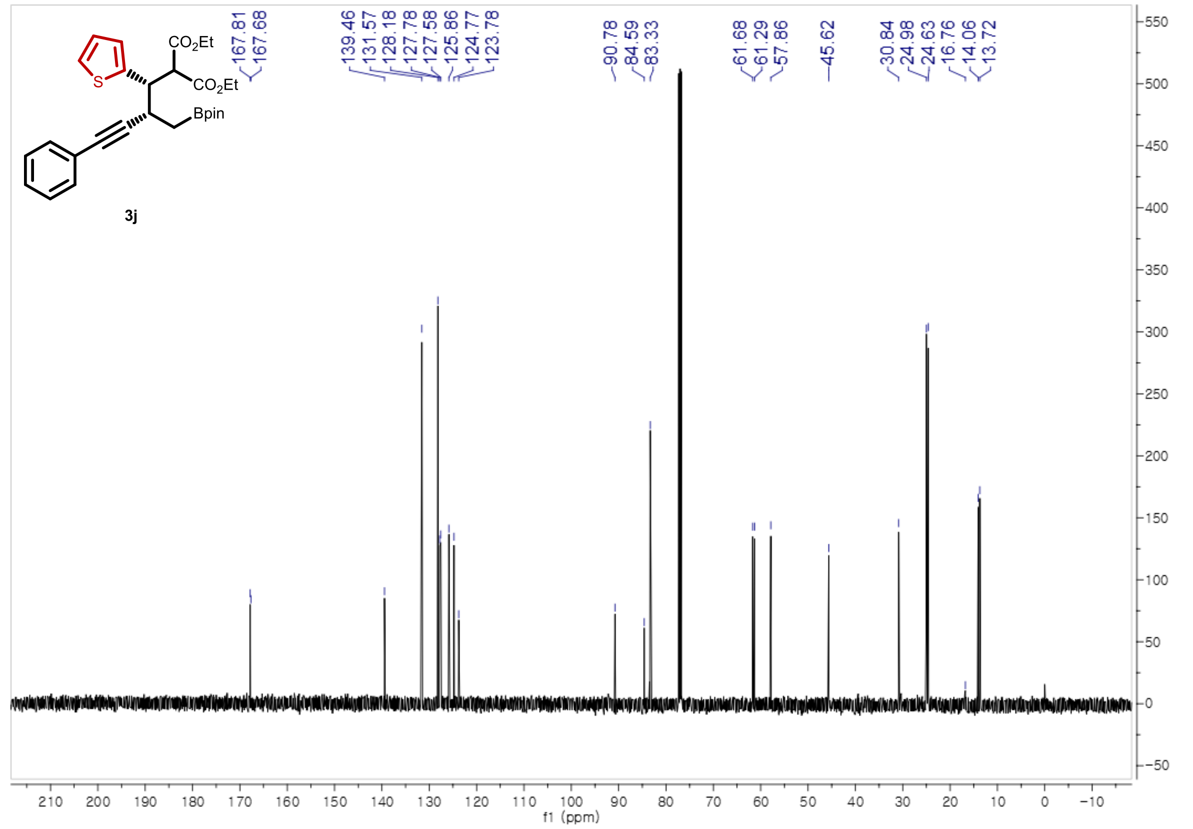

Supplementary Figure 22. ^{13}C NMR of compound **3j**.

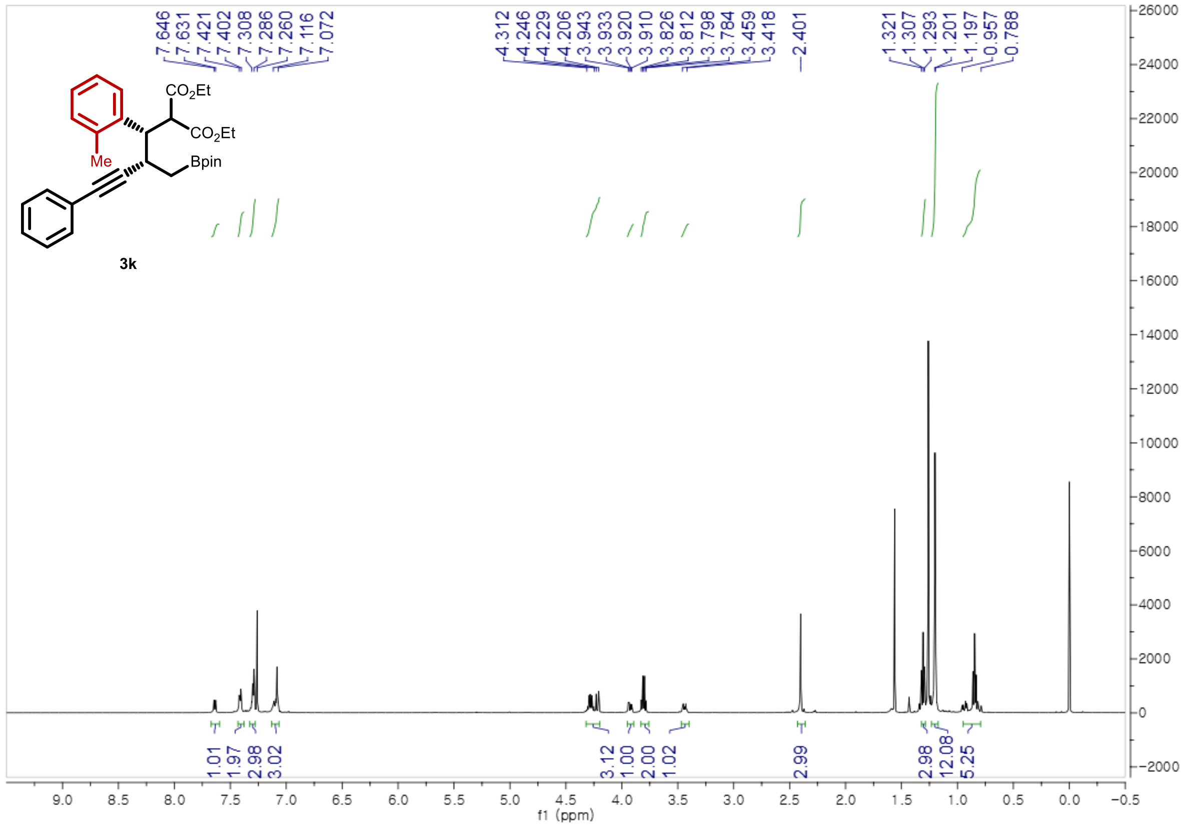

Supplementary Figure 23. ^{1}H NMR of compound **3k**.

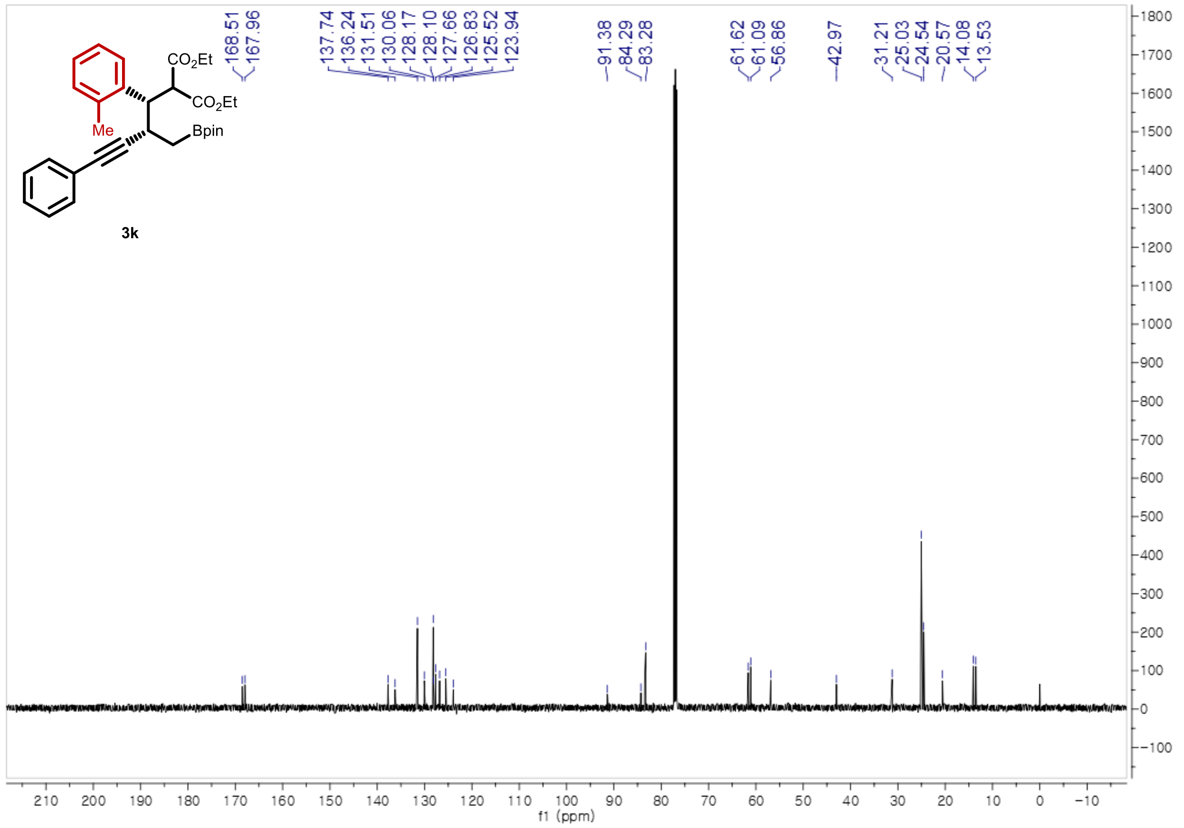

Supplementary Figure 24. ^{13}C NMR of compound **3k**.

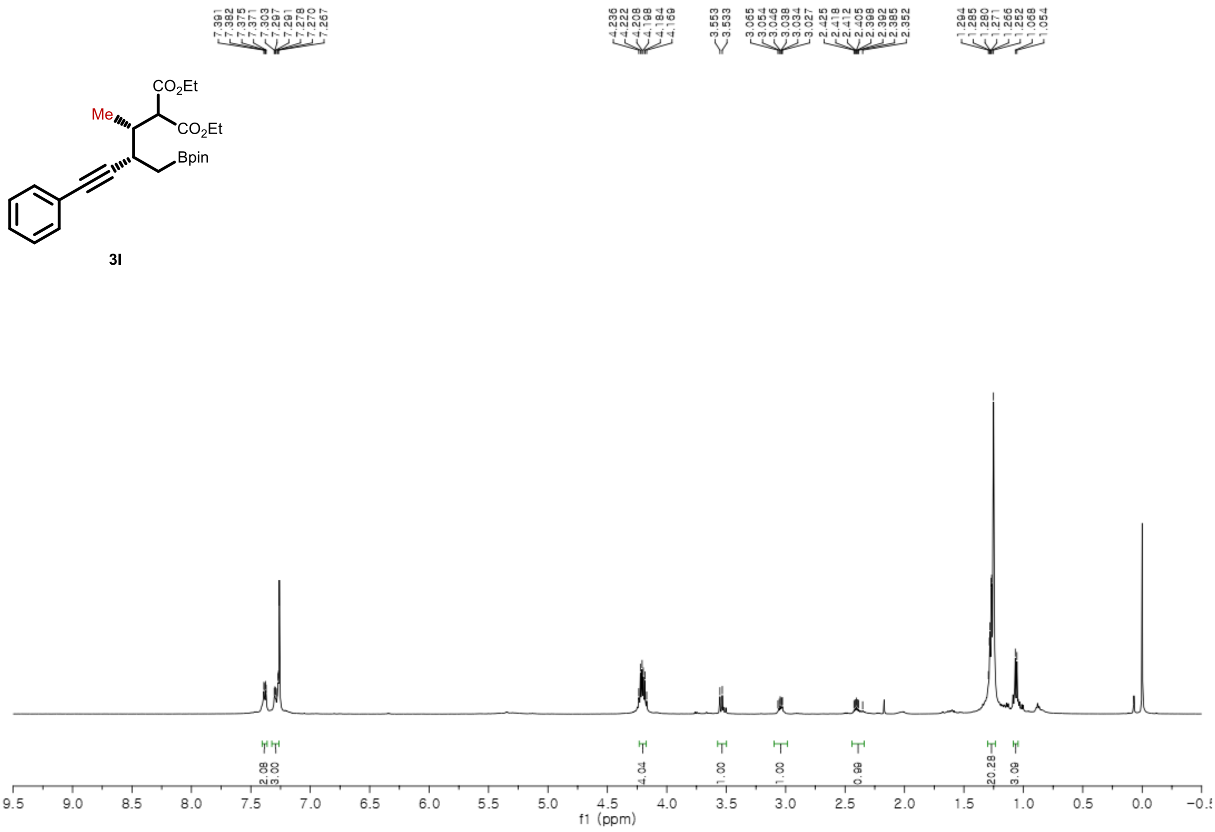

Supplementary Figure 25. ^{1}H NMR of compound **3l**.

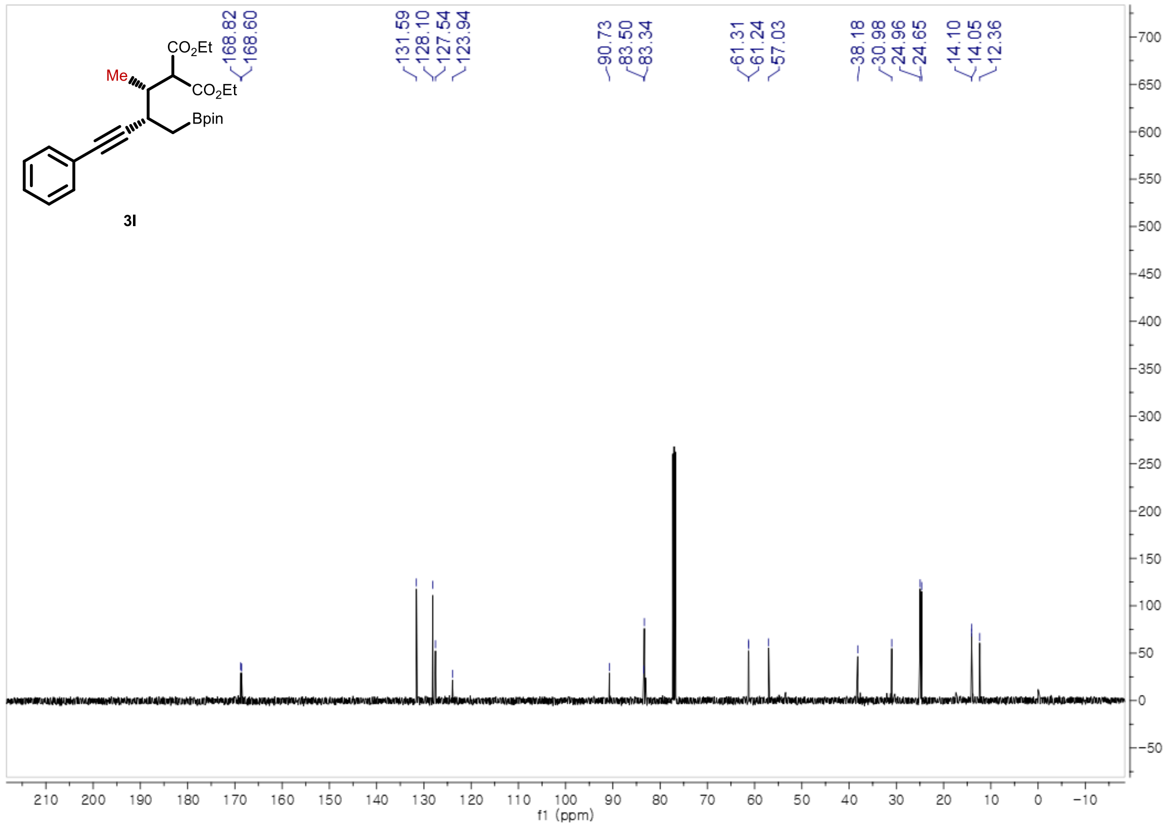

Supplementary Figure 26. ^{13}C NMR of compound **3l**.

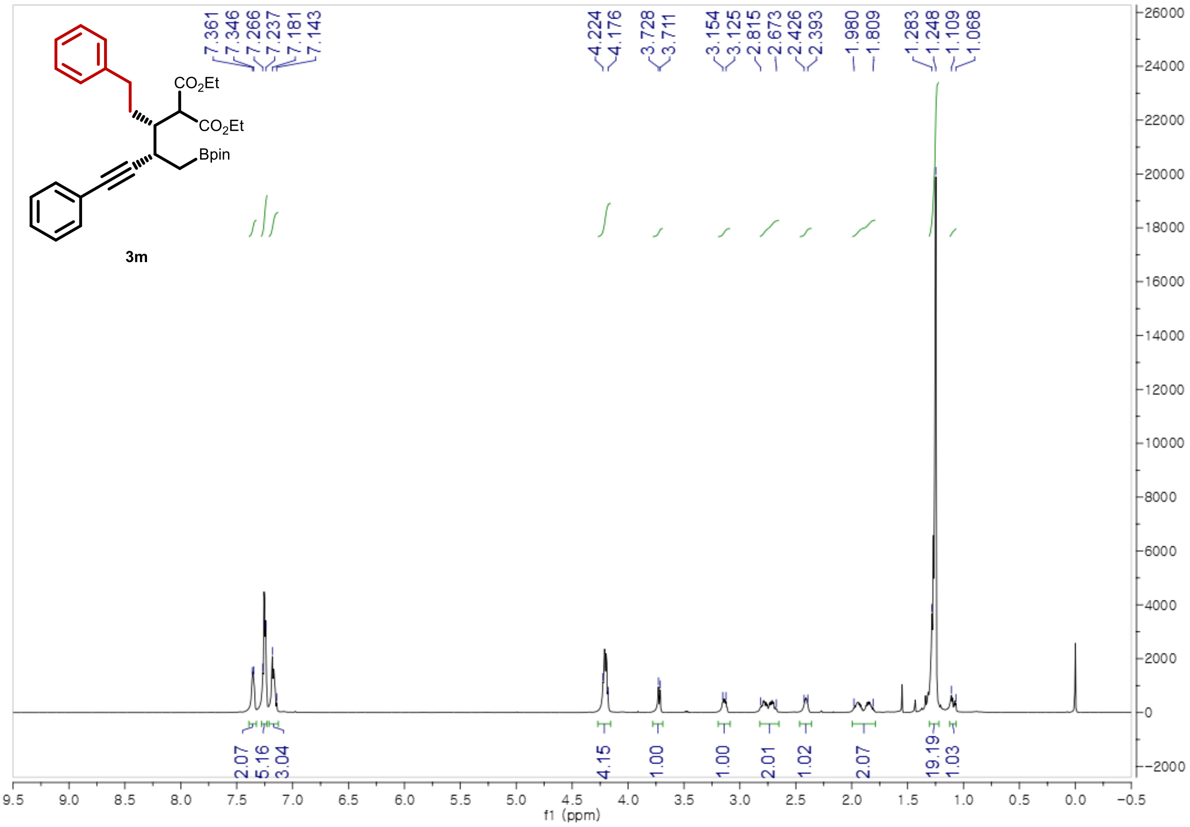

Supplementary Figure 27. ^{1}H NMR of compound **3m**.

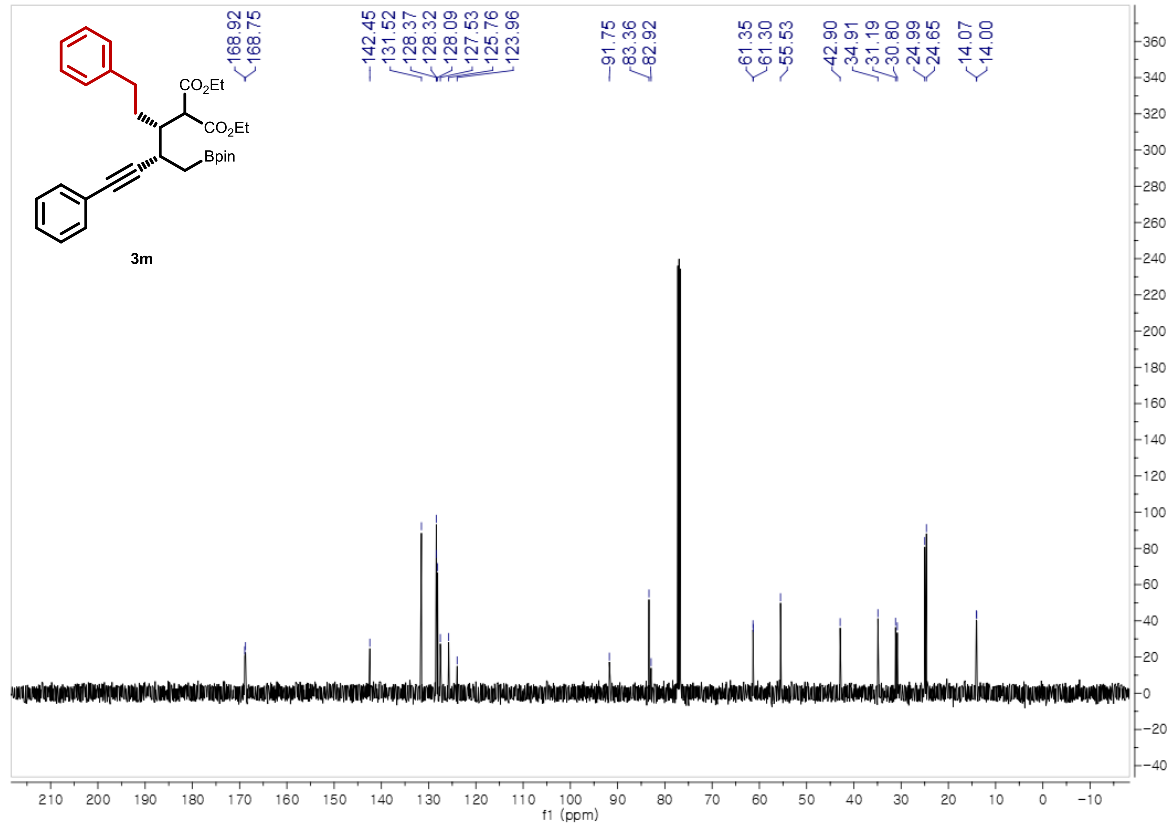

Supplementary Figure 28. ^{13}C NMR of compound **3m**.

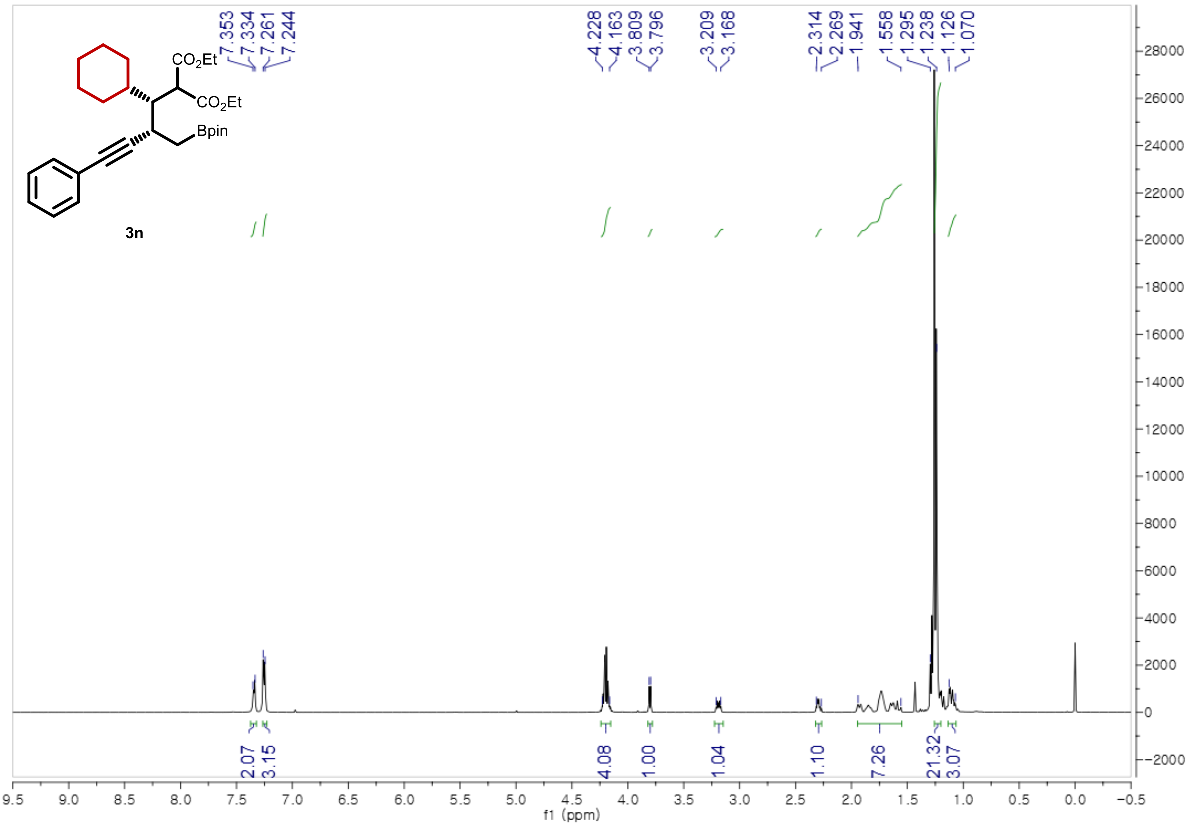

Supplementary Figure 29. ^{1}H NMR of compound **3n**.

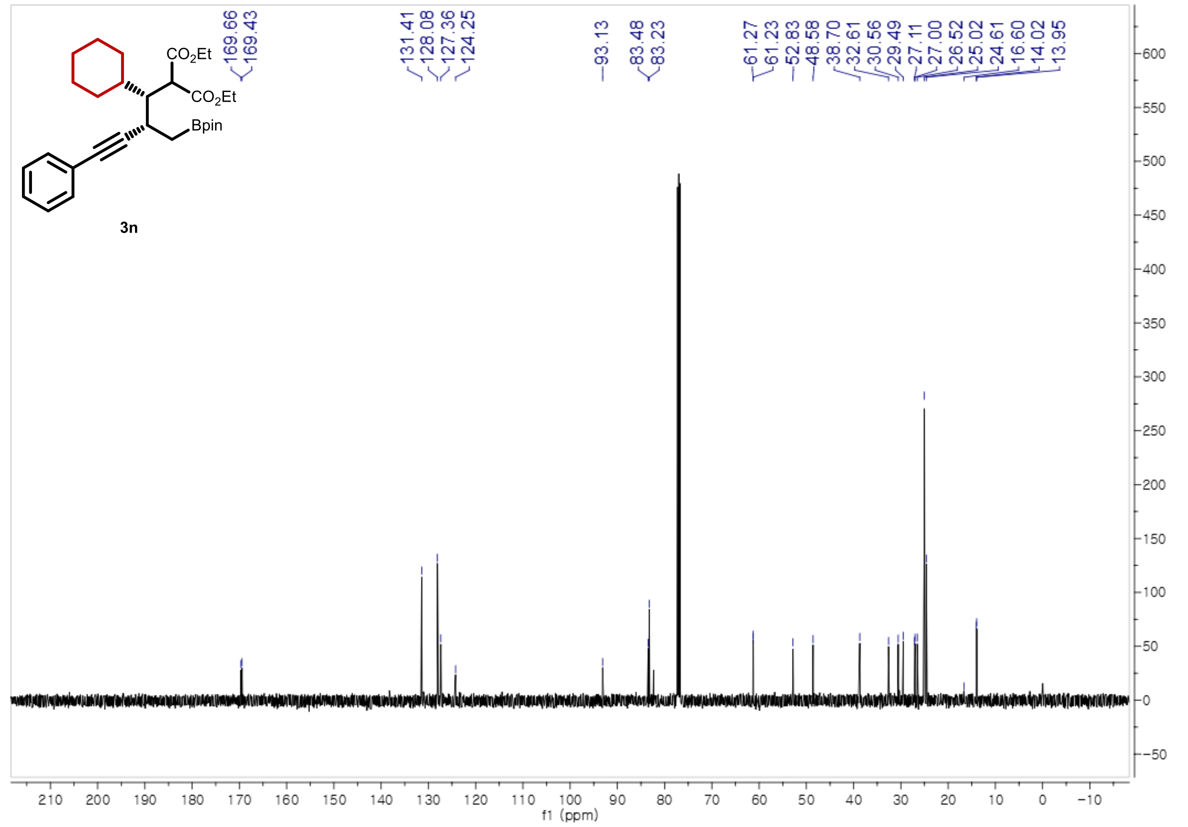

Supplementary Figure 30. ^{13}C NMR of compound **3n**.

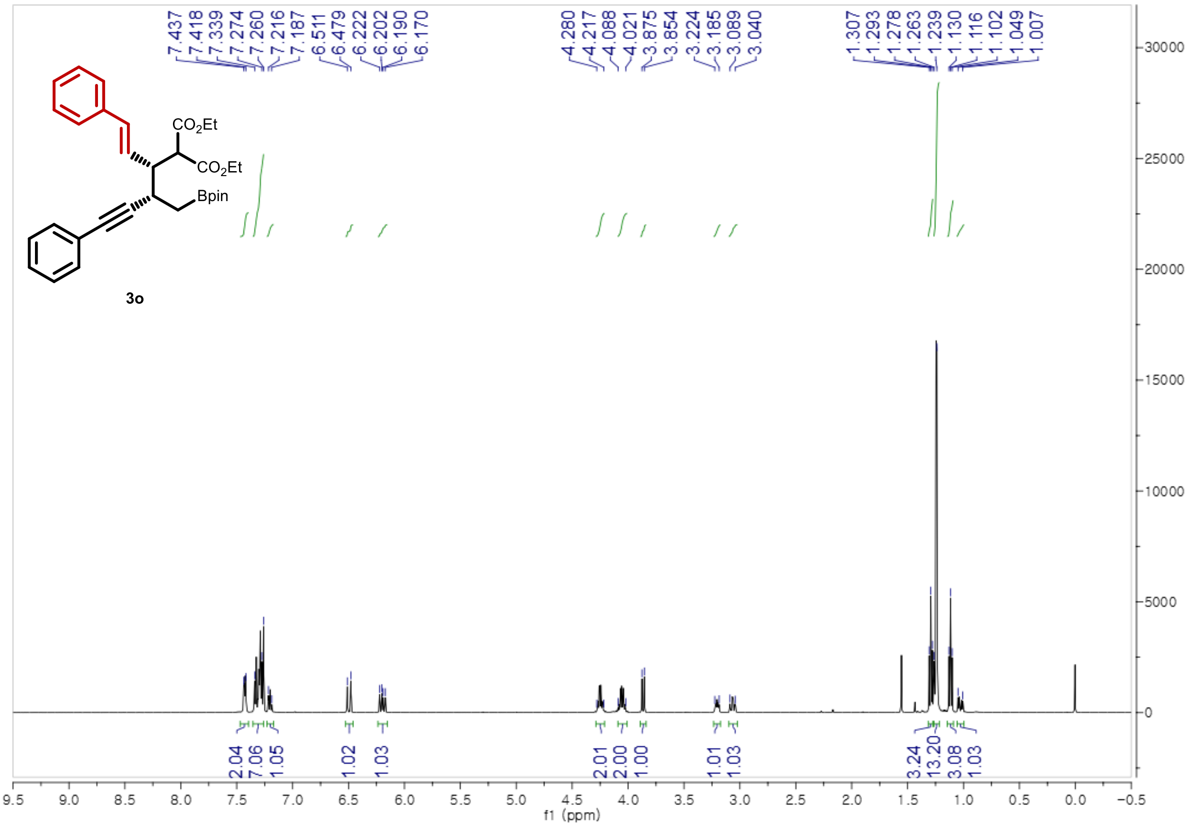

Supplementary Figure 31. ^{1}H NMR of compound **3o**.

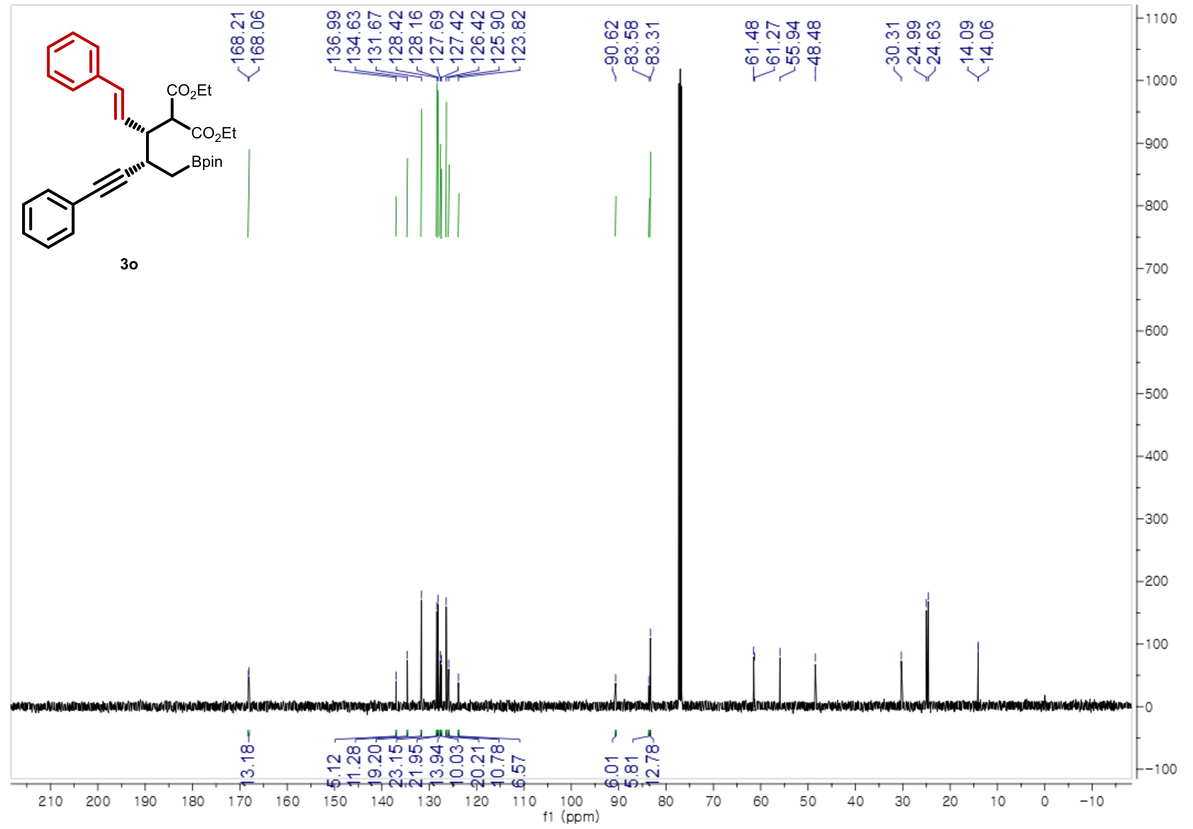

Supplementary Figure 32. ^{13}C NMR of compound **3o**.

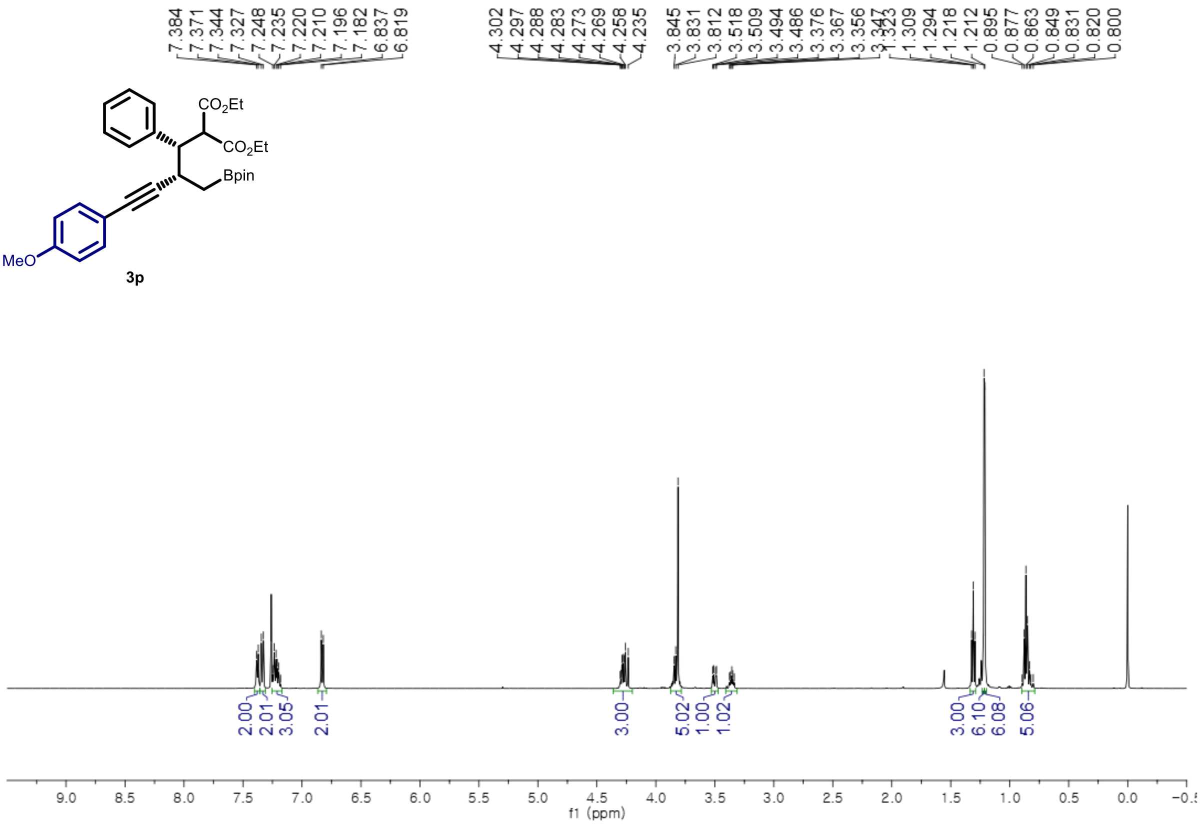

Supplementary Figure 33. ^{1}H NMR of compound **3p**.

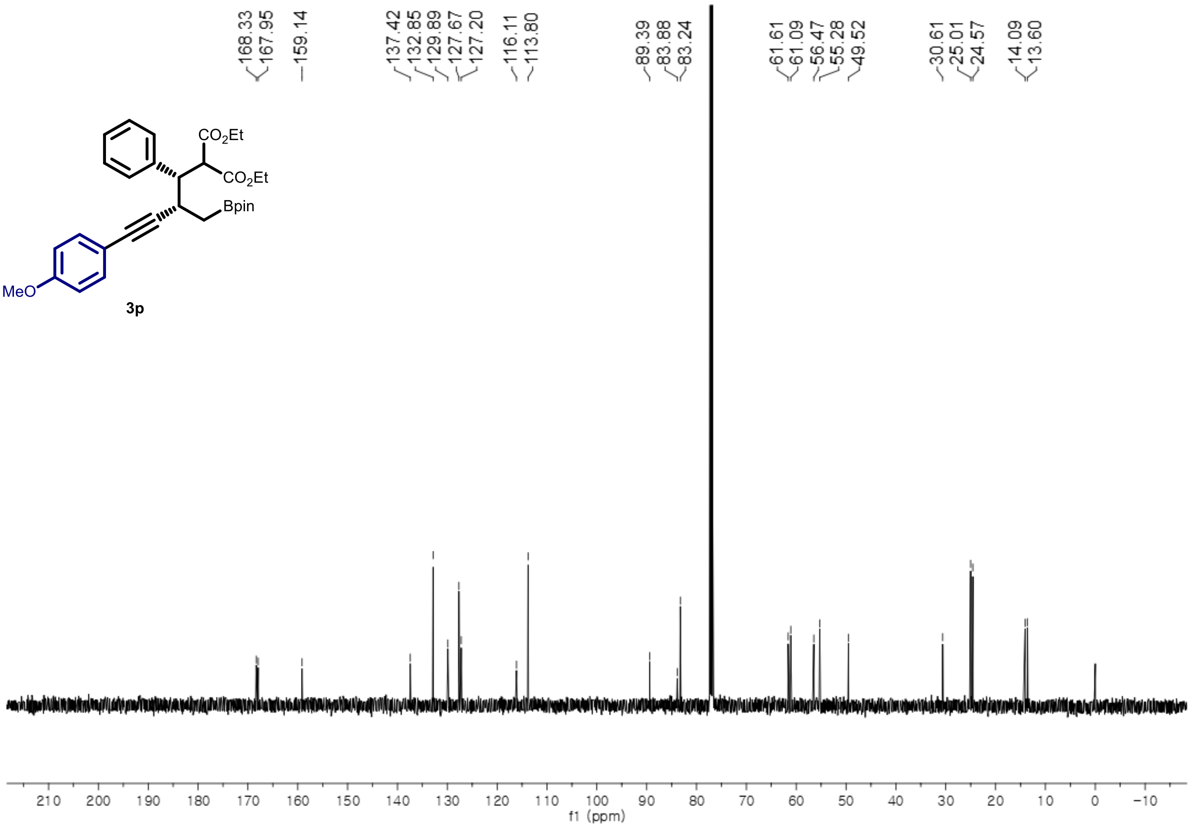

Supplementary Figure 34. ^{13}C NMR of compound **3p**.

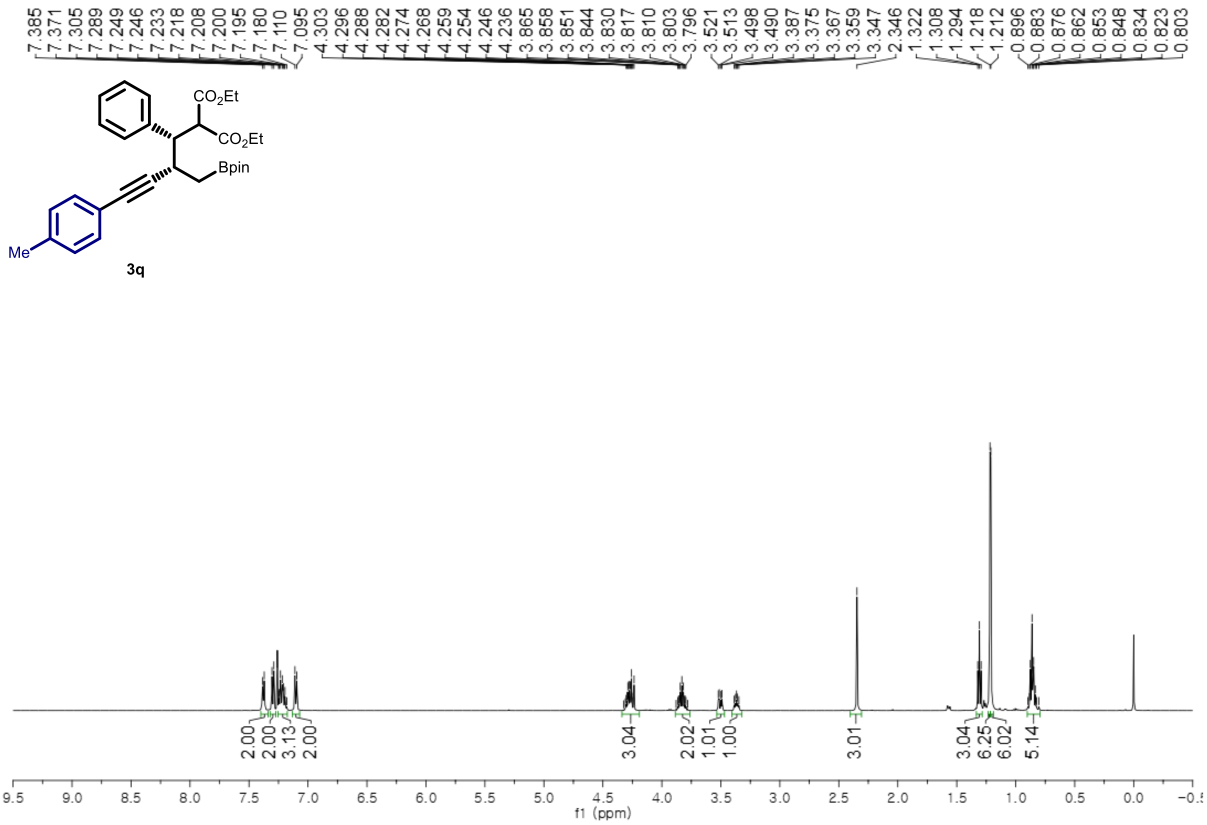

Supplementary Figure 35. ^{1}H NMR of compound **3q**.

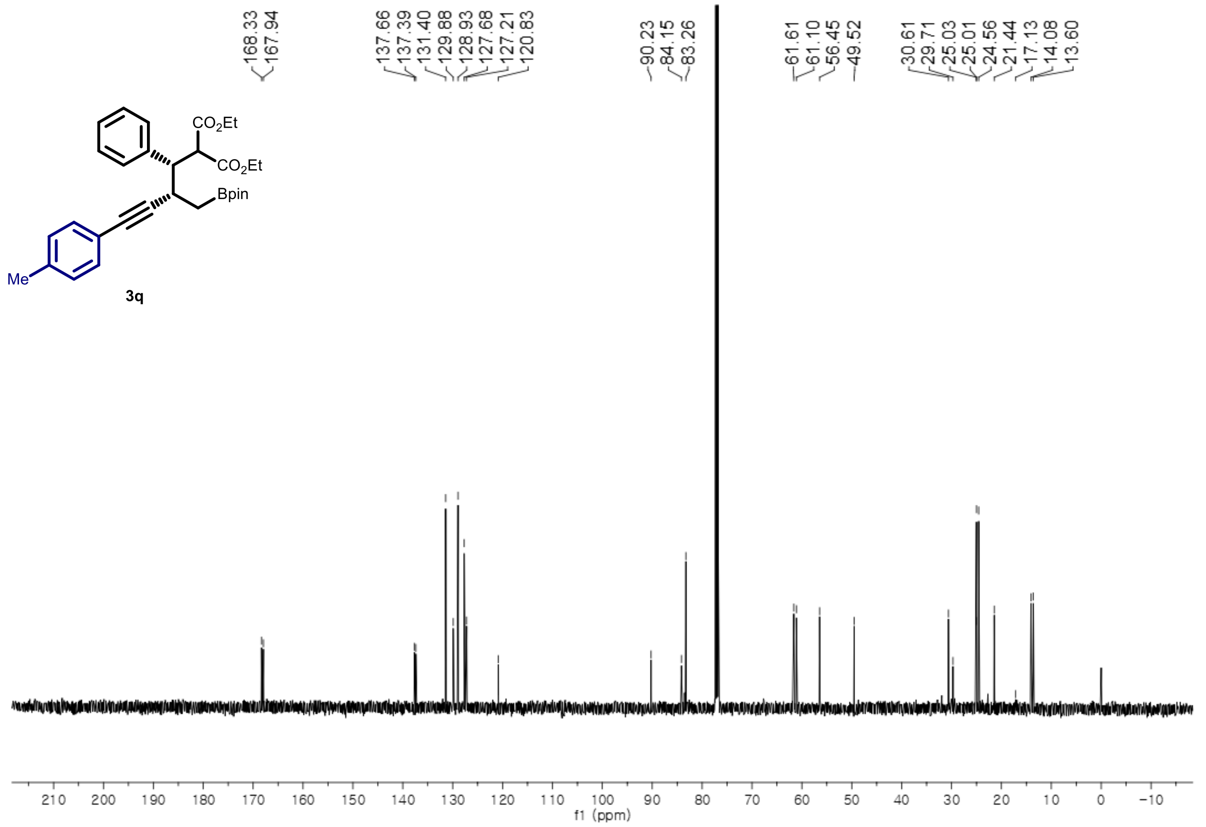

Supplementary Figure 36. ^{13}C NMR of compound **3q**.

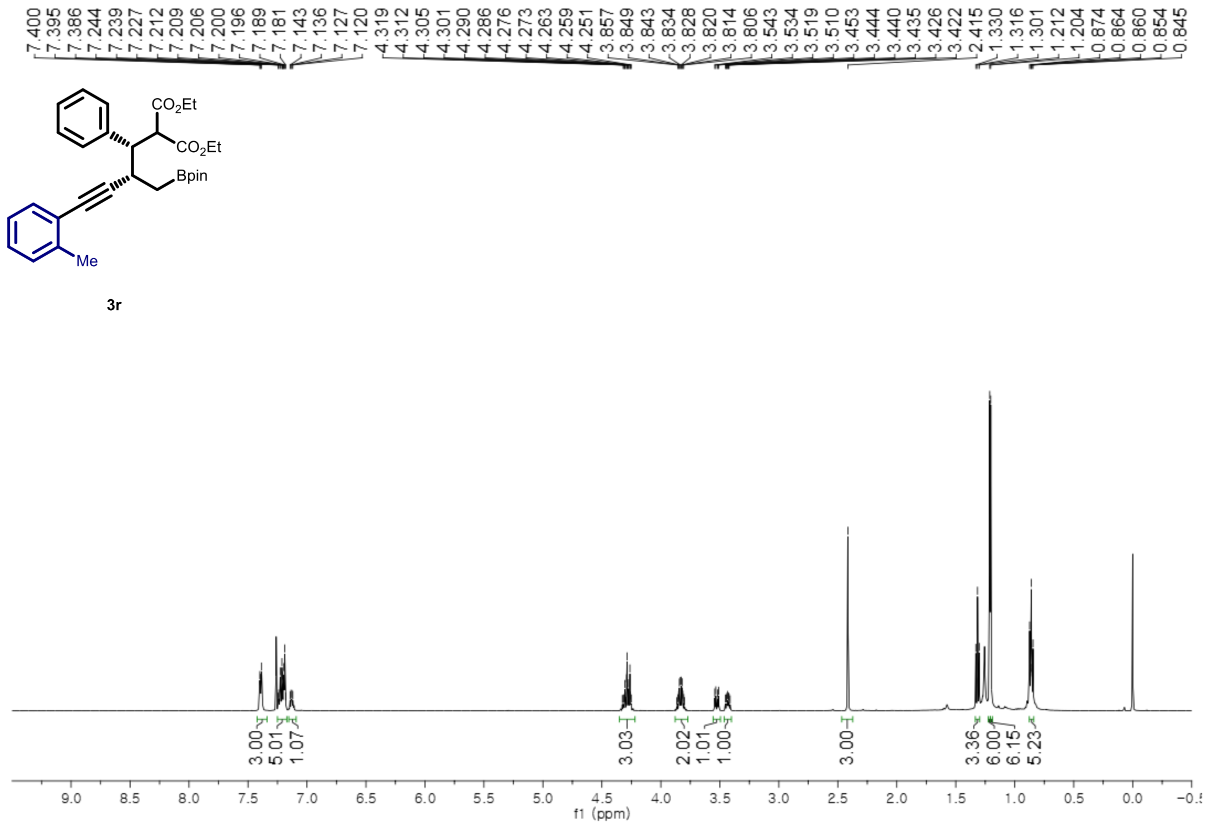

Supplementary Figure 37. ^{1}H NMR of compound **3r**.

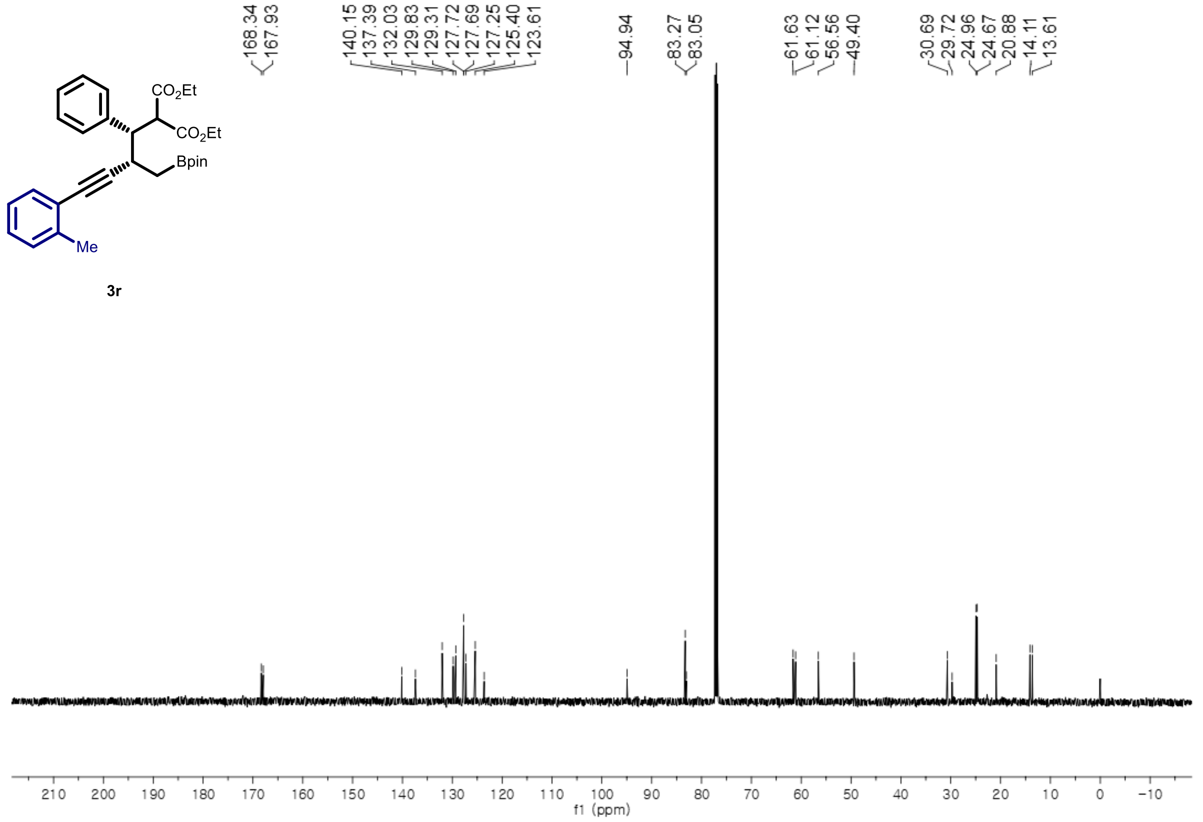

Supplementary Figure 38. ^{13}C NMR of compound **3r**.

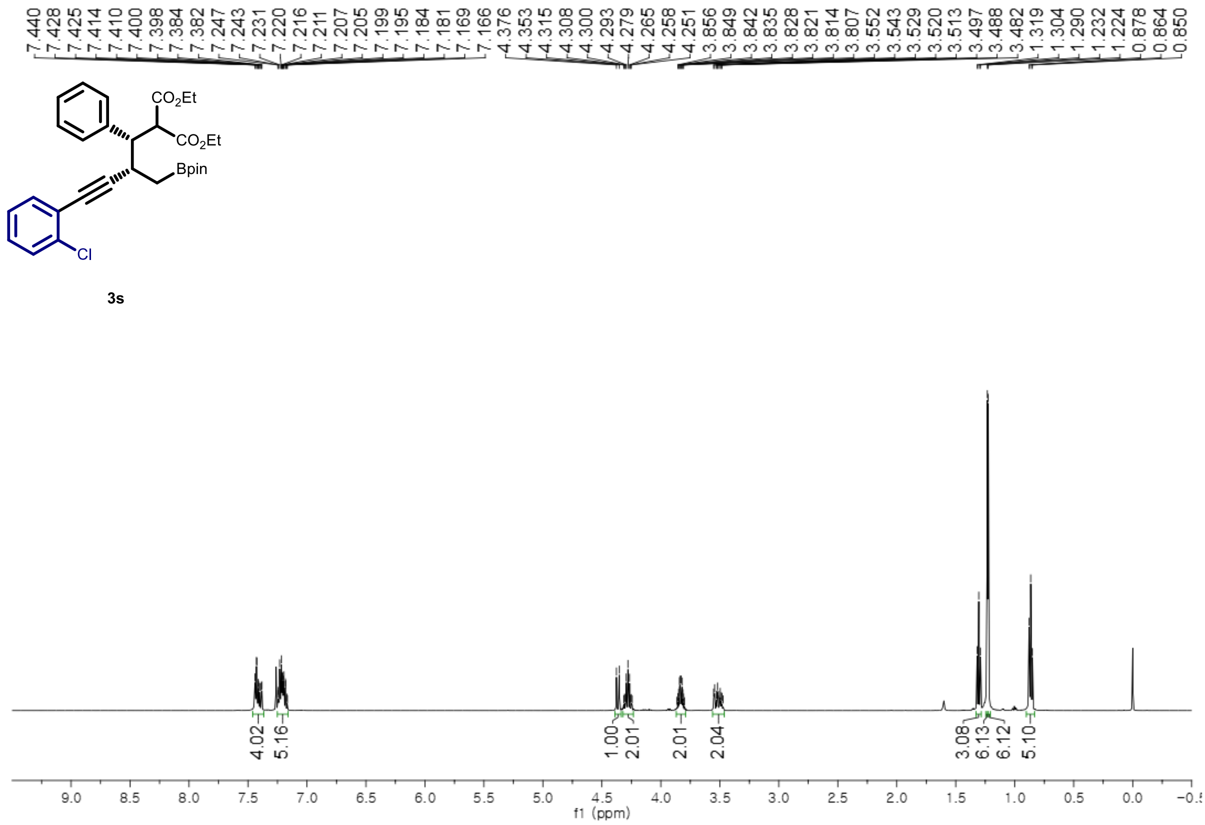

Supplementary Figure 39. ^{1}H NMR of compound **3s**.

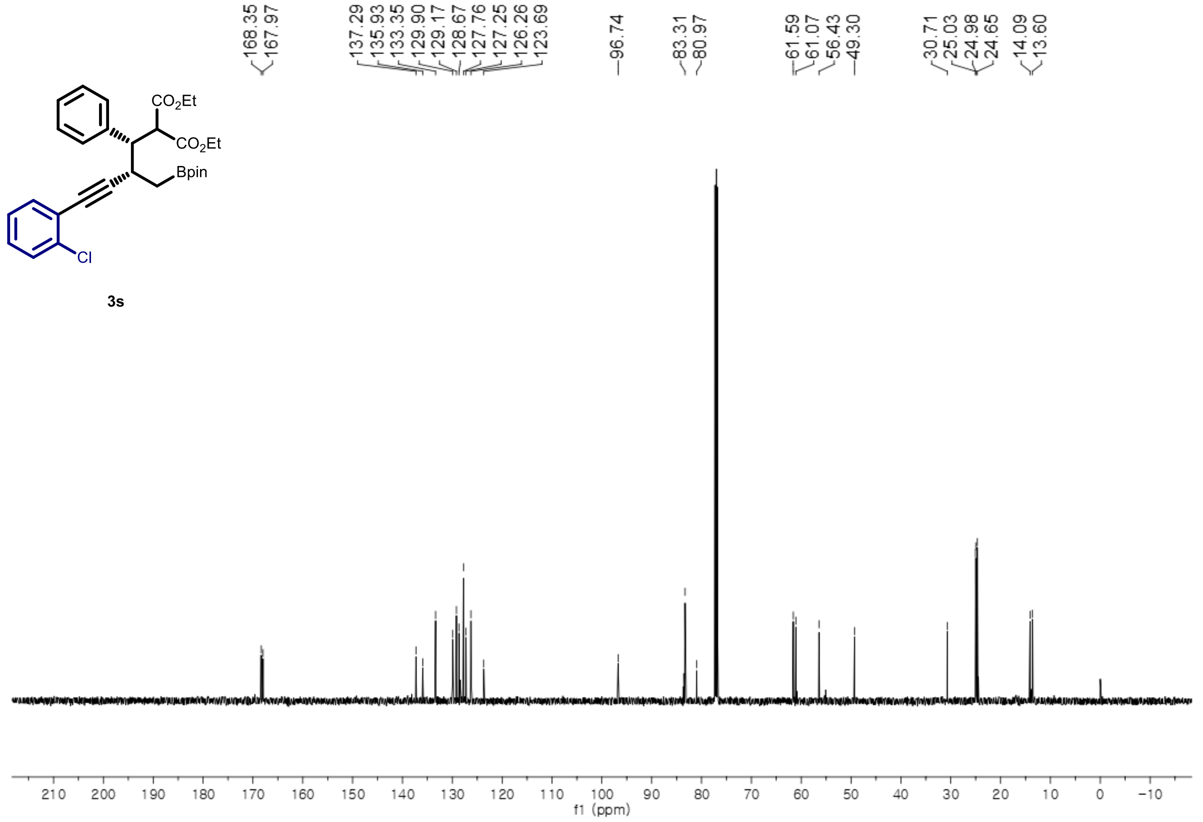

Supplementary Figure 40. ^{13}C NMR of compound **3s**.

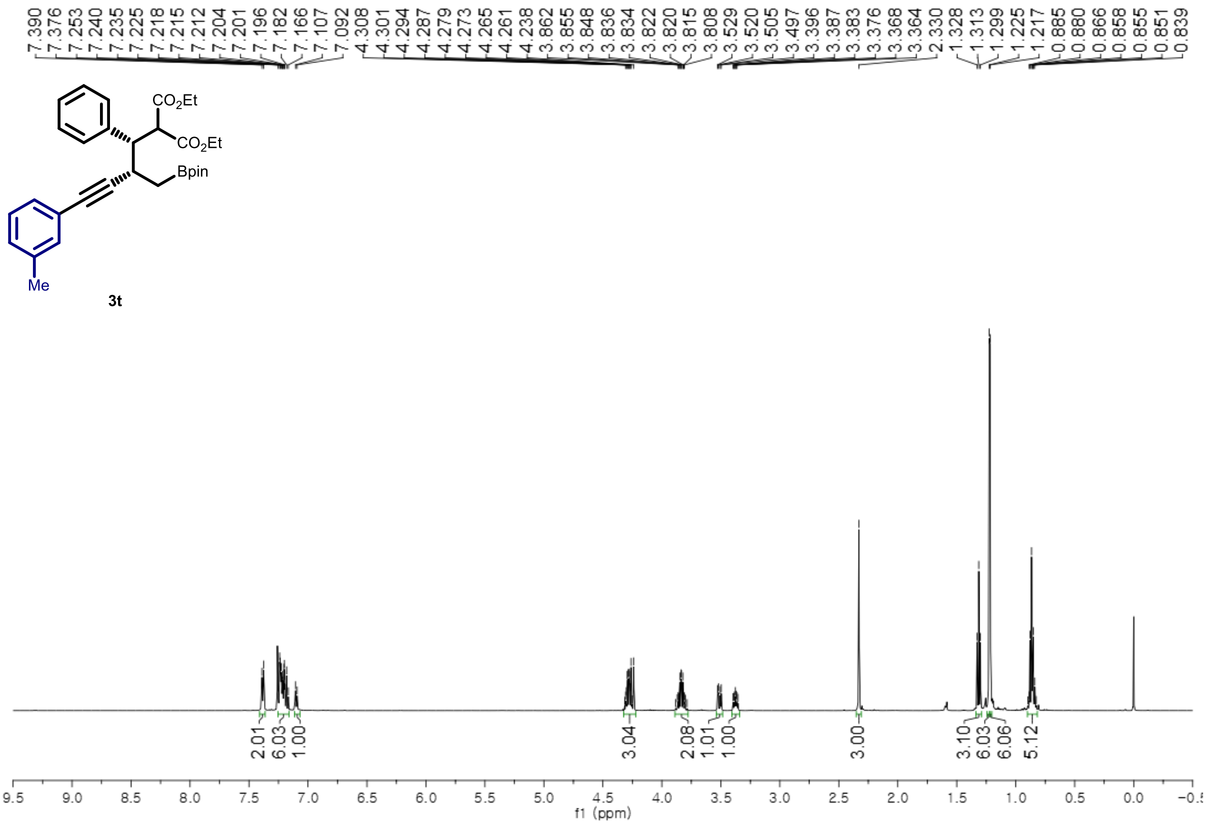

Supplementary Figure 41. ^{1}H NMR of compound **3t**.

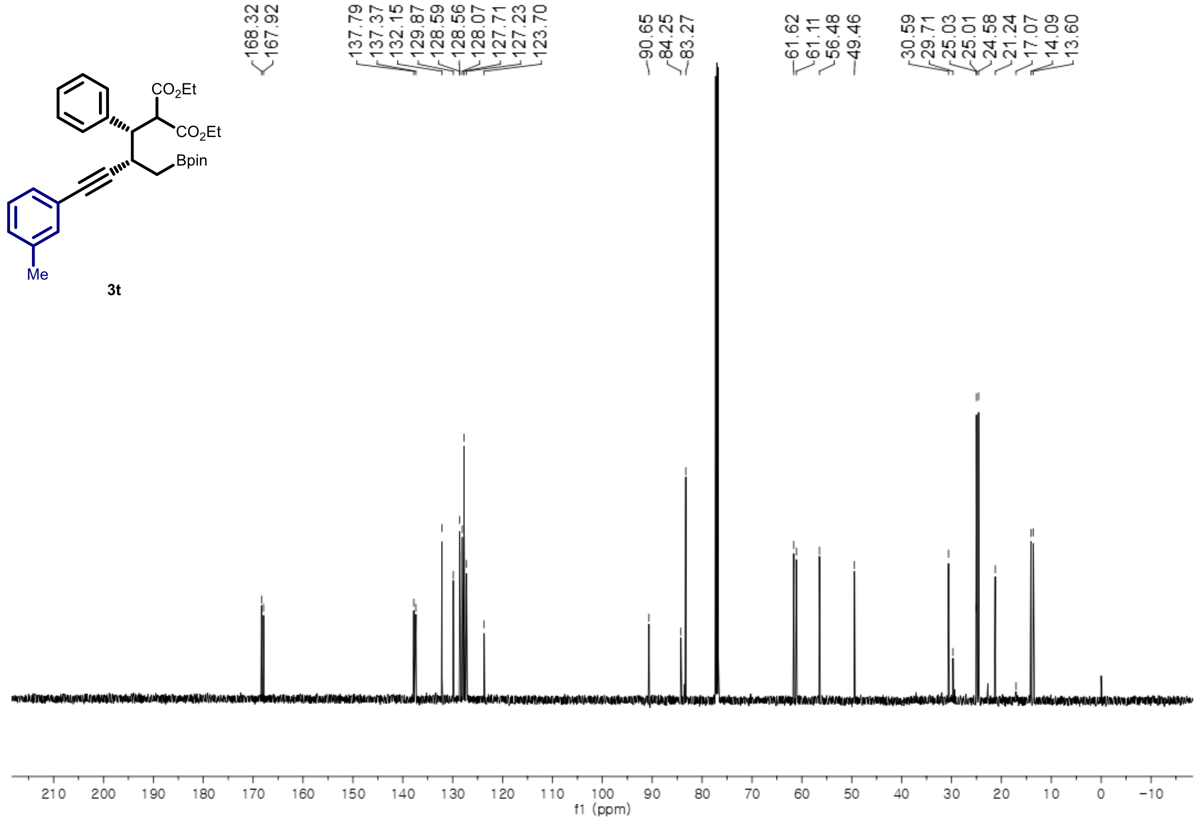

Supplementary Figure 42. ^{13}C NMR of compound **3t**.

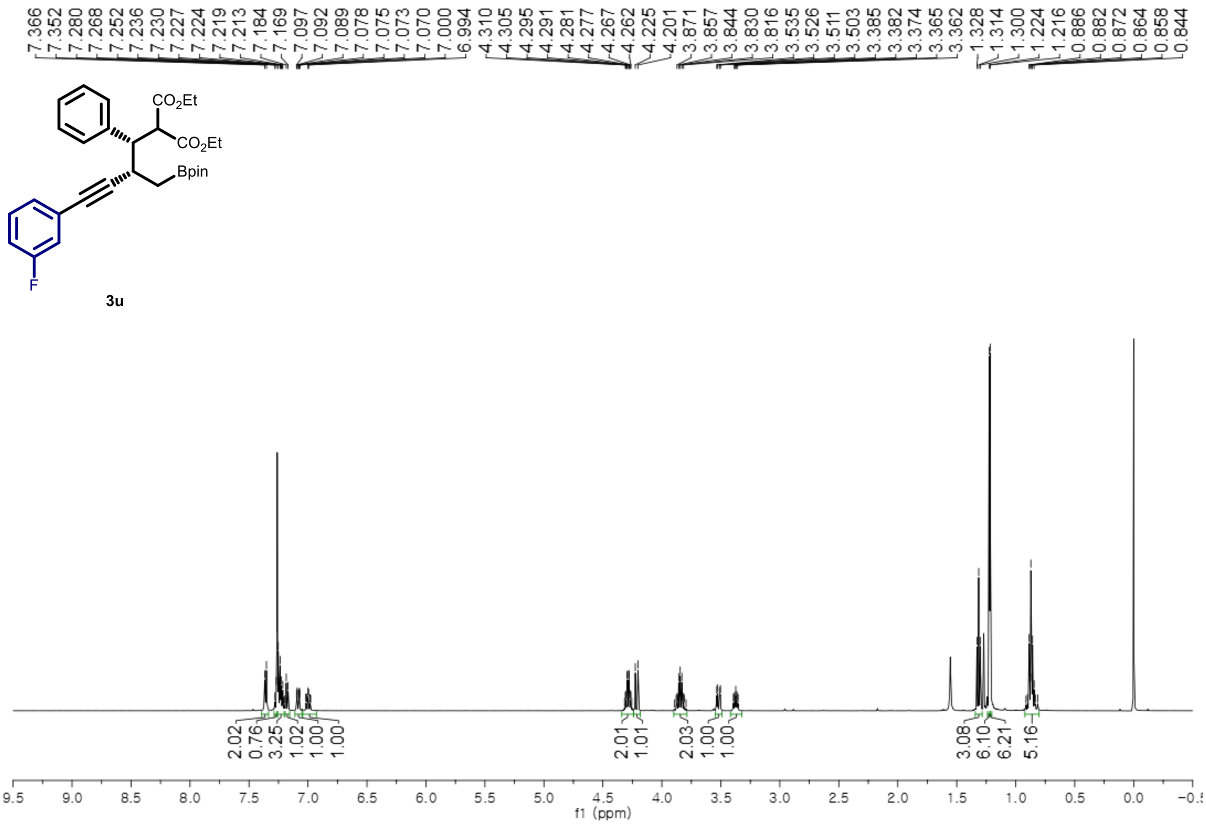

Supplementary Figure 43. ^{1}H NMR of compound **3u**.

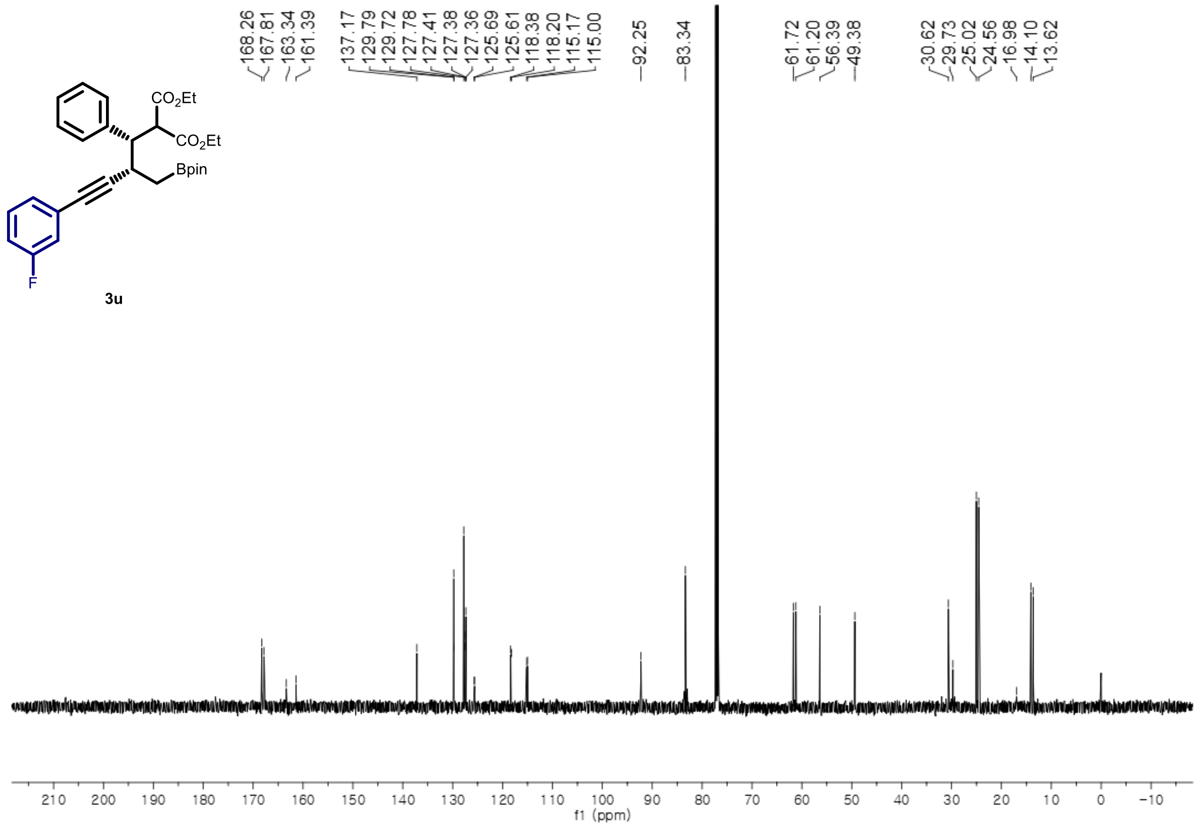

Supplementary Figure 44. ^{13}C NMR of compound **3u**.

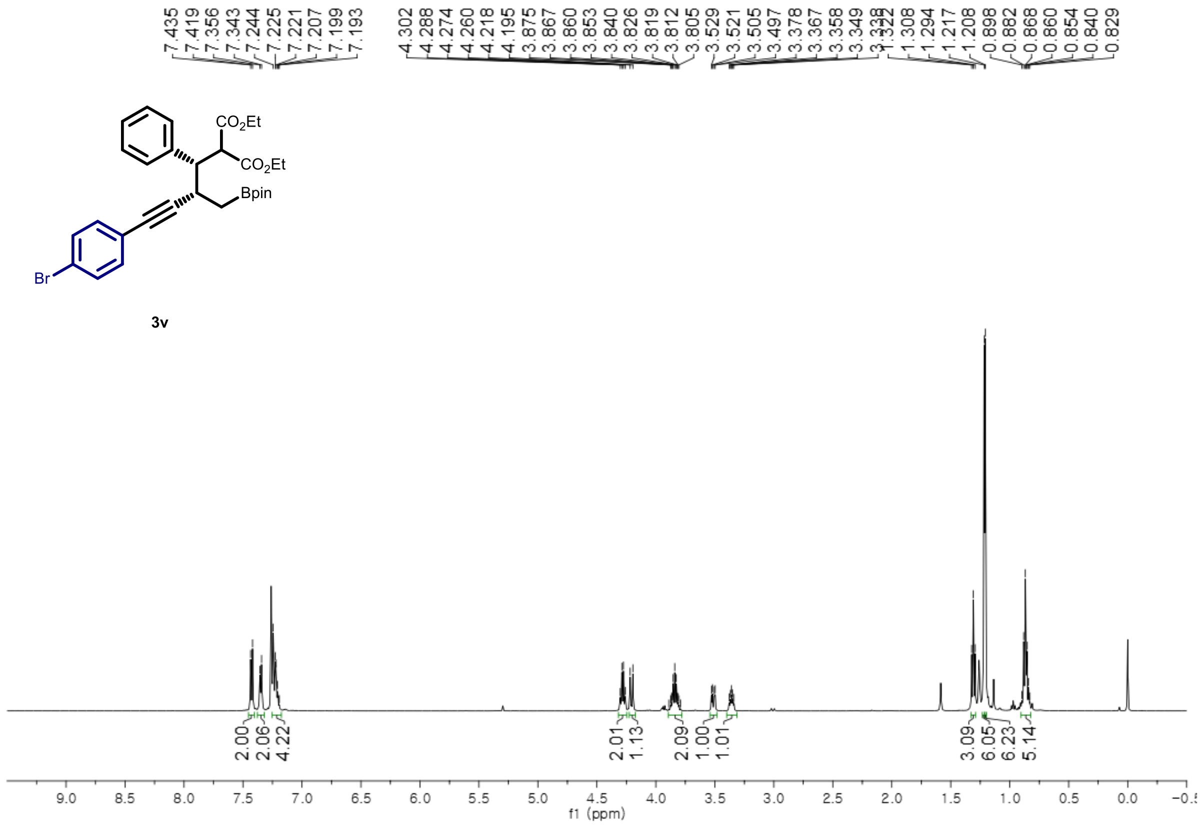

Supplementary Figure 45. ^{1}H NMR of compound **3v**.

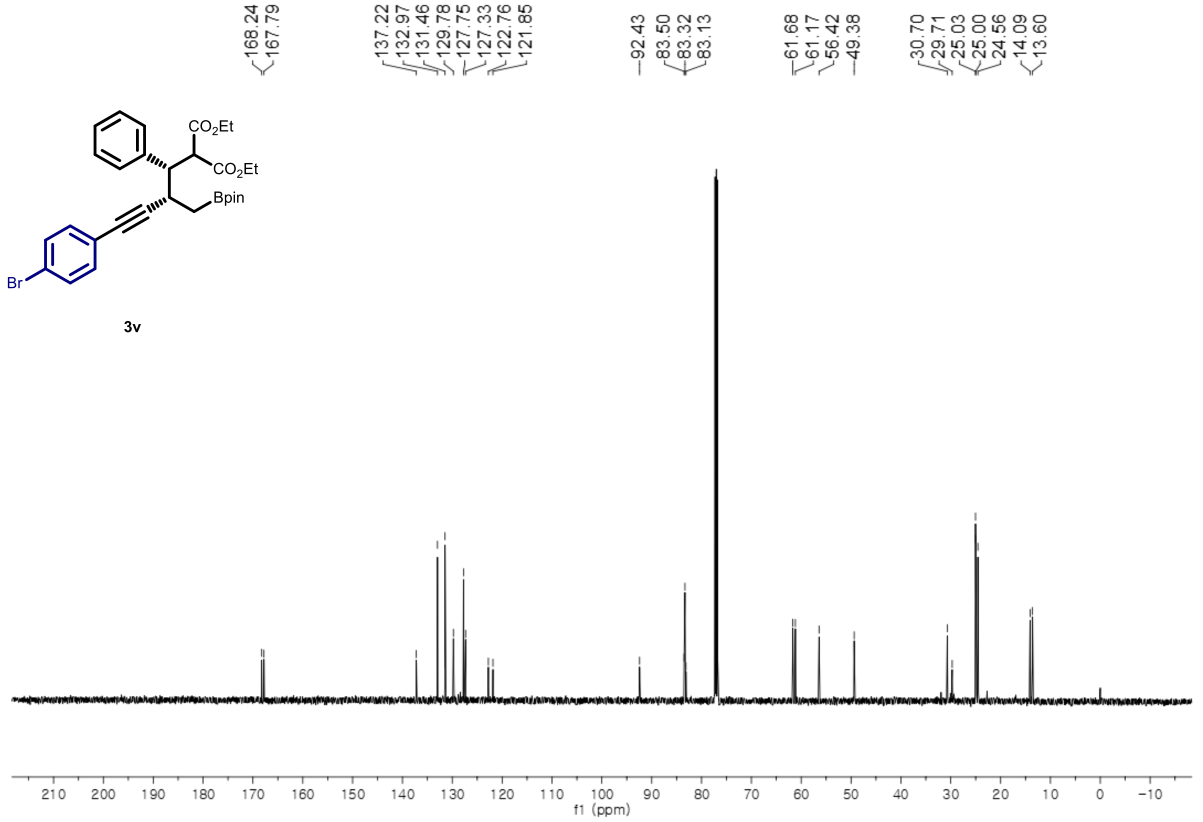

Supplementary Figure 46. ^{13}C NMR of compound **3v**.

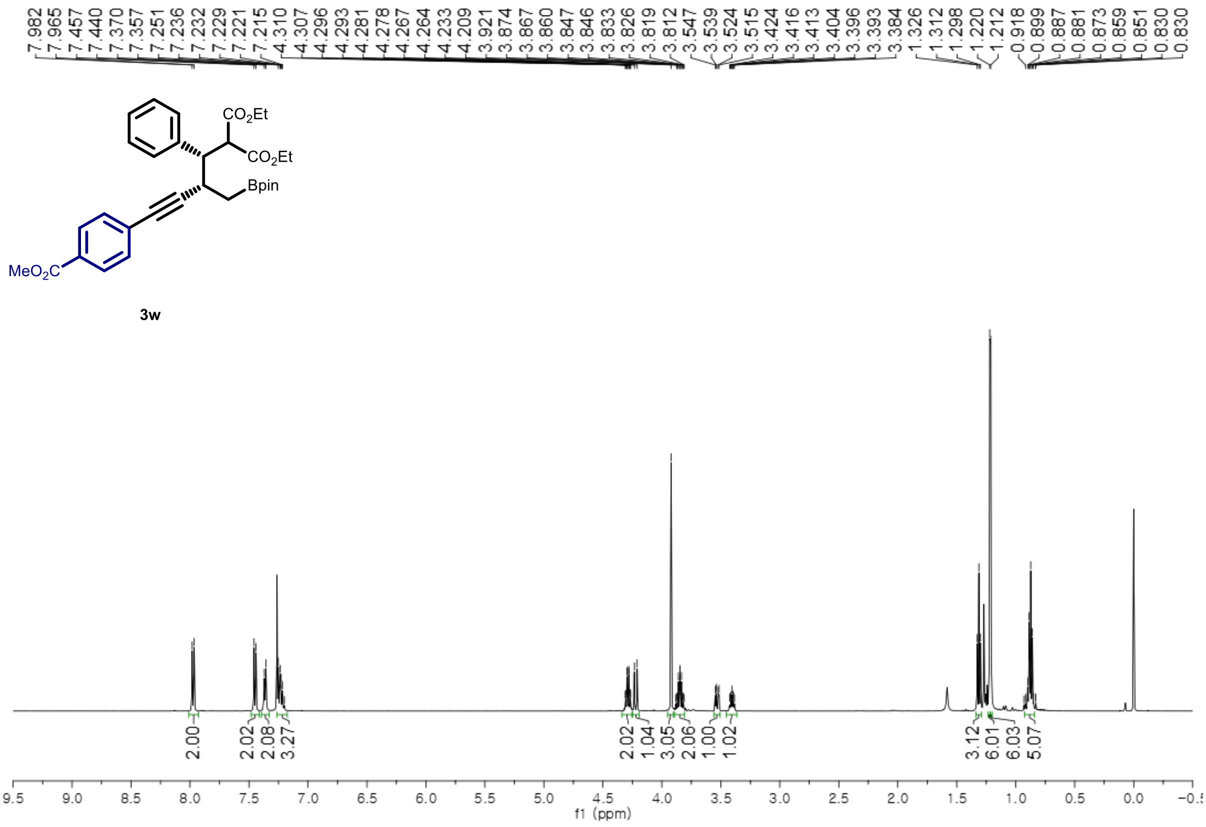

Supplementary Figure 47. ^{1}H NMR of compound **3w**.

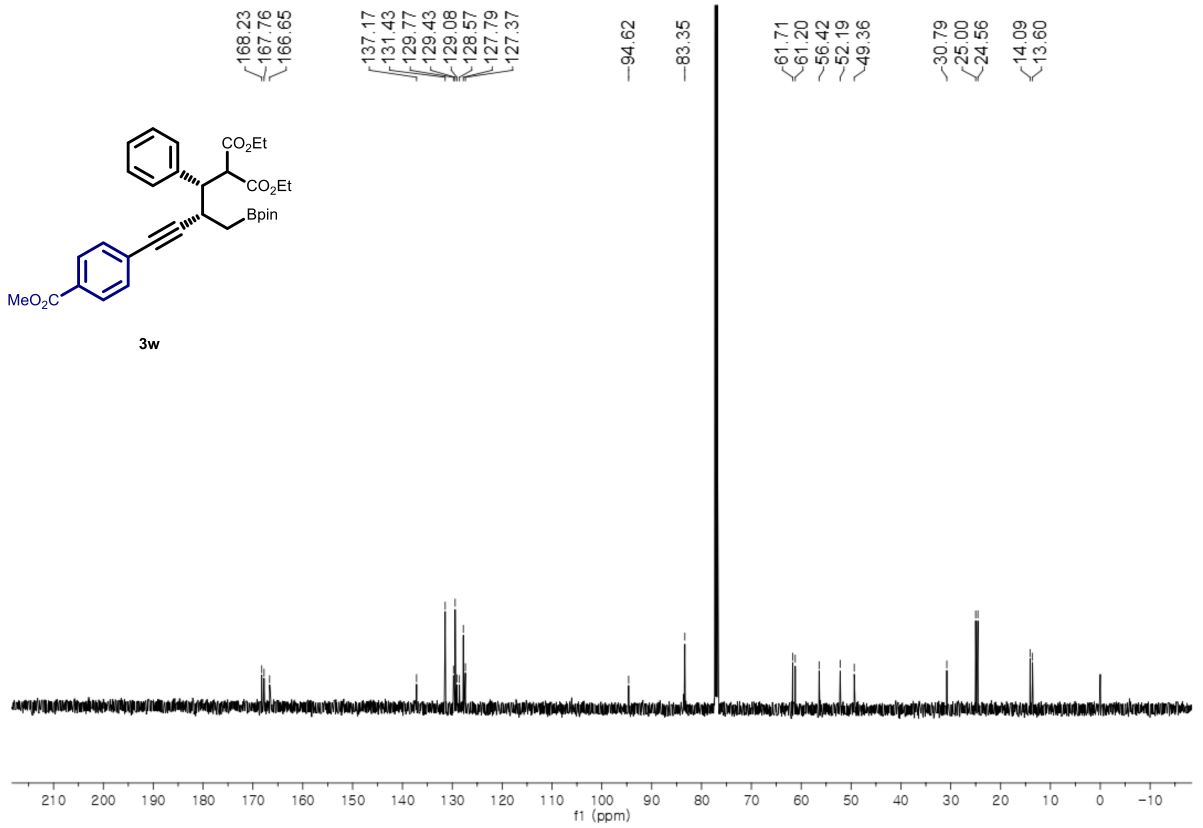

Supplementary Figure 48. ^{13}C NMR of compound **3w**.

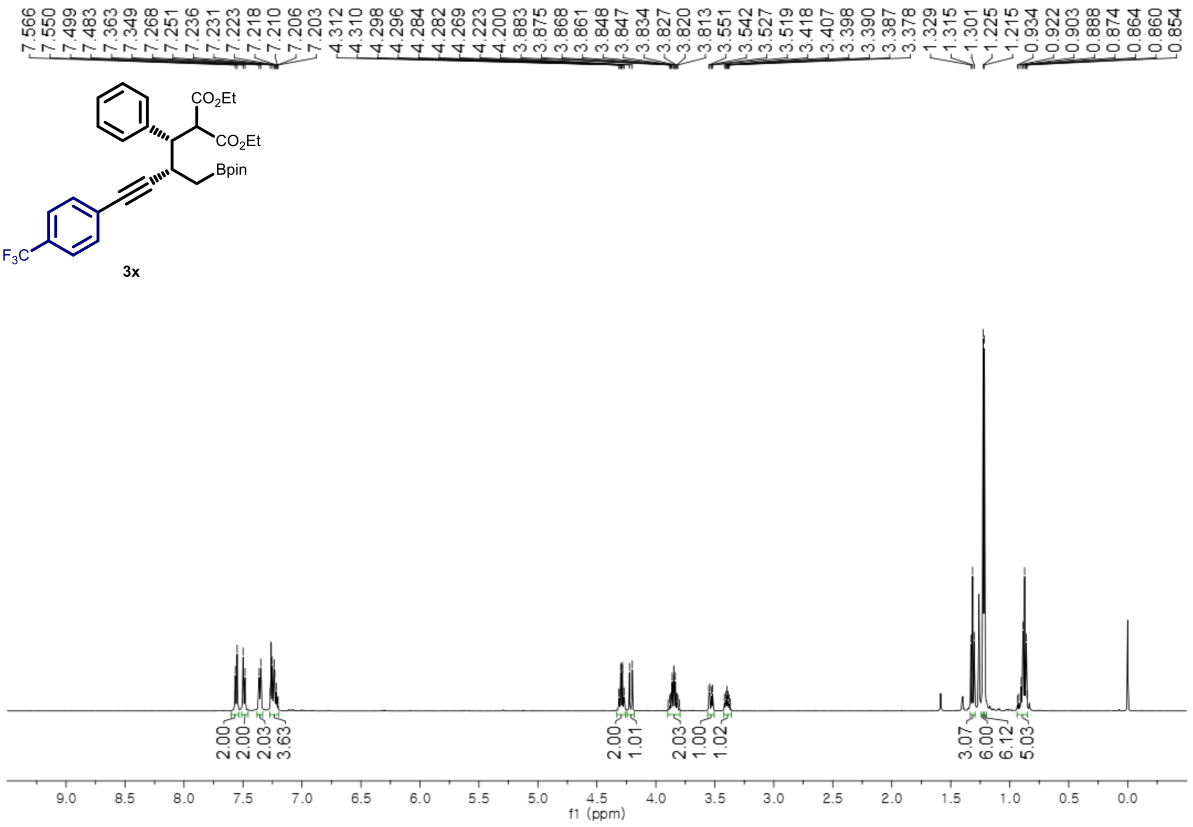

Supplementary Figure 49. ^{1}H NMR of compound **3x**.

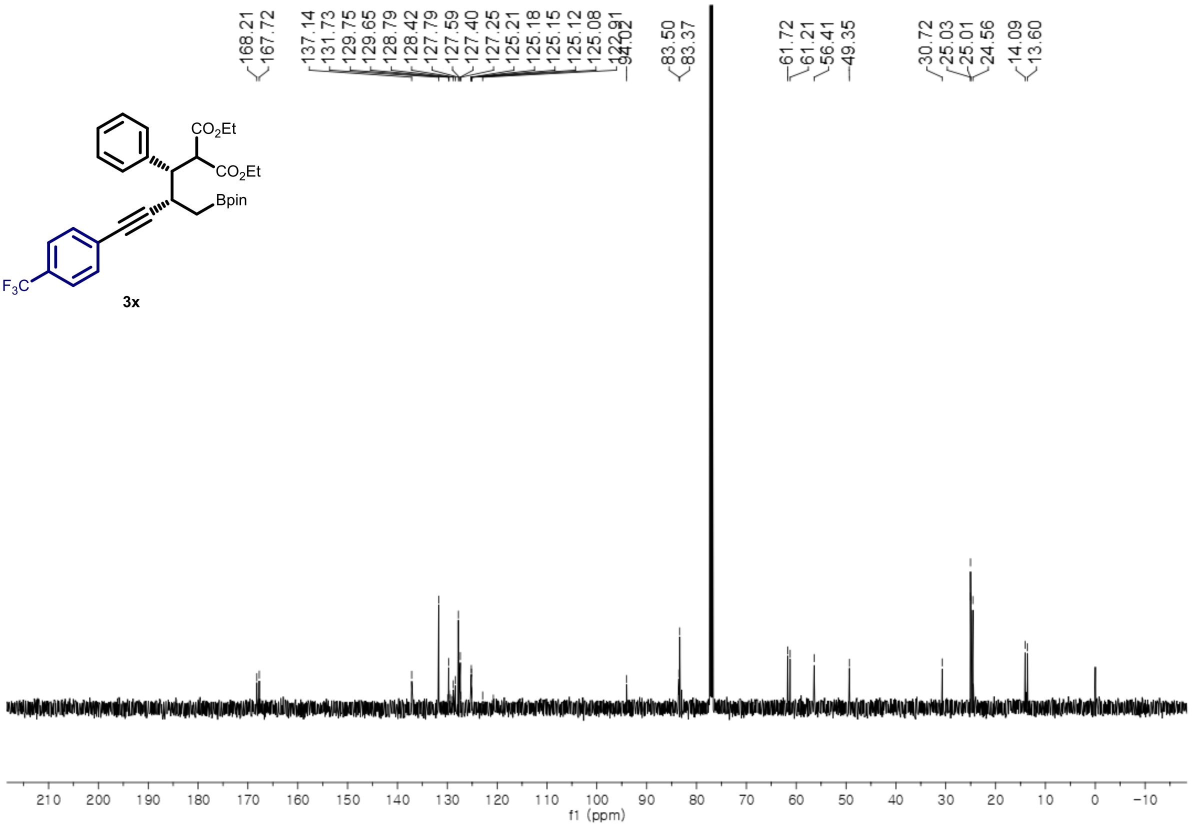

Supplementary Figure 50. ^{13}C NMR of compound **3x**.

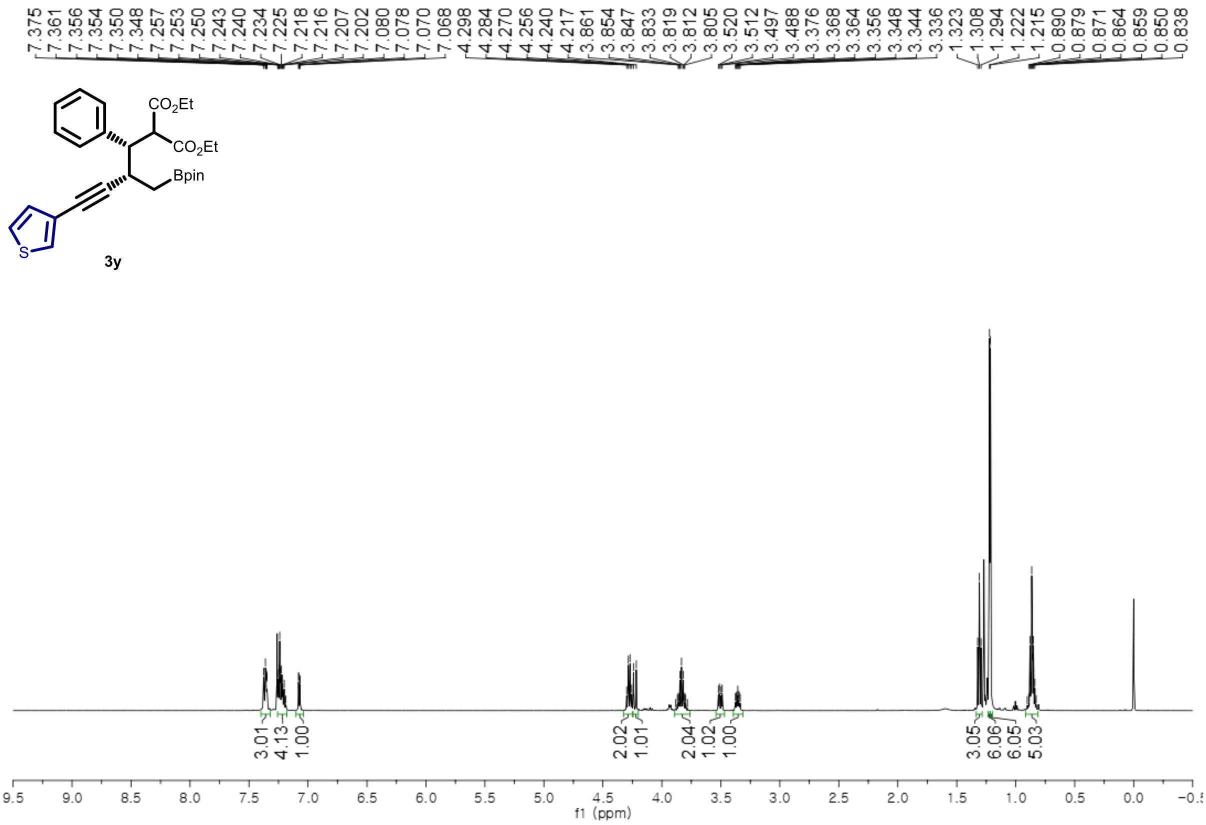

Supplementary Figure 51. ^{1}H NMR of compound **3y**.

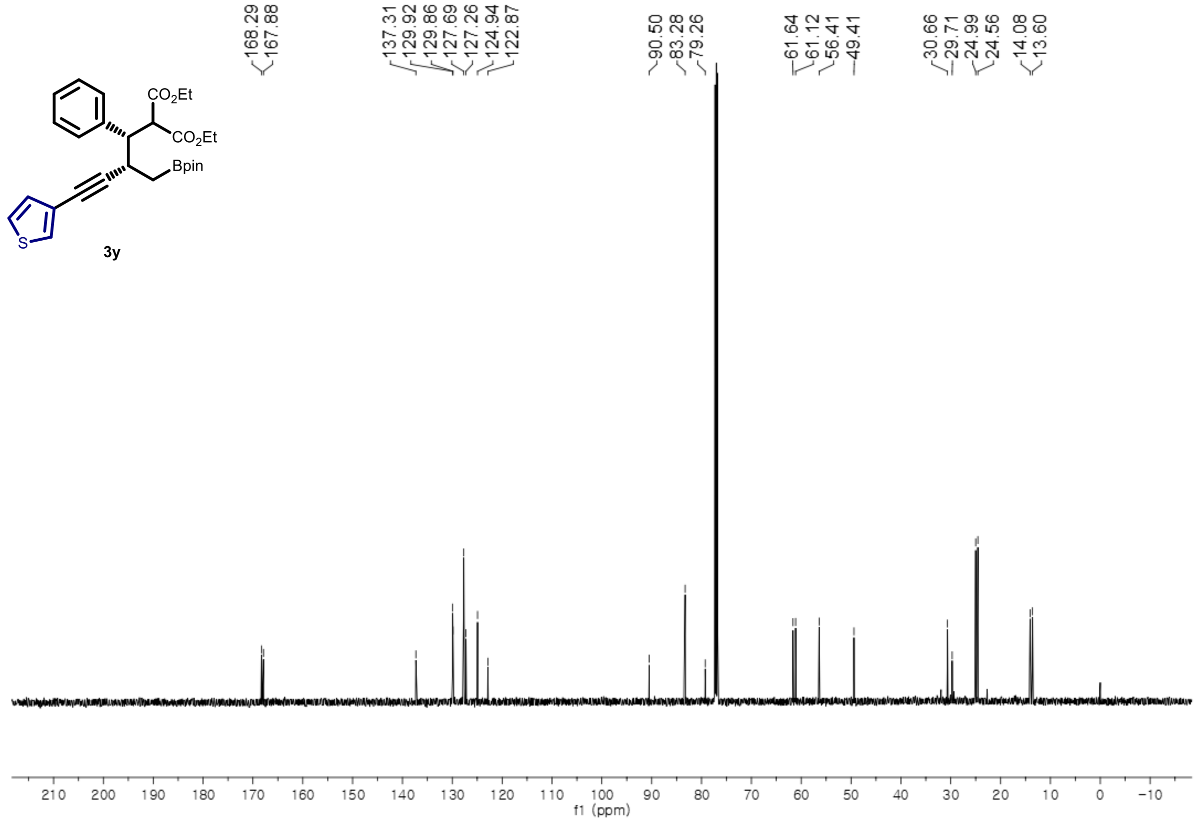

Supplementary Figure 52. ^{13}C NMR of compound **3y**.

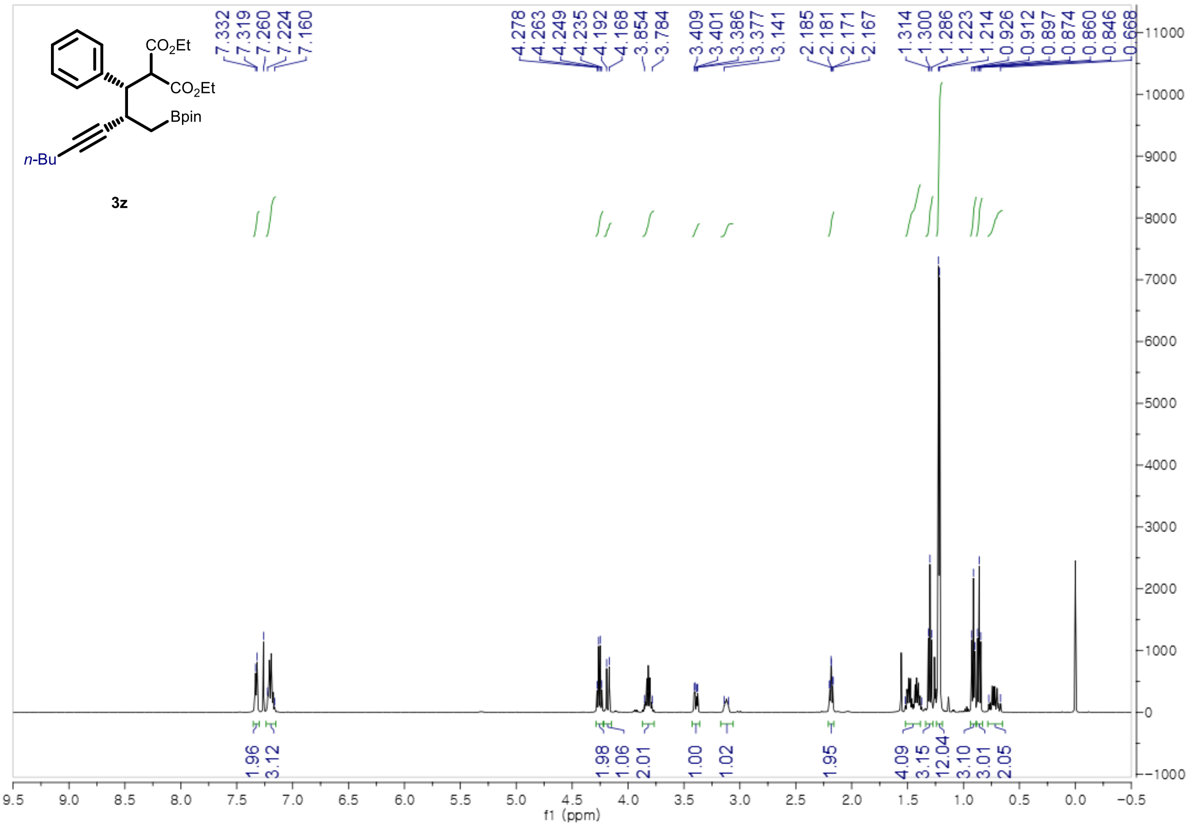

Supplementary Figure 53. ^{1}H NMR of compound **3z**.

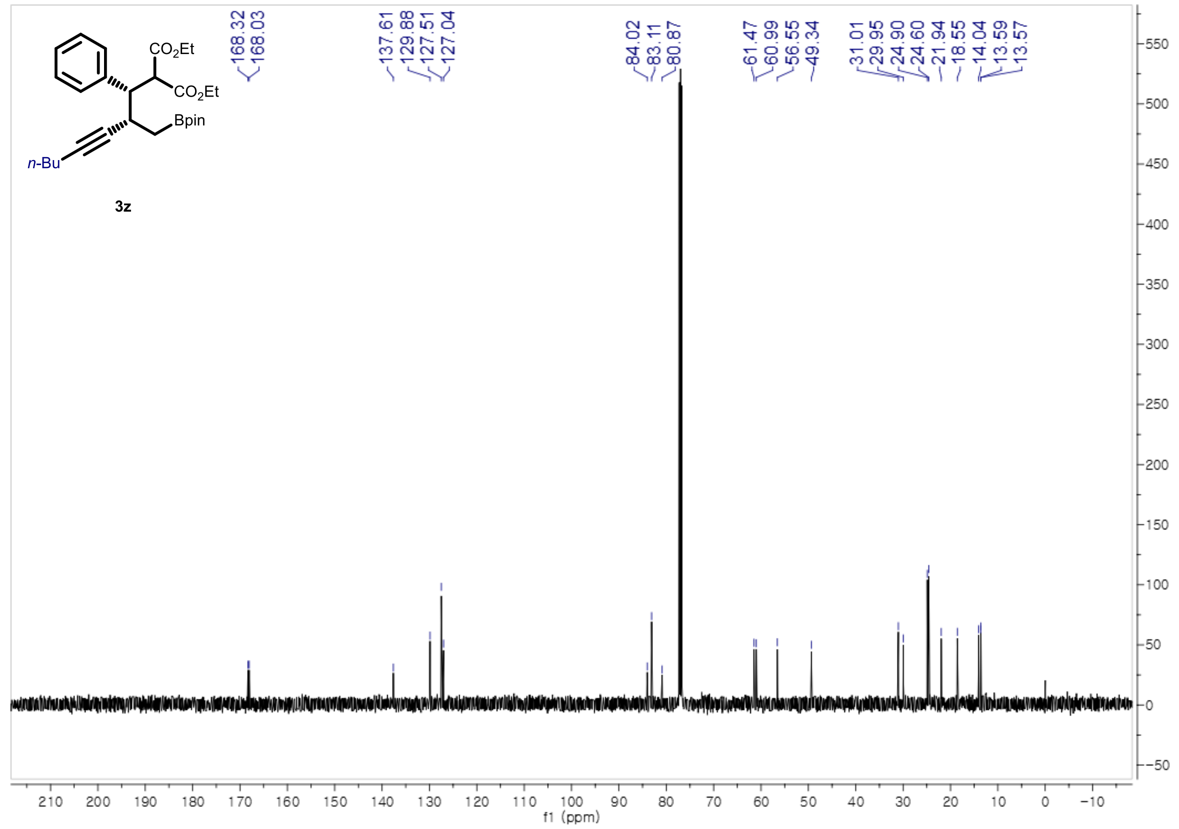

Supplementary Figure 54. ^{13}C NMR of compound **3z**.

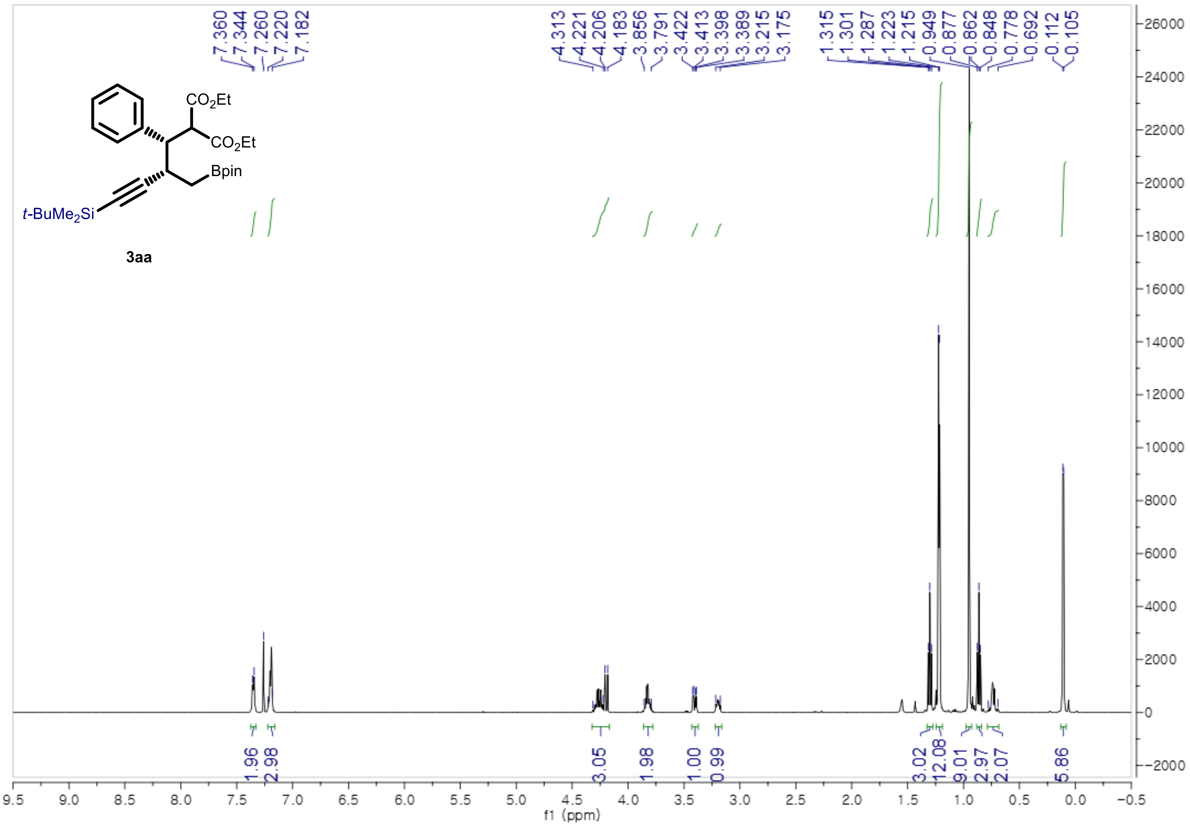

Supplementary Figure 55. ^{1}H NMR of compound **3aa**.

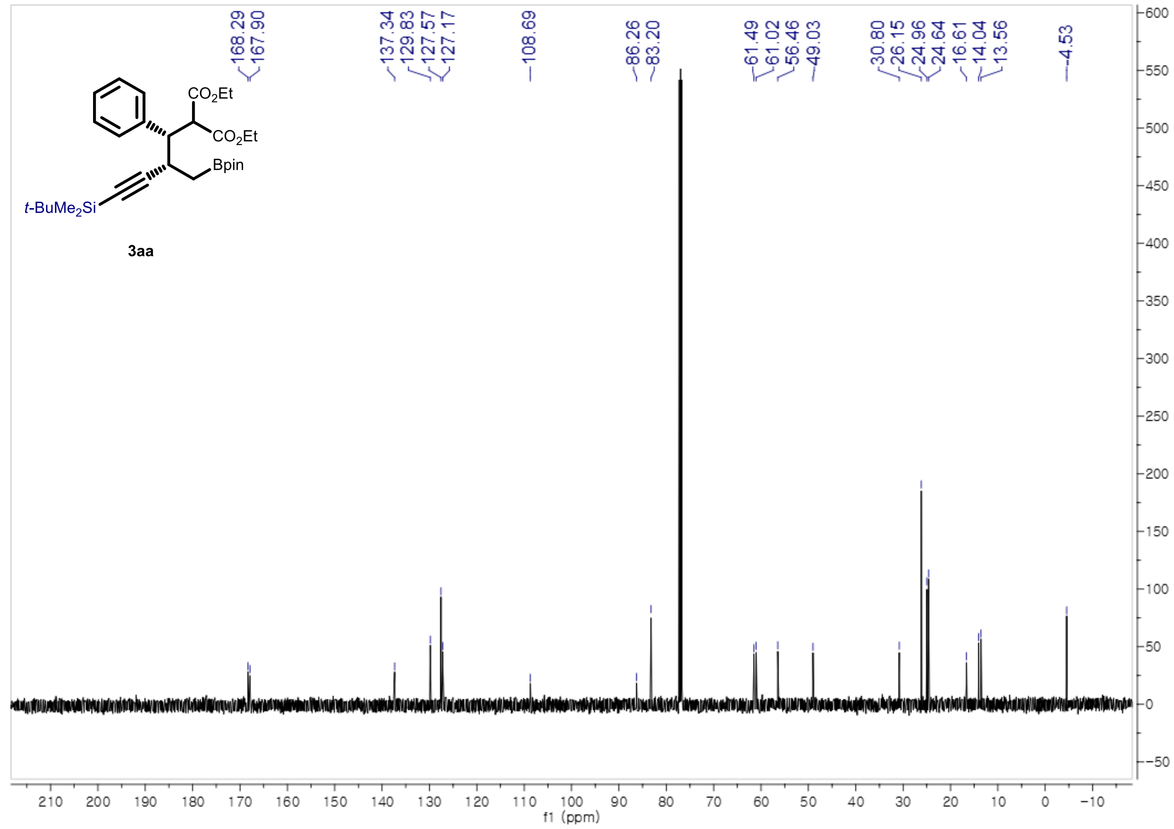

Supplementary Figure 56. ^{13}C NMR of compound **3aa**.

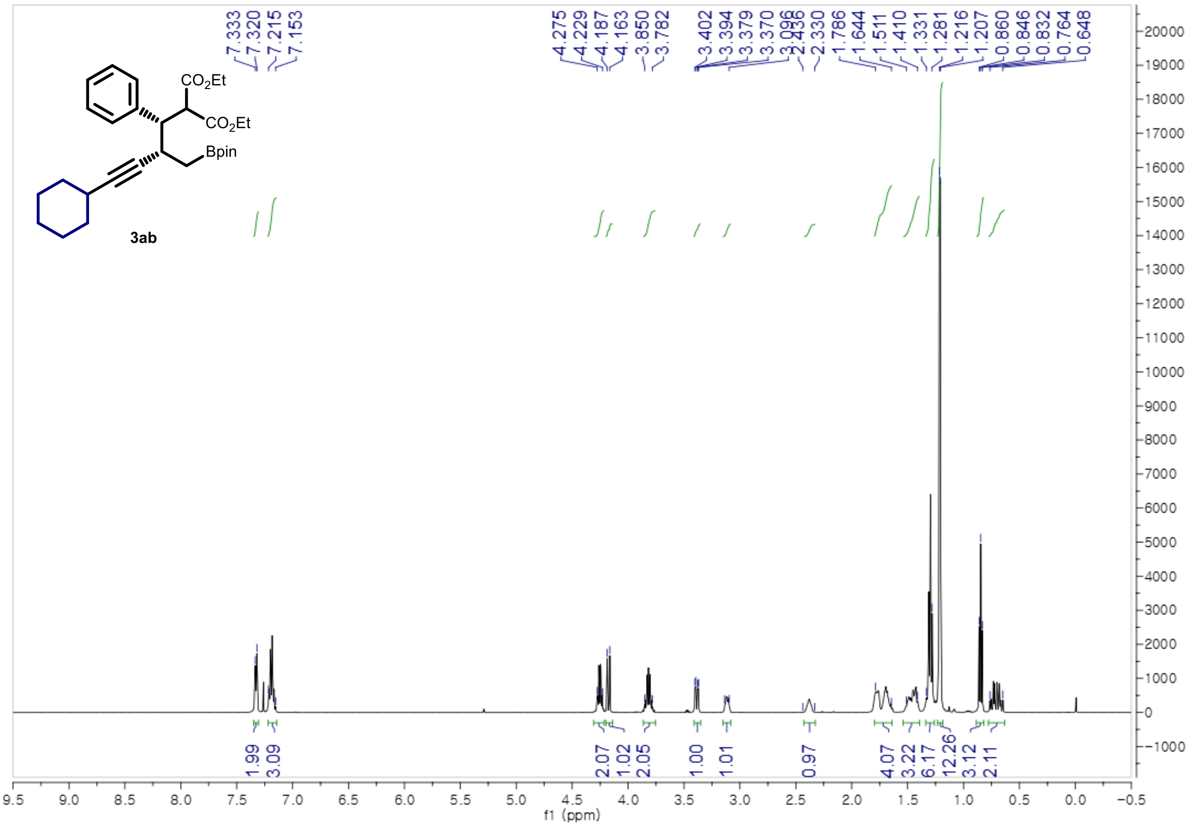

Supplementary Figure 57. ^{1}H NMR of compound **3ab**.

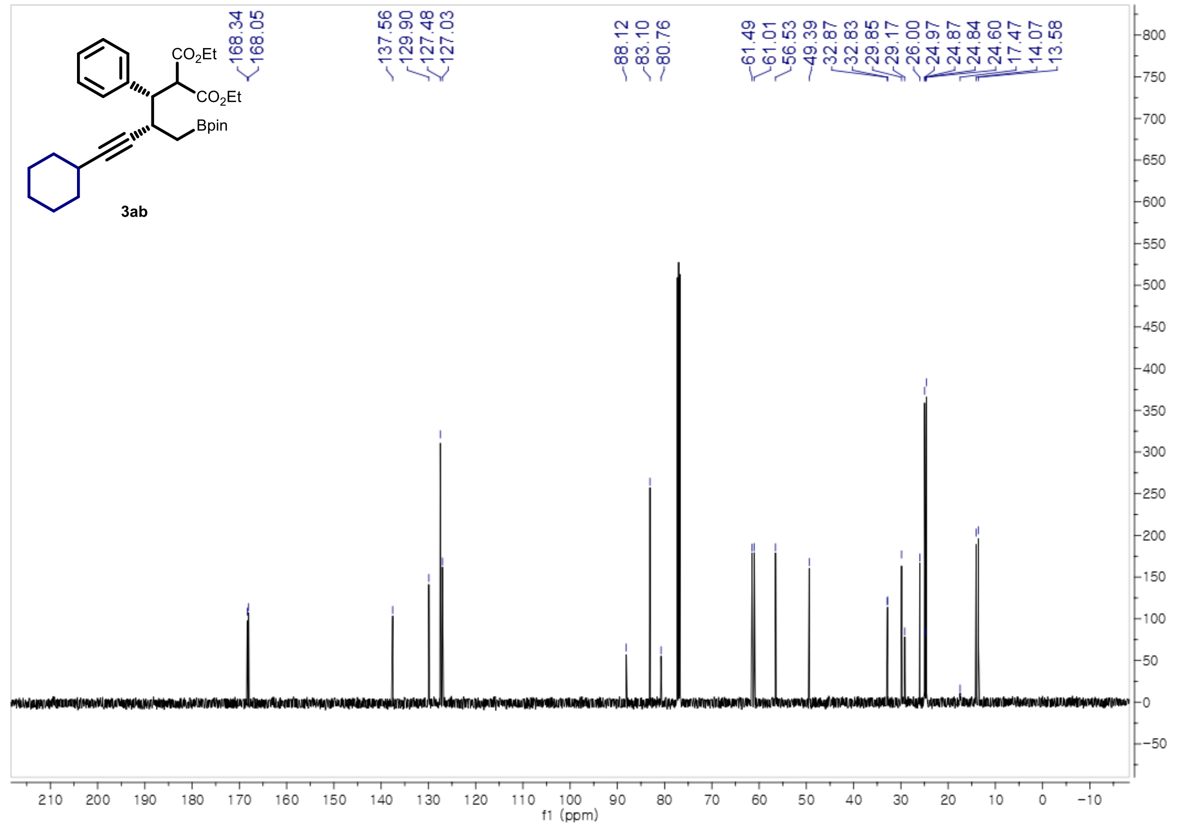

Supplementary Figure 58. ^{13}C NMR of compound **3ab**.

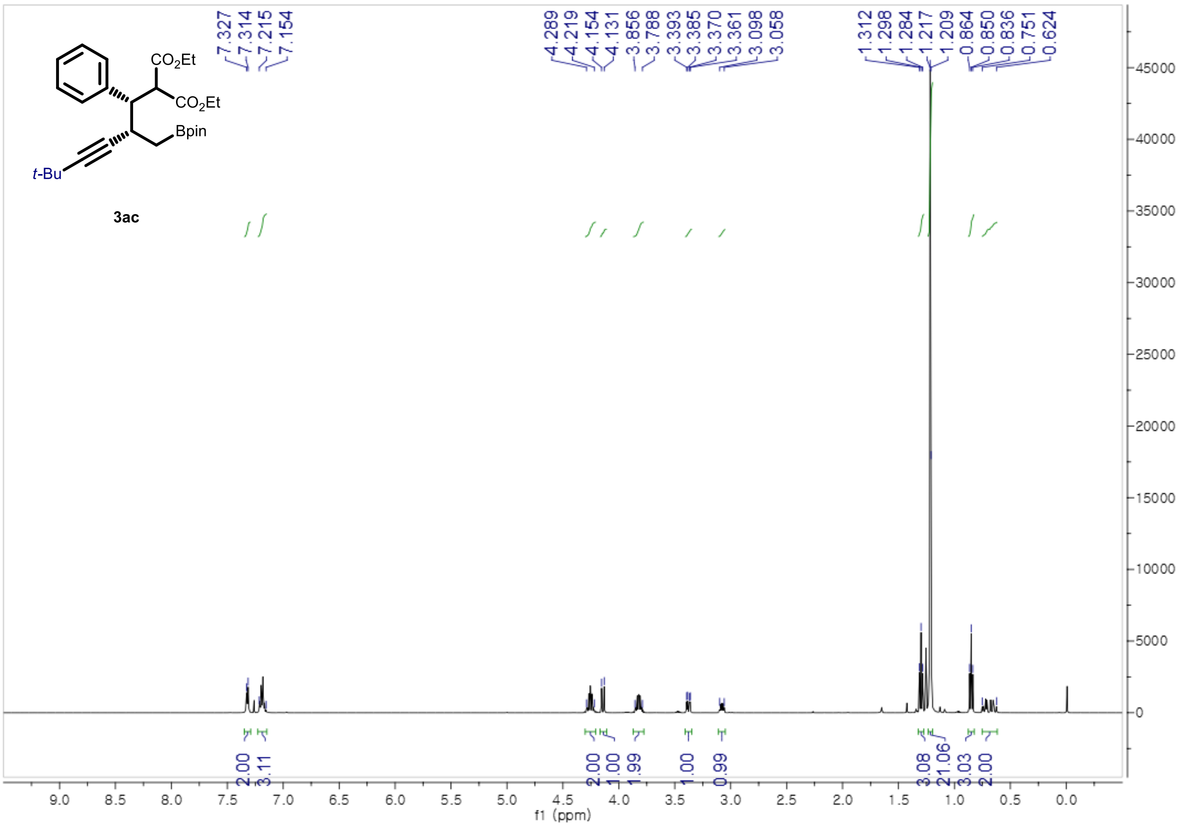

Supplementary Figure 59. ^{1}H NMR of compound **3ac**.

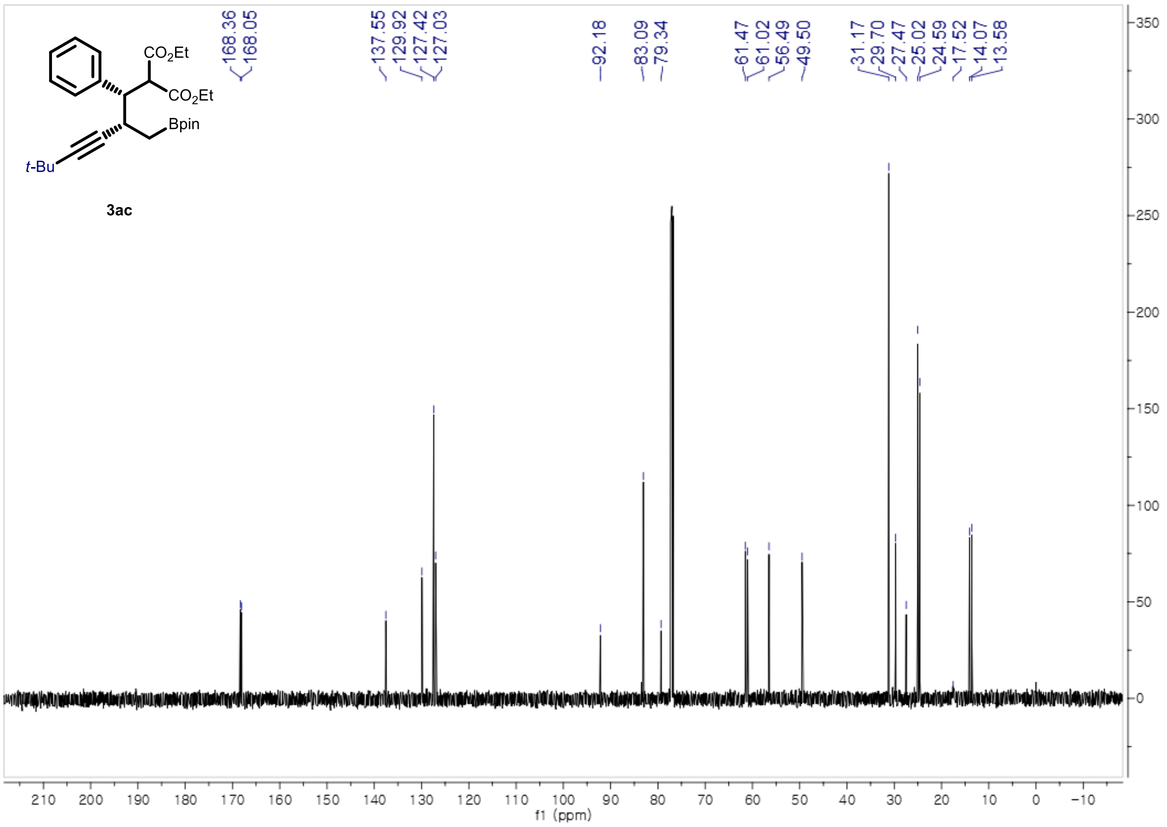

Supplementary Figure 60. ^{13}C NMR of compound **3ac**.

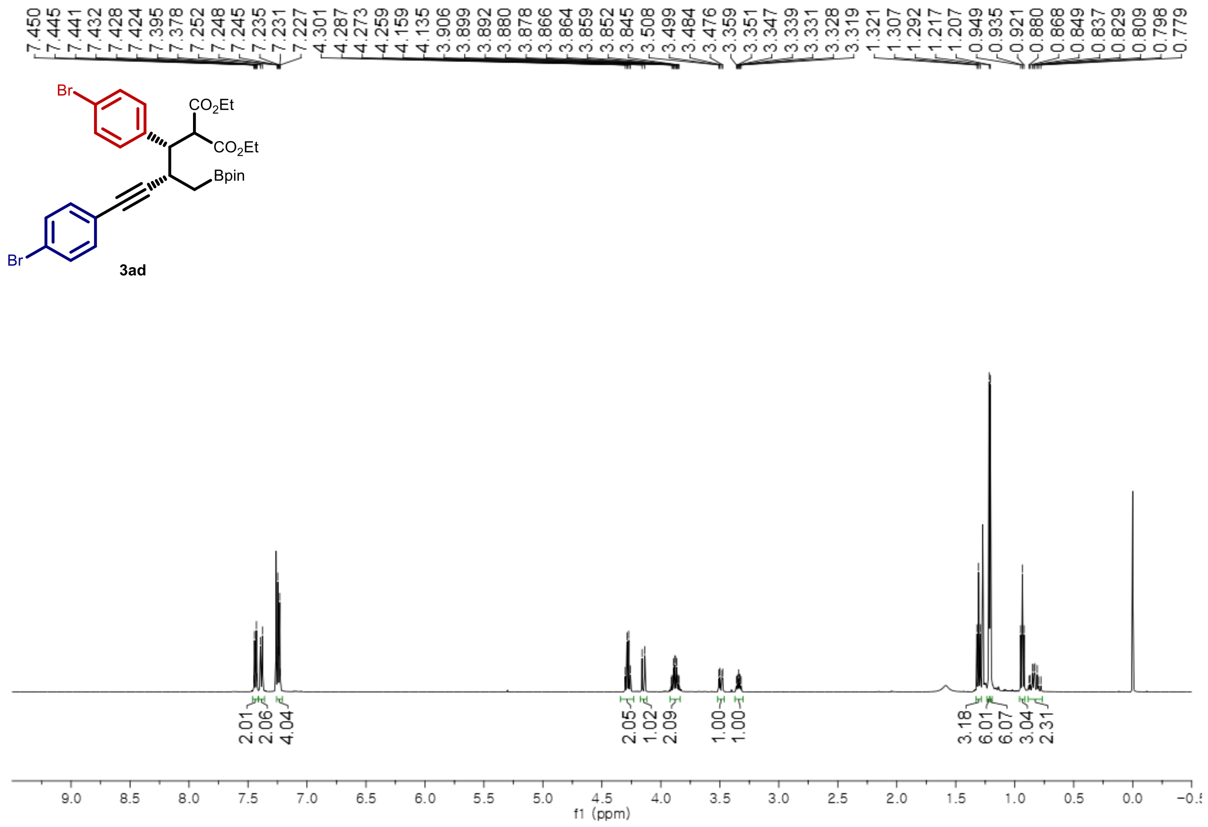

Supplementary Figure 61. ^{1}H NMR of compound **3ad**.

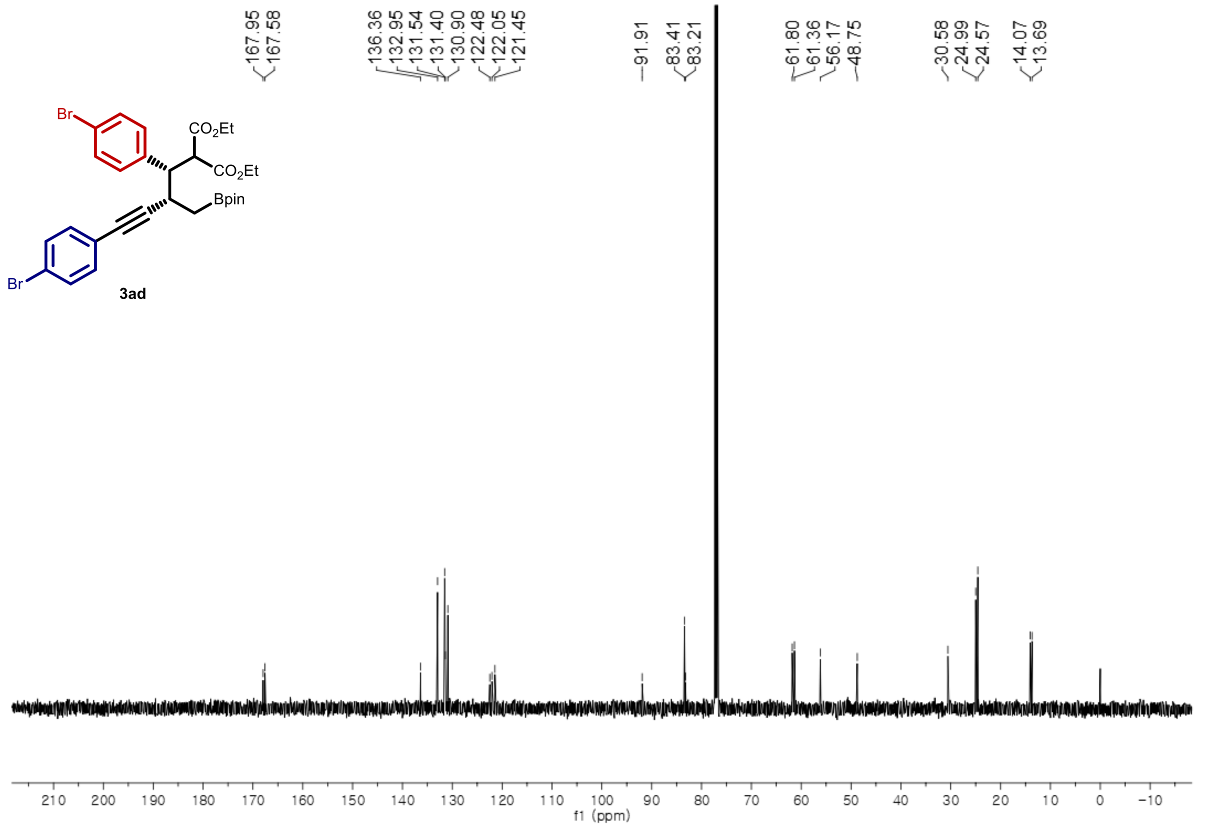

Supplementary Figure 62. ^{13}C NMR of compound **3ad**.

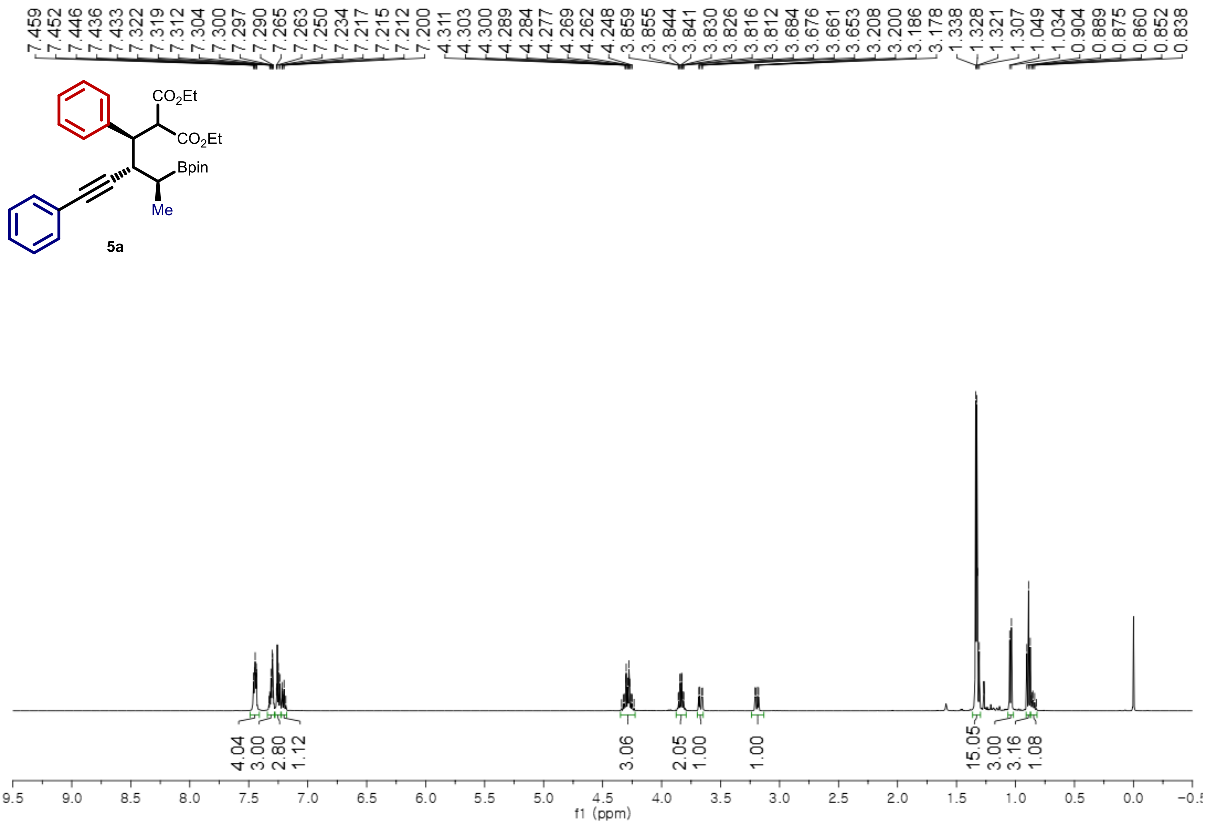

Supplementary Figure 63. ^{1}H NMR of compound **5a**.

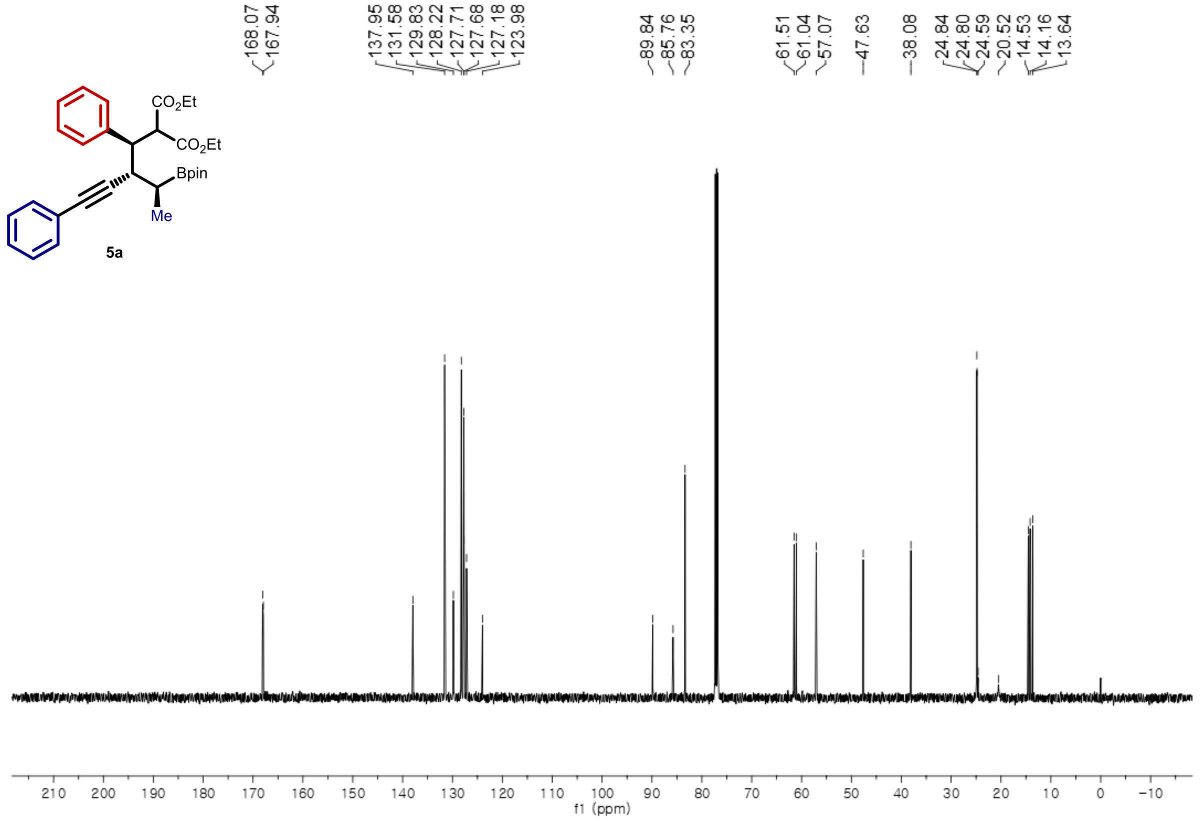

Supplementary Figure 64. ^{13}C NMR of compound **5a**.

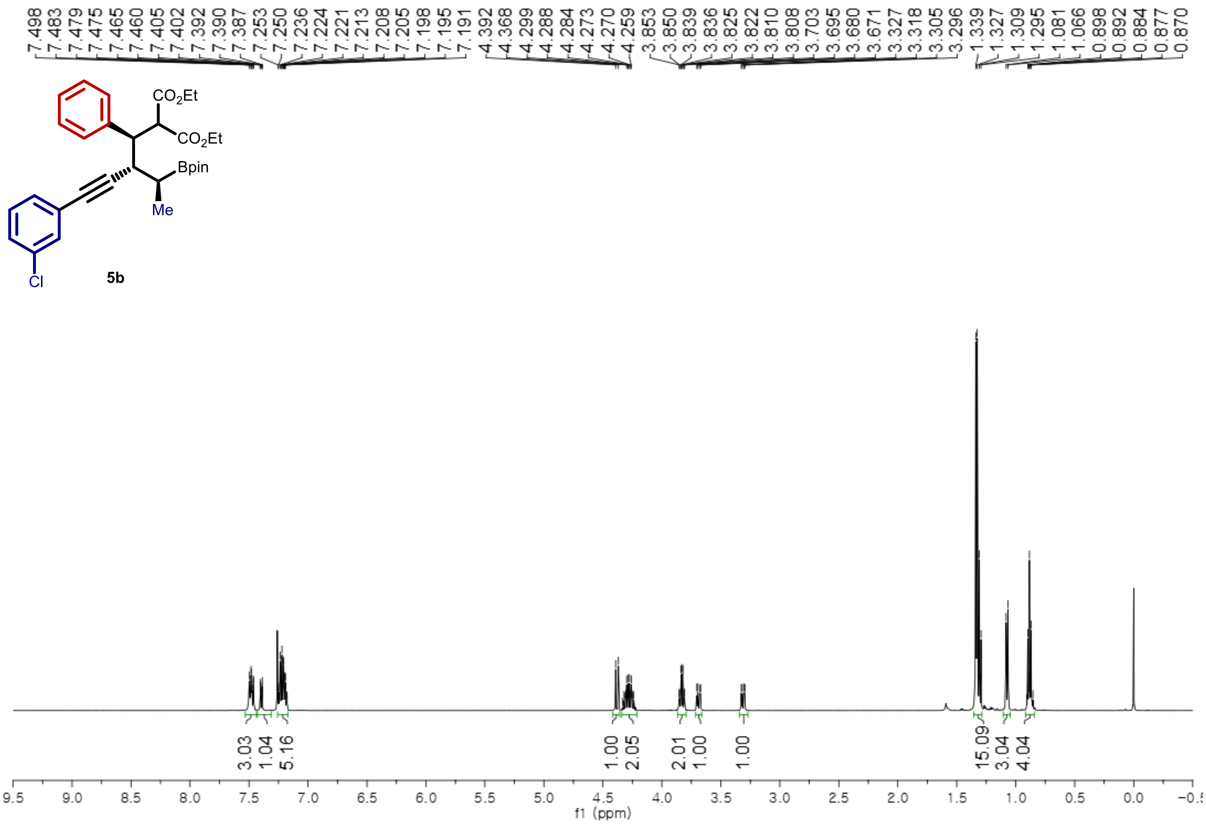

Supplementary Figure 65. ^{1}H NMR of compound **5b**.

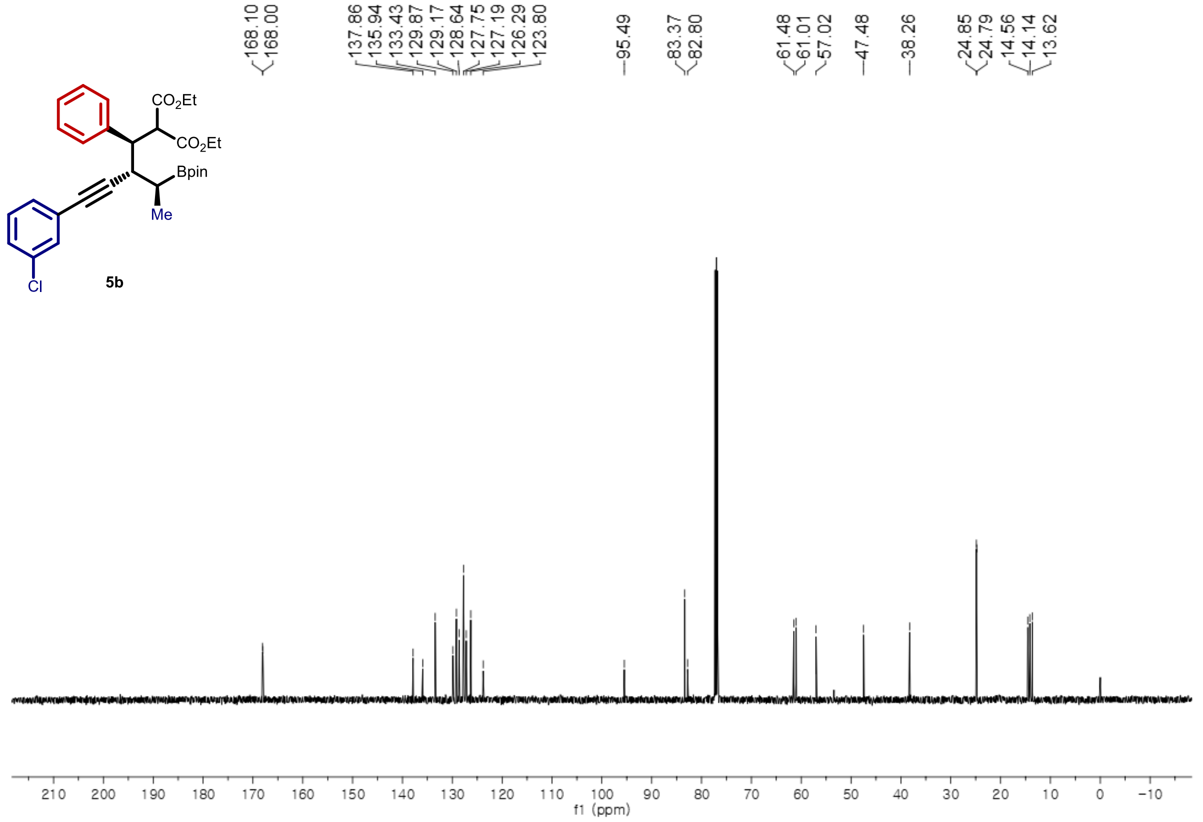

Supplementary Figure 66. ^{13}C NMR of compound **5b**.

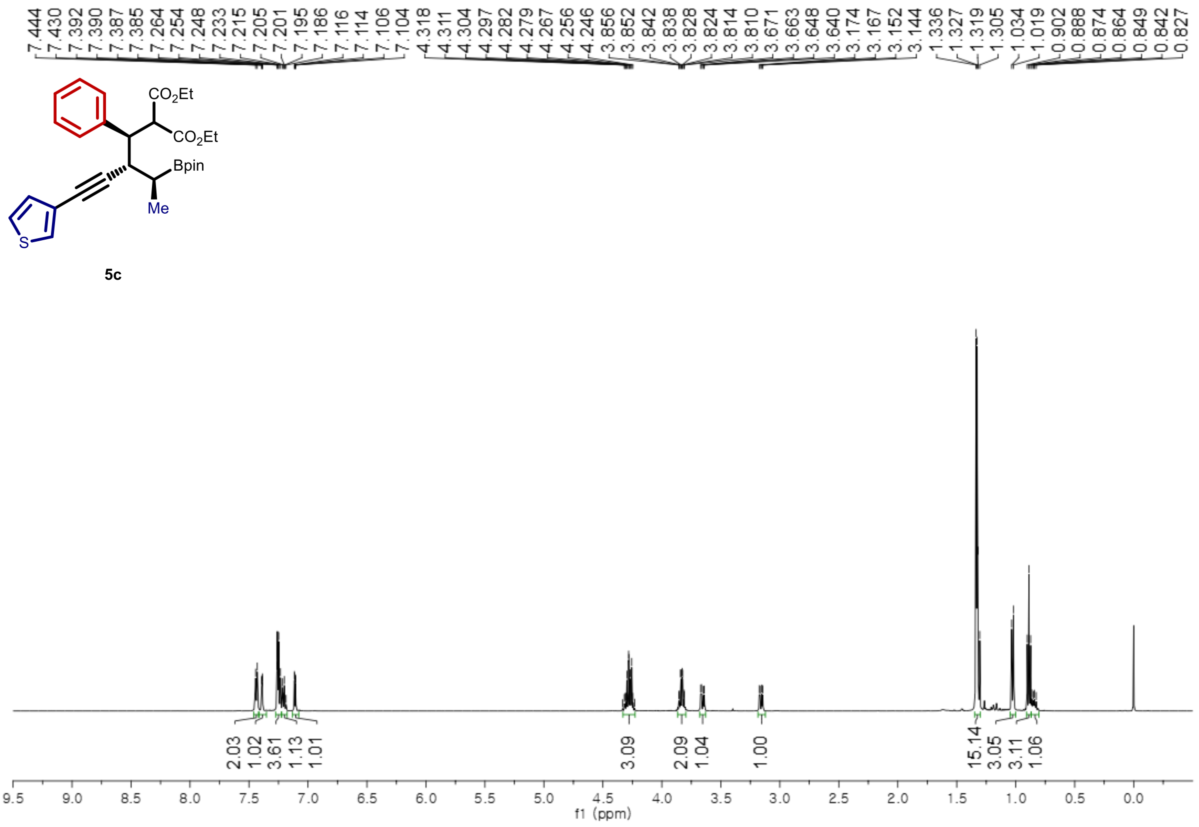

Supplementary Figure 67. ^{1}H NMR of compound **5c**.

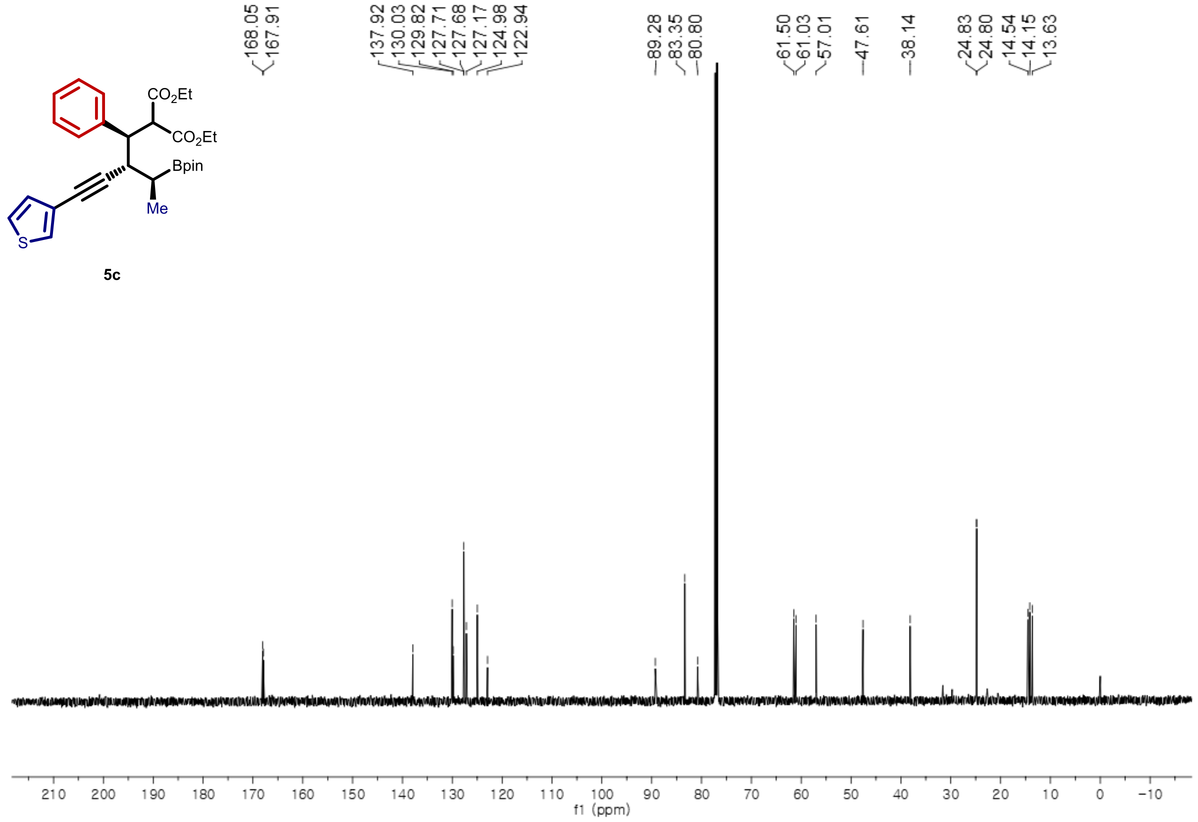

Supplementary Figure 68. ^{13}C NMR of compound **5c**.

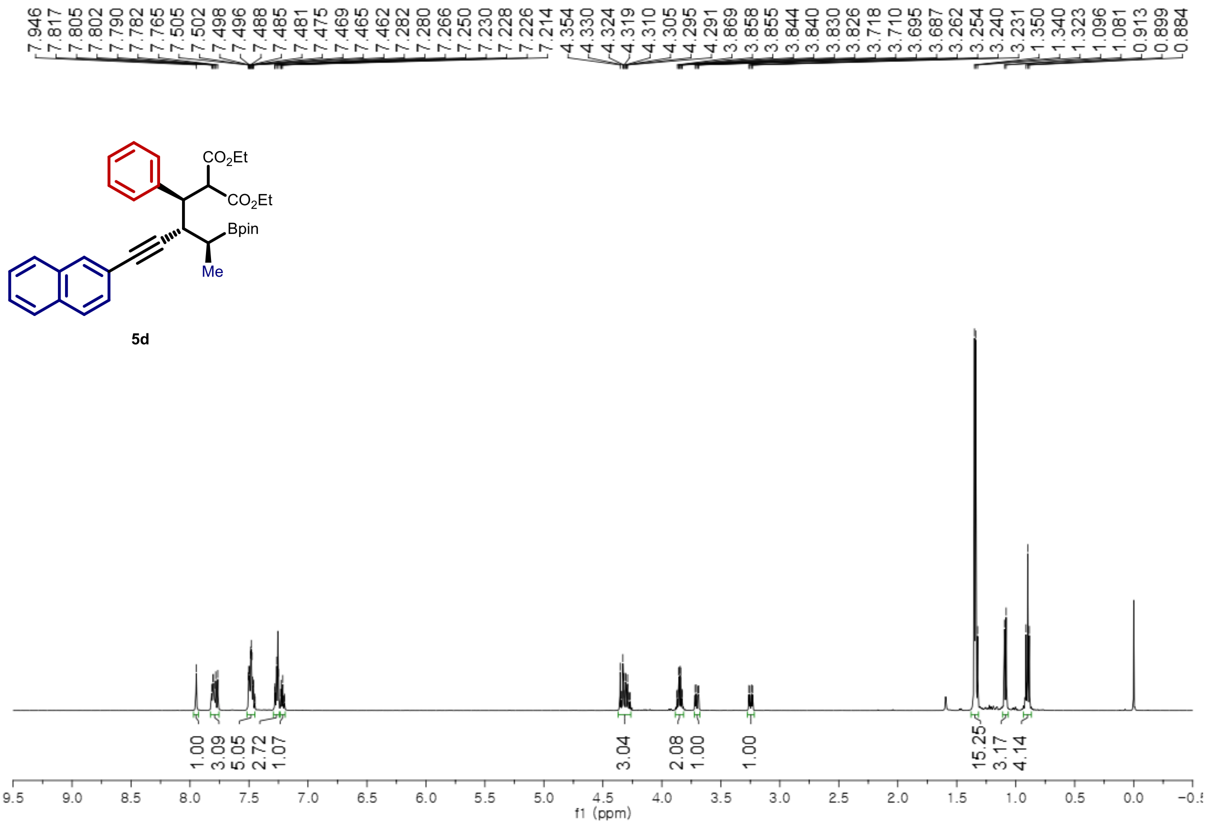

Supplementary Figure 69. ^{1}H NMR of compound **5d**.

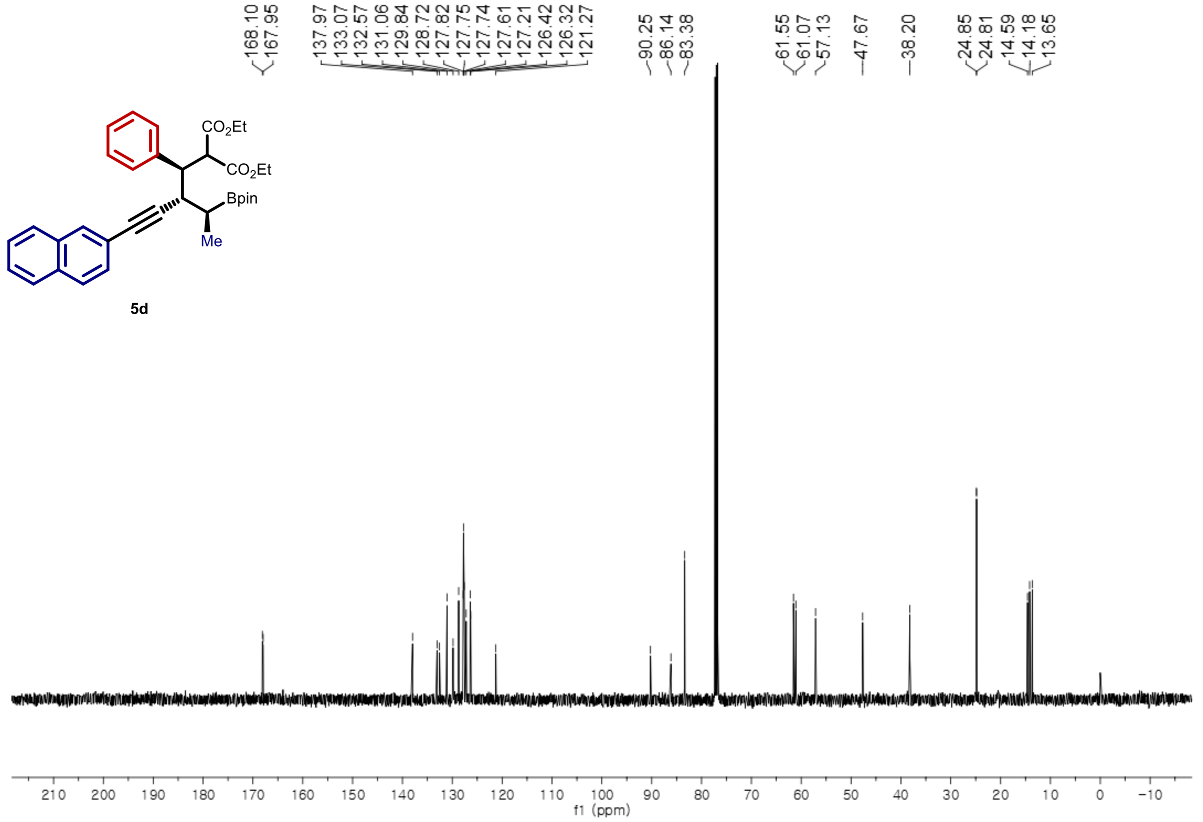

Supplementary Figure 70. ^{13}C NMR of compound **5d**.

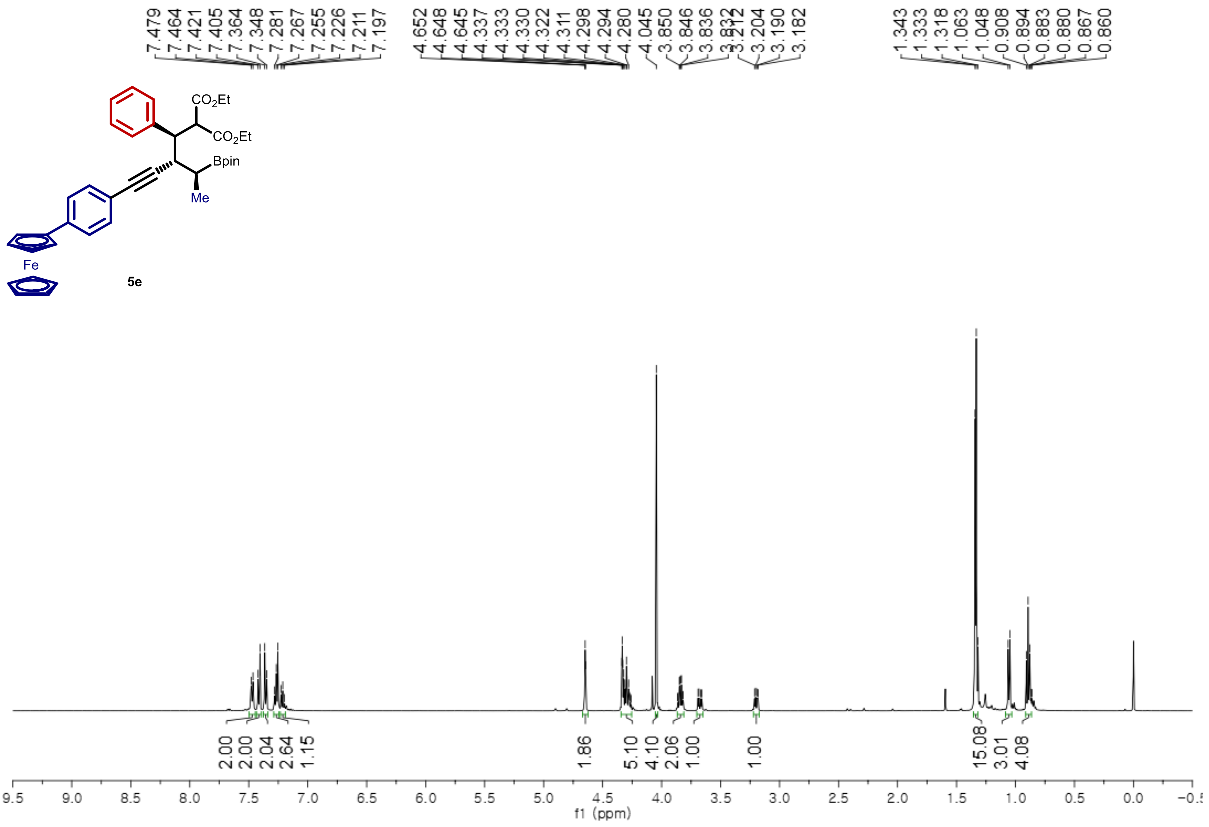

Supplementary Figure 71. ^{1}H NMR of compound **5e**.

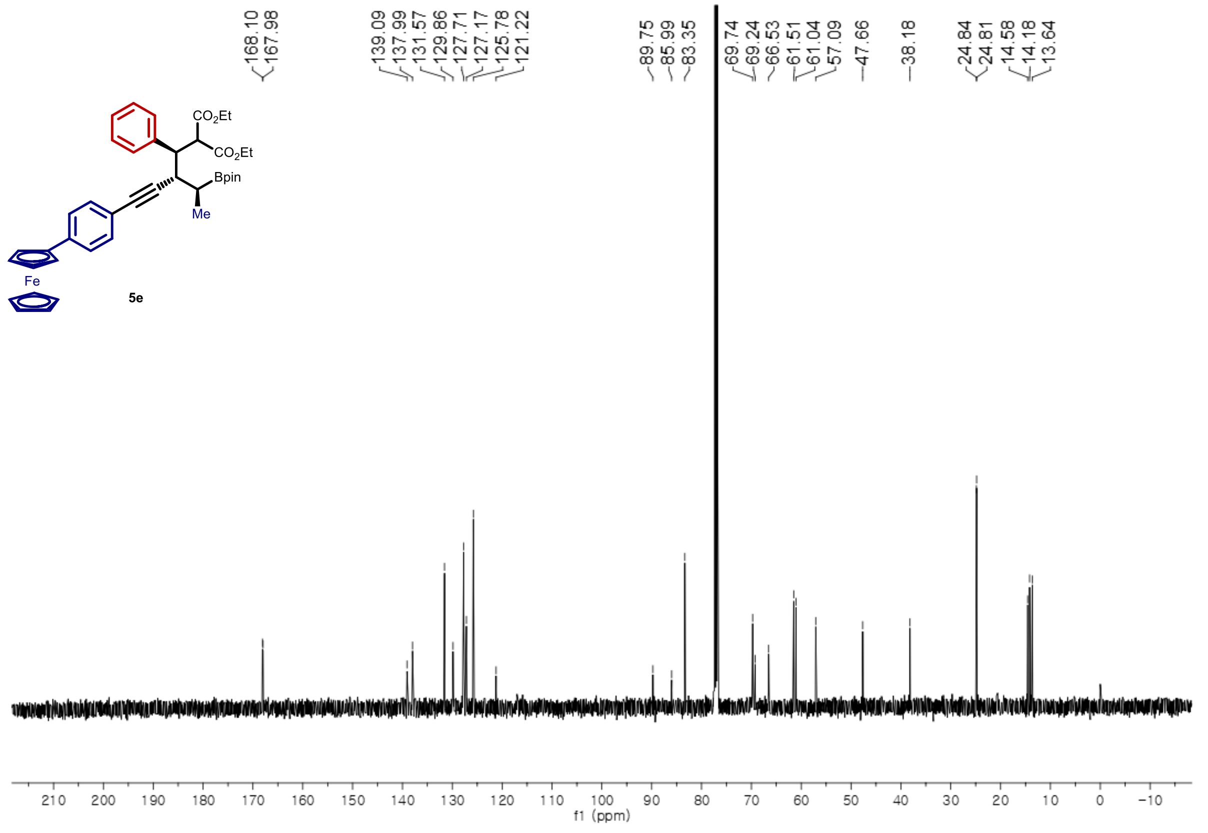

Supplementary Figure 72. ^{13}C NMR of compound **5e**.

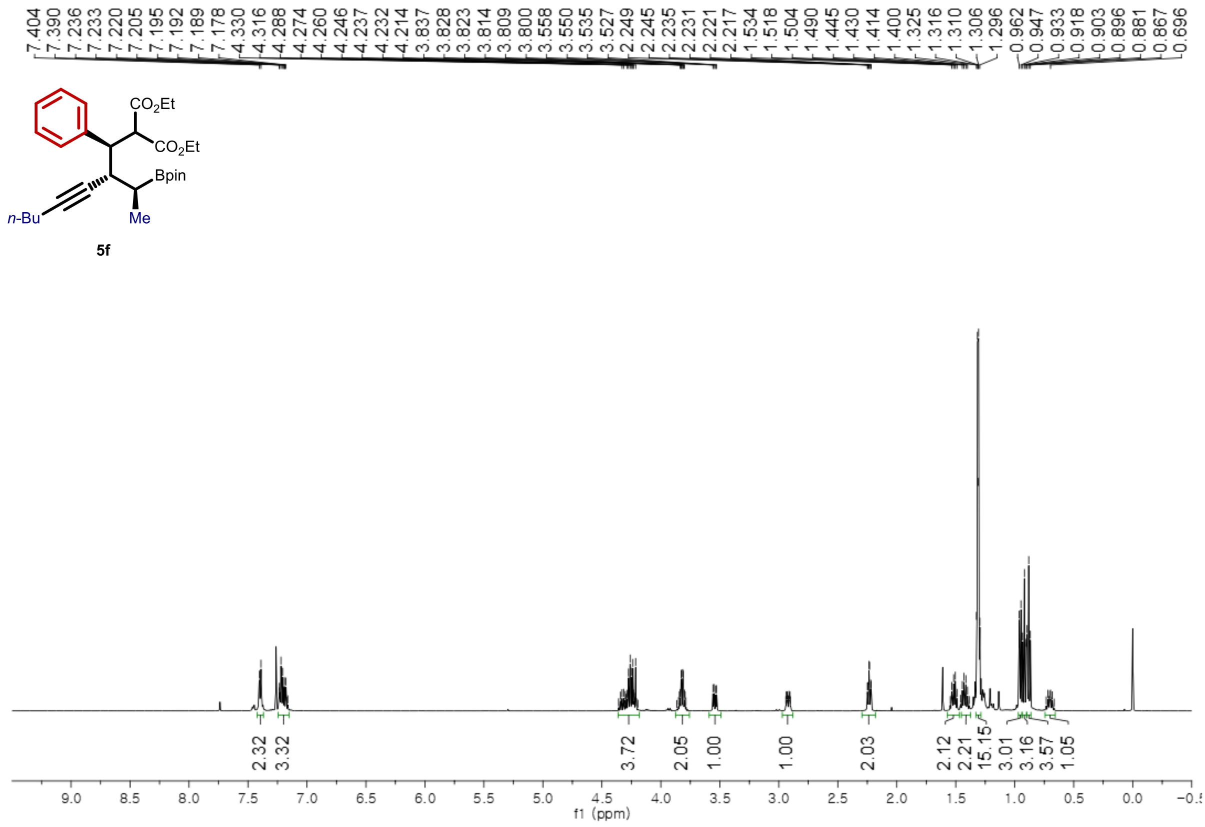

Supplementary Figure 73. ^{1}H NMR of compound **5f**.

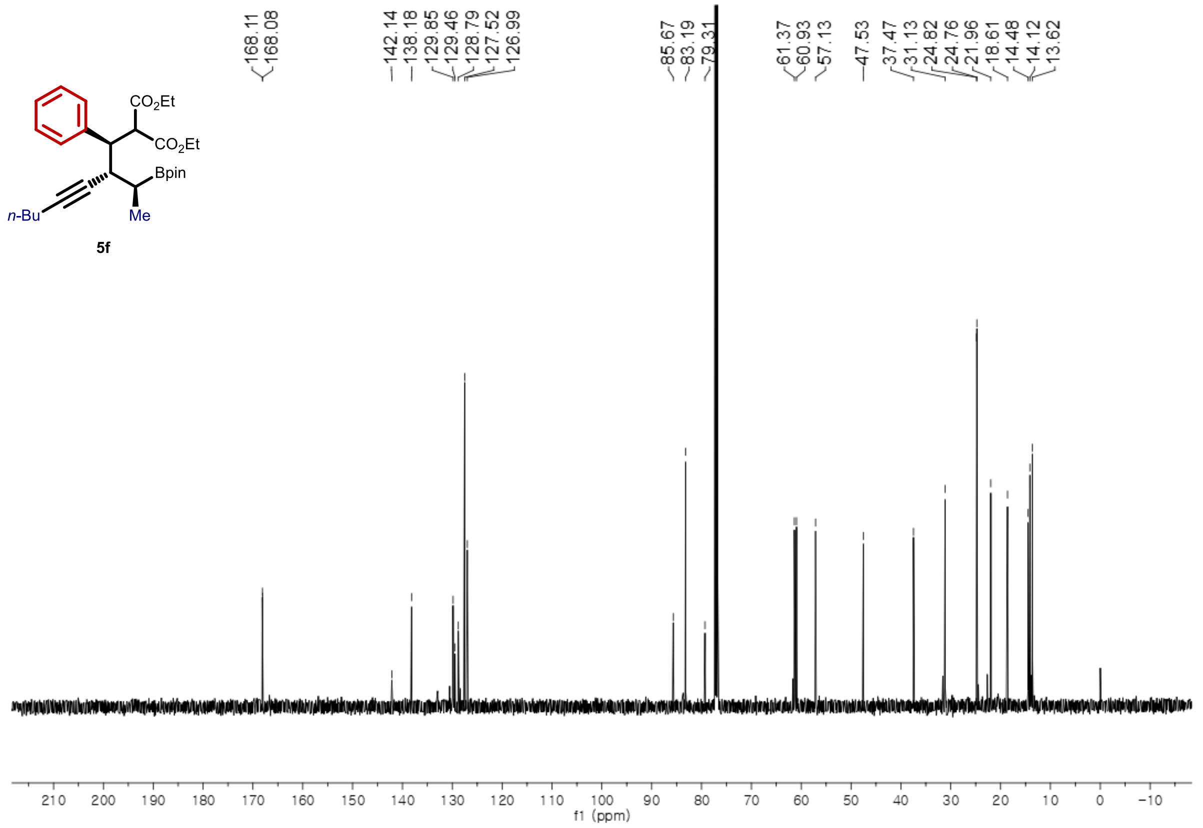

Supplementary Figure 74. ^{13}C NMR of compound **5f**.

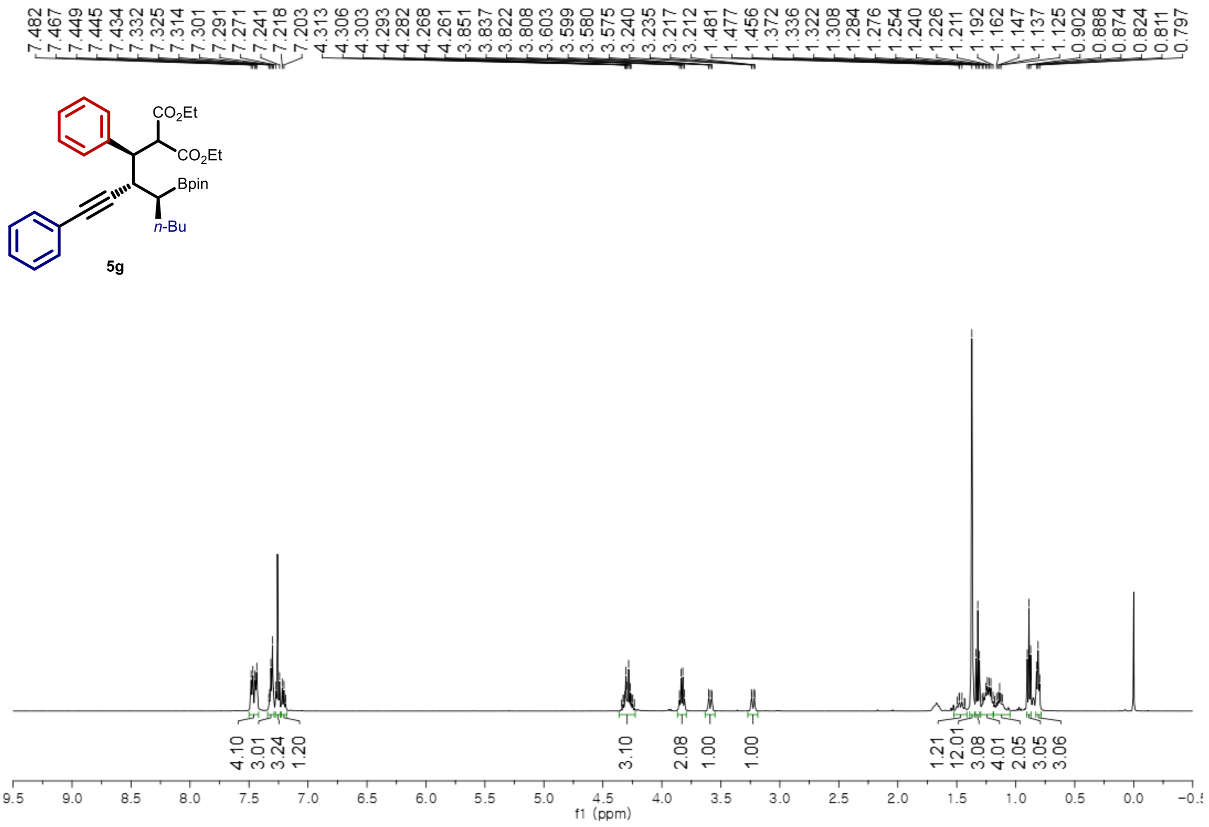

Supplementary Figure 75. ^{1}H NMR of compound **5g**.

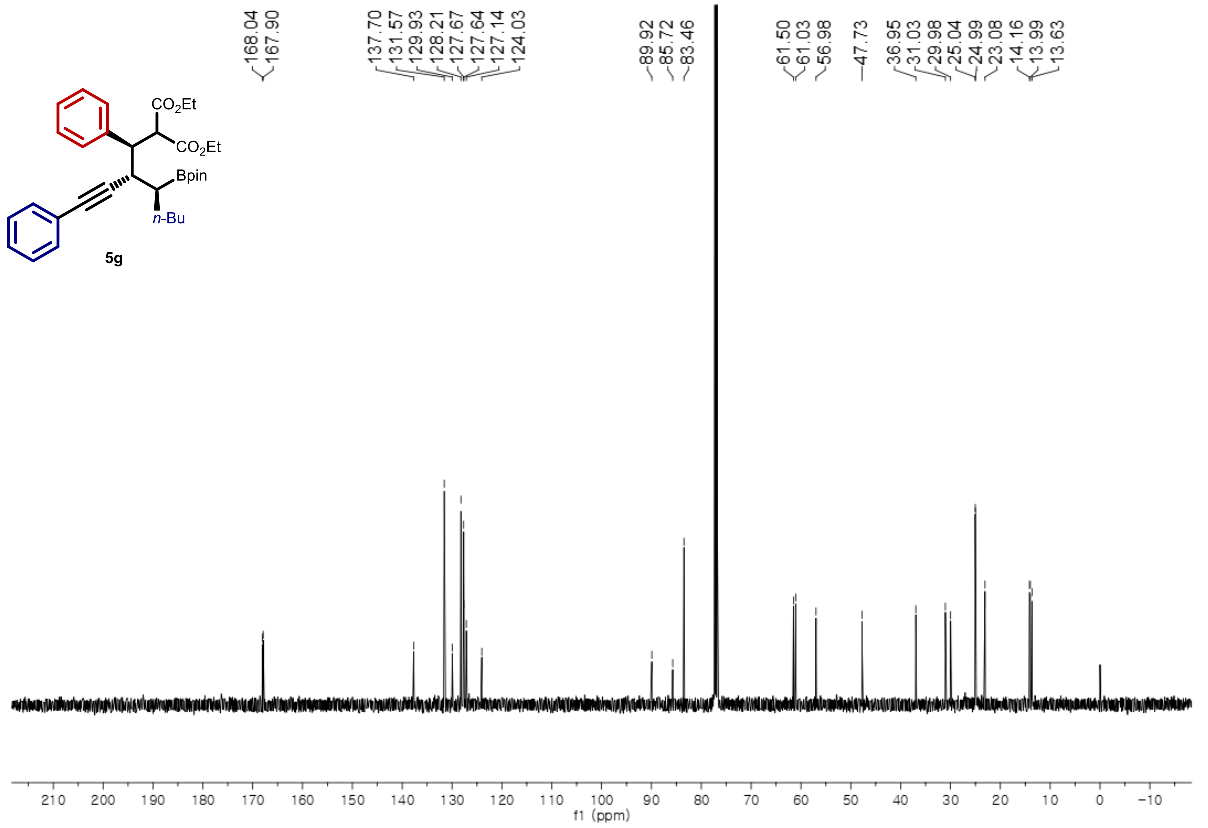

Supplementary Figure 76. ^{13}C NMR of compound **5g**.

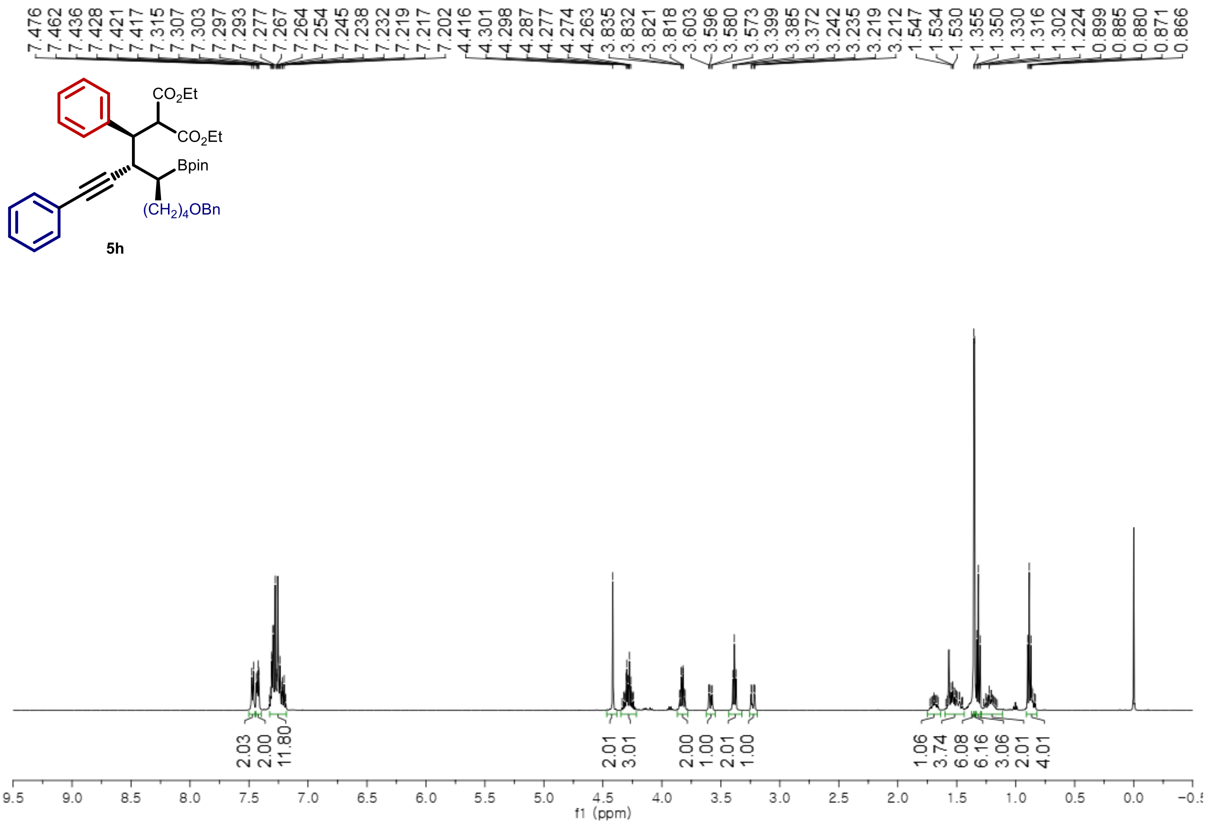

Supplementary Figure 77. ^{1}H NMR of compound **5h**.

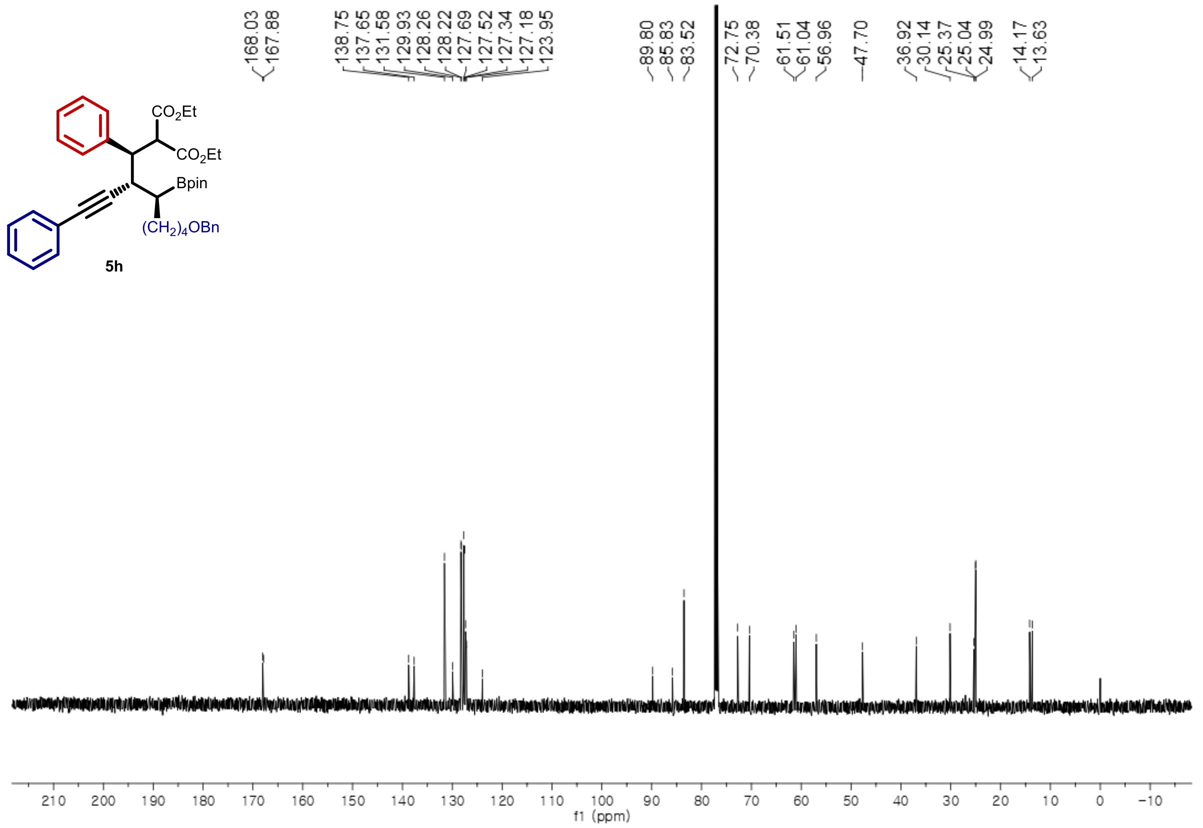

Supplementary Figure 78. ^{13}C NMR of compound **5h**.

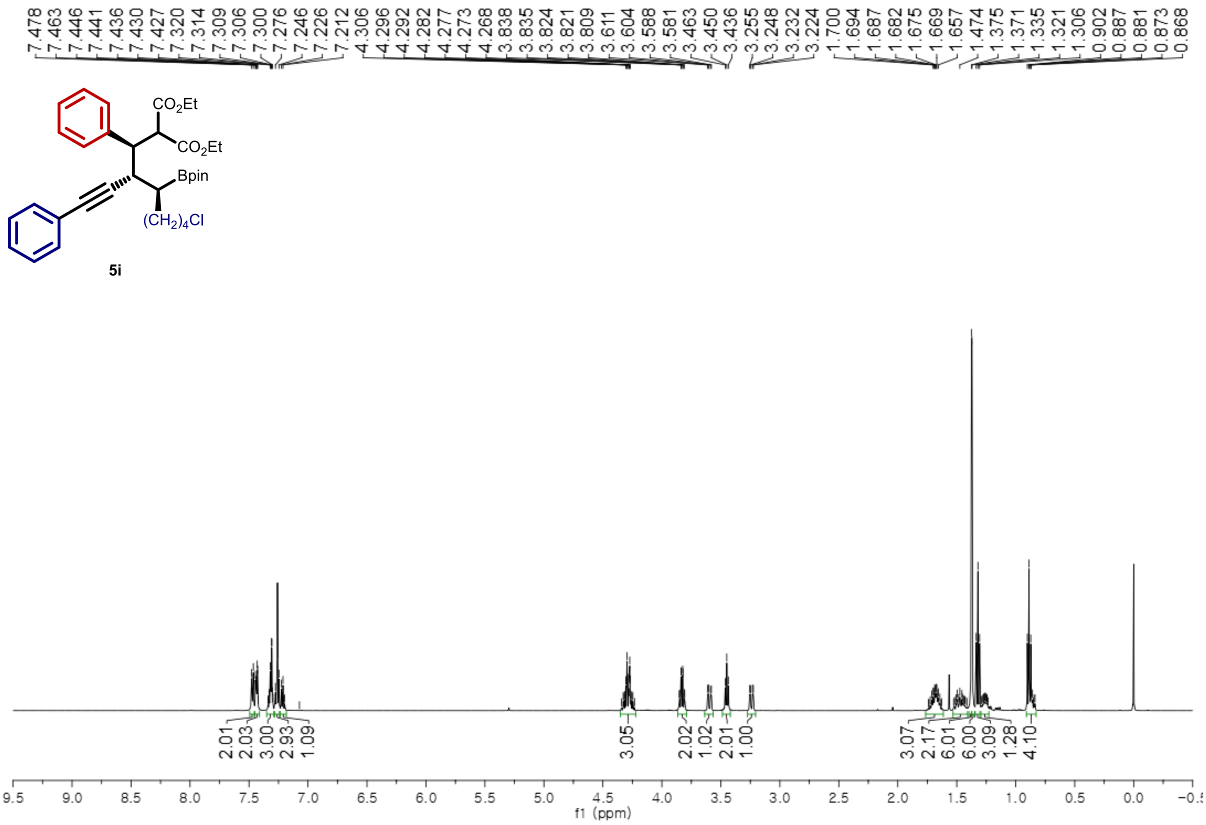

Supplementary Figure 79. ^{1}H NMR of compound **5i**.

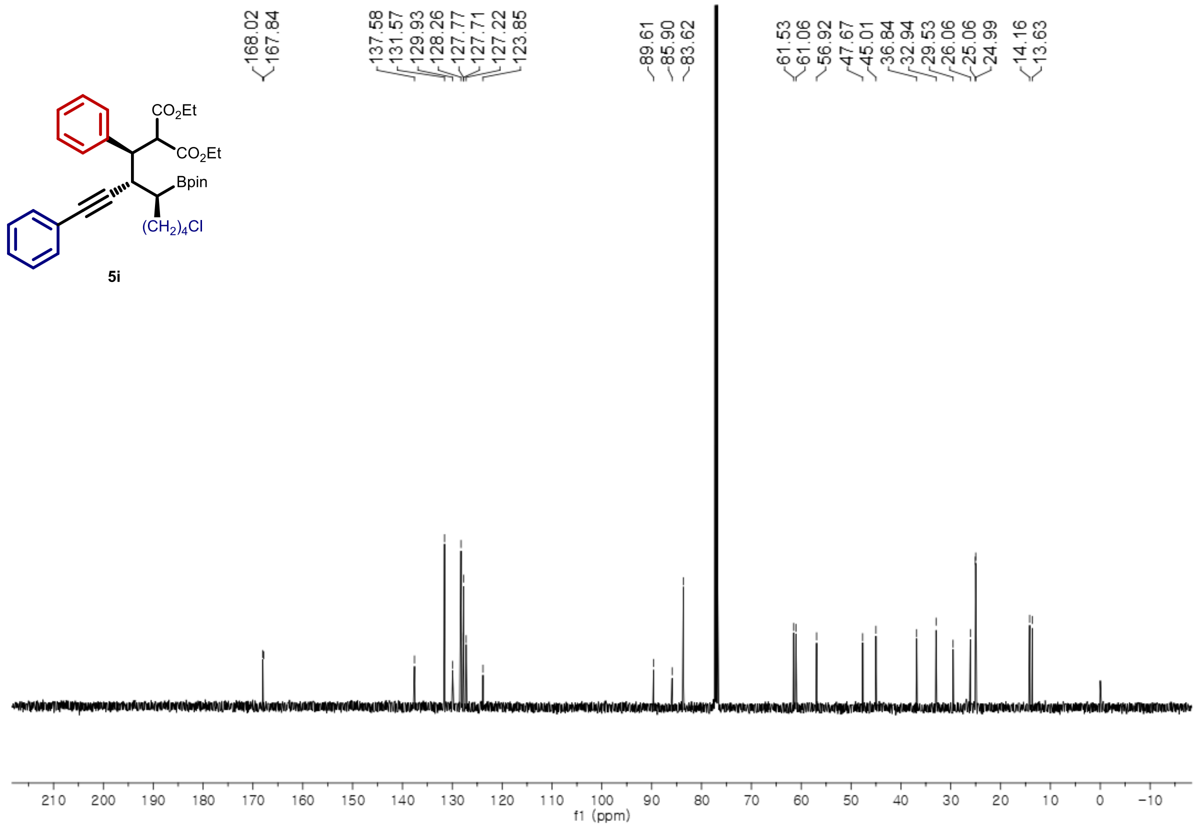

Supplementary Figure 80. ^{13}C NMR of compound **5i**.

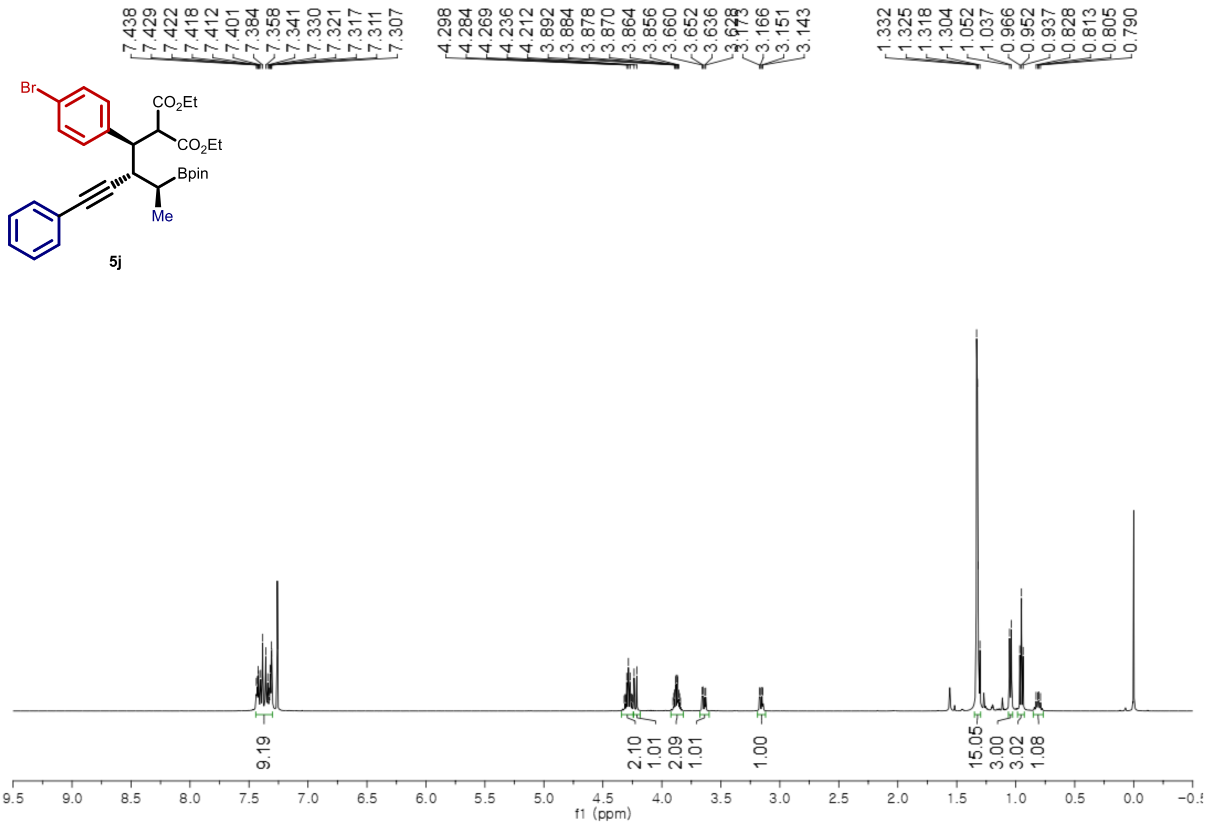

Supplementary Figure 81. ^{1}H NMR of compound **5j**.

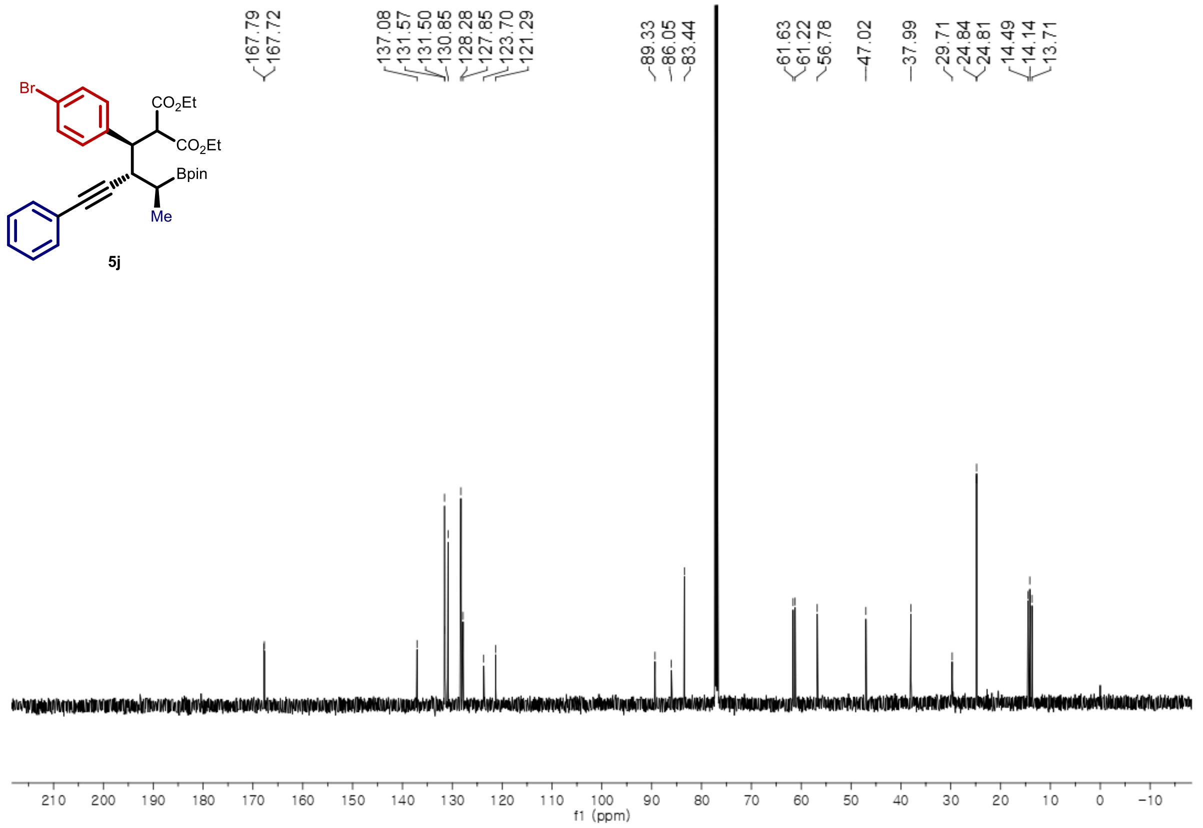

Supplementary Figure 82. ^{13}C NMR of compound **5j**.

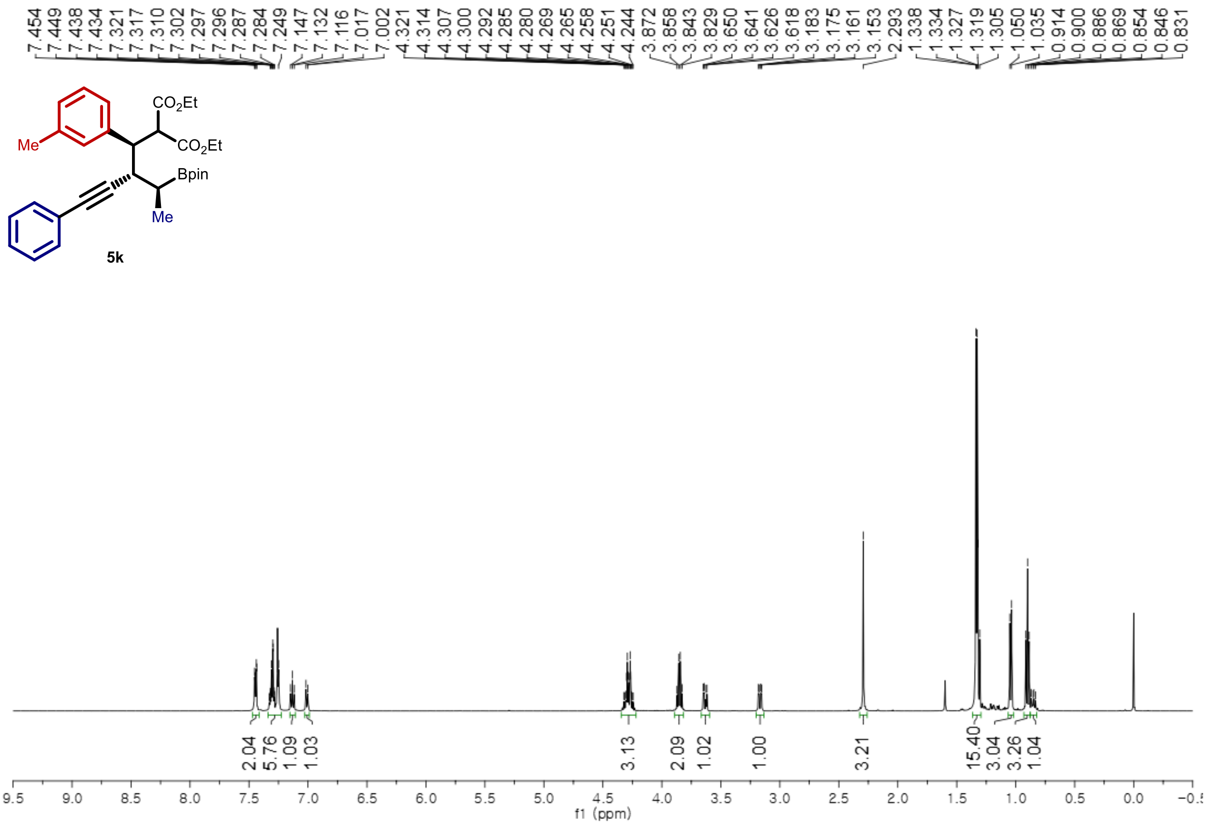

Supplementary Figure 83. ^{1}H NMR of compound **5k**.

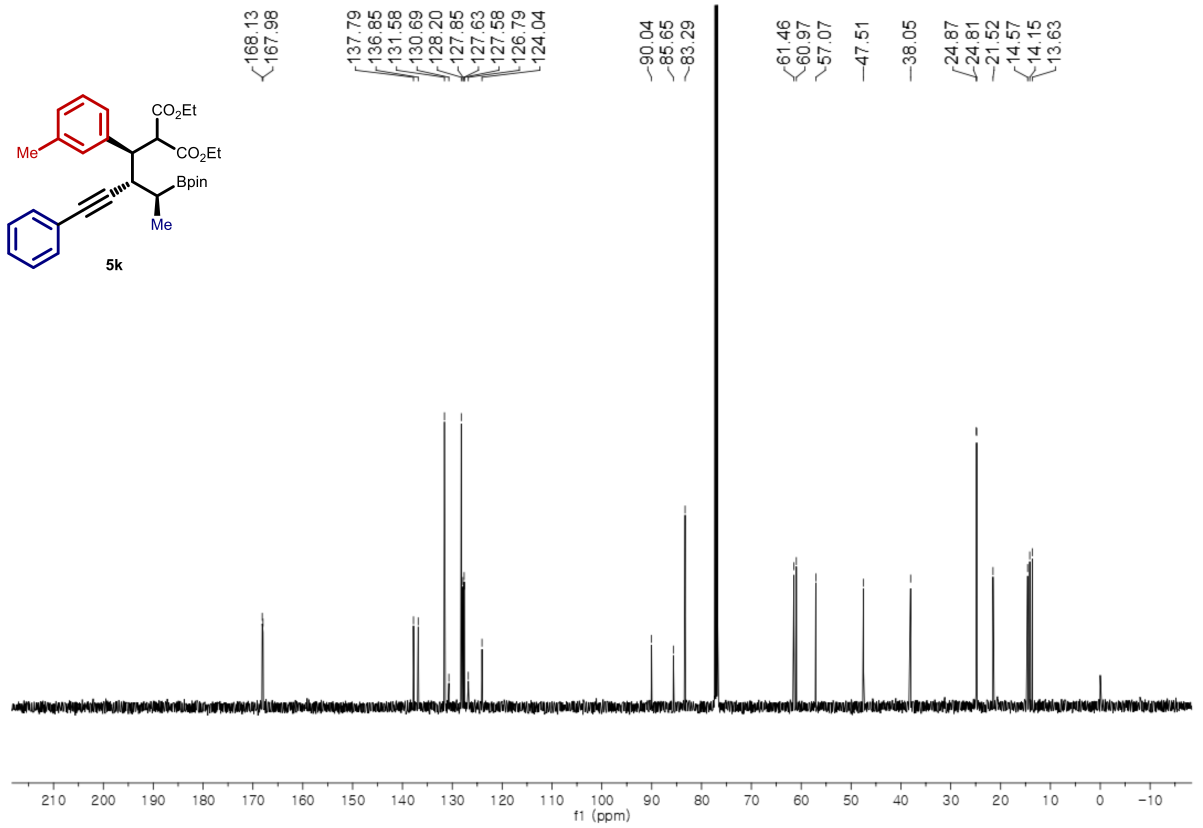

Supplementary Figure 84. ^{13}C NMR of compound **5k**.

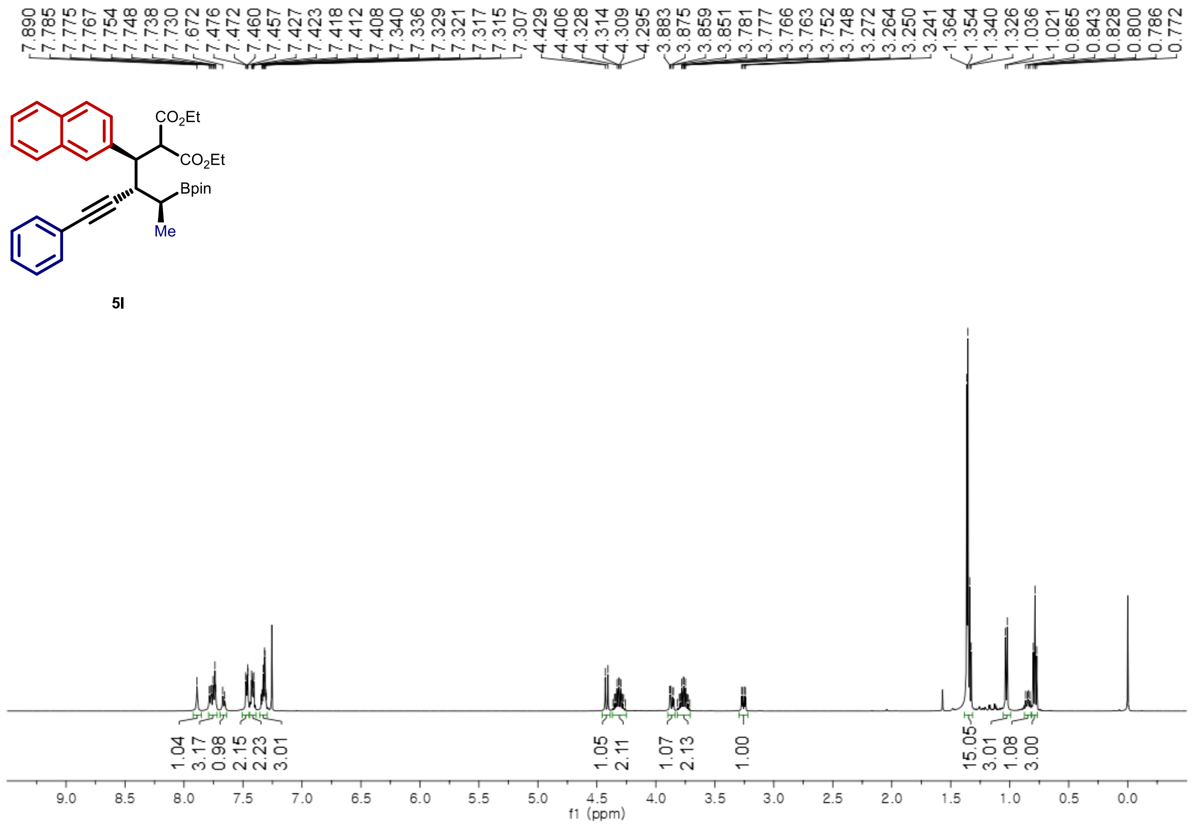

Supplementary Figure 85. ^{1}H NMR of compound **5l**.

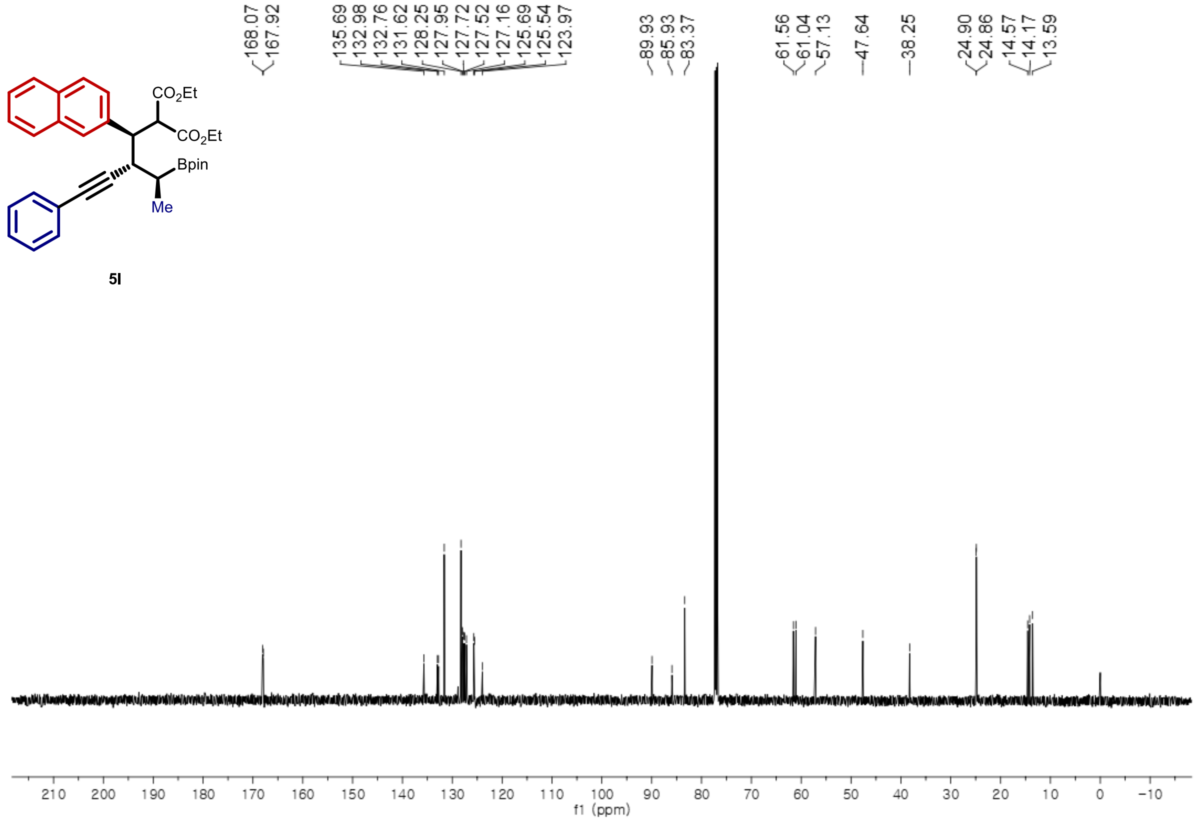

Supplementary Figure 86. ^{13}C NMR of compound **5l**.

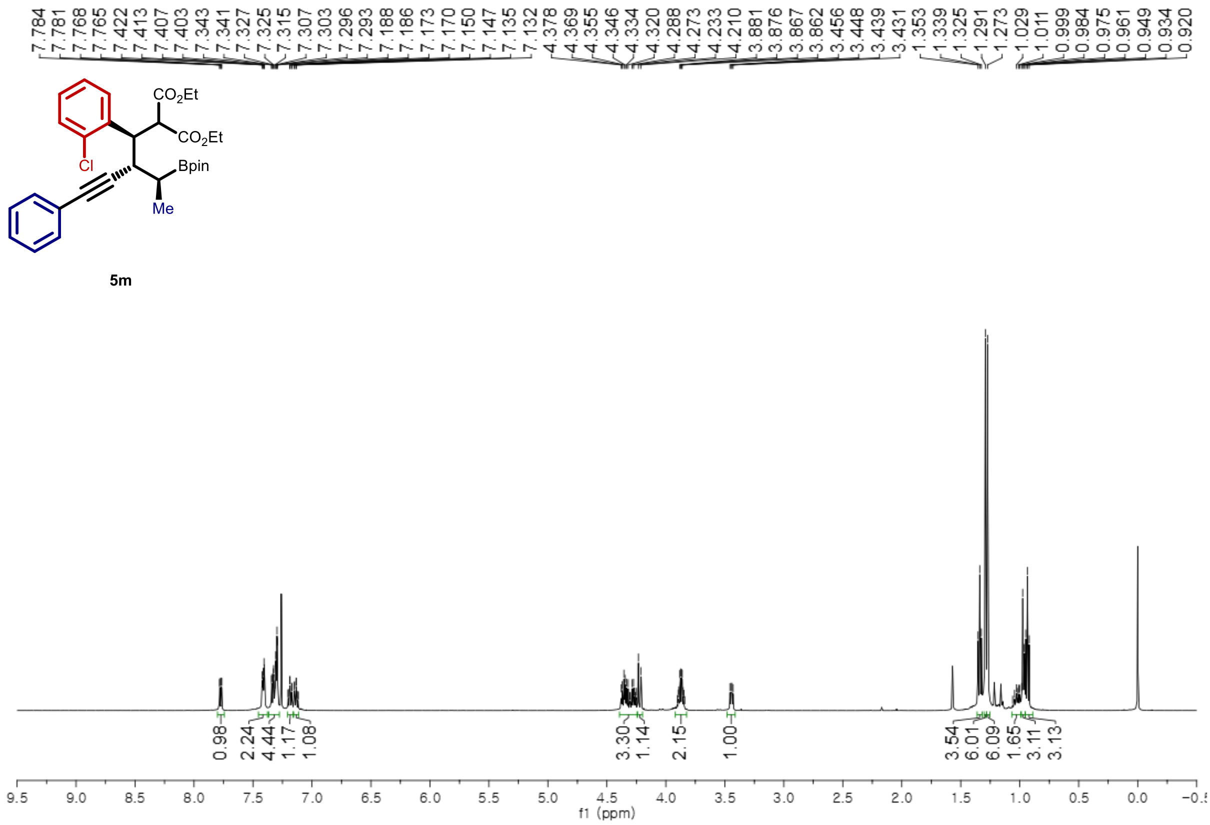

Supplementary Figure 87. ^{1}H NMR of compound **5m**.

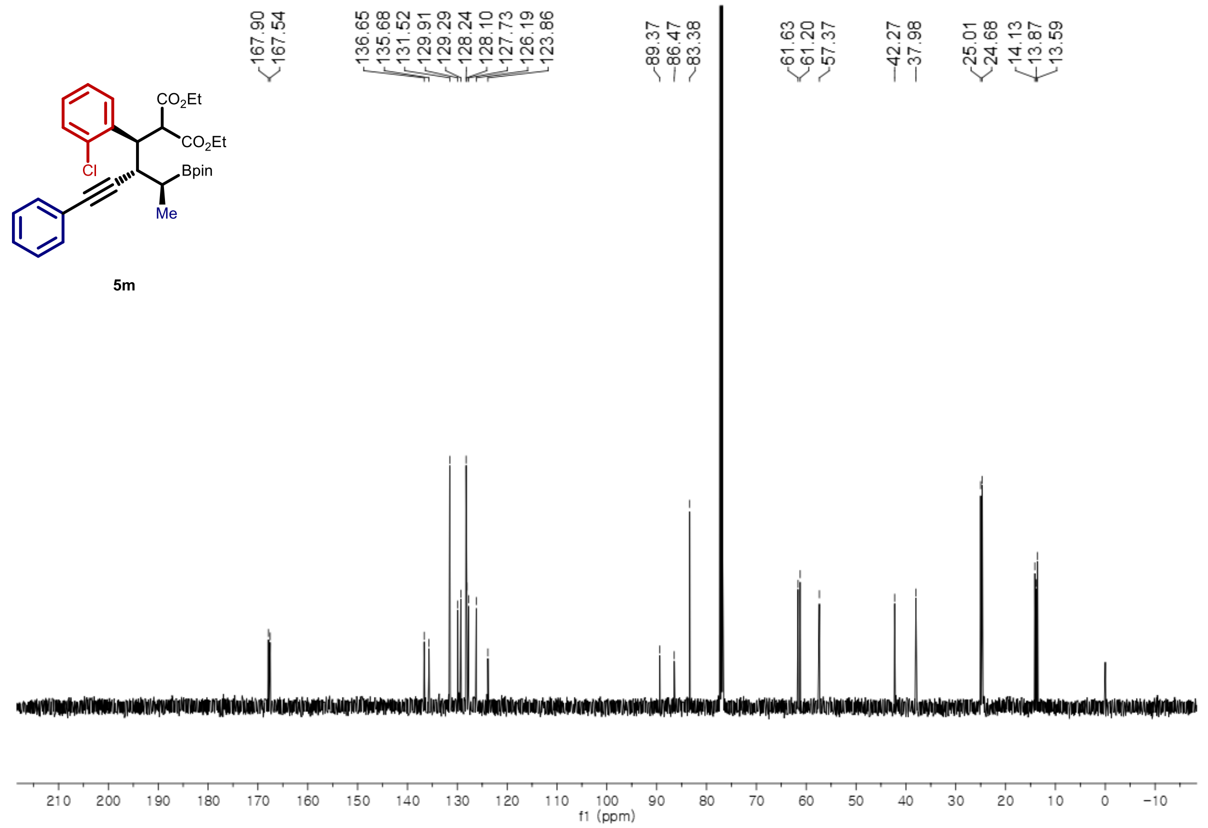

Supplementary Figure 88. ^{13}C NMR of compound **5m**.

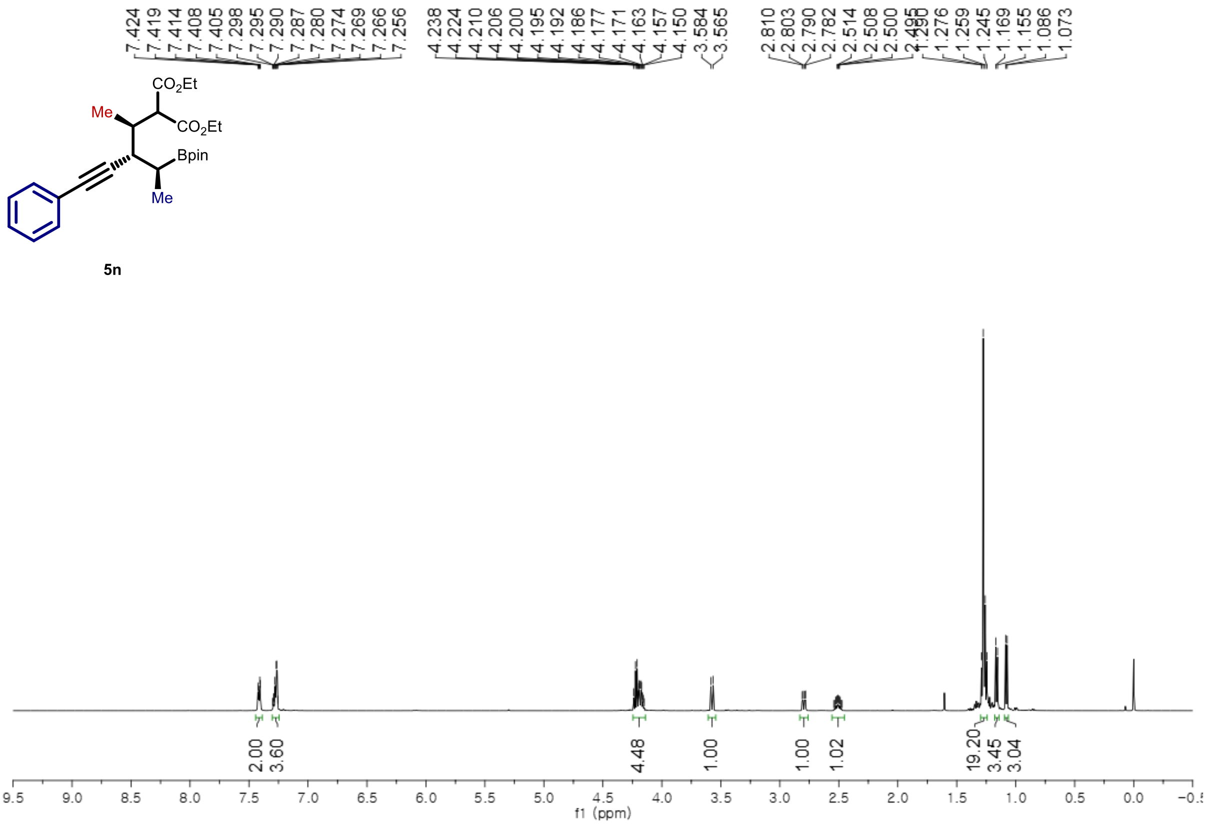

Supplementary Figure 89. ^1H NMR of compound **5n**.

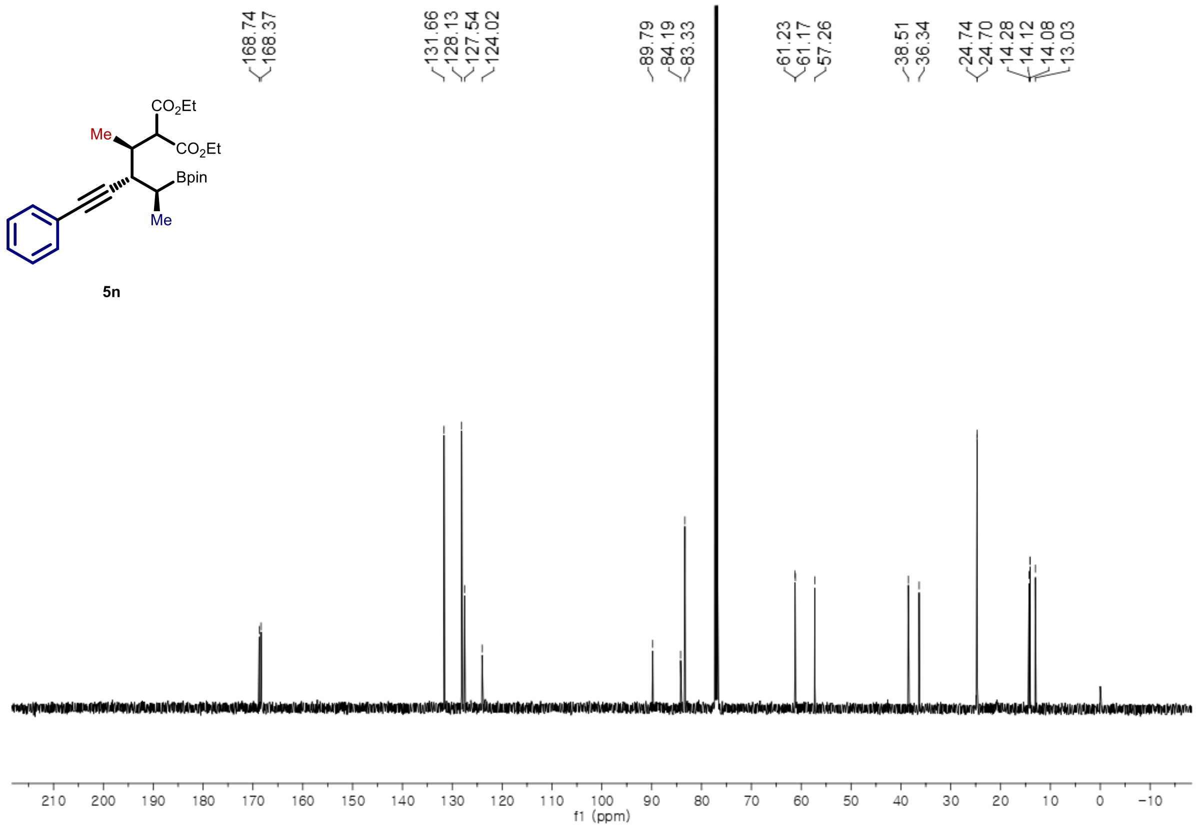

Supplementary Figure 90. ^{13}C NMR of compound **5n**.

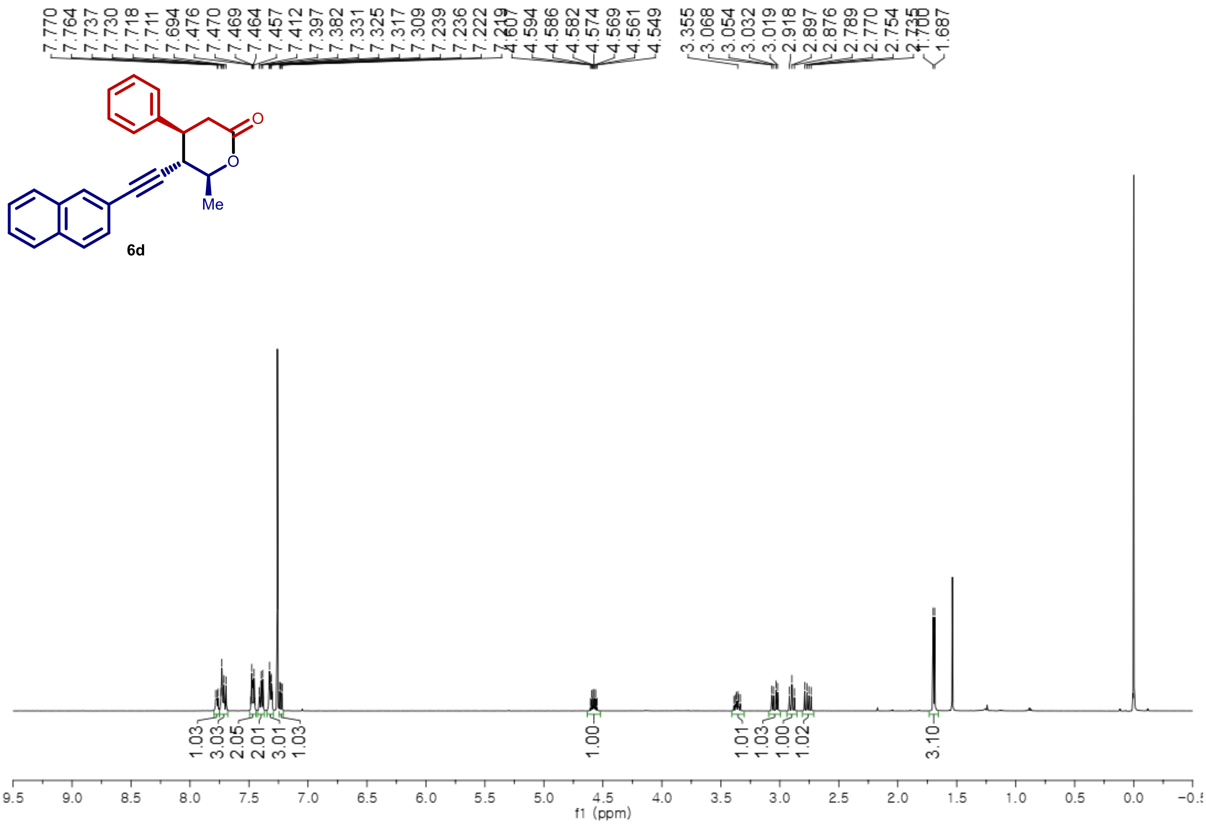

Supplementary Figure 91. ^{1}H NMR of compound **6d**.

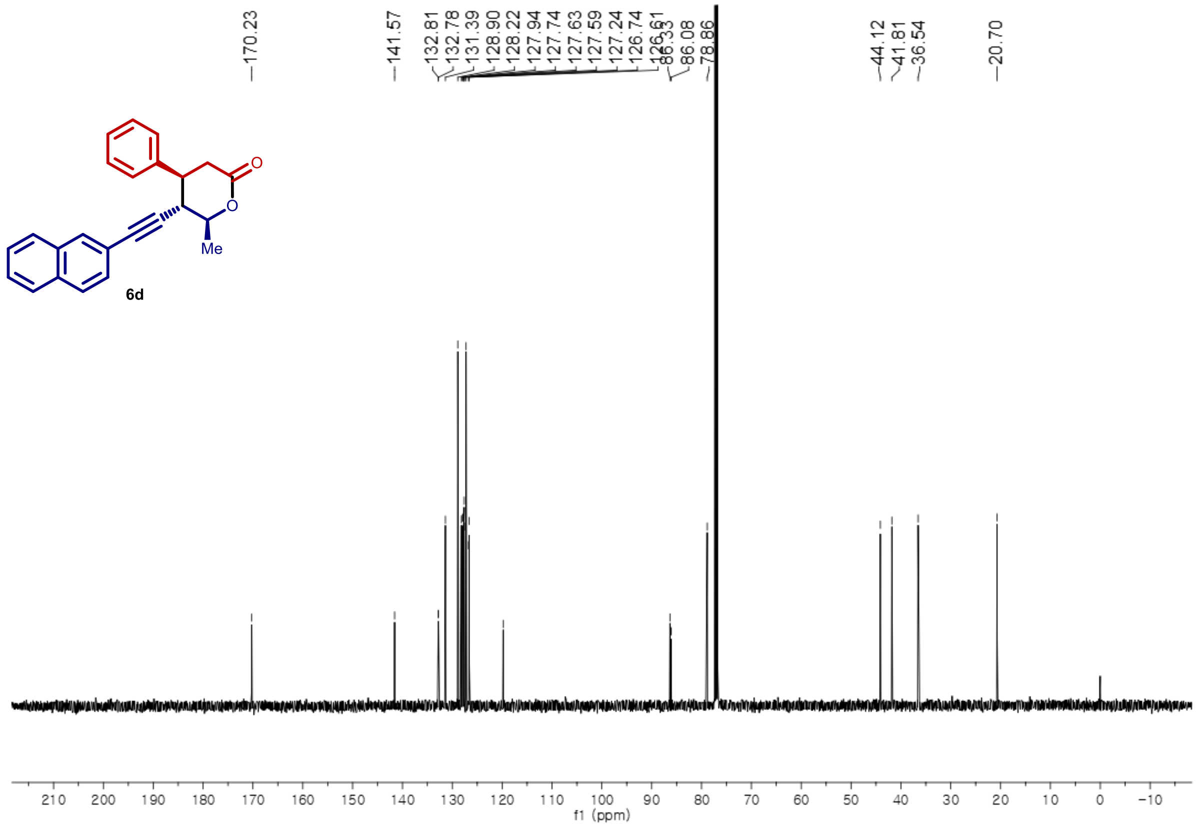

Supplementary Figure 92. ^{13}C NMR of compound **6d**.

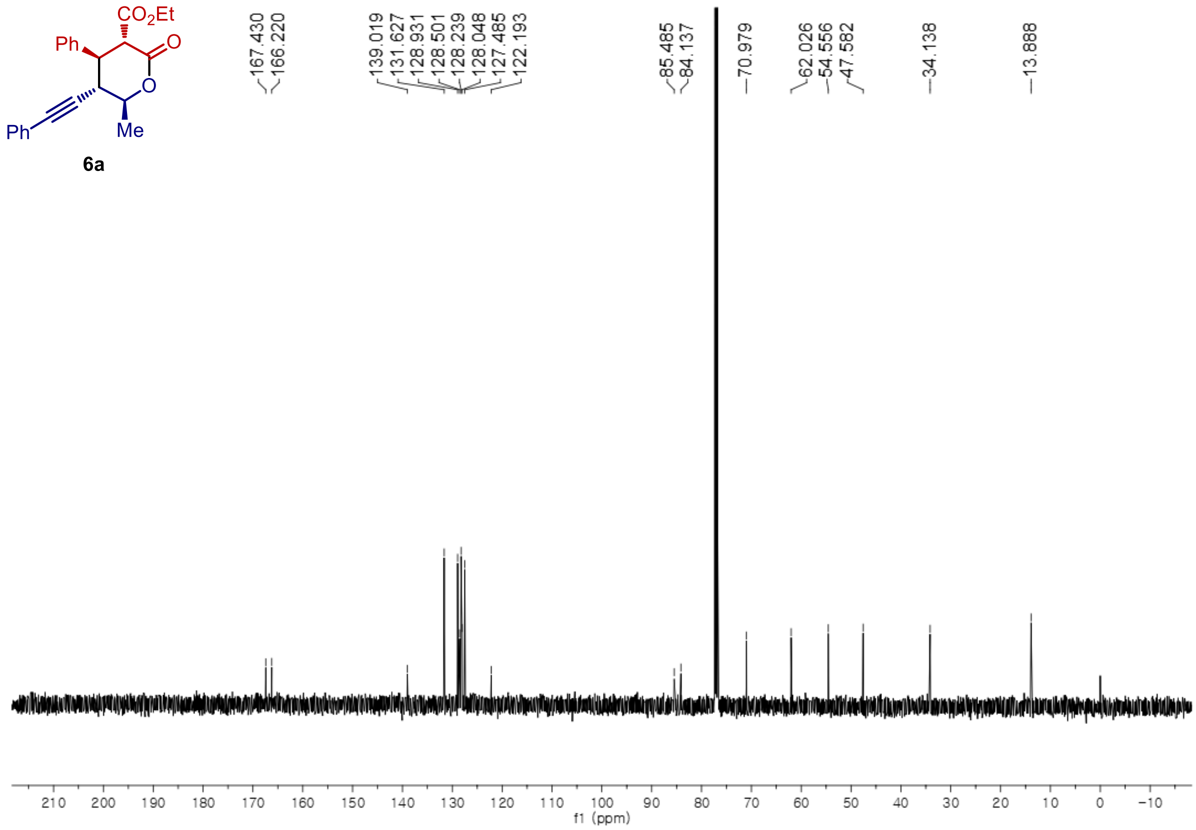

Supplementary Figure 93. ^{13}C NMR of compound **6a**.

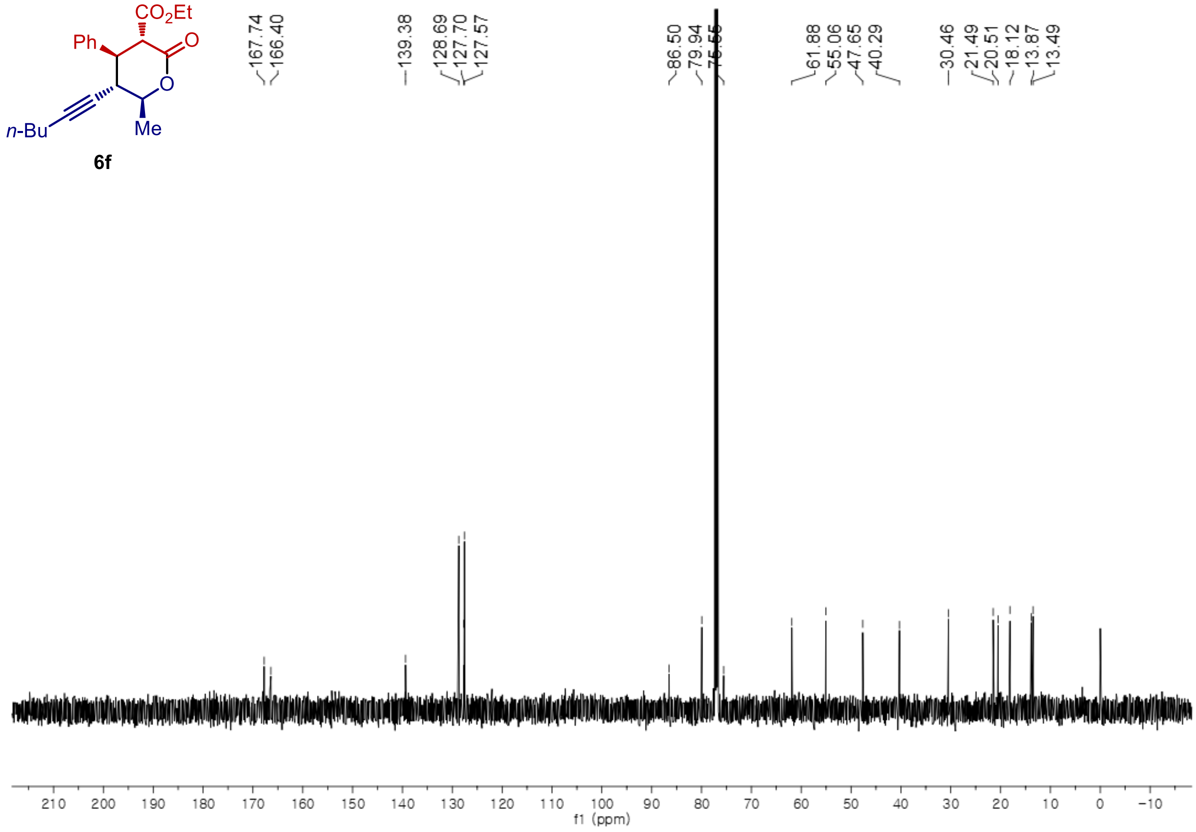

Supplementary Figure 94. ^{13}C NMR of compound **6f**.

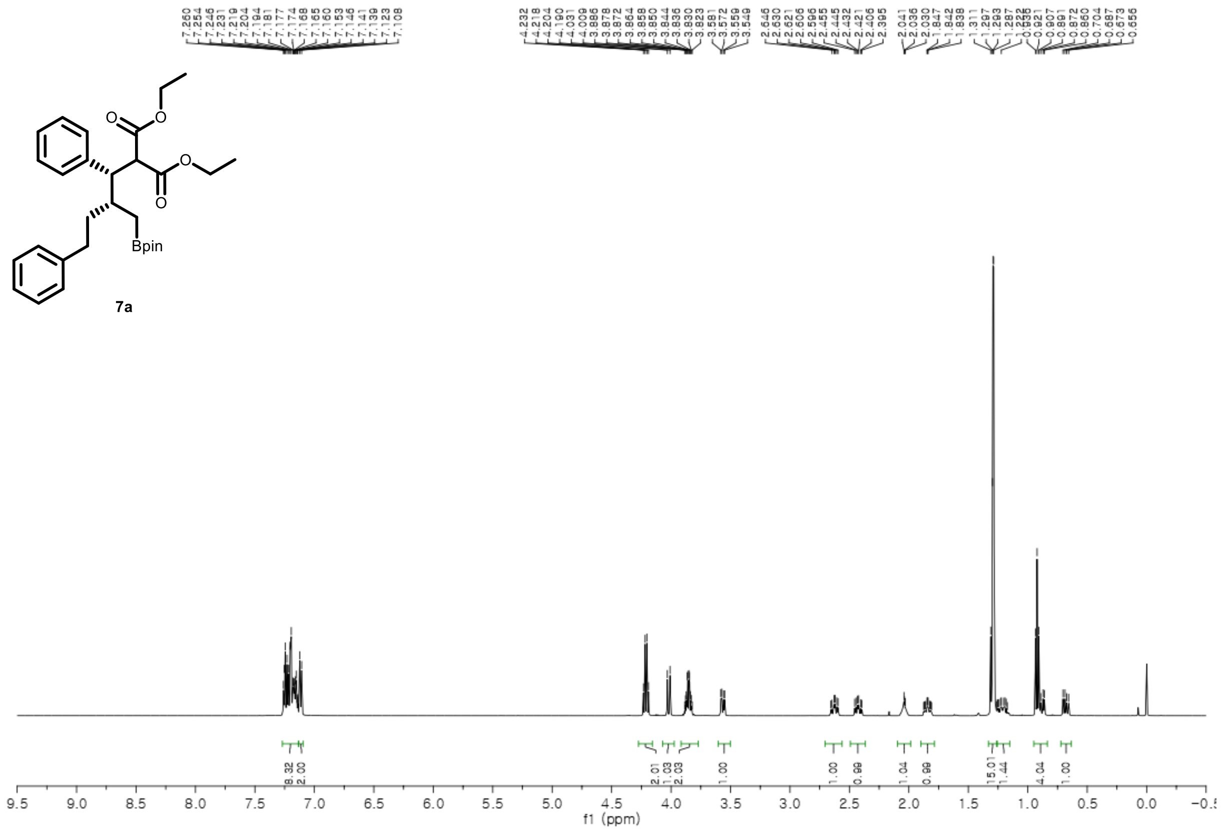

Supplementary Figure 95. ^{1}H NMR of compound **7a**.

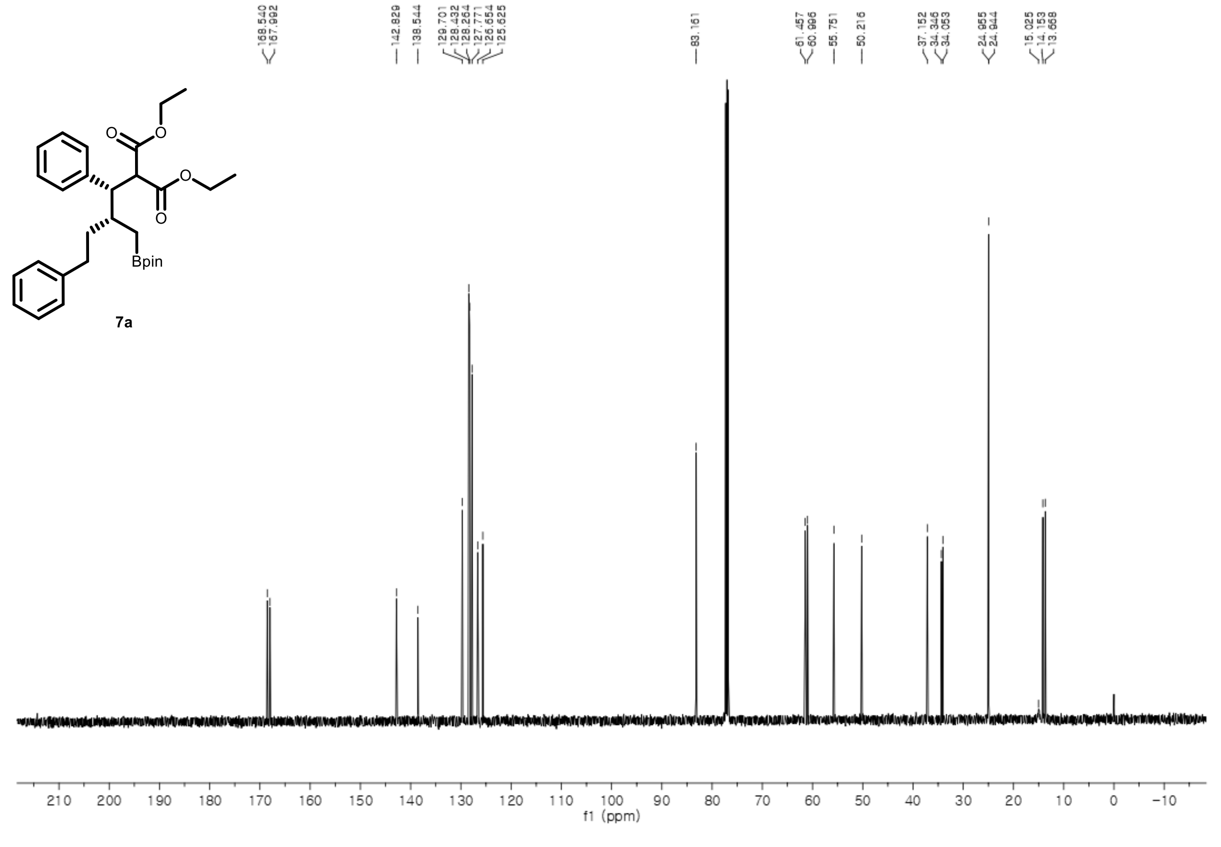

Supplementary Figure 96. ^{13}C NMR of compound **7a**.

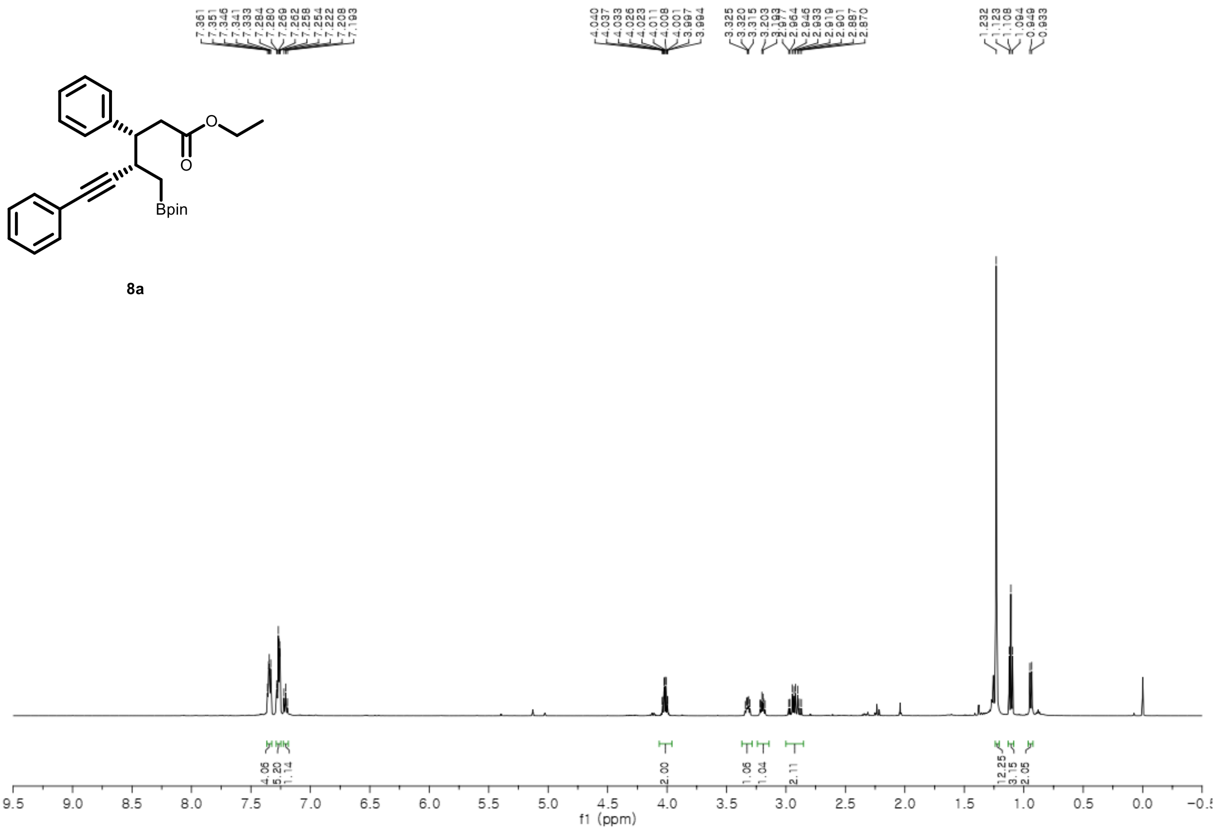

Supplementary Figure 97. ^{1}H NMR of compound **8a**.

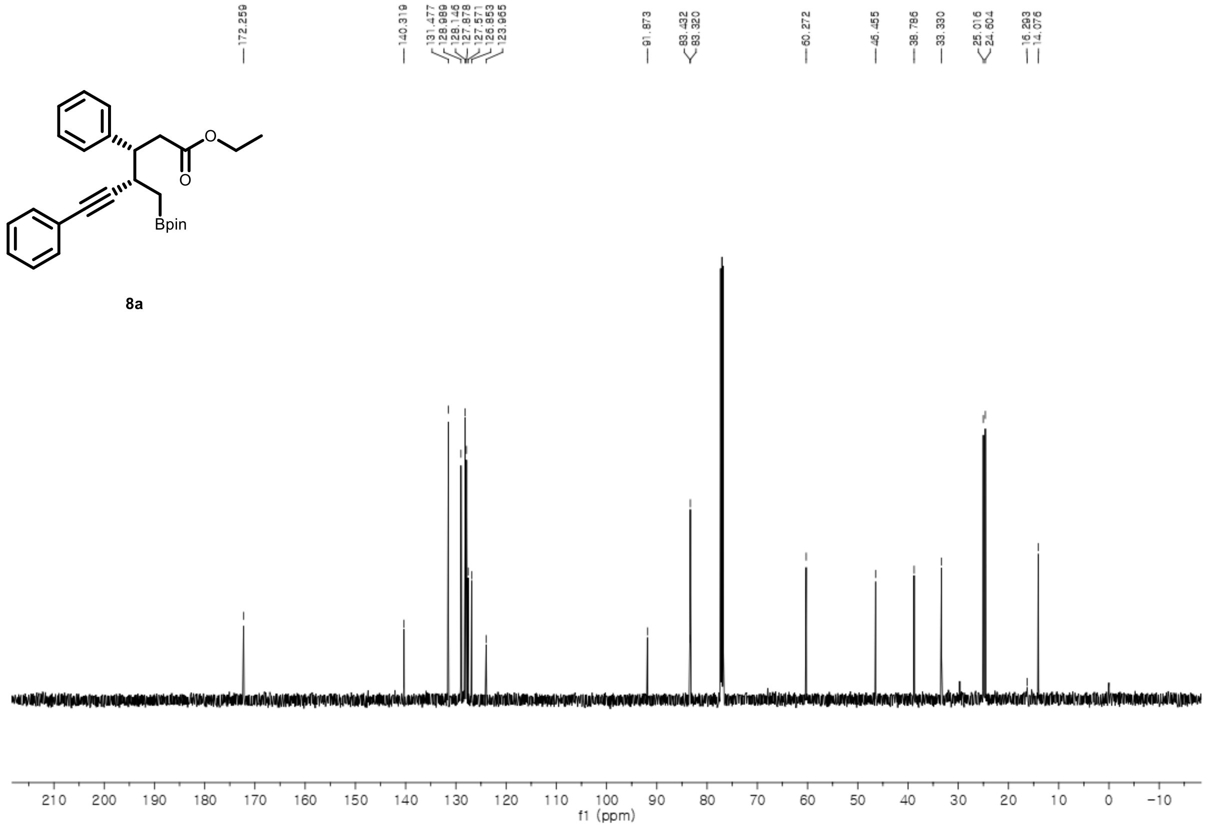

Supplementary Figure 98. ^{13}C NMR of compound **8a**.

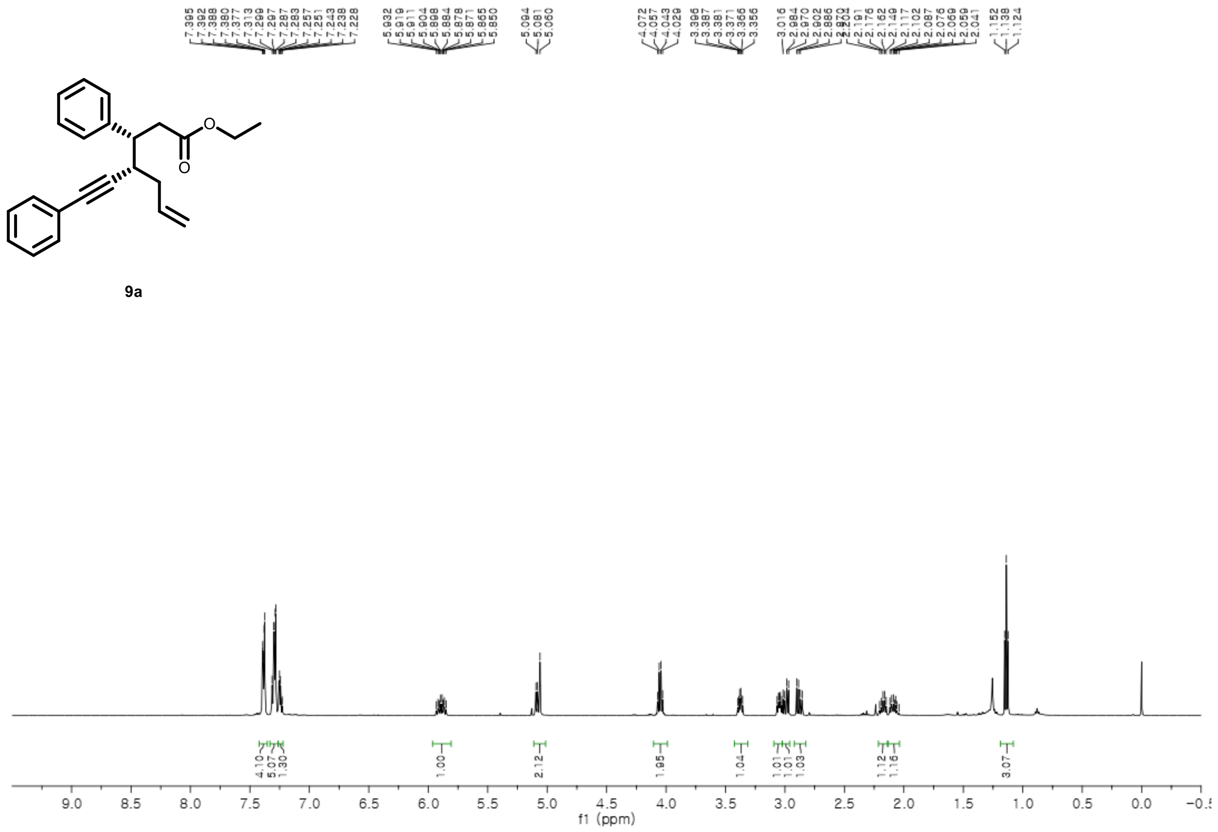

Supplementary Figure 99. ^{1}H NMR of compound **9a**.

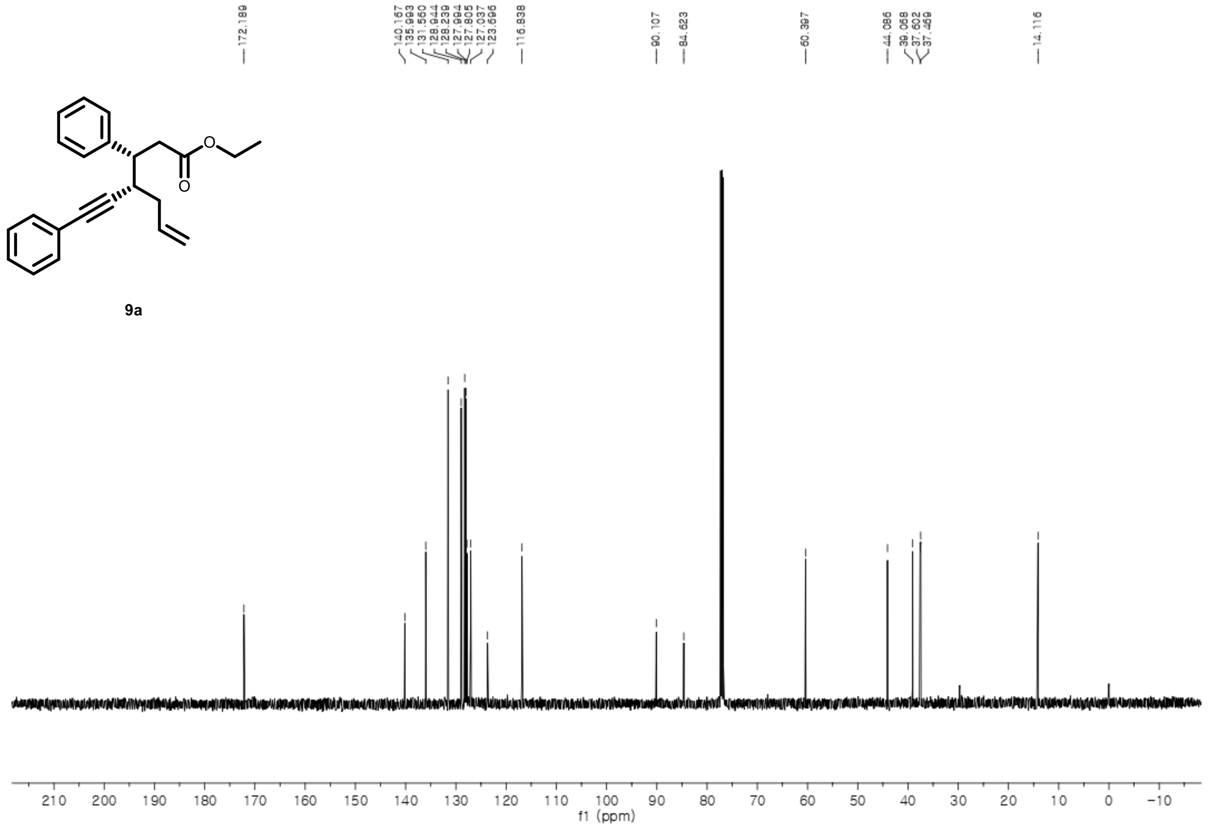

Supplementary Figure 100. ^{13}C NMR of compound **9a**.

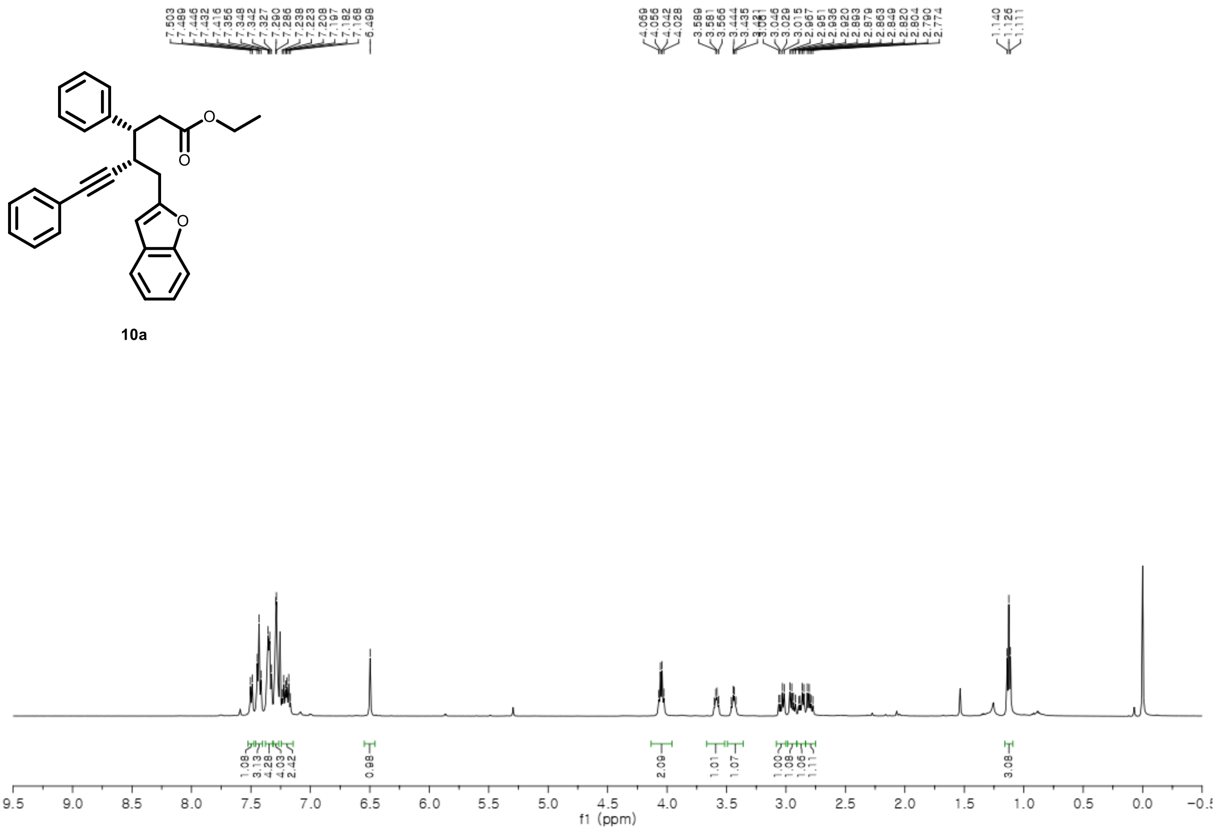

Supplementary Figure 101. ^{1}H NMR of compound **10a**.

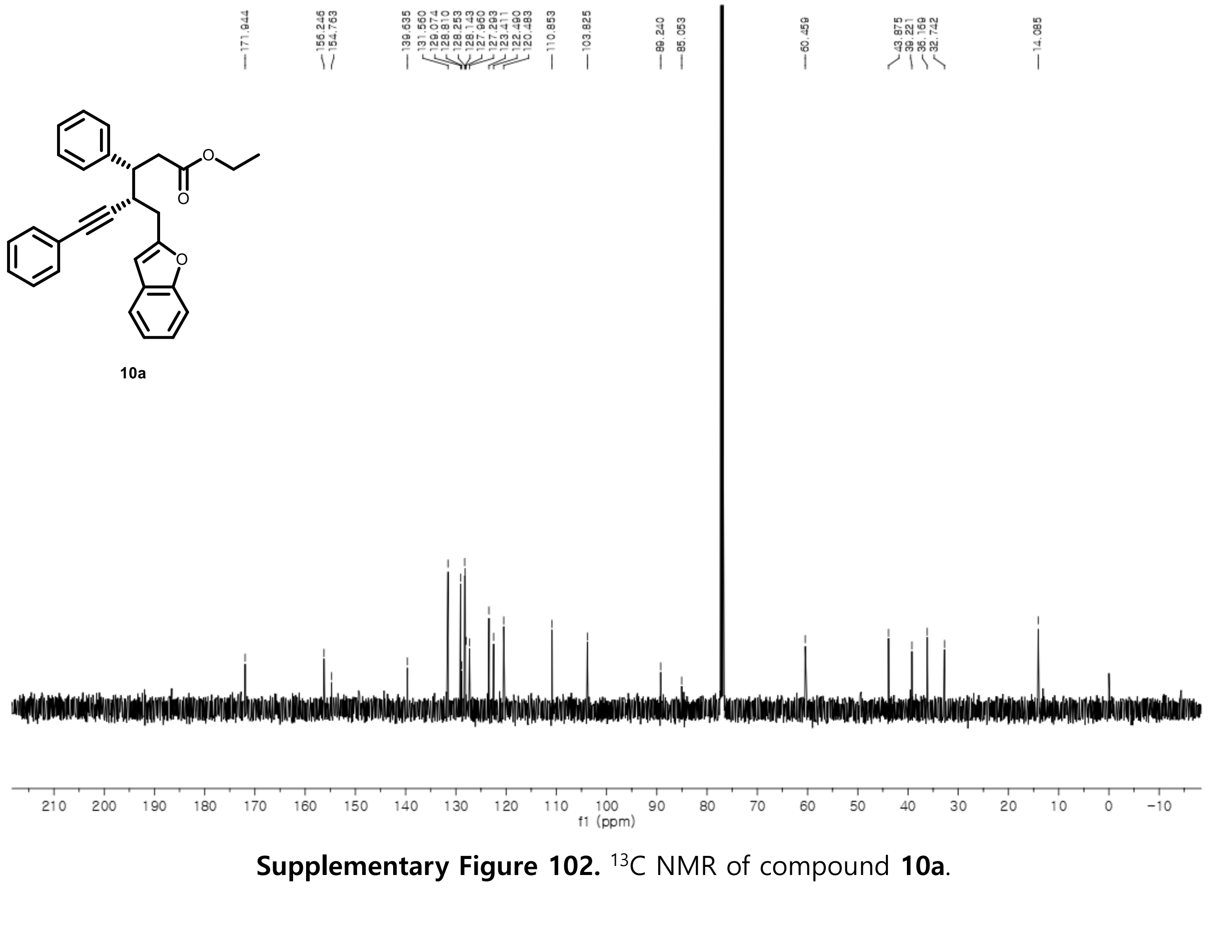

Supplementary Figure 102. ^{13}C NMR of compound **10a**.

IV. Supplementary References

1. Clavier, H., Coutable, L., Guillemin, J.-C. & Mauduit, M. New bidentate alkoxy-NHC ligands for enantioselective copper-catalysed conjugate addition. *Tetrahedron: Asymmetry* **16**, 921 (2005).
2. Clavier, H., Coutable, L., Toupet, L., Guillemin, J.-C. & Mauduit, M. Design and synthesis of new bidentate alkoxy-NHC ligands for enantioselective copper-catalyzed conjugate addition. *J. Organomet. Chem.* **690**, 5237 (2005).
3. Morandi, B., Cheang, J. & Carreira, E. M. Iron-catalyzed preparation of trifluoromethyl substituted vinyl- and alkynylcyclopropanes. *Org. Lett.* **13**, 3080 (2011).
4. Adamson, N. J., Jeddi, H. & Malcolmson, S. J. Preparation of chiral allenes through Pd-catalyzed intermolecular hydroamination of conjugated enynes: enantioselective synthesis enabled by catalyst design. *J. Am. Chem. Soc.* **141**, 8574 (2019).
5. Brown, H. C. & Gupta, S. K. Catecholborane (1,3,2-benzodioxaorole) as a new, general monohydroboration reagent for alkynes. Convenient synthesis of alkeneboronic esters and acids from alkynes via hydroboration. *J. Am. Chem. Soc.* **94**, 4370 (1972).
6. Jang, W. J., Lee, W. L., Moon, J. H., Lee, J. Y. & Yun, J. Copper-catalyzed trans-hydroboration of terminal aryl alkynes: stereodivergent synthesis of alkenylboron compounds. *Org. Lett.* **18**, 1390 (2016).
7. Vogler, T. & Studer, A. Applications of TEMPO in Synthesis. *Synthesis* **13**, 1979 (2008).
